# The presenting symptom signatures of incident cancer: evidence from the English 2018 National Cancer Diagnosis Audit

**DOI:** 10.1038/s41416-023-02507-4

**Published:** 2023-12-06

**Authors:** N. Zakkak, M. E. Barclay, R. Swann, S. McPhail, G. Rubin, G. A. Abel, G. Lyratzopoulos

**Affiliations:** 1 Epidemiology of Cancer Healthcare and Outcomes (ECHO) Group, Department of Behavioural Science and Health, Institute of Epidemiology and Health Care, University College London, London, UK; 2 National Disease Registration Service, NHS England, London, UK; 3 Cancer Intelligence, Cancer Research UK, London, UK; 4 Population Health Sciences Institute, Newcastle University, Newcastle Upon Tyne, UK; 5 Medical School, College of Medicine and Health, University of Exeter, St Luke’s Campus, Heavitree Road, Exeter, London, UK

**Keywords:** Cancer epidemiology, Cancer

## Abstract

**Background:**

Understanding relationships between presenting symptoms and subsequently diagnosed cancers can inform symptom awareness campaigns and investigation strategies.

**Methods:**

We used English National Cancer Diagnosis Audit 2018 data for 55,122 newly diagnosed patients, and examined the relative frequency of presenting symptoms by cancer site, and of cancer sites by presenting symptom.

**Results:**

Among 38 cancer sites (16 cancer groups), three classes were apparent: cancers with a dominant single presenting symptom (e.g. melanoma); cancers with diverse presenting symptoms (e.g. pancreatic); and cancers that are often asymptomatically detected (e.g. chronic lymphocytic leukaemia). Among 83 symptoms (13 symptom groups), two classes were apparent: symptoms chiefly relating to cancers of the same body system (e.g. certain respiratory symptoms mostly relating to respiratory cancers); and symptoms with a diverse cancer site case-mix (e.g. fatigue). The cancer site case-mix of certain symptoms varied by sex.

**Conclusion:**

We detailed associations between presenting symptoms and cancer sites in a large, representative population-based sample of cancer patients. The findings can guide choice of symptoms for inclusion in awareness campaigns, and diagnostic investigation strategies post-presentation when cancer is suspected. They can inform the updating of clinical practice recommendations for specialist referral encompassing a broader range of cancer sites per symptom.

## Introduction

Most cancer patients are diagnosed after the onset of symptoms relating to their disease. In the UK, more than 90% of all cancer patients are diagnosed symptomatically [1, 2]; the similar figure in the US is likely to exceed 80% [3]. Despite ongoing improvements in diagnostic technologies to support asymptomatic cancer detection through screening, most patients are expected to continue to be diagnosed symptomatically in the forthcoming decade [4]. Therefore, among other cancer control strategies, several interventions aimed to advance help-seeking (through raising awareness of possible cancer symptoms among members of the public), and prompt diagnostic investigation or referral of patients presenting with symptoms raising the suspicion of cancer, have been instigated [1, 4–6]. However, clinical guidelines supporting referrals currently chiefly relate to specific cancer sites, although many symptoms (particularly vague/non-organ specific ones) relate to a range of different cancers. In England, multi-specialty diagnostic centres have been developed to assess patients with vague/non-organ specific symptoms, but optimal investigation strategies, either pre-referral and within primary care, or post-referral are unclear [7]. Decisions on choice of target symptoms in public awareness campaigns have traditionally been made based on clinical consensus about associations of a given symptom with different cancers [8]. Attaining such evidence can help to improve the design and evaluation strategies for both symptom awareness campaigns, and clinical practice recommendations for use of primary care investigations or referrals. Information from common blood tests can support the diagnostic process in primary care, but such tests are used in fewer than half of all cancer patients, with large variation by presenting symptoms and cancer site [9]. For these reasons, a fuller understanding of the bidirectional relationships between presenting symptoms and cancer sites is needed.

Against this background, we aimed to first examine the relative frequency of presenting symptoms by cancer site (the ‘symptom signature’ of each cancer site), and second to examine the relative frequency of cancer sites by presenting symptom (the ‘cancer site case-mix’ of each symptom), among incident cancer cases.

## Methods

### Data and study population

**Fig. 1:**
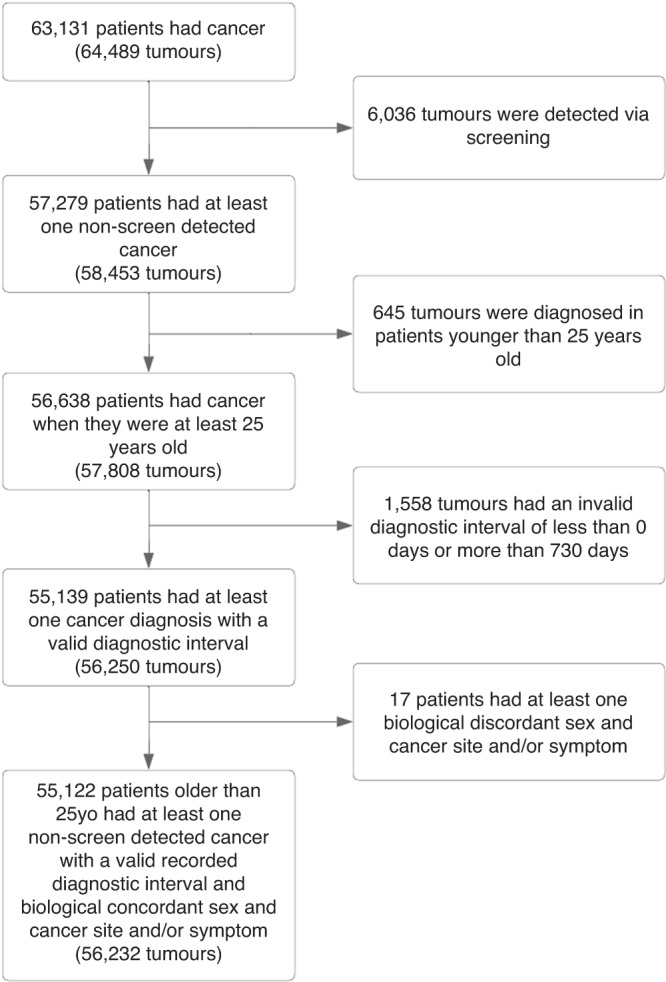
Flowchart describing the process of cohort selection. Diagnostic interval: interval between date of first presentation to GP and date of cancer diagnosis.

Data from the English National Cancer Diagnosis Audit (NCDA) 2018 was analysed. Details of the NCDA methodology have been described previously [2]. Briefly, incident cancer cases diagnosed in 2018, recorded by NHS England National Disease Registration Service, were assigned to the participating general practices which the patient was registered with at the time of their diagnosis. General practitioners completed a questionnaire about the diagnostic process of each patient. 1878 general practices (26% of all practices) chose to take part in the audit, gathering data on 64,489 malignant tumours (20% of incident cancers in 2018), excluding non-melanoma skin cancer. Participating practices were similar to non-participating practices regarding the characteristics of registered populations, practice performance metrics and quality of patient experience. Patients included in the NCDA had similar characteristics (age and sex), cancer types, and cancer stages compared to the incident cohort of cancer patients in England. Patients whose cancer was screening-detected were excluded from analysis, as were patients aged 24 or younger. In patients with more than one tumour diagnosed in 2018, the tumour with the more advanced stage was chosen, or randomly if stage category was missing or identical. The derivation of the analysis sample is described in Fig. 1.

Data were available on 38 cancer sites, which were further categorised into the following cancer site groups: head and neck, upper gastrointestinal (GI), lower GI, hepato-pancreato-biliary (HPB), respiratory, urological, haematological, central nervous system (CNS), sarcoma, skin, ocular, breast, gynaecological, and prostate and other male organs. Two further cancer site groups included cancer sites with a small sample size (<35 cases) that could not be incorporated into other groups as ‘other malignant neoplasms’, and cancers of unknown primary sites (ICD-10 codes C77-C80), comprising a total of 16 cancer site groups. A full list of ICD-10 codes can be found in Supplementary Table 1.

Within NCDA, GPs could record one or more presenting symptoms from a drop-down menu of 83 symptom categories (including a ‘not applicable’ (N/A) and a ‘not known’ (N/K) group). Each of the 83 symptom categories was assigned to one of 14 higher-level groups comprising upper abdominal, lower abdominal, breast, CNS, lump/mass/lymph node, musculoskeletal pain, respiratory, skin lesion, ulceration, urological, female-organ specific, male-organ specific, non-specific symptoms, and no symptoms recorded. The non-specific group included symptoms without organ-specificity (e.g. fever, or fatigue) (Supplementary Table 2).

### Analysis

First, to examine the symptom signature of each cancer site, the proportion of individual symptoms that patients presented with by cancer site was calculated, ignoring combinations in patients presenting with more than one symptom. To further assess presenting symptom burden in individual cancer sites, the mean number of recorded symptoms per patient was calculated, alongside the number of symptoms that occurred in more than 1% and 50% of cases.

Second, to examine cancer site case-mix of each symptom among incident cancer patients, the proportion of patients diagnosed with each cancer site was calculated within each symptom. For both symptom signatures and cancer site case-mix, relevant proportions were calculated alongside their corresponding 95% confidence intervals.

## Results

### Sample description

Among 55,122 patients included in the analysis (Fig. 1), 29,841 (54%) were men and 21,597 (39%) were 60–74 years old. Sample composition by demographic characteristics and cancer site are shown in Table 1. For 11,066 (20%) patients, no presenting symptom was recorded.

**Table 1:** Sample characteristics (sex and age) of patients by cancer site.

	Men				Women			
Cancer Site	*N*	<60	60–74	75+	*N*	<60	60–74	75+
Total	29841	6000 (20.1%)	13182 (44.2%)	10659 (35.7%)	25281	7389 (29.2%)	8415 (33.3%)	9477 (37.5%)
Head and neck								
Larynx	296	73 (24.7%)	141 (47.6%)	82 (27.7%)	72	26 (36.1%)	29 (40.3%)	17 (23.6%)
Oral cavity	335	125 (37.3%)	143 (42.7%)	67 (20.0%)	250	75 (30.0%)	92 (36.8%)	83 (33.2%)
Oropharynx	497	232 (46.7%)	227 (45.7%)	38 (7.65%)	156	75 (48.1%)	62 (39.7%)	19 (12.2%)
Thyroid	186	96 (51.6%)	64 (34.4%)	26 (14.0%)	447	322 (72.0%)	89 (19.9%)	36 (8.05%)
Other head and neck	305	100 (32.8%)	130 (42.6%)	75 (24.6%)	176	62 (35.2%)	63 (35.8%)	51 (29.0%)
Upper gastrointestinal								
Oesophagus	979	185 (18.9%)	455 (46.5%)	339 (34.6%)	412	57 (13.8%)	151 (36.7%)	204 (49.5%)
Stomach	644	120 (18.6%)	222 (34.5%)	302 (46.9%)	385	69 (17.9%)	122 (31.7%)	194 (50.4%)
Lower gastrointestinal								
Anal	91	33 (36.3%)	34 (37.4%)	24 (26.4%)	174	63 (36.2%)	64 (36.8%)	47 (27.0%)
Colon	2195	420 (19.1%)	750 (34.2%)	1025 (46.7%)	2014	356 (17.7%)	585 (29.0%)	1073 (53.3%)
Rectum	1014	236 (23.3%)	389 (38.4%)	389 (38.4%)	627	169 (27.0%)	201 (32.1%)	257 (41.0%)
Small intestine	132	36 (27.3%)	55 (41.7%)	41 (31.1%)	124	30 (24.2%)	44 (35.5%)	50 (40.3%)
*Hepato-pancreato-biliary (HPB)*								
Liver	612	97 (15.8%)	297 (48.5%)	218 (35.6%)	317	52 (16.4%)	103 (32.5%)	162 (51.1%)
Pancreas	872	150 (17.2%)	360 (41.3%)	362 (41.5%)	853	120 (14.1%)	304 (35.6%)	429 (50.3%)
Other HPB	174	18 (10.3%)	77 (44.3%)	79 (45.4%)	251	29 (11.6%)	84 (33.5%)	138 (55.0%)
Respiratory								
Lung	3905	449 (11.5%)	1762 (45.1%)	1694 (43.4%)	3703	454 (12.3%)	1589 (42.9%)	1660 (44.8%)
Mesothelioma	393	6 (1.53%)	167 (42.5%)	220 (56.0%)	71	8 (11.3%)	24 (33.8%)	39 (54.9%)
Urological								
Bladder	1104	93 (8.42%)	410 (37.1%)	601 (54.4%)	414	45 (10.9%)	124 (30.0%)	245 (59.2%)
Kidney	1054	330 (31.3%)	453 (43.0%)	271 (25.7%)	641	170 (26.5%)	250 (39.0%)	221 (34.5%)
Ureteric and other urinary	183	16 (8.74%)	96 (52.5%)	71 (38.8%)	92	7 (7.61%)	38 (41.3%)	47 (51.1%)
Haematological								
Acute leukaemia	252	68 (27.0%)	81 (32.1%)	103 (40.9%)	219	59 (26.9%)	71 (32.4%)	89 (40.6%)
Chronic lymphocytic leukaemia	345	62 (18.0%)	168 (48.7%)	115 (33.3%)	184	21 (11.4%)	83 (45.1%)	80 (43.5%)
Hodgkin lymphoma	133	83 (62.4%)	37 (27.8%)	13 (9.77%)	110	66 (60.0%)	22 (20.0%)	22 (20.0%)
Multiple myeloma	537	108 (20.1%)	229 (42.6%)	200 (37.2%)	367	71 (19.3%)	138 (37.6%)	158 (43.1%)
Non-Hodgkin lymphoma	1134	294 (25.9%)	446 (39.3%)	394 (34.7%)	921	201 (21.8%)	359 (39.0%)	361 (39.2%)
Other haematological	360	99 (27.5%)	144 (40.0%)	117 (32.5%)	228	82 (36.0%)	82 (36.0%)	64 (28.1%)
Central nervous system (CNS)								
CNS	452	182 (40.3%)	182 (40.3%)	88 (19.5%)	325	138 (42.5%)	99 (30.5%)	88 (27.1%)
Sarcoma								
Bone sarcoma	42	24 (57.1%)	8 (19.0%)	10 (23.8%)	21	10 (47.6%)	5 (23.8%)	6 (28.6%)
Connective and soft tissue sarcoma	181	55 (30.4%)	64 (35.4%)	62 (34.3%)	191	66 (34.6%)	59 (30.9%)	66 (34.6%)
Skin								
Melanoma	1366	441 (32.3%)	475 (34.8%)	450 (32.9%)	1424	648 (45.5%)	454 (31.9%)	322 (22.6%)
Ocular								
Ocular	57	25 (43.9%)	18 (31.6%)	14 (24.6%)	54	23 (42.6%)	16 (29.6%)	15 (27.8%)
Breast								
Breast	65	21 (32.3%)	25 (38.5%)	19 (29.2%)	6101	2632 (43.1%)	1545 (25.3%)	1924 (31.5%)
Gynaecological								
Cervix	-	-	-	-	252	142 (56.3%)	65 (25.8%)	45 (17.9%)
Ovary	-	-	-	-	1174	419 (35.7%)	408 (34.8%)	347 (29.6%)
Uterus	-	-	-	-	1572	461 (29.3%)	718 (45.7%)	393 (25.0%)
Vulva/Vagina	-	-	-	-	241	67 (27.8%)	73 (30.3%)	101 (41.9%)
Prostate and other male organs								
Penile	119	34 (28.6%)	58 (48.7%)	27 (22.7%)	-	-	-	-
Prostate	8747	1183 (13.5%)	4750 (54.3%)	2814 (32.2%)	-	-	-	-
Testicular	403	371 (92.1%)	27 (6.70%)	5 (1.24%)	-	-	-	-
Other malignant neoplasms								
Other malignant neoplasms	101	41 (40.6%)	28 (27.7%)	32 (31.7%)	89	24 (27.0%)	23 (25.8%)	42 (47.2%)
Unknown primary								
Unknown primary	576	94 (16.3%)	210 (36.5%)	272 (47.2%)	629	70 (11.1%)	177 (28.1%)	382 (60.7%)

### Symptom signatures

**Fig. 2:**
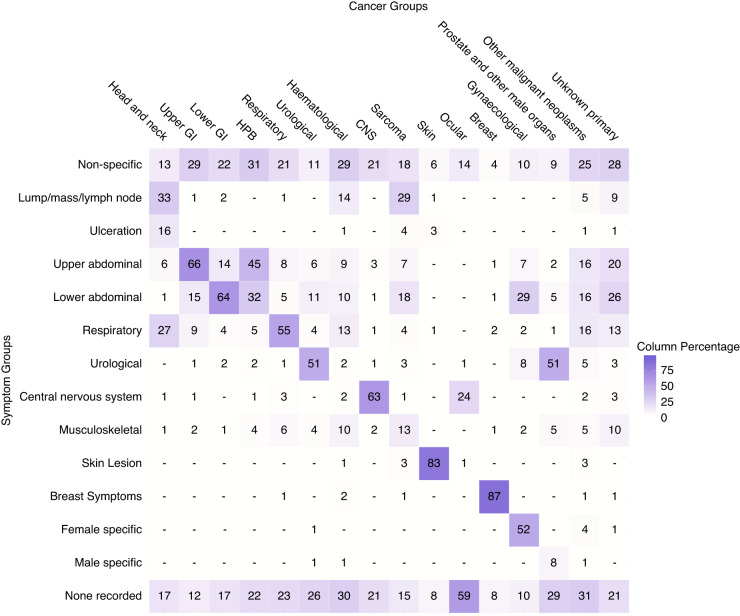
Proportion of symptom groups that patients presented with by cancer group (e.g. 66% of patients with upper GI cancer presented with upper abdominal symptoms). Columns add up to >100% because of multiple symptoms per patient. ‘-‘ represents values <= 0.5% of cases in a given cancer. GI Gastrointestinal, HPB Hepato-pancreato-biliary, CNS Central nervous system.

**Fig. 3:**
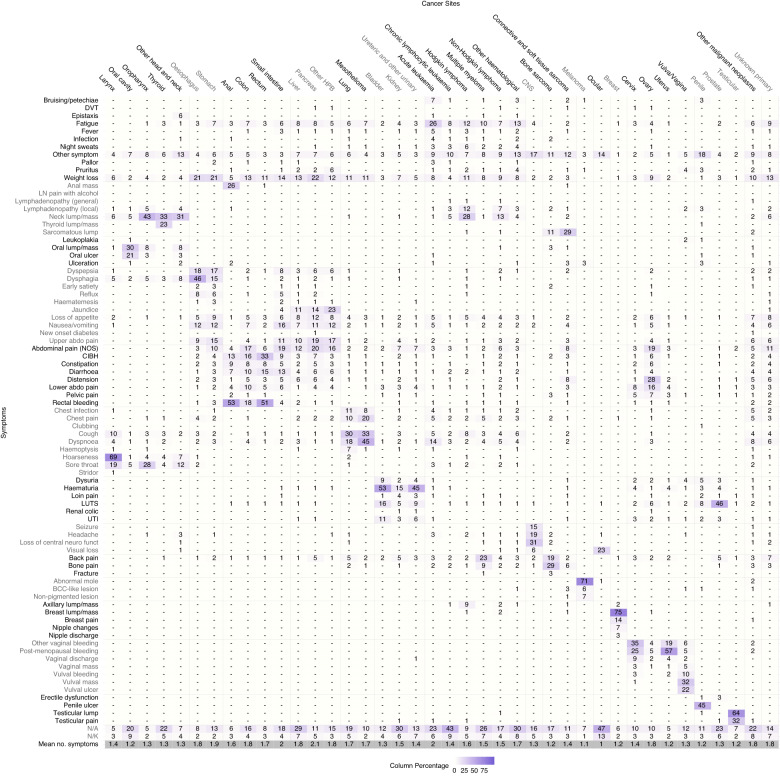
Proportion of symptoms that patients presented with by cancer site (e.g. 69% of patients with laryngeal cancer presented with hoarseness). Columns add to >100% because of multiple symptoms per patient. ‘-‘ represents values <= 0.5% of cases in a given cancer. Mean no. symptoms represents the average number of symptoms experienced per patient in each cancer site, excluding patients without recorded symptoms (N/A or N/K); it is calculated by the total number of symptoms recorded divided by the number of patients that had at least 1 symptom recorded. Cervix, ovary, uterus and vulva/vagina cancers only in women. Penile, prostate and testicular cancers only in men. HPB Hepato-pancreato-biliary, CNS Central nervous system, DVT Deep vein thrombosis, LN pain with alcohol Lymph node pain with alcohol, Abdominal pain (NOS) abdominal pain not otherwise specified, CIBH Change in bowel habit, LUTS Lower urinary tract symptoms, UTI urinary tract infection, N/A never presented to primary care pre-diagnosis*, N/K patient records don’t contain answer to the question.* *According to the NCDA data collection instrument definitions of these categories.

The relative frequencies of presenting symptoms (symptom signatures) of each cancer site are shown in Figs. 2, 3 with exact values in Supplementary Tables 3 and 4.

Considering the 16 cancer site groups, we observe a higher concentration of similar symptoms among cancers relating to organs of the same body system or region (Fig. 2). For example, patients with upper abdominal organ cancers tend to frequently present with upper abdominal symptoms, and the same is observed for gynaecological cancers and gynaecological symptoms. Patients with haematological cancers are an exception to this pattern, as they tend to present with more diverse symptoms.

Considering the 38 individual cancer sites, three principal patterns are apparent (Fig. 3). First, cancers with symptom signatures dominated by a frequent single presenting symptom. Examples include laryngeal cancer (69% of patients presenting with hoarseness), melanoma (71% with abnormal mole/lesion), and breast cancer (75% with breast lump/mass). Second, cancers with a broad range of presenting symptoms. For example, the most frequent presenting symptom among patients with pancreatic cancer (weight loss), renal cancer (haematuria) and multiple myeloma (back pain) was recorded in <25% of patients with each of these cancers. Third, cancers with relatively high percentages of patients without recorded presenting symptoms. For example, ocular cancer and chronic lymphocytic leukaemia have high proportions of patients without recorded symptoms (60% and 52%, respectively). Other notable cancer sites in this group include liver (36%), renal (36%), oral (29%), prostate (29%) and thyroid (27%) cancers. In contrast, laryngeal, oropharyngeal and uterine cancers had <9% of patients without recorded symptoms.

In total, there were 77,089 symptoms corresponding to the 55,122 analysed patients (1.4 symptoms/patient). The mean number of symptoms per patient ranged from 1 for ocular cancer and 1.1 for melanoma, to 2 for acute leukaemia and 2.1 for pancreatic cancer (Fig. 3, bottom row). A further illustration of the breadth of the symptom signature of each cancer site is provided in Table 2, describing the number of symptoms occurring in differently sized sub-cohorts pertinent to each cancer site. Higher count of symptoms occurring in >1% of cases indicate that a cancer site has a broader symptom signature, and vice versa. For example, a total variety of 30 symptoms was recorded in >1% of all patients with non-HL (Non-Hodgkin Lymphoma), compared to 7 symptoms recorded in >1% of women with breast cancer. The number of symptoms occurring in at least half of the cases is an indicator of whether the signature is dominated by specific presenting symptoms (e.g. hoarseness in 69% of patients with laryngeal cancer in comparison to no single symptom having a relative frequency exceeding 25% among pancreatic cancer patients, Fig. 3). Further descriptions of this nature can be found in Supplementary Table 5.

**Table 2:** Number of symptoms per cancer site that occurred in more than 1% and 50% of cases.

Cancer Site	*N*	% with at least one recorded symptom (95% CI)	Number of symptoms with frequency > 1% by cancer site cohort	Number of symptoms with frequency > 50% by cancer site cohort
Head and neck				
Larynx	368	91.8 (88.6, 94.2)	13	1 (hoarseness)
Oral cavity	585	70.6 (66.8, 74.1)	12	0
Oropharynx	653	92.2 (89.9, 94.0)	12	0
Thyroid	633	73.6 (70.0, 76.9)	11	0
Other head and neck	481	88.6 (85.4, 91.1)	15	0
Upper gastrointestinal				
Oesophagus	1391	89.8 (88.1, 91.3)	21	0
Stomach	1029	84.6 (82.3, 86.7)	23	0
Lower gastrointestinal				
Anal	265	91.3 (87.3, 94.1)	16	1 (rectal bleeding)
Colon	4209	80.0 (78.7, 81.2)	17	0
Rectum	1641	88.8 (87.2, 90.3)	15	1 (rectal bleeding)
Small intestine	256	77.7 (72.2, 82.4)	21	0
Hepato-pancreato-biliary (HPB)				
Liver	929	64.4 (61.2, 67.4)	21	0
Pancreas	1725	84.8 (83.0, 86.4)	23	0
Other HPB	425	81.9 (77.9, 85.3)	20	0
Respiratory				
Lung	7608	76.4 (75.4, 77.3)	17	0
Mesothelioma	464	87.5 (84.2, 90.2)	15	0
Urological				
Bladder	1518	84.7 (82.8, 86.4)	11	1 (haematuria)
Kidney	1695	63.9 (61.6, 66.1)	26	0
Ureteric and other urinary	275	80.0 (74.9, 84.3)	19	0
Haematological				
Acute leukaemia	471	72.0 (67.8, 75.8)	27	0
Chronic lymphocytic leukaemia	529	47.6 (43.4, 51.9)	16	0
Hodgkin lymphoma	243	86.0 (81.1, 89.8)	23	0
Multiple myeloma	904	66.6 (63.5, 69.6)	19	0
Non-Hodgkin lymphoma	2055	78.1 (76.3, 79.8)	30	0
Other haematological	588	61.2 (57.2, 65.1)	26	0
Central nervous system (CNS)				
CNS	777	78.6 (75.6, 81.4)	9	0
Sarcoma				
Bone sarcoma	63	79.4 (67.8, 87.5)	15	0
Connective and soft tissue sarcoma	372	86.0 (82.1, 89.2)	26	0
Skin				
Melanoma	2790	91.8 (90.7, 92.8)	6	1 (abnormal mole)
Ocular				
Ocular	111	40.5 (31.9, 49.8)	2	0
Breast				
Breast	6166	91.8 (91.1, 92.5)	7	1 (breast lump/mass)
Gynaecological				
Cervix	252	86.9 (82.2, 90.5)	20	0
Ovary	1174	86.7 (84.7, 88.5)	27	0
Uterus	1572	93.2 (91.8, 94.3)	17	1 (post-menopausal bleeding)
Vulva/Vagina	241	84.6 (79.6, 88.7)	14	0
Prostate and other male organs				
Penile	119	86.6 (79.3, 91.6)	11	0
Prostate	8747	70.1 (69.2, 71.1)	13	0
Testicular	403	90.8 (87.6, 93.3)	6	1 (testicular lump)
Other malignant neoplasms				
Other malignant neoplasms	190	69.5 (62.6, 75.6)	35	0
Unknown primary				
Unknown primary	1205	79.2 (76.8, 81.4)	28	0

### Cancer site case-mix

**Fig. 4:**
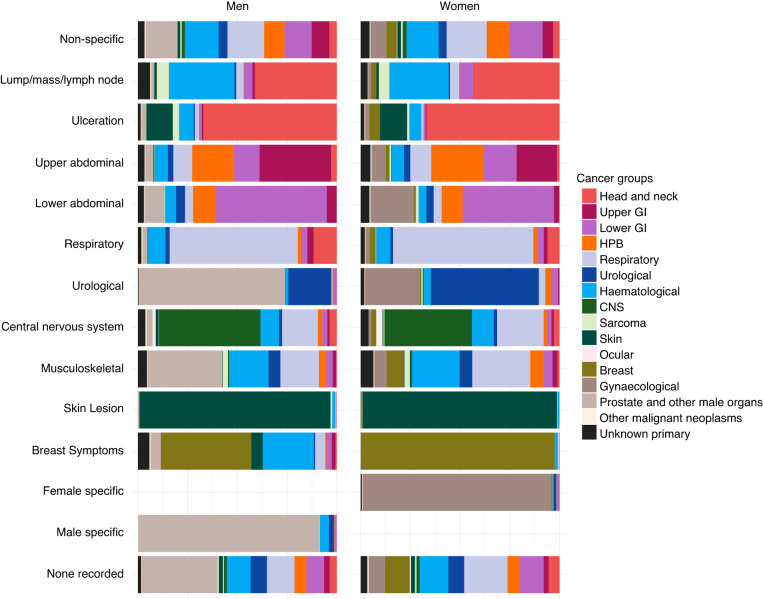
The spectrum of cancer groups contained within each symptom group (proportion of cancer groups within each symptom group). “Gynaecological” and “Prostate and other male organs” cancer groups have similar colours because they occur exclusively in women and men, respectively. GI Gastrointestinal, HPB Hepato-pancreato-biliary, CNS Central nervous system, MSK Musculoskeletal.

**Fig. 5:**
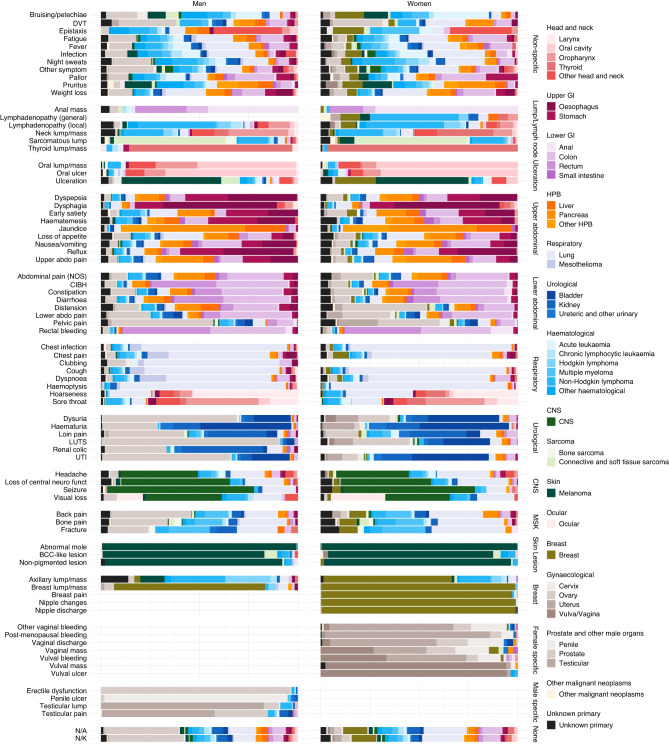
The spectrum of cancer sites contained within each symptom (proportion of cancer sites within each symptom). “Gynaecological” and “Prostate and other male organs” cancer groups have similar colours because they occur exclusively in women and men, respectively. Symptoms with *n* < 20 by sex were excluded from the visualisation: Lymph node pain with alcohol (both), general lymphadenopathy (men), leukoplakia (both), new onset diabetes (both), clubbing (women), stridor (both), renal colic (women), breast pain (men), nipple changes (men), nipple discharge (men). GI Gastrointestinal, HPB Hepato-pancreato-biliary, CNS Central nervous system, DVT Deep Vein Thrombosis, Abdominal pain (NOS) Abdominal pain not otherwise specified, CIBH Change In Bowel Habit, LUTS Lower urinary tract symptoms, UTI Urinary Tract Infection, N/A never presented to primary care pre-diagnosis*, N/K patient records don’t contain answer to the question.* *According to the NCDA data collection instrument definitions of these categories.

The cancer site signatures of different symptoms are illustrated as spectra (Figs. 4, 5), with exact values in Supplementary Tables 6, 7.

Considering the 13 symptom groups, two principal patterns are apparent. Certain presenting symptoms tend to relate to cancers of the same body system or region (Fig. 4). For example, skin lesions almost solely relate to skin cancer cases, respiratory symptoms to respiratory organ cancers, and urological symptoms to urological or sex-specific cancers. In contrast, the group of non-specific symptoms typically relates to a wide range of cancer sites (see also below). Abdominal symptoms (both upper or lower), although often relating to abdominal cancers, also relate to other cancer sites.

Considering the 83 symptom categories, we observed that some tend to principally relate to specific cancer sites (Fig. 5). Examples include anal mass (anal cancer, diagnosed in 46% of men and 72% of women presenting with this symptom), haemoptysis (lung cancer, in 84% of men and 90% of women with this symptom) and abnormal mole (melanoma, in 99% of either men or women presenting with this symptom). In contrast, patients with other symptoms (such as abdominal pain (not otherwise specified), weight loss, fatigue, and night sweats) were subsequently diagnosed with a wider range of cancer sites. For example, among all female patients presenting with abdominal pain (not otherwise specified), 26% were diagnosed with colon, 12% with pancreatic, 16% with ovarian and 44% with another 27 cancer sites, with corresponding figures in males being 28%, 14%, 8% for prostate cancer, and 50% with another 22 cancer sites. Similarly, among female patients presenting with weight loss, 22% were diagnosed with lung, 16% with colon, 10% with pancreatic cancer; among male patients presenting with weight loss, 20% were diagnosed with lung, 11% with colon, 12% with prostate, 9% with oesophageal and 9% with pancreatic cancer.

Certain symptoms have a different cancer site case mix by sex (Figs. 4, 5). For example, although musculoskeletal symptoms often present in sarcoma or haematological cancers in both men and women, in men they also frequently relate to prostate cancer. Similarly, the cancer site case mix of patients without recorded symptoms varied by sex, chiefly reflecting a high percentage (38%) of men without recorded presenting symptoms diagnosed with prostate cancer.

## Discussion

### Summary

Among incident cancer cases, we have mapped the presenting symptom signatures of 38 cancer sites and described the cancer site case-mix of 83 presenting symptoms. Certain presenting symptoms are typically concentrated in cancers of the same body system or region, and vice versa. When the symptom signature of a given cancer is dominated by a single symptom, the cancer case-mix of that symptom is also dominated by the same cancer; conversely, relationships between presenting symptom and cancer site are much weaker for cancers with broader symptom signature. The cancer site case-mix of certain symptoms (e.g. musculoskeletal symptoms) varies by sex.

### Comparisons with literature

Evidence on the presenting symptom signature of 15 cancer sites has been reviewed previously [10]. Generally, relevant prior evidence is concentrated on single cancer sites; in contrast we have examined the symptom signature of 38 cancers simultaneously. Acknowledging this difference, our findings concord with prior evidence, although they expand to an additional 23 cancers sites with little or prior population-based evidence on their presenting symptoms (such as laryngeal, liver, melanoma, mesothelioma, oral, penile, sarcoma, small intestinal, testicular, thyroid, vaginal and vulval) [10].

Consistent with previous literature, we have found breast and bladder cancers to have narrow symptom signatures [10], and haematological [11], pancreatic [12], and renal cancers [10] to have broad symptom signatures. Haemoptysis has a relatively high predictive value for lung cancer, but it only occurs in 20% of patients with lung cancer [13]; consistent with this prior evidence, we found that a fifth of lung cancer patients in our study population presented with haemoptysis. As described previously, we have found that chronic lymphocytic leukaemia patients typically had no recorded symptoms, which concords with prior evidence indicating that this cancer is often detected asymptomatically [11]. Prostate, renal, liver and thyroid cancers also had high percentages of diagnosis without recorded symptoms, consistent with prior knowledge about detection via opportunistic screening or incidental identification in many patients [14]. The observation that ocular and oral cancers also have a high proportion of non-recorded symptoms is novel, and could indicate detection of those cancers outside primary care (e.g. opticians and dentists). In keeping with prior evidence, most cancers arising after upper or lower abdominal symptom presentations related to cancers of the abdomen (e.g. upper and lower GI, hepato-pancreato-biliary, urological, prostate and other male organs/gynaecological) though around one in five related to non-abdominal sites [15, 16]. Concurring with prior evidence, we have observed a diverse symptom signature for colon and rectal cancers [17].

In brief, the findings concord with prior evidence but amplify it substantially in respect of number of presenting symptoms examined and the range of cancer sites considered.

### Strengths and limitations

We covered a wide range of symptoms and cancer sites in a population-based incident cohort of patients with cancer. However, there are several limitations to consider. This was a case-only analysis (only patients with diagnosis of cancer were included). While this is an inherent feature of all epidemiological studies using cancer registry data, it is important that this is borne in mind for interpretation. For example, no inferences can be made about the predictive value of certain symptoms for specific cancers.

By the design of the NCDA questionnaire, GPs were asked to record the first presenting symptom(s) that prompted the suspicion of cancer. Since the surveys were filled by the GPs retrospectively, it is possible that certain symptoms more closely related to the patient’s diagnosis were recorded in the audit. It is also possible that other symptoms, particularly non-specific ones, were present and did relate to the underlying cancer but not deemed to do so by the GP, and therefore were not recorded. However, GPs had access to both coded and free-text data in the patients’ records—a unique feature of NCDA study, which mitigates concerns about reliance on structured (coded) fields in primary care electronic health record data sources [18]. The symptoms recorded relate to the symptoms presented to the GP which may differ from the symptoms experienced at symptom onset.

Although we present associations between individual symptoms and individual cancer sites, we have also grouped symptoms and cancer sites to provide higher-level summaries. The definition of these groups is normative and chiefly guided by anatomical considerations; by its nature includes a degree of heterogeneity.

### Implications

Three main translational implications arise from the findings.

Considering research implications, the results provide foundational evidence that can be used to validate the completeness of phenotyping of cancer symptoms in electronic health record sources, or profile associations between presenting symptoms and diagnostic process measures, such as investigation use.

Considering implications for public health or clinical practice, the findings can guide decision-making about the choice of target symptoms in symptom awareness campaigns, and inform their evaluation, regarding the range of cancer sites where changes in diagnostic pathways and intervals may be observable. Similarly, they can guide investigation strategies in patients presenting with specific symptoms, for example, prioritising certain tests over others (e.g. endoscopy over imaging), given differences in the expected probability of specific cancer sites. Further, novel/emerging diagnostic technologies, such as multi-cancer early detection tests, could be preferentially deployed on symptoms potentially associated with a wider range of cancer sites. As an example, Multi-Cancer Early Detection (MCED) tests provide information on whether a cancer signal was detected (yes/no) and up to two predicted ‘cancer site origins’, i.e. suspected site of underlying cancer [19]. Clinicians may be able to complement such information with evidence on the associations between presenting symptoms and the likely distribution of cancer sites in cases presenting with that symptom, to further inform investigation strategies and test sequencing. Additionally, our study could motivate future studies into examining the probability of specific cancer sites in cancer patients conditional on their presenting symptoms, which could further improve the diagnostic accuracy and usefulness of information that can be derived by MCED tests.

Considering implications for public policy, the findings emphasise the importance of considering the overall risk of cancer (across body organs and systems) in symptom-based referral guidelines for suspected cancer. Instead, current UK guideline recommendations (issued in 2015) chiefly relate to symptoms of specific cancer sites [6], meaning that, as we show, the broader cancer site case-mix of different symptoms (particularly vague symptoms) is not appreciated. There is no equivalent single body of guidelines for presenting symptoms in the US setting, although evidence indicates that diagnostic delays in symptomatic patients subsequently diagnosed with cancer are comparable to those seen in Europe, and that the predictive values of different symptoms among presenters are also similar [20, 21]. Recent evidence demonstrates that the predictive values of three common vague symptoms, i.e. weight loss, fatigue and abdominal pain, do not exceed the 3% normative referral threshold used by NICE when individual cancer sites are considered on their own, but do so when all cancer sites are considered together [22–24].

## Conclusion

The study provides a detailed understanding of bidirectional relationships between presenting symptoms and cancer sites among incident cases, enabling research examining associations between symptomatic presentations and diagnostic process measures. Future clinical practice recommendations for specialist referral ought to encompass a broader range of cancer sites per symptom. The design of symptom awareness campaigns can be appropriately guided regarding choice of target symptoms, and diagnostic strategies can be suitably informed.

### Supplementary information

Supplementary Material

STROBE checklist

## Data Availability

Data used in this study (National Cancer Diagnosis Audit data used in this study are available through application to NHS England Data Access Request Service (DARS).

## Code availability

All analysis was conducted using R (version 4.1.2) and can be accessed online at https://github.com/nadine-zakkak/presenting-symptom-signatures-of-incident-cancer.
