# Supplementary material for: The presenting symptom signatures of incident cancer: evidence from the English 2018 National Cancer Diagnosis Audit
Source: Br J Cancer. 2023 Dec 6;130(2):297–307. doi: 10.1038/s41416-023-02507-4 (PMC10803766; doi:10.1038/s41416-023-02507-4)
Supplement: Supplementary file 1 — Supplementary Material [file 41416_2023_2507_MOESM1_ESM.docx]

# Table of Contents

[Supplementary Table 1. ICD-10 cancer site and cancer group classifications. 2](#_Toc148442576)

[Supplementary Table 2. Symptoms classification into symptom groups. 5](#_Toc148442577)

[Supplementary Table 3. Proportion of symptoms groups occurring in each cancer group. 7](#_Toc148442578)

[Supplementary Table 4. Proportion of symptoms occurring in each cancer site. 13](#_Toc148442579)

[Supplementary Table 5. Number of symptoms per cancer site that occurred in more than 1%, 5%, 10%, 20% and 50% of cases. 89](#_Toc148442580)

[Supplementary Table 6. Proportion of cancer groups occurring in each symptom group. 91](#_Toc148442581)

[Supplementary Table 7. Proportion of cancer sites occurring in each symptom. 97](#_Toc148442582)

## Supplementary table 1

Supplementary Table 1. ICD-10 cancer site and cancer group classifications.

| ICD10 O2 | Cancer Group | Cancer Site |
| --- | --- | --- |
| C00 | Head and neck | Other head and neck |
| C01 | Head and neck | Oropharynx |
| C02 | Head and neck | Oral cavity |
| C03 | Head and neck | Oral cavity |
| C04 | Head and neck | Oral cavity |
| C05 | Head and neck | Oral cavity |
| C06 | Head and neck | Oral cavity |
| C07 | Head and neck | Other head and neck |
| C08 | Head and neck | Other head and neck |
| C09 | Head and neck | Oropharynx |
| C10 | Head and neck | Oropharynx |
| C11 | Head and neck | Other head and neck |
| C12 | Head and neck | Other head and neck |
| C13 | Head and neck | Other head and neck |
| C14 | Head and neck | Other head and neck |
| C15 | Upper gastrointestinal | Oesophagus |
| C16 | Upper gastrointestinal | Stomach |
| C17 | Lower gastrointestinal | Small intestine |
| C18 | Lower gastrointestinal | Colon |
| C19 | Lower gastrointestinal | Colon |
| C20 | Lower gastrointestinal | Rectum |
| C21 | Lower gastrointestinal | Anal |
| C22 | Hepato-pancreato-biliary (HPB) | Liver |
| C23 | Hepato-pancreato-biliary | Other HPB |
| C24 | Hepato-pancreato-biliary | Other HPB |
| C25 | Hepato-pancreato-biliary | Pancreas |
| C26 | Other malignant neoplasms | Other malignant neoplasms |
| C30 | Head and neck | Other head and neck |
| C31 | Head and neck | Other head and neck |
| C32 | Head and neck | Larynx |
| C33 | Respiratory | Lung |
| C34 | Respiratory | Lung |
| C37 | Haematological | Other haematological |
| C38 | Other malignant neoplasms | Other malignant neoplasms |
| C40 | Sarcoma | Bone sarcoma |
| C41 | Sarcoma | Bone sarcoma |
| C43 | Skin | Melanoma |
| C45 | Respiratory | Mesothelioma |
| C46 | Other malignant neoplasms | Other malignant neoplasms |
| C47 | Other malignant neoplasms | Other malignant neoplasms |
| C48 | Sarcoma | Connective and soft tissue sarcoma |
| C49 | Sarcoma | Connective and soft tissue sarcoma |
| C50 | Breast | Breast |
| C51 | Gynaecological | Vulva/Vagina |
| C52 | Gynaecological | Vulva/Vagina |
| C53 | Gynaecological | Cervix |
| C54 | Gynaecological | Uterus |
| C55 | Gynaecological | Uterus |
| C56 | Gynaecological | Ovary |
| C57 | Gynaecological | Ovary |
| C58 | Other malignant neoplasms | Other malignant neoplasms |
| C60 | Prostate and other male organs | Penile |
| C61 | Prostate and other male organs | Prostate |
| C62 | Prostate and other male organs | Testicular |
| C63 | Prostate and other male organs | Testicular |
| C64 | Urological | Kidney |
| C65 | Urological | Ureteric and other urinary |
| C66 | Urological | Ureteric and other urinary |
| C67 | Urological | Bladder |
| C68 | Urological | Ureteric and other urinary |
| C69 | Ocular | Ocular |
| C70 | Central nervous system (CNS) | CNS |
| C71 | Central nervous system | CNS |
| C72 | Central nervous system | CNS |
| C73 | Head and neck | Thyroid |
| C74 | Other malignant neoplasms | Other malignant neoplasms |
| C75 | Other malignant neoplasms | Other malignant neoplasms |
| C76 | Other malignant neoplasms | Other malignant neoplasms |
| C77 | Unknown primary | Unknown primary |
| C78 | Unknown primary | Unknown primary |
| C79 | Unknown primary | Unknown primary |
| C80 | Unknown primary | Unknown primary |
| C81 | Haematological | Hodgkin lymphoma |
| C82 | Haematological | Non-Hodgkin lymphoma |
| C83 | Haematological | Non-Hodgkin lymphoma |
| C84 | Haematological | Other haematological |
| C85 | Haematological | Non-Hodgkin lymphoma |
| C88 | Haematological | Other haematological |
| C90 | Haematological | Multiple myeloma |
| C910 | Haematological | Acute leukaemia |
| C911 | Haematological | Chronic lymphocytic leukaemia |
| C913 | Haematological | Other haematological |
| C914 | Haematological | Other haematological |
| C915 | Haematological | Other haematological |
| C917 | Haematological | Other haematological |
| C919 | Haematological | Other haematological |
| C920 | Haematological | Acute leukaemia |
| C921 | Haematological | Other haematological |
| C923 | Haematological | Other haematological |
| C924 | Haematological | Acute leukaemia |
| C925 | Haematological | Acute leukaemia |
| C927 | Haematological | Other haematological |
| C929 | Haematological | Other haematological |
| C93 | Haematological | Acute leukaemia |
| C94 | Haematological | Acute leukaemia |
| C950 | Haematological | Acute leukaemia |
| C959 | Haematological | Other haematological |
| C96 | Haematological | Other haematological |

## Supplementary table 2

Supplementary Table 2. Symptoms classification into symptom groups.

| Symptom Group | Symptom |
| --- | --- |
| Upper abdominal | Loss of appetite |
| Upper abdominal | Nausea and/or vomiting |
| Upper abdominal | Upper abdominal pain |
| Upper abdominal | Dysphagia |
| Upper abdominal | Dyspepsia |
| Upper abdominal | Jaundice |
| Upper abdominal | Gastroesophageal reflux |
| Upper abdominal | Early satiety |
| Upper abdominal | Haematemesis |
| Upper abdominal | New onset diabetes |
| Lower abdominal | Abdominal pain (Not otherwise specified) |
| Lower abdominal | Rectal bleeding |
| Lower abdominal | Change in bowel habit |
| Lower abdominal | Diarrhoea |
| Lower abdominal | Lower abdominal pain |
| Lower abdominal | Distension |
| Lower abdominal | Constipation |
| Lower abdominal | Pelvic pain |
| Breast Symptoms | Axillary lump/mass |
| Breast Symptoms | Breast lump/mass |
| Breast Symptoms | Breast pain |
| Breast Symptoms | Nipple changes |
| Breast Symptoms | Nipple discharge |
| Central nervous system | Prog/sub-acute loss of central neuro function |
| Central nervous system | Headache |
| Central nervous system | Fit/seizure |
| Central nervous system | Visual disturbance or loss |
| Lump/mass/lymph node | Neck lump/mass |
| Lump/mass/lymph node | Unexplained lump suspicious of sarcoma |
| Lump/mass/lymph node | Lymphadenopathy (generalised) |
| Lump/mass/lymph node | Lymph node pain with alcohol |
| Lump/mass/lymph node | Thyroid lump/mass |
| Lump/mass/lymph node | Anal mass |
| Lump/mass/lymph node | Lymphadenopathy (localised) |
| Musculoskeletal | Back pain |
| Musculoskeletal | Bone pain |
| Musculoskeletal | Fracture |
| Respiratory | Cough |
| Respiratory | Dyspnoea |
| Respiratory | Chest pain |
| Respiratory | Chest infection |
| Respiratory | Haemoptysis |
| Respiratory | Sore throat |
| Respiratory | Hoarseness |
| Respiratory | Clubbing |
| Respiratory | Stridor |
| Skin Lesion | Non-pigmented lesion |
| Skin Lesion | Lesions suspicious of BCC |
| Skin Lesion | Abnormal mole |
| Ulceration | Lip/oral cavity/tongue lump/mass |
| Ulceration | Ulceration |
| Ulceration | Lip/oral cavity/tongue ulcer |
| Ulceration | Leukoplakia |
| Urological | Loin pain |
| Urological | LUTS (nocturia, frequency, hesitancy, urgency, retention) |
| Urological | Haematuria |
| Urological | Dysuria |
| Urological | Urinary tract infection |
| Urological | Renal colic |
| Female specific | Post-menopausal bleeding |
| Female specific | Other vaginal bleeding |
| Female specific | Vaginal discharge |
| Female specific | Vulval mass |
| Female specific | Vulval bleeding |
| Female specific | Vulval ulceration |
| Female specific | Vaginal mass |
| Male specific | Testicular lump |
| Male specific | Erectile dysfunction |
| Male specific | Testicular pain |
| Male specific | Penile ulceration |
| Non-specific | Weight loss |
| Non-specific | Other symptom |
| Non-specific | Fatigue |
| Non-specific | Night sweats |
| Non-specific | Fever |
| Non-specific | Infection |
| Non-specific | Pallor |
| Non-specific | Bruising, bleeding or petechiae |
| Non-specific | Deep vein thrombosis |
| Non-specific | Epistaxis |
| Non-specific | Pruritus |
| None recorded | Not applicable (N/A) |
| None recorded | Not known (N/K) |

## Supplementary table 3

Supplementary Table 3. Proportion of symptoms groups occurring in each cancer group. Proportions add up to >100% because of multiple symptoms per patient.

| Symptom Group | Proportion of Patients by Cancer Group % (95% CI) |
| --- | --- |
| Head and neck (N = 2720) | |
| Non-specific | 13.1 (11.9, 14.4) |
| Lump/mass/lymph node | 32.6 (30.8, 34.4) |
| Ulceration | 16.4 (15.0, 17.8) |
| Upper abdominal | 5.74 (4.92, 6.67) |
| Lower abdominal | 0.51 (0.31, 0.86) |
| Respiratory | 26.9 (25.2, 28.6) |
| Urological | 0.07 (0.02, 0.27) |
| Central nervous system | 1.25 (0.90, 1.74) |
| Musculoskeletal | 0.51 (0.31, 0.86) |
| Skin Lesion | 0.15 (0.06, 0.38) |
| Breast Symptoms | 0.22 (0.10, 0.48) |
| Female specific | 0.07 (0.02, 0.27) |
| Male specific | 0.00 (0.00, 0.14) |
| None recorded | 17.5 (16.1, 18.9) |
| Upper gastrointestinal (N = 2420) | |
| Non-specific | 28.8 (27.1, 30.7) |
| Lump/mass/lymph node | 0.62 (0.38, 1.02) |
| Ulceration | 0.17 (0.06, 0.42) |
| Upper abdominal | 66.3 (64.4, 68.2) |
| Lower abdominal | 15.1 (13.7, 16.6) |
| Respiratory | 8.80 (7.74, 10.00) |
| Urological | 0.70 (0.44, 1.12) |
| Central nervous system | 0.54 (0.31, 0.92) |
| Musculoskeletal | 1.86 (1.39, 2.48) |
| Skin Lesion | 0.00 (0.00, 0.16) |
| Breast Symptoms | 0.17 (0.06, 0.42) |
| Female specific | 0.12 (0.04, 0.36) |
| Male specific | 0.00 (0.00, 0.16) |
| None recorded | 12.4 (11.1, 13.8) |
| Lower gastrointestinal (N = 6371) | |
| Non-specific | 21.7 (20.7, 22.8) |
| Lump/mass/lymph node | 1.82 (1.52, 2.18) |
| Ulceration | 0.13 (0.06, 0.25) |
| Upper abdominal | 14.2 (13.4, 15.1) |
| Lower abdominal | 63.6 (62.4, 64.8) |
| Respiratory | 4.14 (3.68, 4.66) |
| Urological | 1.71 (1.42, 2.06) |
| Central nervous system | 0.42 (0.29, 0.62) |
| Musculoskeletal | 1.37 (1.11, 1.68) |
| Skin Lesion | 0.00 (0.00, 0.06) |
| Breast Symptoms | 0.14 (0.07, 0.27) |
| Female specific | 0.22 (0.13, 0.37) |
| Male specific | 0.14 (0.07, 0.27) |
| None recorded | 17.4 (16.4, 18.3) |
| Hepato-pancreato-biliary (N = 3079) | |
| Non-specific | 31.5 (29.9, 33.1) |
| Lump/mass/lymph node | 0.26 (0.13, 0.51) |
| Ulceration | 0.03 (0.01, 0.18) |
| Upper abdominal | 44.8 (43.0, 46.5) |
| Lower abdominal | 32.0 (30.3, 33.6) |
| Respiratory | 5.20 (4.47, 6.04) |
| Urological | 2.44 (1.95, 3.04) |
| Central nervous system | 0.75 (0.50, 1.12) |
| Musculoskeletal | 3.67 (3.06, 4.39) |
| Skin Lesion | 0.00 (0.00, 0.12) |
| Breast Symptoms | 0.10 (0.03, 0.29) |
| Female specific | 0.16 (0.07, 0.38) |
| Male specific | 0.06 (0.02, 0.24) |
| None recorded | 21.8 (20.3, 23.3) |
| Respiratory (N = 8072) | |
| Non-specific | 21.2 (20.3, 22.1) |
| Lump/mass/lymph node | 1.09 (0.89, 1.34) |
| Ulceration | 0.12 (0.07, 0.23) |
| Upper abdominal | 7.95 (7.38, 8.56) |
| Lower abdominal | 4.79 (4.35, 5.28) |
| Respiratory | 55.2 (54.1, 56.3) |
| Urological | 0.87 (0.69, 1.09) |
| Central nervous system | 2.73 (2.39, 3.10) |
| Musculoskeletal | 6.43 (5.92, 6.99) |
| Skin Lesion | 0.06 (0.03, 0.14) |
| Breast Symptoms | 0.52 (0.39, 0.70) |
| Female specific | 0.05 (0.02, 0.13) |
| Male specific | 0.01 (0.00, 0.07) |
| None recorded | 23.0 (22.1, 23.9) |
| Urological (N = 3488) | |
| Non-specific | 11.2 (10.2, 12.2) |
| Lump/mass/lymph node | 0.46 (0.28, 0.74) |
| Ulceration | 0.06 (0.02, 0.21) |
| Upper abdominal | 5.50 (4.80, 6.31) |
| Lower abdominal | 11.4 (10.4, 12.5) |
| Respiratory | 4.13 (3.52, 4.84) |
| Urological | 50.6 (48.9, 52.3) |
| Central nervous system | 0.49 (0.30, 0.78) |
| Musculoskeletal | 4.04 (3.44, 4.75) |
| Skin Lesion | 0.03 (0.01, 0.16) |
| Breast Symptoms | 0.11 (0.04, 0.29) |
| Female specific | 0.77 (0.53, 1.12) |
| Male specific | 0.63 (0.42, 0.95) |
| None recorded | 25.8 (24.4, 27.3) |
| Haematological (N = 4790) | |
| Non-specific | 29.0 (27.8, 30.3) |
| Lump/mass/lymph node | 13.8 (12.8, 14.8) |
| Ulceration | 0.98 (0.74, 1.30) |
| Upper abdominal | 8.62 (7.86, 9.45) |
| Lower abdominal | 9.52 (8.72, 10.4) |
| Respiratory | 12.8 (11.9, 13.8) |
| Urological | 2.46 (2.06, 2.94) |
| Central nervous system | 2.32 (1.93, 2.78) |
| Musculoskeletal | 9.83 (9.02, 10.7) |
| Skin Lesion | 0.56 (0.39, 0.82) |
| Breast Symptoms | 2.46 (2.06, 2.94) |
| Female specific | 0.19 (0.10, 0.36) |
| Male specific | 0.71 (0.51, 0.99) |
| None recorded | 29.7 (28.4, 31.0) |
| Central nervous system (N = 777) | |
| Non-specific | 21.2 (18.5, 24.2) |
| Lump/mass/lymph node | 0.13 (0.02, 0.73) |
| Ulceration | 0.00 (0.00, 0.49) |
| Upper abdominal | 2.96 (1.98, 4.40) |
| Lower abdominal | 0.51 (0.20, 1.32) |
| Respiratory | 0.90 (0.44, 1.85) |
| Urological | 0.90 (0.44, 1.85) |
| Central nervous system | 62.5 (59.1, 65.9) |
| Musculoskeletal | 1.80 (1.08, 3.00) |
| Skin Lesion | 0.00 (0.00, 0.49) |
| Breast Symptoms | 0.26 (0.07, 0.93) |
| Female specific | 0.00 (0.00, 0.49) |
| Male specific | 0.00 (0.00, 0.49) |
| None recorded | 21.4 (18.6, 24.4) |
| Sarcoma (N = 435) | |
| Non-specific | 18.2 (14.8, 22.1) |
| Lump/mass/lymph node | 28.5 (24.5, 32.9) |
| Ulceration | 3.68 (2.28, 5.89) |
| Upper abdominal | 7.36 (5.26, 10.2) |
| Lower abdominal | 17.7 (14.4, 21.6) |
| Respiratory | 3.91 (2.45, 6.17) |
| Urological | 2.53 (1.42, 4.47) |
| Central nervous system | 0.92 (0.36, 2.34) |
| Musculoskeletal | 13.1 (10.3, 16.6) |
| Skin Lesion | 3.22 (1.93, 5.33) |
| Breast Symptoms | 1.38 (0.63, 2.98) |
| Female specific | 0.23 (0.04, 1.29) |
| Male specific | 0.23 (0.04, 1.29) |
| None recorded | 14.9 (11.9, 18.6) |
| Skin (N = 2790) | |
| Non-specific | 5.73 (4.93, 6.66) |
| Lump/mass/lymph node | 0.93 (0.64, 1.36) |
| Ulceration | 3.26 (2.66, 3.99) |
| Upper abdominal | 0.47 (0.27, 0.80) |
| Lower abdominal | 0.18 (0.08, 0.42) |
| Respiratory | 0.65 (0.41, 1.02) |
| Urological | 0.07 (0.02, 0.26) |
| Central nervous system | 0.36 (0.19, 0.66) |
| Musculoskeletal | 0.22 (0.10, 0.47) |
| Skin Lesion | 83.1 (81.7, 84.5) |
| Breast Symptoms | 0.39 (0.22, 0.70) |
| Female specific | 0.00 (0.00, 0.14) |
| Male specific | 0.00 (0.00, 0.14) |
| None recorded | 8.21 (7.25, 9.28) |
| Ocular (N = 111) | |
| Non-specific | 14.4 (9.07, 22.1) |
| Lump/mass/lymph node | 0.00 (0.00, 3.35) |
| Ulceration | 0.00 (0.00, 3.35) |
| Upper abdominal | 0.00 (0.00, 3.35) |
| Lower abdominal | 0.00 (0.00, 3.35) |
| Respiratory | 0.00 (0.00, 3.35) |
| Urological | 0.90 (0.16, 4.93) |
| Central nervous system | 24.3 (17.3, 33.1) |
| Musculoskeletal | 0.00 (0.00, 3.35) |
| Skin Lesion | 0.90 (0.16, 4.93) |
| Breast Symptoms | 0.00 (0.00, 3.35) |
| Female specific | 0.00 (0.00, 3.35) |
| Male specific | 0.00 (0.00, 3.35) |
| None recorded | 59.5 (50.2, 68.1) |
| Breast (N = 6166) | |
| Non-specific | 3.52 (3.09, 4.01) |
| Lump/mass/lymph node | 0.42 (0.29, 0.62) |
| Ulceration | 0.23 (0.14, 0.38) |
| Upper abdominal | 0.83 (0.63, 1.09) |
| Lower abdominal | 0.63 (0.46, 0.86) |
| Respiratory | 1.52 (1.25, 1.86) |
| Urological | 0.10 (0.04, 0.21) |
| Central nervous system | 0.21 (0.12, 0.36) |
| Musculoskeletal | 1.22 (0.97, 1.52) |
| Skin Lesion | 0.10 (0.04, 0.21) |
| Breast Symptoms | 86.9 (86.0, 87.7) |
| Female specific | 0.15 (0.08, 0.28) |
| Male specific | 0.00 (0.00, 0.06) |
| None recorded | 8.19 (7.53, 8.90) |
| Gynaecological (N = 3239) | |
| Non-specific | 10.3 (9.34, 11.4) |
| Lump/mass/lymph node | 0.46 (0.28, 0.76) |
| Ulceration | 0.19 (0.08, 0.40) |
| Upper abdominal | 6.64 (5.83, 7.55) |
| Lower abdominal | 29.1 (27.5, 30.7) |
| Respiratory | 2.47 (1.99, 3.06) |
| Urological | 8.24 (7.34, 9.24) |
| Central nervous system | 0.22 (0.10, 0.45) |
| Musculoskeletal | 1.85 (1.44, 2.38) |
| Skin Lesion | 0.09 (0.03, 0.27) |
| Breast Symptoms | 0.34 (0.19, 0.61) |
| Female specific | 52.5 (50.7, 54.2) |
| Male specific | 0.00 (0.00, 0.12) |
| None recorded | 10.3 (9.28, 11.4) |
| Prostate and other male organs (N = 9269) | |
| Non-specific | 9.44 (8.86, 10.1) |
| Lump/mass/lymph node | 0.20 (0.13, 0.32) |
| Ulceration | 0.12 (0.07, 0.21) |
| Upper abdominal | 1.55 (1.32, 1.83) |
| Lower abdominal | 5.22 (4.79, 5.69) |
| Respiratory | 1.18 (0.98, 1.42) |
| Urological | 50.7 (49.7, 51.7) |
| Central nervous system | 0.18 (0.11, 0.29) |
| Musculoskeletal | 5.45 (5.00, 5.93) |
| Skin Lesion | 0.02 (0.01, 0.08) |
| Breast Symptoms | 0.09 (0.04, 0.17) |
| Female specific | 0.00 (0.00, 0.04) |
| Male specific | 7.81 (7.28, 8.37) |
| None recorded | 28.8 (27.8, 29.7) |
| Other malignant neoplasms (N = 190) | |
| Non-specific | 25.3 (19.6, 31.9) |
| Lump/mass/lymph node | 4.74 (2.51, 8.76) |
| Ulceration | 1.05 (0.29, 3.76) |
| Upper abdominal | 16.3 (11.7, 22.2) |
| Lower abdominal | 16.3 (11.7, 22.2) |
| Respiratory | 15.8 (11.3, 21.6) |
| Urological | 5.26 (2.88, 9.42) |
| Central nervous system | 2.11 (0.82, 5.29) |
| Musculoskeletal | 5.26 (2.88, 9.42) |
| Skin Lesion | 2.63 (1.13, 6.01) |
| Breast Symptoms | 0.53 (0.09, 2.92) |
| Female specific | 3.68 (1.80, 7.41) |
| Male specific | 0.53 (0.09, 2.92) |
| None recorded | 30.5 (24.4, 37.4) |
| Unknown primary (N = 1205) | |
| Non-specific | 27.7 (25.3, 30.3) |
| Lump/mass/lymph node | 8.55 (7.10, 10.3) |
| Ulceration | 0.91 (0.51, 1.63) |
| Upper abdominal | 20.5 (18.3, 22.9) |
| Lower abdominal | 25.6 (23.2, 28.1) |
| Respiratory | 12.7 (10.9, 14.7) |
| Urological | 2.90 (2.10, 4.01) |
| Central nervous system | 3.32 (2.45, 4.49) |
| Musculoskeletal | 9.88 (8.32, 11.7) |
| Skin Lesion | 0.33 (0.13, 0.85) |
| Breast Symptoms | 1.08 (0.63, 1.84) |
| Female specific | 0.91 (0.51, 1.63) |
| Male specific | 0.00 (0.00, 0.32) |
| None recorded | 20.8 (18.6, 23.2) |

## Supplementary table 4

Supplementary Table 4. Proportion of symptoms occurring in each cancer site. Proportions add up to >100% because of multiple symptoms per patient.

| Symptom Group | Symptom | Proportion of patients by cancer site % (95% CI) |
| --- | --- | --- |
| *Head and neck - Larynx (N = 368)* | | |
| Non-specific | Bruising, bleeding or petechiae | 0.00 (0.00, 1.03) |
| Non-specific | Deep vein thrombosis | 0.00 (0.00, 1.03) |
| Non-specific | Epistaxis | 0.00 (0.00, 1.03) |
| Non-specific | Fatigue | 0.27 (0.05, 1.52) |
| Non-specific | Fever | 0.27 (0.05, 1.52) |
| Non-specific | Infection | 0.00 (0.00, 1.03) |
| Non-specific | Night sweats | 0.00 (0.00, 1.03) |
| Non-specific | Other symptom | 3.80 (2.28, 6.28) |
| Non-specific | Pallor | 0.00 (0.00, 1.03) |
| Non-specific | Pruritus | 0.00 (0.00, 1.03) |
| Non-specific | Weight loss | 5.98 (3.98, 8.89) |
| Lump/mass/lymph node | Anal mass | 0.00 (0.00, 1.03) |
| Lump/mass/lymph node | Lymph node pain with alcohol | 0.00 (0.00, 1.03) |
| Lump/mass/lymph node | Lymphadenopathy (generalised) | 0.27 (0.05, 1.52) |
| Lump/mass/lymph node | Lymphadenopathy (localised) | 1.36 (0.58, 3.14) |
| Lump/mass/lymph node | Neck lump/mass | 5.71 (3.76, 8.57) |
| Lump/mass/lymph node | Thyroid lump/mass | 0.27 (0.05, 1.52) |
| Lump/mass/lymph node | Unexplained lump suspicious of sarcoma | 0.00 (0.00, 1.03) |
| Ulceration | Leukoplakia | 0.27 (0.05, 1.52) |
| Ulceration | Lip/oral cavity/tongue lump/mass | 0.54 (0.15, 1.96) |
| Ulceration | Lip/oral cavity/tongue ulcer | 0.27 (0.05, 1.52) |
| Ulceration | Ulceration | 0.00 (0.00, 1.03) |
| Upper abdominal | Dyspepsia | 0.54 (0.15, 1.96) |
| Upper abdominal | Dysphagia | 4.89 (3.12, 7.60) |
| Upper abdominal | Early satiety | 0.00 (0.00, 1.03) |
| Upper abdominal | Gastroesophageal reflux | 0.27 (0.05, 1.52) |
| Upper abdominal | Haematemesis | 0.00 (0.00, 1.03) |
| Upper abdominal | Jaundice | 0.00 (0.00, 1.03) |
| Upper abdominal | Loss of appetite | 2.17 (1.11, 4.23) |
| Upper abdominal | Nausea and/or vomiting | 0.82 (0.28, 2.37) |
| Upper abdominal | New onset diabetes | 0.00 (0.00, 1.03) |
| Upper abdominal | Upper abdominal pain | 0.00 (0.00, 1.03) |
| Lower abdominal | Abdominal pain (NOS) | 0.00 (0.00, 1.03) |
| Lower abdominal | Change in bowel habit | 0.00 (0.00, 1.03) |
| Lower abdominal | Constipation | 0.27 (0.05, 1.52) |
| Lower abdominal | Diarrhoea | 0.00 (0.00, 1.03) |
| Lower abdominal | Distension | 0.00 (0.00, 1.03) |
| Lower abdominal | Lower abdominal pain | 0.00 (0.00, 1.03) |
| Lower abdominal | Pelvic pain | 0.00 (0.00, 1.03) |
| Lower abdominal | Rectal bleeding | 0.00 (0.00, 1.03) |
| Respiratory | Chest infection | 1.09 (0.42, 2.76) |
| Respiratory | Chest pain | 0.00 (0.00, 1.03) |
| Respiratory | Clubbing | 0.00 (0.00, 1.03) |
| Respiratory | Cough | 9.51 (6.92, 12.9) |
| Respiratory | Dyspnoea | 3.80 (2.28, 6.28) |
| Respiratory | Haemoptysis | 1.09 (0.42, 2.76) |
| Respiratory | Hoarseness | 68.8 (63.8, 73.3) |
| Respiratory | Sore throat | 18.8 (15.1, 23.1) |
| Respiratory | Stridor | 1.09 (0.42, 2.76) |
| Urological | Dysuria | 0.00 (0.00, 1.03) |
| Urological | Haematuria | 0.00 (0.00, 1.03) |
| Urological | Loin pain | 0.00 (0.00, 1.03) |
| Urological | LUTS (nocturia, frequency, hesitancy, urgency, retention) | 0.00 (0.00, 1.03) |
| Urological | Renal colic | 0.00 (0.00, 1.03) |
| Urological | Urinary tract infection | 0.00 (0.00, 1.03) |
| Central nervous system | Fit/seizure | 0.00 (0.00, 1.03) |
| Central nervous system | Headache | 0.27 (0.05, 1.52) |
| Central nervous system | Prog/sub-acute loss of central neuro funct | 0.00 (0.00, 1.03) |
| Central nervous system | Visual disturbance or loss | 0.00 (0.00, 1.03) |
| Musculoskeletal | Back pain | 0.00 (0.00, 1.03) |
| Musculoskeletal | Bone pain | 0.00 (0.00, 1.03) |
| Musculoskeletal | Fracture | 0.00 (0.00, 1.03) |
| Skin Lesion | Abnormal mole | 0.00 (0.00, 1.03) |
| Skin Lesion | Lesions suspicious of BCC | 0.00 (0.00, 1.03) |
| Skin Lesion | Non-pigmented lesion | 0.00 (0.00, 1.03) |
| Breast Symptoms | Axillary lump/mass | 0.00 (0.00, 1.03) |
| Breast Symptoms | Breast lump/mass | 0.00 (0.00, 1.03) |
| Breast Symptoms | Breast pain | 0.00 (0.00, 1.03) |
| Breast Symptoms | Nipple changes | 0.00 (0.00, 1.03) |
| Breast Symptoms | Nipple discharge | 0.00 (0.00, 1.03) |
| Female specific | Other vaginal bleeding | 0.00 (0.00, 1.03) |
| Female specific | Post-menopausal bleeding | 0.00 (0.00, 1.03) |
| Female specific | Vaginal discharge | 0.00 (0.00, 1.03) |
| Female specific | Vaginal mass | 0.00 (0.00, 1.03) |
| Female specific | Vulval bleeding | 0.00 (0.00, 1.03) |
| Female specific | Vulval mass | 0.00 (0.00, 1.03) |
| Female specific | Vulval ulceration | 0.00 (0.00, 1.03) |
| Male specific | Erectile dysfunction | 0.00 (0.00, 1.03) |
| Male specific | Penile ulceration | 0.00 (0.00, 1.03) |
| Male specific | Testicular lump | 0.00 (0.00, 1.03) |
| Male specific | Testicular pain | 0.00 (0.00, 1.03) |
| None recorded | N/A | 4.89 (3.12, 7.60) |
| None recorded | N/K | 3.26 (1.88, 5.61) |
| *Head and neck - Oral cavity (N = 585)* | | |
| Non-specific | Bruising, bleeding or petechiae | 0.00 (0.00, 0.65) |
| Non-specific | Deep vein thrombosis | 0.00 (0.00, 0.65) |
| Non-specific | Epistaxis | 0.34 (0.09, 1.24) |
| Non-specific | Fatigue | 0.34 (0.09, 1.24) |
| Non-specific | Fever | 0.00 (0.00, 0.65) |
| Non-specific | Infection | 0.34 (0.09, 1.24) |
| Non-specific | Night sweats | 0.00 (0.00, 0.65) |
| Non-specific | Other symptom | 7.01 (5.21, 9.37) |
| Non-specific | Pallor | 0.00 (0.00, 0.65) |
| Non-specific | Pruritus | 0.00 (0.00, 0.65) |
| Non-specific | Weight loss | 2.05 (1.18, 3.55) |
| Lump/mass/lymph node | Anal mass | 0.00 (0.00, 0.65) |
| Lump/mass/lymph node | Lymph node pain with alcohol | 0.00 (0.00, 0.65) |
| Lump/mass/lymph node | Lymphadenopathy (generalised) | 0.00 (0.00, 0.65) |
| Lump/mass/lymph node | Lymphadenopathy (localised) | 1.37 (0.69, 2.68) |
| Lump/mass/lymph node | Neck lump/mass | 5.47 (3.90, 7.62) |
| Lump/mass/lymph node | Thyroid lump/mass | 0.00 (0.00, 0.65) |
| Lump/mass/lymph node | Unexplained lump suspicious of sarcoma | 0.34 (0.09, 1.24) |
| Ulceration | Leukoplakia | 1.37 (0.69, 2.68) |
| Ulceration | Lip/oral cavity/tongue lump/mass | 30.4 (26.8, 34.3) |
| Ulceration | Lip/oral cavity/tongue ulcer | 21.2 (18.1, 24.7) |
| Ulceration | Ulceration | 1.03 (0.47, 2.22) |
| Upper abdominal | Dyspepsia | 0.00 (0.00, 0.65) |
| Upper abdominal | Dysphagia | 1.71 (0.93, 3.12) |
| Upper abdominal | Early satiety | 0.00 (0.00, 0.65) |
| Upper abdominal | Gastroesophageal reflux | 0.17 (0.03, 0.96) |
| Upper abdominal | Haematemesis | 0.00 (0.00, 0.65) |
| Upper abdominal | Jaundice | 0.00 (0.00, 0.65) |
| Upper abdominal | Loss of appetite | 0.34 (0.09, 1.24) |
| Upper abdominal | Nausea and/or vomiting | 0.00 (0.00, 0.65) |
| Upper abdominal | New onset diabetes | 0.00 (0.00, 0.65) |
| Upper abdominal | Upper abdominal pain | 0.00 (0.00, 0.65) |
| Lower abdominal | Abdominal pain (NOS) | 0.00 (0.00, 0.65) |
| Lower abdominal | Change in bowel habit | 0.00 (0.00, 0.65) |
| Lower abdominal | Constipation | 0.00 (0.00, 0.65) |
| Lower abdominal | Diarrhoea | 0.00 (0.00, 0.65) |
| Lower abdominal | Distension | 0.00 (0.00, 0.65) |
| Lower abdominal | Lower abdominal pain | 0.00 (0.00, 0.65) |
| Lower abdominal | Pelvic pain | 0.00 (0.00, 0.65) |
| Lower abdominal | Rectal bleeding | 0.00 (0.00, 0.65) |
| Respiratory | Chest infection | 0.00 (0.00, 0.65) |
| Respiratory | Chest pain | 0.00 (0.00, 0.65) |
| Respiratory | Clubbing | 0.00 (0.00, 0.65) |
| Respiratory | Cough | 1.20 (0.58, 2.45) |
| Respiratory | Dyspnoea | 0.51 (0.17, 1.50) |
| Respiratory | Haemoptysis | 0.00 (0.00, 0.65) |
| Respiratory | Hoarseness | 1.03 (0.47, 2.22) |
| Respiratory | Sore throat | 5.47 (3.90, 7.62) |
| Respiratory | Stridor | 0.00 (0.00, 0.65) |
| Urological | Dysuria | 0.00 (0.00, 0.65) |
| Urological | Haematuria | 0.00 (0.00, 0.65) |
| Urological | Loin pain | 0.00 (0.00, 0.65) |
| Urological | LUTS (nocturia, frequency, hesitancy, urgency, retention) | 0.00 (0.00, 0.65) |
| Urological | Renal colic | 0.00 (0.00, 0.65) |
| Urological | Urinary tract infection | 0.00 (0.00, 0.65) |
| Central nervous system | Fit/seizure | 0.00 (0.00, 0.65) |
| Central nervous system | Headache | 0.00 (0.00, 0.65) |
| Central nervous system | Prog/sub-acute loss of central neuro funct | 0.00 (0.00, 0.65) |
| Central nervous system | Visual disturbance or loss | 0.00 (0.00, 0.65) |
| Musculoskeletal | Back pain | 0.17 (0.03, 0.96) |
| Musculoskeletal | Bone pain | 0.17 (0.03, 0.96) |
| Musculoskeletal | Fracture | 0.00 (0.00, 0.65) |
| Skin Lesion | Abnormal mole | 0.00 (0.00, 0.65) |
| Skin Lesion | Lesions suspicious of BCC | 0.17 (0.03, 0.96) |
| Skin Lesion | Non-pigmented lesion | 0.00 (0.00, 0.65) |
| Breast Symptoms | Axillary lump/mass | 0.00 (0.00, 0.65) |
| Breast Symptoms | Breast lump/mass | 0.17 (0.03, 0.96) |
| Breast Symptoms | Breast pain | 0.00 (0.00, 0.65) |
| Breast Symptoms | Nipple changes | 0.00 (0.00, 0.65) |
| Breast Symptoms | Nipple discharge | 0.00 (0.00, 0.65) |
| Female specific | Other vaginal bleeding | 0.17 (0.03, 0.96) |
| Female specific | Post-menopausal bleeding | 0.00 (0.00, 0.65) |
| Female specific | Vaginal discharge | 0.00 (0.00, 0.65) |
| Female specific | Vaginal mass | 0.00 (0.00, 0.65) |
| Female specific | Vulval bleeding | 0.00 (0.00, 0.65) |
| Female specific | Vulval mass | 0.00 (0.00, 0.65) |
| Female specific | Vulval ulceration | 0.00 (0.00, 0.65) |
| Male specific | Erectile dysfunction | 0.00 (0.00, 0.65) |
| Male specific | Penile ulceration | 0.00 (0.00, 0.65) |
| Male specific | Testicular lump | 0.00 (0.00, 0.65) |
| Male specific | Testicular pain | 0.00 (0.00, 0.65) |
| None recorded | N/A | 20.0 (17.0, 23.4) |
| None recorded | N/K | 9.40 (7.29, 12.0) |
| *Head and neck - Oropharynx (N = 653)* | | |
| Non-specific | Bruising, bleeding or petechiae | 0.15 (0.03, 0.86) |
| Non-specific | Deep vein thrombosis | 0.15 (0.03, 0.86) |
| Non-specific | Epistaxis | 0.46 (0.16, 1.34) |
| Non-specific | Fatigue | 0.92 (0.42, 1.99) |
| Non-specific | Fever | 0.00 (0.00, 0.58) |
| Non-specific | Infection | 0.00 (0.00, 0.58) |
| Non-specific | Night sweats | 0.31 (0.08, 1.11) |
| Non-specific | Other symptom | 7.66 (5.86, 9.95) |
| Non-specific | Pallor | 0.15 (0.03, 0.86) |
| Non-specific | Pruritus | 0.00 (0.00, 0.58) |
| Non-specific | Weight loss | 4.44 (3.11, 6.31) |
| Lump/mass/lymph node | Anal mass | 0.00 (0.00, 0.58) |
| Lump/mass/lymph node | Lymph node pain with alcohol | 0.00 (0.00, 0.58) |
| Lump/mass/lymph node | Lymphadenopathy (generalised) | 0.31 (0.08, 1.11) |
| Lump/mass/lymph node | Lymphadenopathy (localised) | 5.36 (3.88, 7.36) |
| Lump/mass/lymph node | Neck lump/mass | 43.2 (39.4, 47.0) |
| Lump/mass/lymph node | Thyroid lump/mass | 0.00 (0.00, 0.58) |
| Lump/mass/lymph node | Unexplained lump suspicious of sarcoma | 0.00 (0.00, 0.58) |
| Ulceration | Leukoplakia | 0.15 (0.03, 0.86) |
| Ulceration | Lip/oral cavity/tongue lump/mass | 7.66 (5.86, 9.95) |
| Ulceration | Lip/oral cavity/tongue ulcer | 2.76 (1.75, 4.31) |
| Ulceration | Ulceration | 0.15 (0.03, 0.86) |
| Upper abdominal | Dyspepsia | 0.00 (0.00, 0.58) |
| Upper abdominal | Dysphagia | 5.36 (3.88, 7.36) |
| Upper abdominal | Early satiety | 0.15 (0.03, 0.86) |
| Upper abdominal | Gastroesophageal reflux | 0.46 (0.16, 1.34) |
| Upper abdominal | Haematemesis | 0.15 (0.03, 0.86) |
| Upper abdominal | Jaundice | 0.00 (0.00, 0.58) |
| Upper abdominal | Loss of appetite | 1.23 (0.62, 2.40) |
| Upper abdominal | Nausea and/or vomiting | 0.31 (0.08, 1.11) |
| Upper abdominal | New onset diabetes | 0.00 (0.00, 0.58) |
| Upper abdominal | Upper abdominal pain | 0.15 (0.03, 0.86) |
| Lower abdominal | Abdominal pain (NOS) | 0.00 (0.00, 0.58) |
| Lower abdominal | Change in bowel habit | 0.00 (0.00, 0.58) |
| Lower abdominal | Constipation | 0.00 (0.00, 0.58) |
| Lower abdominal | Diarrhoea | 0.00 (0.00, 0.58) |
| Lower abdominal | Distension | 0.00 (0.00, 0.58) |
| Lower abdominal | Lower abdominal pain | 0.15 (0.03, 0.86) |
| Lower abdominal | Pelvic pain | 0.00 (0.00, 0.58) |
| Lower abdominal | Rectal bleeding | 0.00 (0.00, 0.58) |
| Respiratory | Chest infection | 0.46 (0.16, 1.34) |
| Respiratory | Chest pain | 0.77 (0.33, 1.78) |
| Respiratory | Clubbing | 0.15 (0.03, 0.86) |
| Respiratory | Cough | 2.91 (1.87, 4.50) |
| Respiratory | Dyspnoea | 0.61 (0.24, 1.56) |
| Respiratory | Haemoptysis | 1.07 (0.52, 2.20) |
| Respiratory | Hoarseness | 4.13 (2.86, 5.95) |
| Respiratory | Sore throat | 28.0 (24.7, 31.6) |
| Respiratory | Stridor | 0.00 (0.00, 0.58) |
| Urological | Dysuria | 0.00 (0.00, 0.58) |
| Urological | Haematuria | 0.00 (0.00, 0.58) |
| Urological | Loin pain | 0.00 (0.00, 0.58) |
| Urological | LUTS (nocturia, frequency, hesitancy, urgency, retention) | 0.15 (0.03, 0.86) |
| Urological | Renal colic | 0.00 (0.00, 0.58) |
| Urological | Urinary tract infection | 0.00 (0.00, 0.58) |
| Central nervous system | Fit/seizure | 0.15 (0.03, 0.86) |
| Central nervous system | Headache | 0.92 (0.42, 1.99) |
| Central nervous system | Prog/sub-acute loss of central neuro funct | 0.15 (0.03, 0.86) |
| Central nervous system | Visual disturbance or loss | 0.00 (0.00, 0.58) |
| Musculoskeletal | Back pain | 0.15 (0.03, 0.86) |
| Musculoskeletal | Bone pain | 0.00 (0.00, 0.58) |
| Musculoskeletal | Fracture | 0.00 (0.00, 0.58) |
| Skin Lesion | Abnormal mole | 0.15 (0.03, 0.86) |
| Skin Lesion | Lesions suspicious of BCC | 0.00 (0.00, 0.58) |
| Skin Lesion | Non-pigmented lesion | 0.00 (0.00, 0.58) |
| Breast Symptoms | Axillary lump/mass | 0.00 (0.00, 0.58) |
| Breast Symptoms | Breast lump/mass | 0.00 (0.00, 0.58) |
| Breast Symptoms | Breast pain | 0.00 (0.00, 0.58) |
| Breast Symptoms | Nipple changes | 0.00 (0.00, 0.58) |
| Breast Symptoms | Nipple discharge | 0.00 (0.00, 0.58) |
| Female specific | Other vaginal bleeding | 0.00 (0.00, 0.58) |
| Female specific | Post-menopausal bleeding | 0.00 (0.00, 0.58) |
| Female specific | Vaginal discharge | 0.00 (0.00, 0.58) |
| Female specific | Vaginal mass | 0.00 (0.00, 0.58) |
| Female specific | Vulval bleeding | 0.15 (0.03, 0.86) |
| Female specific | Vulval mass | 0.00 (0.00, 0.58) |
| Female specific | Vulval ulceration | 0.00 (0.00, 0.58) |
| Male specific | Erectile dysfunction | 0.00 (0.00, 0.58) |
| Male specific | Penile ulceration | 0.00 (0.00, 0.58) |
| Male specific | Testicular lump | 0.00 (0.00, 0.58) |
| Male specific | Testicular pain | 0.00 (0.00, 0.58) |
| None recorded | N/A | 5.36 (3.88, 7.36) |
| None recorded | N/K | 2.45 (1.51, 3.94) |
| *Head and neck - Thyroid (N = 633)* | | |
| Non-specific | Bruising, bleeding or petechiae | 0.00 (0.00, 0.60) |
| Non-specific | Deep vein thrombosis | 0.00 (0.00, 0.60) |
| Non-specific | Epistaxis | 0.00 (0.00, 0.60) |
| Non-specific | Fatigue | 3.00 (1.93, 4.64) |
| Non-specific | Fever | 0.16 (0.03, 0.89) |
| Non-specific | Infection | 0.00 (0.00, 0.60) |
| Non-specific | Night sweats | 0.16 (0.03, 0.89) |
| Non-specific | Other symptom | 6.32 (4.67, 8.49) |
| Non-specific | Pallor | 0.00 (0.00, 0.60) |
| Non-specific | Pruritus | 0.00 (0.00, 0.60) |
| Non-specific | Weight loss | 2.05 (1.20, 3.48) |
| Lump/mass/lymph node | Anal mass | 0.00 (0.00, 0.60) |
| Lump/mass/lymph node | Lymph node pain with alcohol | 0.00 (0.00, 0.60) |
| Lump/mass/lymph node | Lymphadenopathy (generalised) | 0.16 (0.03, 0.89) |
| Lump/mass/lymph node | Lymphadenopathy (localised) | 0.47 (0.16, 1.38) |
| Lump/mass/lymph node | Neck lump/mass | 33.0 (29.5, 36.8) |
| Lump/mass/lymph node | Thyroid lump/mass | 22.9 (19.8, 26.3) |
| Lump/mass/lymph node | Unexplained lump suspicious of sarcoma | 0.16 (0.03, 0.89) |
| Ulceration | Leukoplakia | 0.00 (0.00, 0.60) |
| Ulceration | Lip/oral cavity/tongue lump/mass | 0.16 (0.03, 0.89) |
| Ulceration | Lip/oral cavity/tongue ulcer | 0.00 (0.00, 0.60) |
| Ulceration | Ulceration | 0.00 (0.00, 0.60) |
| Upper abdominal | Dyspepsia | 0.00 (0.00, 0.60) |
| Upper abdominal | Dysphagia | 2.69 (1.68, 4.26) |
| Upper abdominal | Early satiety | 0.00 (0.00, 0.60) |
| Upper abdominal | Gastroesophageal reflux | 0.00 (0.00, 0.60) |
| Upper abdominal | Haematemesis | 0.00 (0.00, 0.60) |
| Upper abdominal | Jaundice | 0.00 (0.00, 0.60) |
| Upper abdominal | Loss of appetite | 0.32 (0.09, 1.14) |
| Upper abdominal | Nausea and/or vomiting | 0.32 (0.09, 1.14) |
| Upper abdominal | New onset diabetes | 0.16 (0.03, 0.89) |
| Upper abdominal | Upper abdominal pain | 0.16 (0.03, 0.89) |
| Lower abdominal | Abdominal pain (NOS) | 0.16 (0.03, 0.89) |
| Lower abdominal | Change in bowel habit | 0.47 (0.16, 1.38) |
| Lower abdominal | Constipation | 0.16 (0.03, 0.89) |
| Lower abdominal | Diarrhoea | 0.00 (0.00, 0.60) |
| Lower abdominal | Distension | 0.16 (0.03, 0.89) |
| Lower abdominal | Lower abdominal pain | 0.00 (0.00, 0.60) |
| Lower abdominal | Pelvic pain | 0.16 (0.03, 0.89) |
| Lower abdominal | Rectal bleeding | 0.00 (0.00, 0.60) |
| Respiratory | Chest infection | 0.32 (0.09, 1.14) |
| Respiratory | Chest pain | 0.79 (0.34, 1.84) |
| Respiratory | Clubbing | 0.16 (0.03, 0.89) |
| Respiratory | Cough | 3.32 (2.18, 5.02) |
| Respiratory | Dyspnoea | 2.05 (1.20, 3.48) |
| Respiratory | Haemoptysis | 0.32 (0.09, 1.14) |
| Respiratory | Hoarseness | 4.11 (2.82, 5.95) |
| Respiratory | Sore throat | 4.27 (2.95, 6.13) |
| Respiratory | Stridor | 0.47 (0.16, 1.38) |
| Urological | Dysuria | 0.00 (0.00, 0.60) |
| Urological | Haematuria | 0.00 (0.00, 0.60) |
| Urological | Loin pain | 0.00 (0.00, 0.60) |
| Urological | LUTS (nocturia, frequency, hesitancy, urgency, retention) | 0.16 (0.03, 0.89) |
| Urological | Renal colic | 0.00 (0.00, 0.60) |
| Urological | Urinary tract infection | 0.00 (0.00, 0.60) |
| Central nervous system | Fit/seizure | 0.16 (0.03, 0.89) |
| Central nervous system | Headache | 0.32 (0.09, 1.14) |
| Central nervous system | Prog/sub-acute loss of central neuro funct | 0.00 (0.00, 0.60) |
| Central nervous system | Visual disturbance or loss | 0.00 (0.00, 0.60) |
| Musculoskeletal | Back pain | 1.11 (0.54, 2.26) |
| Musculoskeletal | Bone pain | 0.47 (0.16, 1.38) |
| Musculoskeletal | Fracture | 0.16 (0.03, 0.89) |
| Skin Lesion | Abnormal mole | 0.00 (0.00, 0.60) |
| Skin Lesion | Lesions suspicious of BCC | 0.00 (0.00, 0.60) |
| Skin Lesion | Non-pigmented lesion | 0.00 (0.00, 0.60) |
| Breast Symptoms | Axillary lump/mass | 0.16 (0.03, 0.89) |
| Breast Symptoms | Breast lump/mass | 0.47 (0.16, 1.38) |
| Breast Symptoms | Breast pain | 0.32 (0.09, 1.14) |
| Breast Symptoms | Nipple changes | 0.00 (0.00, 0.60) |
| Breast Symptoms | Nipple discharge | 0.00 (0.00, 0.60) |
| Female specific | Other vaginal bleeding | 0.00 (0.00, 0.60) |
| Female specific | Post-menopausal bleeding | 0.00 (0.00, 0.60) |
| Female specific | Vaginal discharge | 0.00 (0.00, 0.60) |
| Female specific | Vaginal mass | 0.00 (0.00, 0.60) |
| Female specific | Vulval bleeding | 0.00 (0.00, 0.60) |
| Female specific | Vulval mass | 0.00 (0.00, 0.60) |
| Female specific | Vulval ulceration | 0.00 (0.00, 0.60) |
| Male specific | Erectile dysfunction | 0.00 (0.00, 0.60) |
| Male specific | Penile ulceration | 0.00 (0.00, 0.60) |
| Male specific | Testicular lump | 0.00 (0.00, 0.60) |
| Male specific | Testicular pain | 0.00 (0.00, 0.60) |
| None recorded | N/A | 21.8 (18.8, 25.2) |
| None recorded | N/K | 4.58 (3.21, 6.50) |
| *Head and neck - Other head and neck (N = 481)* | | |
| Non-specific | Bruising, bleeding or petechiae | 0.00 (0.00, 0.79) |
| Non-specific | Deep vein thrombosis | 0.00 (0.00, 0.79) |
| Non-specific | Epistaxis | 5.61 (3.89, 8.04) |
| Non-specific | Fatigue | 0.62 (0.21, 1.82) |
| Non-specific | Fever | 0.42 (0.11, 1.50) |
| Non-specific | Infection | 1.04 (0.44, 2.41) |
| Non-specific | Night sweats | 0.00 (0.00, 0.79) |
| Non-specific | Other symptom | 12.9 (10.2, 16.2) |
| Non-specific | Pallor | 0.21 (0.04, 1.17) |
| Non-specific | Pruritus | 0.00 (0.00, 0.79) |
| Non-specific | Weight loss | 3.53 (2.22, 5.59) |
| Lump/mass/lymph node | Anal mass | 0.00 (0.00, 0.79) |
| Lump/mass/lymph node | Lymph node pain with alcohol | 0.21 (0.04, 1.17) |
| Lump/mass/lymph node | Lymphadenopathy (generalised) | 0.00 (0.00, 0.79) |
| Lump/mass/lymph node | Lymphadenopathy (localised) | 4.37 (2.87, 6.58) |
| Lump/mass/lymph node | Neck lump/mass | 30.6 (26.6, 34.8) |
| Lump/mass/lymph node | Thyroid lump/mass | 0.00 (0.00, 0.79) |
| Lump/mass/lymph node | Unexplained lump suspicious of sarcoma | 0.42 (0.11, 1.50) |
| Ulceration | Leukoplakia | 0.00 (0.00, 0.79) |
| Ulceration | Lip/oral cavity/tongue lump/mass | 8.11 (5.99, 10.9) |
| Ulceration | Lip/oral cavity/tongue ulcer | 3.12 (1.90, 5.08) |
| Ulceration | Ulceration | 2.49 (1.43, 4.31) |
| Upper abdominal | Dyspepsia | 0.42 (0.11, 1.50) |
| Upper abdominal | Dysphagia | 7.69 (5.63, 10.4) |
| Upper abdominal | Early satiety | 0.00 (0.00, 0.79) |
| Upper abdominal | Gastroesophageal reflux | 0.21 (0.04, 1.17) |
| Upper abdominal | Haematemesis | 0.21 (0.04, 1.17) |
| Upper abdominal | Jaundice | 0.00 (0.00, 0.79) |
| Upper abdominal | Loss of appetite | 0.83 (0.32, 2.12) |
| Upper abdominal | Nausea and/or vomiting | 0.42 (0.11, 1.50) |
| Upper abdominal | New onset diabetes | 0.00 (0.00, 0.79) |
| Upper abdominal | Upper abdominal pain | 0.00 (0.00, 0.79) |
| Lower abdominal | Abdominal pain (NOS) | 0.00 (0.00, 0.79) |
| Lower abdominal | Change in bowel habit | 0.00 (0.00, 0.79) |
| Lower abdominal | Constipation | 0.21 (0.04, 1.17) |
| Lower abdominal | Diarrhoea | 0.00 (0.00, 0.79) |
| Lower abdominal | Distension | 0.00 (0.00, 0.79) |
| Lower abdominal | Lower abdominal pain | 0.42 (0.11, 1.50) |
| Lower abdominal | Pelvic pain | 0.21 (0.04, 1.17) |
| Lower abdominal | Rectal bleeding | 0.21 (0.04, 1.17) |
| Respiratory | Chest infection | 0.21 (0.04, 1.17) |
| Respiratory | Chest pain | 0.42 (0.11, 1.50) |
| Respiratory | Clubbing | 0.00 (0.00, 0.79) |
| Respiratory | Cough | 1.66 (0.85, 3.25) |
| Respiratory | Dyspnoea | 0.42 (0.11, 1.50) |
| Respiratory | Haemoptysis | 0.42 (0.11, 1.50) |
| Respiratory | Hoarseness | 7.28 (5.28, 9.95) |
| Respiratory | Sore throat | 11.6 (9.08, 14.8) |
| Respiratory | Stridor | 0.42 (0.11, 1.50) |
| Urological | Dysuria | 0.00 (0.00, 0.79) |
| Urological | Haematuria | 0.00 (0.00, 0.79) |
| Urological | Loin pain | 0.00 (0.00, 0.79) |
| Urological | LUTS (nocturia, frequency, hesitancy, urgency, retention) | 0.00 (0.00, 0.79) |
| Urological | Renal colic | 0.00 (0.00, 0.79) |
| Urological | Urinary tract infection | 0.00 (0.00, 0.79) |
| Central nervous system | Fit/seizure | 0.00 (0.00, 0.79) |
| Central nervous system | Headache | 3.33 (2.06, 5.33) |
| Central nervous system | Prog/sub-acute loss of central neuro funct | 0.83 (0.32, 2.12) |
| Central nervous system | Visual disturbance or loss | 1.25 (0.57, 2.69) |
| Musculoskeletal | Back pain | 0.00 (0.00, 0.79) |
| Musculoskeletal | Bone pain | 0.00 (0.00, 0.79) |
| Musculoskeletal | Fracture | 0.00 (0.00, 0.79) |
| Skin Lesion | Abnormal mole | 0.00 (0.00, 0.79) |
| Skin Lesion | Lesions suspicious of BCC | 0.42 (0.11, 1.50) |
| Skin Lesion | Non-pigmented lesion | 0.00 (0.00, 0.79) |
| Breast Symptoms | Axillary lump/mass | 0.00 (0.00, 0.79) |
| Breast Symptoms | Breast lump/mass | 0.00 (0.00, 0.79) |
| Breast Symptoms | Breast pain | 0.00 (0.00, 0.79) |
| Breast Symptoms | Nipple changes | 0.00 (0.00, 0.79) |
| Breast Symptoms | Nipple discharge | 0.00 (0.00, 0.79) |
| Female specific | Other vaginal bleeding | 0.00 (0.00, 0.79) |
| Female specific | Post-menopausal bleeding | 0.00 (0.00, 0.79) |
| Female specific | Vaginal discharge | 0.00 (0.00, 0.79) |
| Female specific | Vaginal mass | 0.00 (0.00, 0.79) |
| Female specific | Vulval bleeding | 0.00 (0.00, 0.79) |
| Female specific | Vulval mass | 0.00 (0.00, 0.79) |
| Female specific | Vulval ulceration | 0.00 (0.00, 0.79) |
| Male specific | Erectile dysfunction | 0.00 (0.00, 0.79) |
| Male specific | Penile ulceration | 0.00 (0.00, 0.79) |
| Male specific | Testicular lump | 0.00 (0.00, 0.79) |
| Male specific | Testicular pain | 0.00 (0.00, 0.79) |
| None recorded | N/A | 7.48 (5.45, 10.2) |
| None recorded | N/K | 3.95 (2.54, 6.09) |
| *Upper gastrointestinal - Oesophagus (N = 1391)* | | |
| Non-specific | Bruising, bleeding or petechiae | 0.14 (0.04, 0.52) |
| Non-specific | Deep vein thrombosis | 0.22 (0.07, 0.63) |
| Non-specific | Epistaxis | 0.07 (0.01, 0.41) |
| Non-specific | Fatigue | 3.24 (2.43, 4.30) |
| Non-specific | Fever | 0.07 (0.01, 0.41) |
| Non-specific | Infection | 0.22 (0.07, 0.63) |
| Non-specific | Night sweats | 0.43 (0.20, 0.94) |
| Non-specific | Other symptom | 4.24 (3.30, 5.43) |
| Non-specific | Pallor | 0.14 (0.04, 0.52) |
| Non-specific | Pruritus | 0.14 (0.04, 0.52) |
| Non-specific | Weight loss | 20.8 (18.8, 23.1) |
| Lump/mass/lymph node | Anal mass | 0.00 (0.00, 0.28) |
| Lump/mass/lymph node | Lymph node pain with alcohol | 0.00 (0.00, 0.28) |
| Lump/mass/lymph node | Lymphadenopathy (generalised) | 0.00 (0.00, 0.28) |
| Lump/mass/lymph node | Lymphadenopathy (localised) | 0.22 (0.07, 0.63) |
| Lump/mass/lymph node | Neck lump/mass | 0.43 (0.20, 0.94) |
| Lump/mass/lymph node | Thyroid lump/mass | 0.00 (0.00, 0.28) |
| Lump/mass/lymph node | Unexplained lump suspicious of sarcoma | 0.00 (0.00, 0.28) |
| Ulceration | Leukoplakia | 0.00 (0.00, 0.28) |
| Ulceration | Lip/oral cavity/tongue lump/mass | 0.22 (0.07, 0.63) |
| Ulceration | Lip/oral cavity/tongue ulcer | 0.00 (0.00, 0.28) |
| Ulceration | Ulceration | 0.00 (0.00, 0.28) |
| Upper abdominal | Dyspepsia | 18.5 (16.5, 20.6) |
| Upper abdominal | Dysphagia | 46.0 (43.4, 48.6) |
| Upper abdominal | Early satiety | 1.58 (1.05, 2.38) |
| Upper abdominal | Gastroesophageal reflux | 8.12 (6.80, 9.68) |
| Upper abdominal | Haematemesis | 1.22 (0.76, 1.95) |
| Upper abdominal | Jaundice | 0.22 (0.07, 0.63) |
| Upper abdominal | Loss of appetite | 5.25 (4.19, 6.55) |
| Upper abdominal | Nausea and/or vomiting | 11.9 (10.3, 13.7) |
| Upper abdominal | New onset diabetes | 0.00 (0.00, 0.28) |
| Upper abdominal | Upper abdominal pain | 9.49 (8.06, 11.1) |
| Lower abdominal | Abdominal pain (NOS) | 3.45 (2.61, 4.55) |
| Lower abdominal | Change in bowel habit | 1.73 (1.16, 2.55) |
| Lower abdominal | Constipation | 1.58 (1.05, 2.38) |
| Lower abdominal | Diarrhoea | 0.79 (0.44, 1.41) |
| Lower abdominal | Distension | 1.58 (1.05, 2.38) |
| Lower abdominal | Lower abdominal pain | 0.79 (0.44, 1.41) |
| Lower abdominal | Pelvic pain | 0.00 (0.00, 0.28) |
| Lower abdominal | Rectal bleeding | 1.08 (0.65, 1.77) |
| Respiratory | Chest infection | 0.29 (0.11, 0.74) |
| Respiratory | Chest pain | 4.24 (3.30, 5.43) |
| Respiratory | Clubbing | 0.07 (0.01, 0.41) |
| Respiratory | Cough | 3.02 (2.24, 4.06) |
| Respiratory | Dyspnoea | 1.94 (1.34, 2.81) |
| Respiratory | Haemoptysis | 0.14 (0.04, 0.52) |
| Respiratory | Hoarseness | 0.79 (0.44, 1.41) |
| Respiratory | Sore throat | 1.58 (1.05, 2.38) |
| Respiratory | Stridor | 0.00 (0.00, 0.28) |
| Urological | Dysuria | 0.00 (0.00, 0.28) |
| Urological | Haematuria | 0.07 (0.01, 0.41) |
| Urological | Loin pain | 0.14 (0.04, 0.52) |
| Urological | LUTS (nocturia, frequency, hesitancy, urgency, retention) | 0.07 (0.01, 0.41) |
| Urological | Renal colic | 0.07 (0.01, 0.41) |
| Urological | Urinary tract infection | 0.00 (0.00, 0.28) |
| Central nervous system | Fit/seizure | 0.00 (0.00, 0.28) |
| Central nervous system | Headache | 0.22 (0.07, 0.63) |
| Central nervous system | Prog/sub-acute loss of central neuro funct | 0.14 (0.04, 0.52) |
| Central nervous system | Visual disturbance or loss | 0.00 (0.00, 0.28) |
| Musculoskeletal | Back pain | 1.37 (0.88, 2.12) |
| Musculoskeletal | Bone pain | 0.43 (0.20, 0.94) |
| Musculoskeletal | Fracture | 0.00 (0.00, 0.28) |
| Skin Lesion | Abnormal mole | 0.00 (0.00, 0.28) |
| Skin Lesion | Lesions suspicious of BCC | 0.00 (0.00, 0.28) |
| Skin Lesion | Non-pigmented lesion | 0.00 (0.00, 0.28) |
| Breast Symptoms | Axillary lump/mass | 0.07 (0.01, 0.41) |
| Breast Symptoms | Breast lump/mass | 0.14 (0.04, 0.52) |
| Breast Symptoms | Breast pain | 0.00 (0.00, 0.28) |
| Breast Symptoms | Nipple changes | 0.00 (0.00, 0.28) |
| Breast Symptoms | Nipple discharge | 0.00 (0.00, 0.28) |
| Female specific | Other vaginal bleeding | 0.00 (0.00, 0.28) |
| Female specific | Post-menopausal bleeding | 0.00 (0.00, 0.28) |
| Female specific | Vaginal discharge | 0.00 (0.00, 0.28) |
| Female specific | Vaginal mass | 0.00 (0.00, 0.28) |
| Female specific | Vulval bleeding | 0.00 (0.00, 0.28) |
| Female specific | Vulval mass | 0.00 (0.00, 0.28) |
| Female specific | Vulval ulceration | 0.00 (0.00, 0.28) |
| Male specific | Erectile dysfunction | 0.00 (0.00, 0.28) |
| Male specific | Penile ulceration | 0.00 (0.00, 0.28) |
| Male specific | Testicular lump | 0.00 (0.00, 0.28) |
| Male specific | Testicular pain | 0.00 (0.00, 0.28) |
| None recorded | N/A | 7.98 (6.67, 9.52) |
| None recorded | N/K | 2.23 (1.57, 3.15) |
| *Upper gastrointestinal - Stomach (N = 1029)* | | |
| Non-specific | Bruising, bleeding or petechiae | 0.10 (0.02, 0.55) |
| Non-specific | Deep vein thrombosis | 0.10 (0.02, 0.55) |
| Non-specific | Epistaxis | 0.00 (0.00, 0.37) |
| Non-specific | Fatigue | 6.80 (5.42, 8.51) |
| Non-specific | Fever | 0.49 (0.21, 1.13) |
| Non-specific | Infection | 0.29 (0.10, 0.85) |
| Non-specific | Night sweats | 0.29 (0.10, 0.85) |
| Non-specific | Other symptom | 6.32 (4.99, 7.97) |
| Non-specific | Pallor | 1.65 (1.03, 2.63) |
| Non-specific | Pruritus | 0.19 (0.05, 0.71) |
| Non-specific | Weight loss | 20.7 (18.3, 23.3) |
| Lump/mass/lymph node | Anal mass | 0.00 (0.00, 0.37) |
| Lump/mass/lymph node | Lymph node pain with alcohol | 0.00 (0.00, 0.37) |
| Lump/mass/lymph node | Lymphadenopathy (generalised) | 0.00 (0.00, 0.37) |
| Lump/mass/lymph node | Lymphadenopathy (localised) | 0.10 (0.02, 0.55) |
| Lump/mass/lymph node | Neck lump/mass | 0.39 (0.15, 1.00) |
| Lump/mass/lymph node | Thyroid lump/mass | 0.10 (0.02, 0.55) |
| Lump/mass/lymph node | Unexplained lump suspicious of sarcoma | 0.10 (0.02, 0.55) |
| Ulceration | Leukoplakia | 0.00 (0.00, 0.37) |
| Ulceration | Lip/oral cavity/tongue lump/mass | 0.00 (0.00, 0.37) |
| Ulceration | Lip/oral cavity/tongue ulcer | 0.10 (0.02, 0.55) |
| Ulceration | Ulceration | 0.00 (0.00, 0.37) |
| Upper abdominal | Dyspepsia | 17.2 (15.0, 19.6) |
| Upper abdominal | Dysphagia | 15.2 (13.1, 17.5) |
| Upper abdominal | Early satiety | 2.53 (1.73, 3.68) |
| Upper abdominal | Gastroesophageal reflux | 5.64 (4.39, 7.22) |
| Upper abdominal | Haematemesis | 2.82 (1.97, 4.02) |
| Upper abdominal | Jaundice | 0.29 (0.10, 0.85) |
| Upper abdominal | Loss of appetite | 9.14 (7.52, 11.1) |
| Upper abdominal | Nausea and/or vomiting | 12.2 (10.4, 14.4) |
| Upper abdominal | New onset diabetes | 0.00 (0.00, 0.37) |
| Upper abdominal | Upper abdominal pain | 15.0 (12.9, 17.3) |
| Lower abdominal | Abdominal pain (NOS) | 9.82 (8.14, 11.8) |
| Lower abdominal | Change in bowel habit | 3.89 (2.87, 5.25) |
| Lower abdominal | Constipation | 2.92 (2.05, 4.13) |
| Lower abdominal | Diarrhoea | 3.30 (2.37, 4.58) |
| Lower abdominal | Distension | 3.11 (2.21, 4.36) |
| Lower abdominal | Lower abdominal pain | 1.94 (1.26, 2.98) |
| Lower abdominal | Pelvic pain | 0.00 (0.00, 0.37) |
| Lower abdominal | Rectal bleeding | 3.50 (2.54, 4.81) |
| Respiratory | Chest infection | 0.58 (0.27, 1.27) |
| Respiratory | Chest pain | 1.85 (1.19, 2.87) |
| Respiratory | Clubbing | 0.00 (0.00, 0.37) |
| Respiratory | Cough | 1.65 (1.03, 2.63) |
| Respiratory | Dyspnoea | 3.21 (2.29, 4.47) |
| Respiratory | Haemoptysis | 0.19 (0.05, 0.71) |
| Respiratory | Hoarseness | 0.00 (0.00, 0.37) |
| Respiratory | Sore throat | 0.10 (0.02, 0.55) |
| Respiratory | Stridor | 0.00 (0.00, 0.37) |
| Urological | Dysuria | 0.39 (0.15, 1.00) |
| Urological | Haematuria | 0.39 (0.15, 1.00) |
| Urological | Loin pain | 0.00 (0.00, 0.37) |
| Urological | LUTS (nocturia, frequency, hesitancy, urgency, retention) | 0.10 (0.02, 0.55) |
| Urological | Renal colic | 0.00 (0.00, 0.37) |
| Urological | Urinary tract infection | 0.29 (0.10, 0.85) |
| Central nervous system | Fit/seizure | 0.10 (0.02, 0.55) |
| Central nervous system | Headache | 0.68 (0.33, 1.40) |
| Central nervous system | Prog/sub-acute loss of central neuro funct | 0.00 (0.00, 0.37) |
| Central nervous system | Visual disturbance or loss | 0.00 (0.00, 0.37) |
| Musculoskeletal | Back pain | 1.94 (1.26, 2.98) |
| Musculoskeletal | Bone pain | 0.19 (0.05, 0.71) |
| Musculoskeletal | Fracture | 0.00 (0.00, 0.37) |
| Skin Lesion | Abnormal mole | 0.00 (0.00, 0.37) |
| Skin Lesion | Lesions suspicious of BCC | 0.00 (0.00, 0.37) |
| Skin Lesion | Non-pigmented lesion | 0.00 (0.00, 0.37) |
| Breast Symptoms | Axillary lump/mass | 0.00 (0.00, 0.37) |
| Breast Symptoms | Breast lump/mass | 0.00 (0.00, 0.37) |
| Breast Symptoms | Breast pain | 0.00 (0.00, 0.37) |
| Breast Symptoms | Nipple changes | 0.10 (0.02, 0.55) |
| Breast Symptoms | Nipple discharge | 0.10 (0.02, 0.55) |
| Female specific | Other vaginal bleeding | 0.19 (0.05, 0.71) |
| Female specific | Post-menopausal bleeding | 0.10 (0.02, 0.55) |
| Female specific | Vaginal discharge | 0.00 (0.00, 0.37) |
| Female specific | Vaginal mass | 0.00 (0.00, 0.37) |
| Female specific | Vulval bleeding | 0.00 (0.00, 0.37) |
| Female specific | Vulval mass | 0.00 (0.00, 0.37) |
| Female specific | Vulval ulceration | 0.00 (0.00, 0.37) |
| Male specific | Erectile dysfunction | 0.00 (0.00, 0.37) |
| Male specific | Penile ulceration | 0.00 (0.00, 0.37) |
| Male specific | Testicular lump | 0.00 (0.00, 0.37) |
| Male specific | Testicular pain | 0.00 (0.00, 0.37) |
| None recorded | N/A | 12.9 (11.0, 15.1) |
| None recorded | N/K | 2.43 (1.65, 3.56) |
| *Lower gastrointestinal - Anal (N = 265)* | | |
| Non-specific | Bruising, bleeding or petechiae | 0.00 (0.00, 1.43) |
| Non-specific | Deep vein thrombosis | 0.00 (0.00, 1.43) |
| Non-specific | Epistaxis | 0.00 (0.00, 1.43) |
| Non-specific | Fatigue | 2.64 (1.29, 5.35) |
| Non-specific | Fever | 0.00 (0.00, 1.43) |
| Non-specific | Infection | 1.13 (0.39, 3.27) |
| Non-specific | Night sweats | 0.00 (0.00, 1.43) |
| Non-specific | Other symptom | 5.28 (3.17, 8.67) |
| Non-specific | Pallor | 0.38 (0.07, 2.11) |
| Non-specific | Pruritus | 1.13 (0.39, 3.27) |
| Non-specific | Weight loss | 5.28 (3.17, 8.67) |
| Lump/mass/lymph node | Anal mass | 25.7 (20.8, 31.2) |
| Lump/mass/lymph node | Lymph node pain with alcohol | 0.00 (0.00, 1.43) |
| Lump/mass/lymph node | Lymphadenopathy (generalised) | 0.00 (0.00, 1.43) |
| Lump/mass/lymph node | Lymphadenopathy (localised) | 0.75 (0.21, 2.71) |
| Lump/mass/lymph node | Neck lump/mass | 0.00 (0.00, 1.43) |
| Lump/mass/lymph node | Thyroid lump/mass | 0.00 (0.00, 1.43) |
| Lump/mass/lymph node | Unexplained lump suspicious of sarcoma | 0.00 (0.00, 1.43) |
| Ulceration | Leukoplakia | 0.00 (0.00, 1.43) |
| Ulceration | Lip/oral cavity/tongue lump/mass | 0.00 (0.00, 1.43) |
| Ulceration | Lip/oral cavity/tongue ulcer | 0.00 (0.00, 1.43) |
| Ulceration | Ulceration | 1.51 (0.59, 3.82) |
| Upper abdominal | Dyspepsia | 0.00 (0.00, 1.43) |
| Upper abdominal | Dysphagia | 0.00 (0.00, 1.43) |
| Upper abdominal | Early satiety | 0.00 (0.00, 1.43) |
| Upper abdominal | Gastroesophageal reflux | 0.00 (0.00, 1.43) |
| Upper abdominal | Haematemesis | 0.00 (0.00, 1.43) |
| Upper abdominal | Jaundice | 0.00 (0.00, 1.43) |
| Upper abdominal | Loss of appetite | 1.13 (0.39, 3.27) |
| Upper abdominal | Nausea and/or vomiting | 0.00 (0.00, 1.43) |
| Upper abdominal | New onset diabetes | 0.00 (0.00, 1.43) |
| Upper abdominal | Upper abdominal pain | 0.38 (0.07, 2.11) |
| Lower abdominal | Abdominal pain (NOS) | 4.15 (2.33, 7.28) |
| Lower abdominal | Change in bowel habit | 12.8 (9.33, 17.4) |
| Lower abdominal | Constipation | 9.06 (6.16, 13.1) |
| Lower abdominal | Diarrhoea | 6.79 (4.34, 10.5) |
| Lower abdominal | Distension | 1.13 (0.39, 3.27) |
| Lower abdominal | Lower abdominal pain | 3.77 (2.06, 6.81) |
| Lower abdominal | Pelvic pain | 2.26 (1.04, 4.85) |
| Lower abdominal | Rectal bleeding | 52.8 (46.8, 58.8) |
| Respiratory | Chest infection | 0.00 (0.00, 1.43) |
| Respiratory | Chest pain | 0.00 (0.00, 1.43) |
| Respiratory | Clubbing | 0.00 (0.00, 1.43) |
| Respiratory | Cough | 0.00 (0.00, 1.43) |
| Respiratory | Dyspnoea | 0.38 (0.07, 2.11) |
| Respiratory | Haemoptysis | 0.38 (0.07, 2.11) |
| Respiratory | Hoarseness | 0.00 (0.00, 1.43) |
| Respiratory | Sore throat | 0.00 (0.00, 1.43) |
| Respiratory | Stridor | 0.00 (0.00, 1.43) |
| Urological | Dysuria | 0.00 (0.00, 1.43) |
| Urological | Haematuria | 0.00 (0.00, 1.43) |
| Urological | Loin pain | 0.00 (0.00, 1.43) |
| Urological | LUTS (nocturia, frequency, hesitancy, urgency, retention) | 0.75 (0.21, 2.71) |
| Urological | Renal colic | 0.00 (0.00, 1.43) |
| Urological | Urinary tract infection | 0.00 (0.00, 1.43) |
| Central nervous system | Fit/seizure | 0.00 (0.00, 1.43) |
| Central nervous system | Headache | 0.00 (0.00, 1.43) |
| Central nervous system | Prog/sub-acute loss of central neuro funct | 0.00 (0.00, 1.43) |
| Central nervous system | Visual disturbance or loss | 0.00 (0.00, 1.43) |
| Musculoskeletal | Back pain | 0.75 (0.21, 2.71) |
| Musculoskeletal | Bone pain | 0.38 (0.07, 2.11) |
| Musculoskeletal | Fracture | 0.38 (0.07, 2.11) |
| Skin Lesion | Abnormal mole | 0.00 (0.00, 1.43) |
| Skin Lesion | Lesions suspicious of BCC | 0.00 (0.00, 1.43) |
| Skin Lesion | Non-pigmented lesion | 0.00 (0.00, 1.43) |
| Breast Symptoms | Axillary lump/mass | 0.00 (0.00, 1.43) |
| Breast Symptoms | Breast lump/mass | 0.38 (0.07, 2.11) |
| Breast Symptoms | Breast pain | 0.38 (0.07, 2.11) |
| Breast Symptoms | Nipple changes | 0.00 (0.00, 1.43) |
| Breast Symptoms | Nipple discharge | 0.00 (0.00, 1.43) |
| Female specific | Other vaginal bleeding | 0.00 (0.00, 1.43) |
| Female specific | Post-menopausal bleeding | 0.00 (0.00, 1.43) |
| Female specific | Vaginal discharge | 0.00 (0.00, 1.43) |
| Female specific | Vaginal mass | 0.00 (0.00, 1.43) |
| Female specific | Vulval bleeding | 0.38 (0.07, 2.11) |
| Female specific | Vulval mass | 0.00 (0.00, 1.43) |
| Female specific | Vulval ulceration | 0.00 (0.00, 1.43) |
| Male specific | Erectile dysfunction | 0.00 (0.00, 1.43) |
| Male specific | Penile ulceration | 0.00 (0.00, 1.43) |
| Male specific | Testicular lump | 0.00 (0.00, 1.43) |
| Male specific | Testicular pain | 0.00 (0.00, 1.43) |
| None recorded | N/A | 6.04 (3.75, 9.58) |
| None recorded | N/K | 2.64 (1.29, 5.35) |
| *Lower gastrointestinal - Colon (N = 4209)* | | |
| Non-specific | Bruising, bleeding or petechiae | 0.12 (0.05, 0.28) |
| Non-specific | Deep vein thrombosis | 0.24 (0.13, 0.44) |
| Non-specific | Epistaxis | 0.02 (0.00, 0.13) |
| Non-specific | Fatigue | 7.41 (6.66, 8.24) |
| Non-specific | Fever | 0.69 (0.48, 0.99) |
| Non-specific | Infection | 0.14 (0.07, 0.31) |
| Non-specific | Night sweats | 0.50 (0.33, 0.76) |
| Non-specific | Other symptom | 5.46 (4.82, 6.19) |
| Non-specific | Pallor | 0.81 (0.58, 1.13) |
| Non-specific | Pruritus | 0.29 (0.16, 0.50) |
| Non-specific | Weight loss | 13.0 (12.0, 14.0) |
| Lump/mass/lymph node | Anal mass | 0.21 (0.11, 0.41) |
| Lump/mass/lymph node | Lymph node pain with alcohol | 0.00 (0.00, 0.09) |
| Lump/mass/lymph node | Lymphadenopathy (generalised) | 0.00 (0.00, 0.09) |
| Lump/mass/lymph node | Lymphadenopathy (localised) | 0.10 (0.04, 0.24) |
| Lump/mass/lymph node | Neck lump/mass | 0.10 (0.04, 0.24) |
| Lump/mass/lymph node | Thyroid lump/mass | 0.00 (0.00, 0.09) |
| Lump/mass/lymph node | Unexplained lump suspicious of sarcoma | 0.07 (0.02, 0.21) |
| Ulceration | Leukoplakia | 0.00 (0.00, 0.09) |
| Ulceration | Lip/oral cavity/tongue lump/mass | 0.02 (0.00, 0.13) |
| Ulceration | Lip/oral cavity/tongue ulcer | 0.05 (0.01, 0.17) |
| Ulceration | Ulceration | 0.02 (0.00, 0.13) |
| Upper abdominal | Dyspepsia | 1.90 (1.53, 2.36) |
| Upper abdominal | Dysphagia | 0.78 (0.56, 1.10) |
| Upper abdominal | Early satiety | 0.36 (0.22, 0.59) |
| Upper abdominal | Gastroesophageal reflux | 0.67 (0.46, 0.96) |
| Upper abdominal | Haematemesis | 0.36 (0.22, 0.59) |
| Upper abdominal | Jaundice | 0.24 (0.13, 0.44) |
| Upper abdominal | Loss of appetite | 5.16 (4.53, 5.87) |
| Upper abdominal | Nausea and/or vomiting | 7.15 (6.41, 7.97) |
| Upper abdominal | New onset diabetes | 0.02 (0.00, 0.13) |
| Upper abdominal | Upper abdominal pain | 3.99 (3.44, 4.63) |
| Lower abdominal | Abdominal pain (NOS) | 17.1 (16.0, 18.2) |
| Lower abdominal | Change in bowel habit | 15.9 (14.8, 17.1) |
| Lower abdominal | Constipation | 8.05 (7.27, 8.92) |
| Lower abdominal | Diarrhoea | 10.4 (9.54, 11.4) |
| Lower abdominal | Distension | 5.16 (4.53, 5.87) |
| Lower abdominal | Lower abdominal pain | 9.98 (9.11, 10.9) |
| Lower abdominal | Pelvic pain | 0.76 (0.54, 1.07) |
| Lower abdominal | Rectal bleeding | 17.6 (16.5, 18.8) |
| Respiratory | Chest infection | 0.29 (0.16, 0.50) |
| Respiratory | Chest pain | 0.67 (0.46, 0.96) |
| Respiratory | Clubbing | 0.07 (0.02, 0.21) |
| Respiratory | Cough | 1.14 (0.86, 1.51) |
| Respiratory | Dyspnoea | 3.92 (3.37, 4.55) |
| Respiratory | Haemoptysis | 0.10 (0.04, 0.24) |
| Respiratory | Hoarseness | 0.12 (0.05, 0.28) |
| Respiratory | Sore throat | 0.07 (0.02, 0.21) |
| Respiratory | Stridor | 0.00 (0.00, 0.09) |
| Urological | Dysuria | 0.29 (0.16, 0.50) |
| Urological | Haematuria | 0.33 (0.20, 0.56) |
| Urological | Loin pain | 0.21 (0.11, 0.41) |
| Urological | LUTS (nocturia, frequency, hesitancy, urgency, retention) | 0.81 (0.58, 1.13) |
| Urological | Renal colic | 0.02 (0.00, 0.13) |
| Urological | Urinary tract infection | 0.43 (0.27, 0.68) |
| Central nervous system | Fit/seizure | 0.02 (0.00, 0.13) |
| Central nervous system | Headache | 0.31 (0.18, 0.53) |
| Central nervous system | Prog/sub-acute loss of central neuro funct | 0.17 (0.08, 0.34) |
| Central nervous system | Visual disturbance or loss | 0.02 (0.00, 0.13) |
| Musculoskeletal | Back pain | 1.24 (0.94, 1.62) |
| Musculoskeletal | Bone pain | 0.19 (0.10, 0.37) |
| Musculoskeletal | Fracture | 0.02 (0.00, 0.13) |
| Skin Lesion | Abnormal mole | 0.00 (0.00, 0.09) |
| Skin Lesion | Lesions suspicious of BCC | 0.00 (0.00, 0.09) |
| Skin Lesion | Non-pigmented lesion | 0.00 (0.00, 0.09) |
| Breast Symptoms | Axillary lump/mass | 0.02 (0.00, 0.13) |
| Breast Symptoms | Breast lump/mass | 0.10 (0.04, 0.24) |
| Breast Symptoms | Breast pain | 0.02 (0.00, 0.13) |
| Breast Symptoms | Nipple changes | 0.02 (0.00, 0.13) |
| Breast Symptoms | Nipple discharge | 0.00 (0.00, 0.09) |
| Female specific | Other vaginal bleeding | 0.05 (0.01, 0.17) |
| Female specific | Post-menopausal bleeding | 0.07 (0.02, 0.21) |
| Female specific | Vaginal discharge | 0.02 (0.00, 0.13) |
| Female specific | Vaginal mass | 0.00 (0.00, 0.09) |
| Female specific | Vulval bleeding | 0.02 (0.00, 0.13) |
| Female specific | Vulval mass | 0.00 (0.00, 0.09) |
| Female specific | Vulval ulceration | 0.00 (0.00, 0.09) |
| Male specific | Erectile dysfunction | 0.02 (0.00, 0.13) |
| Male specific | Penile ulceration | 0.00 (0.00, 0.09) |
| Male specific | Testicular lump | 0.07 (0.02, 0.21) |
| Male specific | Testicular pain | 0.07 (0.02, 0.21) |
| None recorded | N/A | 16.5 (15.4, 17.6) |
| None recorded | N/K | 3.54 (3.02, 4.14) |
| *Lower gastrointestinal - Rectum (N = 1641)* | | |
| Non-specific | Bruising, bleeding or petechiae | 0.00 (0.00, 0.23) |
| Non-specific | Deep vein thrombosis | 0.12 (0.03, 0.44) |
| Non-specific | Epistaxis | 0.00 (0.00, 0.23) |
| Non-specific | Fatigue | 2.99 (2.27, 3.93) |
| Non-specific | Fever | 0.06 (0.01, 0.34) |
| Non-specific | Infection | 0.06 (0.01, 0.34) |
| Non-specific | Night sweats | 0.24 (0.09, 0.63) |
| Non-specific | Other symptom | 2.80 (2.11, 3.72) |
| Non-specific | Pallor | 0.37 (0.17, 0.80) |
| Non-specific | Pruritus | 0.18 (0.06, 0.54) |
| Non-specific | Weight loss | 10.8 (9.38, 12.4) |
| Lump/mass/lymph node | Anal mass | 1.40 (0.94, 2.09) |
| Lump/mass/lymph node | Lymph node pain with alcohol | 0.00 (0.00, 0.23) |
| Lump/mass/lymph node | Lymphadenopathy (generalised) | 0.00 (0.00, 0.23) |
| Lump/mass/lymph node | Lymphadenopathy (localised) | 0.06 (0.01, 0.34) |
| Lump/mass/lymph node | Neck lump/mass | 0.12 (0.03, 0.44) |
| Lump/mass/lymph node | Thyroid lump/mass | 0.00 (0.00, 0.23) |
| Lump/mass/lymph node | Unexplained lump suspicious of sarcoma | 0.00 (0.00, 0.23) |
| Ulceration | Leukoplakia | 0.00 (0.00, 0.23) |
| Ulceration | Lip/oral cavity/tongue lump/mass | 0.00 (0.00, 0.23) |
| Ulceration | Lip/oral cavity/tongue ulcer | 0.00 (0.00, 0.23) |
| Ulceration | Ulceration | 0.00 (0.00, 0.23) |
| Upper abdominal | Dyspepsia | 0.61 (0.33, 1.12) |
| Upper abdominal | Dysphagia | 0.18 (0.06, 0.54) |
| Upper abdominal | Early satiety | 0.00 (0.00, 0.23) |
| Upper abdominal | Gastroesophageal reflux | 0.30 (0.13, 0.71) |
| Upper abdominal | Haematemesis | 0.12 (0.03, 0.44) |
| Upper abdominal | Jaundice | 0.06 (0.01, 0.34) |
| Upper abdominal | Loss of appetite | 3.47 (2.69, 4.47) |
| Upper abdominal | Nausea and/or vomiting | 1.71 (1.18, 2.46) |
| Upper abdominal | New onset diabetes | 0.00 (0.00, 0.23) |
| Upper abdominal | Upper abdominal pain | 1.28 (0.84, 1.95) |
| Lower abdominal | Abdominal pain (NOS) | 5.55 (4.54, 6.76) |
| Lower abdominal | Change in bowel habit | 32.7 (30.4, 35.0) |
| Lower abdominal | Constipation | 7.92 (6.71, 9.33) |
| Lower abdominal | Diarrhoea | 15.4 (13.8, 17.2) |
| Lower abdominal | Distension | 3.05 (2.32, 3.99) |
| Lower abdominal | Lower abdominal pain | 4.94 (3.99, 6.09) |
| Lower abdominal | Pelvic pain | 0.85 (0.51, 1.43) |
| Lower abdominal | Rectal bleeding | 51.2 (48.8, 53.7) |
| Respiratory | Chest infection | 0.18 (0.06, 0.54) |
| Respiratory | Chest pain | 0.24 (0.09, 0.63) |
| Respiratory | Clubbing | 0.00 (0.00, 0.23) |
| Respiratory | Cough | 0.55 (0.29, 1.04) |
| Respiratory | Dyspnoea | 0.61 (0.33, 1.12) |
| Respiratory | Haemoptysis | 0.06 (0.01, 0.34) |
| Respiratory | Hoarseness | 0.00 (0.00, 0.23) |
| Respiratory | Sore throat | 0.00 (0.00, 0.23) |
| Respiratory | Stridor | 0.00 (0.00, 0.23) |
| Urological | Dysuria | 0.06 (0.01, 0.34) |
| Urological | Haematuria | 0.18 (0.06, 0.54) |
| Urological | Loin pain | 0.06 (0.01, 0.34) |
| Urological | LUTS (nocturia, frequency, hesitancy, urgency, retention) | 1.04 (0.65, 1.65) |
| Urological | Renal colic | 0.00 (0.00, 0.23) |
| Urological | Urinary tract infection | 0.12 (0.03, 0.44) |
| Central nervous system | Fit/seizure | 0.00 (0.00, 0.23) |
| Central nervous system | Headache | 0.06 (0.01, 0.34) |
| Central nervous system | Prog/sub-acute loss of central neuro funct | 0.18 (0.06, 0.54) |
| Central nervous system | Visual disturbance or loss | 0.00 (0.00, 0.23) |
| Musculoskeletal | Back pain | 0.98 (0.60, 1.58) |
| Musculoskeletal | Bone pain | 0.37 (0.17, 0.80) |
| Musculoskeletal | Fracture | 0.00 (0.00, 0.23) |
| Skin Lesion | Abnormal mole | 0.00 (0.00, 0.23) |
| Skin Lesion | Lesions suspicious of BCC | 0.00 (0.00, 0.23) |
| Skin Lesion | Non-pigmented lesion | 0.00 (0.00, 0.23) |
| Breast Symptoms | Axillary lump/mass | 0.00 (0.00, 0.23) |
| Breast Symptoms | Breast lump/mass | 0.00 (0.00, 0.23) |
| Breast Symptoms | Breast pain | 0.00 (0.00, 0.23) |
| Breast Symptoms | Nipple changes | 0.00 (0.00, 0.23) |
| Breast Symptoms | Nipple discharge | 0.00 (0.00, 0.23) |
| Female specific | Other vaginal bleeding | 0.06 (0.01, 0.34) |
| Female specific | Post-menopausal bleeding | 0.18 (0.06, 0.54) |
| Female specific | Vaginal discharge | 0.00 (0.00, 0.23) |
| Female specific | Vaginal mass | 0.00 (0.00, 0.23) |
| Female specific | Vulval bleeding | 0.06 (0.01, 0.34) |
| Female specific | Vulval mass | 0.00 (0.00, 0.23) |
| Female specific | Vulval ulceration | 0.00 (0.00, 0.23) |
| Male specific | Erectile dysfunction | 0.00 (0.00, 0.23) |
| Male specific | Penile ulceration | 0.00 (0.00, 0.23) |
| Male specific | Testicular lump | 0.00 (0.00, 0.23) |
| Male specific | Testicular pain | 0.12 (0.03, 0.44) |
| None recorded | N/A | 8.10 (6.88, 9.53) |
| None recorded | N/K | 3.05 (2.32, 3.99) |
| *Lower gastrointestinal - Small intestine (N = 256)* | | |
| Non-specific | Bruising, bleeding or petechiae | 0.00 (0.00, 1.48) |
| Non-specific | Deep vein thrombosis | 0.00 (0.00, 1.48) |
| Non-specific | Epistaxis | 0.00 (0.00, 1.48) |
| Non-specific | Fatigue | 6.25 (3.88, 9.91) |
| Non-specific | Fever | 2.73 (1.33, 5.54) |
| Non-specific | Infection | 0.00 (0.00, 1.48) |
| Non-specific | Night sweats | 0.39 (0.07, 2.18) |
| Non-specific | Other symptom | 3.12 (1.59, 6.04) |
| Non-specific | Pallor | 0.78 (0.21, 2.80) |
| Non-specific | Pruritus | 1.17 (0.40, 3.39) |
| Non-specific | Weight loss | 14.1 (10.3, 18.9) |
| Lump/mass/lymph node | Anal mass | 0.00 (0.00, 1.48) |
| Lump/mass/lymph node | Lymph node pain with alcohol | 0.00 (0.00, 1.48) |
| Lump/mass/lymph node | Lymphadenopathy (generalised) | 0.00 (0.00, 1.48) |
| Lump/mass/lymph node | Lymphadenopathy (localised) | 0.00 (0.00, 1.48) |
| Lump/mass/lymph node | Neck lump/mass | 0.39 (0.07, 2.18) |
| Lump/mass/lymph node | Thyroid lump/mass | 0.00 (0.00, 1.48) |
| Lump/mass/lymph node | Unexplained lump suspicious of sarcoma | 0.00 (0.00, 1.48) |
| Ulceration | Leukoplakia | 0.00 (0.00, 1.48) |
| Ulceration | Lip/oral cavity/tongue lump/mass | 0.00 (0.00, 1.48) |
| Ulceration | Lip/oral cavity/tongue ulcer | 0.00 (0.00, 1.48) |
| Ulceration | Ulceration | 0.00 (0.00, 1.48) |
| Upper abdominal | Dyspepsia | 7.81 (5.11, 11.8) |
| Upper abdominal | Dysphagia | 1.56 (0.61, 3.95) |
| Upper abdominal | Early satiety | 0.78 (0.21, 2.80) |
| Upper abdominal | Gastroesophageal reflux | 4.69 (2.70, 8.01) |
| Upper abdominal | Haematemesis | 1.95 (0.84, 4.49) |
| Upper abdominal | Jaundice | 3.52 (1.86, 6.55) |
| Upper abdominal | Loss of appetite | 5.86 (3.58, 9.44) |
| Upper abdominal | Nausea and/or vomiting | 16.4 (12.4, 21.4) |
| Upper abdominal | New onset diabetes | 0.00 (0.00, 1.48) |
| Upper abdominal | Upper abdominal pain | 11.3 (8.00, 15.8) |
| Lower abdominal | Abdominal pain (NOS) | 18.8 (14.4, 24.0) |
| Lower abdominal | Change in bowel habit | 9.38 (6.38, 13.6) |
| Lower abdominal | Constipation | 5.47 (3.29, 8.97) |
| Lower abdominal | Diarrhoea | 13.3 (9.66, 18.0) |
| Lower abdominal | Distension | 5.47 (3.29, 8.97) |
| Lower abdominal | Lower abdominal pain | 6.25 (3.88, 9.91) |
| Lower abdominal | Pelvic pain | 0.39 (0.07, 2.18) |
| Lower abdominal | Rectal bleeding | 3.91 (2.14, 7.04) |
| Respiratory | Chest infection | 0.00 (0.00, 1.48) |
| Respiratory | Chest pain | 0.00 (0.00, 1.48) |
| Respiratory | Clubbing | 0.39 (0.07, 2.18) |
| Respiratory | Cough | 0.78 (0.21, 2.80) |
| Respiratory | Dyspnoea | 1.95 (0.84, 4.49) |
| Respiratory | Haemoptysis | 0.00 (0.00, 1.48) |
| Respiratory | Hoarseness | 0.00 (0.00, 1.48) |
| Respiratory | Sore throat | 0.00 (0.00, 1.48) |
| Respiratory | Stridor | 0.00 (0.00, 1.48) |
| Urological | Dysuria | 0.00 (0.00, 1.48) |
| Urological | Haematuria | 0.78 (0.21, 2.80) |
| Urological | Loin pain | 0.78 (0.21, 2.80) |
| Urological | LUTS (nocturia, frequency, hesitancy, urgency, retention) | 0.78 (0.21, 2.80) |
| Urological | Renal colic | 0.39 (0.07, 2.18) |
| Urological | Urinary tract infection | 0.00 (0.00, 1.48) |
| Central nervous system | Fit/seizure | 0.00 (0.00, 1.48) |
| Central nervous system | Headache | 0.39 (0.07, 2.18) |
| Central nervous system | Prog/sub-acute loss of central neuro funct | 0.39 (0.07, 2.18) |
| Central nervous system | Visual disturbance or loss | 0.00 (0.00, 1.48) |
| Musculoskeletal | Back pain | 0.78 (0.21, 2.80) |
| Musculoskeletal | Bone pain | 0.00 (0.00, 1.48) |
| Musculoskeletal | Fracture | 0.00 (0.00, 1.48) |
| Skin Lesion | Abnormal mole | 0.00 (0.00, 1.48) |
| Skin Lesion | Lesions suspicious of BCC | 0.00 (0.00, 1.48) |
| Skin Lesion | Non-pigmented lesion | 0.00 (0.00, 1.48) |
| Breast Symptoms | Axillary lump/mass | 0.39 (0.07, 2.18) |
| Breast Symptoms | Breast lump/mass | 0.00 (0.00, 1.48) |
| Breast Symptoms | Breast pain | 0.00 (0.00, 1.48) |
| Breast Symptoms | Nipple changes | 0.00 (0.00, 1.48) |
| Breast Symptoms | Nipple discharge | 0.00 (0.00, 1.48) |
| Female specific | Other vaginal bleeding | 0.00 (0.00, 1.48) |
| Female specific | Post-menopausal bleeding | 0.39 (0.07, 2.18) |
| Female specific | Vaginal discharge | 0.00 (0.00, 1.48) |
| Female specific | Vaginal mass | 0.39 (0.07, 2.18) |
| Female specific | Vulval bleeding | 0.00 (0.00, 1.48) |
| Female specific | Vulval mass | 0.00 (0.00, 1.48) |
| Female specific | Vulval ulceration | 0.00 (0.00, 1.48) |
| Male specific | Erectile dysfunction | 0.00 (0.00, 1.48) |
| Male specific | Penile ulceration | 0.00 (0.00, 1.48) |
| Male specific | Testicular lump | 0.00 (0.00, 1.48) |
| Male specific | Testicular pain | 0.00 (0.00, 1.48) |
| None recorded | N/A | 18.4 (14.1, 23.6) |
| None recorded | N/K | 3.91 (2.14, 7.04) |
| *Hepato-pancreato-biliary - Liver (N = 929)* | | |
| Non-specific | Bruising, bleeding or petechiae | 0.32 (0.11, 0.95) |
| Non-specific | Deep vein thrombosis | 0.32 (0.11, 0.95) |
| Non-specific | Epistaxis | 0.32 (0.11, 0.95) |
| Non-specific | Fatigue | 7.97 (6.39, 9.88) |
| Non-specific | Fever | 0.65 (0.30, 1.40) |
| Non-specific | Infection | 0.43 (0.17, 1.10) |
| Non-specific | Night sweats | 0.32 (0.11, 0.95) |
| Non-specific | Other symptom | 6.67 (5.24, 8.46) |
| Non-specific | Pallor | 0.65 (0.30, 1.40) |
| Non-specific | Pruritus | 1.94 (1.23, 3.04) |
| Non-specific | Weight loss | 12.9 (10.9, 15.2) |
| Lump/mass/lymph node | Anal mass | 0.00 (0.00, 0.41) |
| Lump/mass/lymph node | Lymph node pain with alcohol | 0.00 (0.00, 0.41) |
| Lump/mass/lymph node | Lymphadenopathy (generalised) | 0.11 (0.02, 0.61) |
| Lump/mass/lymph node | Lymphadenopathy (localised) | 0.00 (0.00, 0.41) |
| Lump/mass/lymph node | Neck lump/mass | 0.32 (0.11, 0.95) |
| Lump/mass/lymph node | Thyroid lump/mass | 0.00 (0.00, 0.41) |
| Lump/mass/lymph node | Unexplained lump suspicious of sarcoma | 0.11 (0.02, 0.61) |
| Ulceration | Leukoplakia | 0.00 (0.00, 0.41) |
| Ulceration | Lip/oral cavity/tongue lump/mass | 0.00 (0.00, 0.41) |
| Ulceration | Lip/oral cavity/tongue ulcer | 0.00 (0.00, 0.41) |
| Ulceration | Ulceration | 0.00 (0.00, 0.41) |
| Upper abdominal | Dyspepsia | 3.01 (2.09, 4.32) |
| Upper abdominal | Dysphagia | 0.97 (0.51, 1.83) |
| Upper abdominal | Early satiety | 0.65 (0.30, 1.40) |
| Upper abdominal | Gastroesophageal reflux | 1.18 (0.66, 2.11) |
| Upper abdominal | Haematemesis | 0.86 (0.44, 1.69) |
| Upper abdominal | Jaundice | 10.7 (8.83, 12.8) |
| Upper abdominal | Loss of appetite | 7.97 (6.39, 9.88) |
| Upper abdominal | Nausea and/or vomiting | 6.89 (5.43, 8.70) |
| Upper abdominal | New onset diabetes | 0.00 (0.00, 0.41) |
| Upper abdominal | Upper abdominal pain | 10.1 (8.34, 12.2) |
| Lower abdominal | Abdominal pain (NOS) | 11.6 (9.72, 13.8) |
| Lower abdominal | Change in bowel habit | 3.34 (2.36, 4.70) |
| Lower abdominal | Constipation | 2.48 (1.66, 3.69) |
| Lower abdominal | Diarrhoea | 3.66 (2.63, 5.07) |
| Lower abdominal | Distension | 6.35 (4.96, 8.11) |
| Lower abdominal | Lower abdominal pain | 1.29 (0.74, 2.24) |
| Lower abdominal | Pelvic pain | 0.22 (0.06, 0.78) |
| Lower abdominal | Rectal bleeding | 1.51 (0.90, 2.51) |
| Respiratory | Chest infection | 0.43 (0.17, 1.10) |
| Respiratory | Chest pain | 1.51 (0.90, 2.51) |
| Respiratory | Clubbing | 0.00 (0.00, 0.41) |
| Respiratory | Cough | 1.08 (0.59, 1.97) |
| Respiratory | Dyspnoea | 2.91 (2.00, 4.20) |
| Respiratory | Haemoptysis | 0.75 (0.37, 1.55) |
| Respiratory | Hoarseness | 0.00 (0.00, 0.41) |
| Respiratory | Sore throat | 0.32 (0.11, 0.95) |
| Respiratory | Stridor | 0.00 (0.00, 0.41) |
| Urological | Dysuria | 0.32 (0.11, 0.95) |
| Urological | Haematuria | 0.97 (0.51, 1.83) |
| Urological | Loin pain | 0.32 (0.11, 0.95) |
| Urological | LUTS (nocturia, frequency, hesitancy, urgency, retention) | 0.86 (0.44, 1.69) |
| Urological | Renal colic | 0.00 (0.00, 0.41) |
| Urological | Urinary tract infection | 0.65 (0.30, 1.40) |
| Central nervous system | Fit/seizure | 0.00 (0.00, 0.41) |
| Central nervous system | Headache | 0.32 (0.11, 0.95) |
| Central nervous system | Prog/sub-acute loss of central neuro funct | 0.32 (0.11, 0.95) |
| Central nervous system | Visual disturbance or loss | 0.00 (0.00, 0.41) |
| Musculoskeletal | Back pain | 1.29 (0.74, 2.24) |
| Musculoskeletal | Bone pain | 0.22 (0.06, 0.78) |
| Musculoskeletal | Fracture | 0.11 (0.02, 0.61) |
| Skin Lesion | Abnormal mole | 0.00 (0.00, 0.41) |
| Skin Lesion | Lesions suspicious of BCC | 0.00 (0.00, 0.41) |
| Skin Lesion | Non-pigmented lesion | 0.00 (0.00, 0.41) |
| Breast Symptoms | Axillary lump/mass | 0.00 (0.00, 0.41) |
| Breast Symptoms | Breast lump/mass | 0.00 (0.00, 0.41) |
| Breast Symptoms | Breast pain | 0.00 (0.00, 0.41) |
| Breast Symptoms | Nipple changes | 0.00 (0.00, 0.41) |
| Breast Symptoms | Nipple discharge | 0.00 (0.00, 0.41) |
| Female specific | Other vaginal bleeding | 0.00 (0.00, 0.41) |
| Female specific | Post-menopausal bleeding | 0.11 (0.02, 0.61) |
| Female specific | Vaginal discharge | 0.00 (0.00, 0.41) |
| Female specific | Vaginal mass | 0.00 (0.00, 0.41) |
| Female specific | Vulval bleeding | 0.00 (0.00, 0.41) |
| Female specific | Vulval mass | 0.00 (0.00, 0.41) |
| Female specific | Vulval ulceration | 0.00 (0.00, 0.41) |
| Male specific | Erectile dysfunction | 0.00 (0.00, 0.41) |
| Male specific | Penile ulceration | 0.00 (0.00, 0.41) |
| Male specific | Testicular lump | 0.00 (0.00, 0.41) |
| Male specific | Testicular pain | 0.11 (0.02, 0.61) |
| None recorded | N/A | 29.0 (26.1, 32.0) |
| None recorded | N/K | 6.67 (5.24, 8.46) |
| *Hepato-pancreato-biliary - Pancreas (N = 1725)* | | |
| Non-specific | Bruising, bleeding or petechiae | 0.06 (0.01, 0.33) |
| Non-specific | Deep vein thrombosis | 0.70 (0.40, 1.21) |
| Non-specific | Epistaxis | 0.17 (0.06, 0.51) |
| Non-specific | Fatigue | 8.06 (6.86, 9.44) |
| Non-specific | Fever | 0.93 (0.57, 1.50) |
| Non-specific | Infection | 0.35 (0.16, 0.76) |
| Non-specific | Night sweats | 0.64 (0.36, 1.14) |
| Non-specific | Other symptom | 6.67 (5.58, 7.94) |
| Non-specific | Pallor | 0.35 (0.16, 0.76) |
| Non-specific | Pruritus | 2.43 (1.81, 3.27) |
| Non-specific | Weight loss | 22.1 (20.2, 24.2) |
| Lump/mass/lymph node | Anal mass | 0.00 (0.00, 0.22) |
| Lump/mass/lymph node | Lymph node pain with alcohol | 0.00 (0.00, 0.22) |
| Lump/mass/lymph node | Lymphadenopathy (generalised) | 0.06 (0.01, 0.33) |
| Lump/mass/lymph node | Lymphadenopathy (localised) | 0.12 (0.03, 0.42) |
| Lump/mass/lymph node | Neck lump/mass | 0.00 (0.00, 0.22) |
| Lump/mass/lymph node | Thyroid lump/mass | 0.00 (0.00, 0.22) |
| Lump/mass/lymph node | Unexplained lump suspicious of sarcoma | 0.00 (0.00, 0.22) |
| Ulceration | Leukoplakia | 0.00 (0.00, 0.22) |
| Ulceration | Lip/oral cavity/tongue lump/mass | 0.00 (0.00, 0.22) |
| Ulceration | Lip/oral cavity/tongue ulcer | 0.00 (0.00, 0.22) |
| Ulceration | Ulceration | 0.06 (0.01, 0.33) |
| Upper abdominal | Dyspepsia | 6.03 (5.00, 7.25) |
| Upper abdominal | Dysphagia | 1.86 (1.32, 2.61) |
| Upper abdominal | Early satiety | 1.33 (0.89, 1.99) |
| Upper abdominal | Gastroesophageal reflux | 1.51 (1.03, 2.20) |
| Upper abdominal | Haematemesis | 0.58 (0.32, 1.06) |
| Upper abdominal | Jaundice | 13.9 (12.3, 15.6) |
| Upper abdominal | Loss of appetite | 11.8 (10.3, 13.4) |
| Upper abdominal | Nausea and/or vomiting | 11.1 (9.73, 12.7) |
| Upper abdominal | New onset diabetes | 0.93 (0.57, 1.50) |
| Upper abdominal | Upper abdominal pain | 18.7 (17.0, 20.6) |
| Lower abdominal | Abdominal pain (NOS) | 19.5 (17.7, 21.5) |
| Lower abdominal | Change in bowel habit | 7.30 (6.17, 8.63) |
| Lower abdominal | Constipation | 5.45 (4.47, 6.62) |
| Lower abdominal | Diarrhoea | 6.32 (5.26, 7.57) |
| Lower abdominal | Distension | 5.97 (4.95, 7.19) |
| Lower abdominal | Lower abdominal pain | 3.59 (2.81, 4.58) |
| Lower abdominal | Pelvic pain | 0.35 (0.16, 0.76) |
| Lower abdominal | Rectal bleeding | 1.22 (0.80, 1.85) |
| Respiratory | Chest infection | 0.46 (0.24, 0.91) |
| Respiratory | Chest pain | 2.26 (1.66, 3.08) |
| Respiratory | Clubbing | 0.00 (0.00, 0.22) |
| Respiratory | Cough | 1.16 (0.75, 1.78) |
| Respiratory | Dyspnoea | 1.39 (0.94, 2.06) |
| Respiratory | Haemoptysis | 0.06 (0.01, 0.33) |
| Respiratory | Hoarseness | 0.17 (0.06, 0.51) |
| Respiratory | Sore throat | 0.00 (0.00, 0.22) |
| Respiratory | Stridor | 0.00 (0.00, 0.22) |
| Urological | Dysuria | 0.35 (0.16, 0.76) |
| Urological | Haematuria | 0.52 (0.27, 0.99) |
| Urological | Loin pain | 0.23 (0.09, 0.59) |
| Urological | LUTS (nocturia, frequency, hesitancy, urgency, retention) | 0.87 (0.53, 1.43) |
| Urological | Renal colic | 0.06 (0.01, 0.33) |
| Urological | Urinary tract infection | 0.64 (0.36, 1.14) |
| Central nervous system | Fit/seizure | 0.00 (0.00, 0.22) |
| Central nervous system | Headache | 0.29 (0.12, 0.68) |
| Central nervous system | Prog/sub-acute loss of central neuro funct | 0.35 (0.16, 0.76) |
| Central nervous system | Visual disturbance or loss | 0.00 (0.00, 0.22) |
| Musculoskeletal | Back pain | 4.99 (4.05, 6.12) |
| Musculoskeletal | Bone pain | 0.29 (0.12, 0.68) |
| Musculoskeletal | Fracture | 0.06 (0.01, 0.33) |
| Skin Lesion | Abnormal mole | 0.00 (0.00, 0.22) |
| Skin Lesion | Lesions suspicious of BCC | 0.00 (0.00, 0.22) |
| Skin Lesion | Non-pigmented lesion | 0.00 (0.00, 0.22) |
| Breast Symptoms | Axillary lump/mass | 0.00 (0.00, 0.22) |
| Breast Symptoms | Breast lump/mass | 0.06 (0.01, 0.33) |
| Breast Symptoms | Breast pain | 0.06 (0.01, 0.33) |
| Breast Symptoms | Nipple changes | 0.00 (0.00, 0.22) |
| Breast Symptoms | Nipple discharge | 0.00 (0.00, 0.22) |
| Female specific | Other vaginal bleeding | 0.00 (0.00, 0.22) |
| Female specific | Post-menopausal bleeding | 0.06 (0.01, 0.33) |
| Female specific | Vaginal discharge | 0.06 (0.01, 0.33) |
| Female specific | Vaginal mass | 0.06 (0.01, 0.33) |
| Female specific | Vulval bleeding | 0.00 (0.00, 0.22) |
| Female specific | Vulval mass | 0.06 (0.01, 0.33) |
| Female specific | Vulval ulceration | 0.00 (0.00, 0.22) |
| Male specific | Erectile dysfunction | 0.00 (0.00, 0.22) |
| Male specific | Penile ulceration | 0.00 (0.00, 0.22) |
| Male specific | Testicular lump | 0.06 (0.01, 0.33) |
| Male specific | Testicular pain | 0.00 (0.00, 0.22) |
| None recorded | N/A | 10.6 (9.19, 12.1) |
| None recorded | N/K | 4.64 (3.74, 5.73) |
| *Hepato-pancreato-biliary - Other Hepato-pancreato-biliary (N = 425)* | | |
| Non-specific | Bruising, bleeding or petechiae | 0.24 (0.04, 1.32) |
| Non-specific | Deep vein thrombosis | 0.71 (0.24, 2.05) |
| Non-specific | Epistaxis | 0.00 (0.00, 0.90) |
| Non-specific | Fatigue | 5.41 (3.63, 7.99) |
| Non-specific | Fever | 0.94 (0.37, 2.39) |
| Non-specific | Infection | 0.00 (0.00, 0.90) |
| Non-specific | Night sweats | 0.47 (0.13, 1.70) |
| Non-specific | Other symptom | 6.35 (4.40, 9.09) |
| Non-specific | Pallor | 0.47 (0.13, 1.70) |
| Non-specific | Pruritus | 5.65 (3.82, 8.26) |
| Non-specific | Weight loss | 12.0 (9.25, 15.4) |
| Lump/mass/lymph node | Anal mass | 0.00 (0.00, 0.90) |
| Lump/mass/lymph node | Lymph node pain with alcohol | 0.00 (0.00, 0.90) |
| Lump/mass/lymph node | Lymphadenopathy (generalised) | 0.00 (0.00, 0.90) |
| Lump/mass/lymph node | Lymphadenopathy (localised) | 0.00 (0.00, 0.90) |
| Lump/mass/lymph node | Neck lump/mass | 0.00 (0.00, 0.90) |
| Lump/mass/lymph node | Thyroid lump/mass | 0.00 (0.00, 0.90) |
| Lump/mass/lymph node | Unexplained lump suspicious of sarcoma | 0.00 (0.00, 0.90) |
| Ulceration | Leukoplakia | 0.00 (0.00, 0.90) |
| Ulceration | Lip/oral cavity/tongue lump/mass | 0.00 (0.00, 0.90) |
| Ulceration | Lip/oral cavity/tongue ulcer | 0.00 (0.00, 0.90) |
| Ulceration | Ulceration | 0.00 (0.00, 0.90) |
| Upper abdominal | Dyspepsia | 5.65 (3.82, 8.26) |
| Upper abdominal | Dysphagia | 0.71 (0.24, 2.05) |
| Upper abdominal | Early satiety | 0.00 (0.00, 0.90) |
| Upper abdominal | Gastroesophageal reflux | 0.24 (0.04, 1.32) |
| Upper abdominal | Haematemesis | 1.18 (0.50, 2.72) |
| Upper abdominal | Jaundice | 23.3 (19.5, 27.5) |
| Upper abdominal | Loss of appetite | 8.47 (6.18, 11.5) |
| Upper abdominal | Nausea and/or vomiting | 11.8 (9.04, 15.2) |
| Upper abdominal | New onset diabetes | 0.00 (0.00, 0.90) |
| Upper abdominal | Upper abdominal pain | 17.4 (14.1, 21.3) |
| Lower abdominal | Abdominal pain (NOS) | 15.8 (12.6, 19.5) |
| Lower abdominal | Change in bowel habit | 2.59 (1.45, 4.57) |
| Lower abdominal | Constipation | 2.59 (1.45, 4.57) |
| Lower abdominal | Diarrhoea | 6.35 (4.40, 9.09) |
| Lower abdominal | Distension | 4.47 (2.88, 6.88) |
| Lower abdominal | Lower abdominal pain | 3.76 (2.33, 6.03) |
| Lower abdominal | Pelvic pain | 0.00 (0.00, 0.90) |
| Lower abdominal | Rectal bleeding | 1.18 (0.50, 2.72) |
| Respiratory | Chest infection | 0.00 (0.00, 0.90) |
| Respiratory | Chest pain | 2.12 (1.12, 3.98) |
| Respiratory | Clubbing | 0.00 (0.00, 0.90) |
| Respiratory | Cough | 0.94 (0.37, 2.39) |
| Respiratory | Dyspnoea | 1.41 (0.65, 3.05) |
| Respiratory | Haemoptysis | 0.00 (0.00, 0.90) |
| Respiratory | Hoarseness | 0.00 (0.00, 0.90) |
| Respiratory | Sore throat | 0.00 (0.00, 0.90) |
| Respiratory | Stridor | 0.00 (0.00, 0.90) |
| Urological | Dysuria | 0.24 (0.04, 1.32) |
| Urological | Haematuria | 0.71 (0.24, 2.05) |
| Urological | Loin pain | 0.00 (0.00, 0.90) |
| Urological | LUTS (nocturia, frequency, hesitancy, urgency, retention) | 0.24 (0.04, 1.32) |
| Urological | Renal colic | 0.00 (0.00, 0.90) |
| Urological | Urinary tract infection | 0.47 (0.13, 1.70) |
| Central nervous system | Fit/seizure | 0.24 (0.04, 1.32) |
| Central nervous system | Headache | 0.71 (0.24, 2.05) |
| Central nervous system | Prog/sub-acute loss of central neuro funct | 0.47 (0.13, 1.70) |
| Central nervous system | Visual disturbance or loss | 0.00 (0.00, 0.90) |
| Musculoskeletal | Back pain | 1.41 (0.65, 3.05) |
| Musculoskeletal | Bone pain | 0.47 (0.13, 1.70) |
| Musculoskeletal | Fracture | 0.00 (0.00, 0.90) |
| Skin Lesion | Abnormal mole | 0.00 (0.00, 0.90) |
| Skin Lesion | Lesions suspicious of BCC | 0.00 (0.00, 0.90) |
| Skin Lesion | Non-pigmented lesion | 0.00 (0.00, 0.90) |
| Breast Symptoms | Axillary lump/mass | 0.00 (0.00, 0.90) |
| Breast Symptoms | Breast lump/mass | 0.24 (0.04, 1.32) |
| Breast Symptoms | Breast pain | 0.00 (0.00, 0.90) |
| Breast Symptoms | Nipple changes | 0.00 (0.00, 0.90) |
| Breast Symptoms | Nipple discharge | 0.00 (0.00, 0.90) |
| Female specific | Other vaginal bleeding | 0.00 (0.00, 0.90) |
| Female specific | Post-menopausal bleeding | 0.00 (0.00, 0.90) |
| Female specific | Vaginal discharge | 0.24 (0.04, 1.32) |
| Female specific | Vaginal mass | 0.00 (0.00, 0.90) |
| Female specific | Vulval bleeding | 0.00 (0.00, 0.90) |
| Female specific | Vulval mass | 0.00 (0.00, 0.90) |
| Female specific | Vulval ulceration | 0.00 (0.00, 0.90) |
| Male specific | Erectile dysfunction | 0.00 (0.00, 0.90) |
| Male specific | Penile ulceration | 0.00 (0.00, 0.90) |
| Male specific | Testicular lump | 0.00 (0.00, 0.90) |
| Male specific | Testicular pain | 0.00 (0.00, 0.90) |
| None recorded | N/A | 14.8 (11.8, 18.5) |
| None recorded | N/K | 3.29 (1.97, 5.45) |
| *Respiratory - Lung (N = 7608)* | | |
| Non-specific | Bruising, bleeding or petechiae | 0.11 (0.05, 0.21) |
| Non-specific | Deep vein thrombosis | 0.20 (0.12, 0.33) |
| Non-specific | Epistaxis | 0.08 (0.04, 0.17) |
| Non-specific | Fatigue | 5.60 (5.10, 6.14) |
| Non-specific | Fever | 0.74 (0.57, 0.95) |
| Non-specific | Infection | 0.55 (0.41, 0.75) |
| Non-specific | Night sweats | 0.70 (0.53, 0.91) |
| Non-specific | Other symptom | 6.05 (5.53, 6.60) |
| Non-specific | Pallor | 0.16 (0.09, 0.28) |
| Non-specific | Pruritus | 0.17 (0.10, 0.29) |
| Non-specific | Weight loss | 11.5 (10.8, 12.2) |
| Lump/mass/lymph node | Anal mass | 0.01 (0.00, 0.07) |
| Lump/mass/lymph node | Lymph node pain with alcohol | 0.00 (0.00, 0.05) |
| Lump/mass/lymph node | Lymphadenopathy (generalised) | 0.01 (0.00, 0.07) |
| Lump/mass/lymph node | Lymphadenopathy (localised) | 0.21 (0.13, 0.34) |
| Lump/mass/lymph node | Neck lump/mass | 0.87 (0.68, 1.10) |
| Lump/mass/lymph node | Thyroid lump/mass | 0.01 (0.00, 0.07) |
| Lump/mass/lymph node | Unexplained lump suspicious of sarcoma | 0.07 (0.03, 0.15) |
| Ulceration | Leukoplakia | 0.01 (0.00, 0.07) |
| Ulceration | Lip/oral cavity/tongue lump/mass | 0.05 (0.02, 0.14) |
| Ulceration | Lip/oral cavity/tongue ulcer | 0.05 (0.02, 0.14) |
| Ulceration | Ulceration | 0.03 (0.01, 0.10) |
| Upper abdominal | Dyspepsia | 0.64 (0.49, 0.85) |
| Upper abdominal | Dysphagia | 0.97 (0.78, 1.22) |
| Upper abdominal | Early satiety | 0.20 (0.12, 0.33) |
| Upper abdominal | Gastroesophageal reflux | 0.35 (0.24, 0.52) |
| Upper abdominal | Haematemesis | 0.12 (0.06, 0.22) |
| Upper abdominal | Jaundice | 0.16 (0.09, 0.28) |
| Upper abdominal | Loss of appetite | 4.26 (3.83, 4.74) |
| Upper abdominal | Nausea and/or vomiting | 1.75 (1.48, 2.07) |
| Upper abdominal | New onset diabetes | 0.00 (0.00, 0.05) |
| Upper abdominal | Upper abdominal pain | 1.42 (1.18, 1.71) |
| Lower abdominal | Abdominal pain (NOS) | 1.96 (1.67, 2.29) |
| Lower abdominal | Change in bowel habit | 0.76 (0.59, 0.98) |
| Lower abdominal | Constipation | 0.67 (0.51, 0.88) |
| Lower abdominal | Diarrhoea | 0.85 (0.67, 1.09) |
| Lower abdominal | Distension | 0.51 (0.38, 0.70) |
| Lower abdominal | Lower abdominal pain | 0.47 (0.34, 0.65) |
| Lower abdominal | Pelvic pain | 0.35 (0.24, 0.52) |
| Lower abdominal | Rectal bleeding | 0.25 (0.16, 0.39) |
| Respiratory | Chest infection | 10.8 (10.1, 11.5) |
| Respiratory | Chest pain | 9.69 (9.04, 10.4) |
| Respiratory | Clubbing | 0.46 (0.33, 0.64) |
| Respiratory | Cough | 29.8 (28.8, 30.8) |
| Respiratory | Dyspnoea | 18.2 (17.3, 19.0) |
| Respiratory | Haemoptysis | 6.52 (5.99, 7.10) |
| Respiratory | Hoarseness | 1.71 (1.44, 2.03) |
| Respiratory | Sore throat | 0.68 (0.52, 0.90) |
| Respiratory | Stridor | 0.16 (0.09, 0.28) |
| Urological | Dysuria | 0.05 (0.02, 0.14) |
| Urological | Haematuria | 0.24 (0.15, 0.37) |
| Urological | Loin pain | 0.16 (0.09, 0.28) |
| Urological | LUTS (nocturia, frequency, hesitancy, urgency, retention) | 0.38 (0.27, 0.55) |
| Urological | Renal colic | 0.05 (0.02, 0.14) |
| Urological | Urinary tract infection | 0.12 (0.06, 0.22) |
| Central nervous system | Fit/seizure | 0.30 (0.20, 0.45) |
| Central nervous system | Headache | 1.13 (0.92, 1.39) |
| Central nervous system | Prog/sub-acute loss of central neuro funct | 1.38 (1.14, 1.67) |
| Central nervous system | Visual disturbance or loss | 0.29 (0.19, 0.44) |
| Musculoskeletal | Back pain | 4.63 (4.18, 5.12) |
| Musculoskeletal | Bone pain | 2.13 (1.83, 2.48) |
| Musculoskeletal | Fracture | 0.24 (0.15, 0.37) |
| Skin Lesion | Abnormal mole | 0.01 (0.00, 0.07) |
| Skin Lesion | Lesions suspicious of BCC | 0.01 (0.00, 0.07) |
| Skin Lesion | Non-pigmented lesion | 0.03 (0.01, 0.10) |
| Breast Symptoms | Axillary lump/mass | 0.12 (0.06, 0.22) |
| Breast Symptoms | Breast lump/mass | 0.22 (0.14, 0.36) |
| Breast Symptoms | Breast pain | 0.16 (0.09, 0.28) |
| Breast Symptoms | Nipple changes | 0.05 (0.02, 0.14) |
| Breast Symptoms | Nipple discharge | 0.00 (0.00, 0.05) |
| Female specific | Other vaginal bleeding | 0.00 (0.00, 0.05) |
| Female specific | Post-menopausal bleeding | 0.01 (0.00, 0.07) |
| Female specific | Vaginal discharge | 0.00 (0.00, 0.05) |
| Female specific | Vaginal mass | 0.00 (0.00, 0.05) |
| Female specific | Vulval bleeding | 0.01 (0.00, 0.07) |
| Female specific | Vulval mass | 0.00 (0.00, 0.05) |
| Female specific | Vulval ulceration | 0.00 (0.00, 0.05) |
| Male specific | Erectile dysfunction | 0.00 (0.00, 0.05) |
| Male specific | Penile ulceration | 0.00 (0.00, 0.05) |
| Male specific | Testicular lump | 0.01 (0.00, 0.07) |
| Male specific | Testicular pain | 0.00 (0.00, 0.05) |
| None recorded | N/A | 19.0 (18.1, 19.9) |
| None recorded | N/K | 4.61 (4.16, 5.11) |
| *Respiratory - Mesothelioma (N = 464)* | | |
| Non-specific | Bruising, bleeding or petechiae | 0.00 (0.00, 0.82) |
| Non-specific | Deep vein thrombosis | 0.00 (0.00, 0.82) |
| Non-specific | Epistaxis | 0.00 (0.00, 0.82) |
| Non-specific | Fatigue | 7.33 (5.29, 10.1) |
| Non-specific | Fever | 0.86 (0.34, 2.20) |
| Non-specific | Infection | 0.22 (0.04, 1.21) |
| Non-specific | Night sweats | 0.65 (0.22, 1.88) |
| Non-specific | Other symptom | 4.09 (2.64, 6.31) |
| Non-specific | Pallor | 0.22 (0.04, 1.21) |
| Non-specific | Pruritus | 0.00 (0.00, 0.82) |
| Non-specific | Weight loss | 10.8 (8.27, 13.9) |
| Lump/mass/lymph node | Anal mass | 0.00 (0.00, 0.82) |
| Lump/mass/lymph node | Lymph node pain with alcohol | 0.00 (0.00, 0.82) |
| Lump/mass/lymph node | Lymphadenopathy (generalised) | 0.00 (0.00, 0.82) |
| Lump/mass/lymph node | Lymphadenopathy (localised) | 0.22 (0.04, 1.21) |
| Lump/mass/lymph node | Neck lump/mass | 0.00 (0.00, 0.82) |
| Lump/mass/lymph node | Thyroid lump/mass | 0.00 (0.00, 0.82) |
| Lump/mass/lymph node | Unexplained lump suspicious of sarcoma | 0.00 (0.00, 0.82) |
| Ulceration | Leukoplakia | 0.00 (0.00, 0.82) |
| Ulceration | Lip/oral cavity/tongue lump/mass | 0.00 (0.00, 0.82) |
| Ulceration | Lip/oral cavity/tongue ulcer | 0.22 (0.04, 1.21) |
| Ulceration | Ulceration | 0.00 (0.00, 0.82) |
| Upper abdominal | Dyspepsia | 0.43 (0.12, 1.56) |
| Upper abdominal | Dysphagia | 0.00 (0.00, 0.82) |
| Upper abdominal | Early satiety | 0.22 (0.04, 1.21) |
| Upper abdominal | Gastroesophageal reflux | 0.22 (0.04, 1.21) |
| Upper abdominal | Haematemesis | 0.00 (0.00, 0.82) |
| Upper abdominal | Jaundice | 0.22 (0.04, 1.21) |
| Upper abdominal | Loss of appetite | 3.45 (2.13, 5.53) |
| Upper abdominal | Nausea and/or vomiting | 0.65 (0.22, 1.88) |
| Upper abdominal | New onset diabetes | 0.00 (0.00, 0.82) |
| Upper abdominal | Upper abdominal pain | 1.72 (0.88, 3.36) |
| Lower abdominal | Abdominal pain (NOS) | 1.72 (0.88, 3.36) |
| Lower abdominal | Change in bowel habit | 1.08 (0.46, 2.50) |
| Lower abdominal | Constipation | 0.86 (0.34, 2.20) |
| Lower abdominal | Diarrhoea | 1.29 (0.59, 2.79) |
| Lower abdominal | Distension | 1.29 (0.59, 2.79) |
| Lower abdominal | Lower abdominal pain | 0.65 (0.22, 1.88) |
| Lower abdominal | Pelvic pain | 0.00 (0.00, 0.82) |
| Lower abdominal | Rectal bleeding | 0.00 (0.00, 0.82) |
| Respiratory | Chest infection | 8.19 (6.02, 11.0) |
| Respiratory | Chest pain | 20.5 (17.1, 24.4) |
| Respiratory | Clubbing | 0.22 (0.04, 1.21) |
| Respiratory | Cough | 33.0 (28.9, 37.4) |
| Respiratory | Dyspnoea | 45.3 (40.8, 49.8) |
| Respiratory | Haemoptysis | 1.29 (0.59, 2.79) |
| Respiratory | Hoarseness | 0.22 (0.04, 1.21) |
| Respiratory | Sore throat | 0.22 (0.04, 1.21) |
| Respiratory | Stridor | 0.00 (0.00, 0.82) |
| Urological | Dysuria | 0.22 (0.04, 1.21) |
| Urological | Haematuria | 0.22 (0.04, 1.21) |
| Urological | Loin pain | 0.00 (0.00, 0.82) |
| Urological | LUTS (nocturia, frequency, hesitancy, urgency, retention) | 0.00 (0.00, 0.82) |
| Urological | Renal colic | 0.00 (0.00, 0.82) |
| Urological | Urinary tract infection | 0.00 (0.00, 0.82) |
| Central nervous system | Fit/seizure | 0.00 (0.00, 0.82) |
| Central nervous system | Headache | 0.00 (0.00, 0.82) |
| Central nervous system | Prog/sub-acute loss of central neuro funct | 0.22 (0.04, 1.21) |
| Central nervous system | Visual disturbance or loss | 0.00 (0.00, 0.82) |
| Musculoskeletal | Back pain | 2.16 (1.17, 3.92) |
| Musculoskeletal | Bone pain | 0.86 (0.34, 2.20) |
| Musculoskeletal | Fracture | 0.00 (0.00, 0.82) |
| Skin Lesion | Abnormal mole | 0.22 (0.04, 1.21) |
| Skin Lesion | Lesions suspicious of BCC | 0.22 (0.04, 1.21) |
| Skin Lesion | Non-pigmented lesion | 0.22 (0.04, 1.21) |
| Breast Symptoms | Axillary lump/mass | 0.00 (0.00, 0.82) |
| Breast Symptoms | Breast lump/mass | 0.22 (0.04, 1.21) |
| Breast Symptoms | Breast pain | 0.00 (0.00, 0.82) |
| Breast Symptoms | Nipple changes | 0.00 (0.00, 0.82) |
| Breast Symptoms | Nipple discharge | 0.00 (0.00, 0.82) |
| Female specific | Other vaginal bleeding | 0.43 (0.12, 1.56) |
| Female specific | Post-menopausal bleeding | 0.00 (0.00, 0.82) |
| Female specific | Vaginal discharge | 0.00 (0.00, 0.82) |
| Female specific | Vaginal mass | 0.00 (0.00, 0.82) |
| Female specific | Vulval bleeding | 0.00 (0.00, 0.82) |
| Female specific | Vulval mass | 0.00 (0.00, 0.82) |
| Female specific | Vulval ulceration | 0.00 (0.00, 0.82) |
| Male specific | Erectile dysfunction | 0.00 (0.00, 0.82) |
| Male specific | Penile ulceration | 0.00 (0.00, 0.82) |
| Male specific | Testicular lump | 0.00 (0.00, 0.82) |
| Male specific | Testicular pain | 0.00 (0.00, 0.82) |
| None recorded | N/A | 10.1 (7.70, 13.2) |
| None recorded | N/K | 2.37 (1.33, 4.19) |
| *Urological - Bladder (N = 1518)* | | |
| Non-specific | Bruising, bleeding or petechiae | 0.00 (0.00, 0.25) |
| Non-specific | Deep vein thrombosis | 0.13 (0.04, 0.48) |
| Non-specific | Epistaxis | 0.00 (0.00, 0.25) |
| Non-specific | Fatigue | 1.52 (1.01, 2.26) |
| Non-specific | Fever | 0.33 (0.14, 0.77) |
| Non-specific | Infection | 0.26 (0.10, 0.68) |
| Non-specific | Night sweats | 0.13 (0.04, 0.48) |
| Non-specific | Other symptom | 3.03 (2.28, 4.02) |
| Non-specific | Pallor | 0.07 (0.01, 0.37) |
| Non-specific | Pruritus | 0.00 (0.00, 0.25) |
| Non-specific | Weight loss | 2.57 (1.89, 3.49) |
| Lump/mass/lymph node | Anal mass | 0.00 (0.00, 0.25) |
| Lump/mass/lymph node | Lymph node pain with alcohol | 0.00 (0.00, 0.25) |
| Lump/mass/lymph node | Lymphadenopathy (generalised) | 0.00 (0.00, 0.25) |
| Lump/mass/lymph node | Lymphadenopathy (localised) | 0.13 (0.04, 0.48) |
| Lump/mass/lymph node | Neck lump/mass | 0.00 (0.00, 0.25) |
| Lump/mass/lymph node | Thyroid lump/mass | 0.00 (0.00, 0.25) |
| Lump/mass/lymph node | Unexplained lump suspicious of sarcoma | 0.00 (0.00, 0.25) |
| Ulceration | Leukoplakia | 0.00 (0.00, 0.25) |
| Ulceration | Lip/oral cavity/tongue lump/mass | 0.00 (0.00, 0.25) |
| Ulceration | Lip/oral cavity/tongue ulcer | 0.00 (0.00, 0.25) |
| Ulceration | Ulceration | 0.07 (0.01, 0.37) |
| Upper abdominal | Dyspepsia | 0.33 (0.14, 0.77) |
| Upper abdominal | Dysphagia | 0.00 (0.00, 0.25) |
| Upper abdominal | Early satiety | 0.00 (0.00, 0.25) |
| Upper abdominal | Gastroesophageal reflux | 0.13 (0.04, 0.48) |
| Upper abdominal | Haematemesis | 0.20 (0.07, 0.58) |
| Upper abdominal | Jaundice | 0.33 (0.14, 0.77) |
| Upper abdominal | Loss of appetite | 0.92 (0.55, 1.54) |
| Upper abdominal | Nausea and/or vomiting | 0.59 (0.31, 1.12) |
| Upper abdominal | New onset diabetes | 0.00 (0.00, 0.25) |
| Upper abdominal | Upper abdominal pain | 0.46 (0.22, 0.95) |
| Lower abdominal | Abdominal pain (NOS) | 1.58 (1.06, 2.34) |
| Lower abdominal | Change in bowel habit | 0.46 (0.22, 0.95) |
| Lower abdominal | Constipation | 0.79 (0.45, 1.38) |
| Lower abdominal | Diarrhoea | 0.59 (0.31, 1.12) |
| Lower abdominal | Distension | 0.46 (0.22, 0.95) |
| Lower abdominal | Lower abdominal pain | 2.90 (2.17, 3.87) |
| Lower abdominal | Pelvic pain | 1.12 (0.70, 1.79) |
| Lower abdominal | Rectal bleeding | 0.20 (0.07, 0.58) |
| Respiratory | Chest infection | 0.13 (0.04, 0.48) |
| Respiratory | Chest pain | 0.13 (0.04, 0.48) |
| Respiratory | Clubbing | 0.00 (0.00, 0.25) |
| Respiratory | Cough | 0.40 (0.18, 0.86) |
| Respiratory | Dyspnoea | 0.59 (0.31, 1.12) |
| Respiratory | Haemoptysis | 0.07 (0.01, 0.37) |
| Respiratory | Hoarseness | 0.00 (0.00, 0.25) |
| Respiratory | Sore throat | 0.00 (0.00, 0.25) |
| Respiratory | Stridor | 0.00 (0.00, 0.25) |
| Urological | Dysuria | 9.29 (7.93, 10.9) |
| Urological | Haematuria | 53.3 (50.8, 55.8) |
| Urological | Loin pain | 0.99 (0.60, 1.62) |
| Urological | LUTS (nocturia, frequency, hesitancy, urgency, retention) | 15.7 (13.9, 17.6) |
| Urological | Renal colic | 0.13 (0.04, 0.48) |
| Urological | Urinary tract infection | 11.0 (9.52, 12.7) |
| Central nervous system | Fit/seizure | 0.00 (0.00, 0.25) |
| Central nervous system | Headache | 0.07 (0.01, 0.37) |
| Central nervous system | Prog/sub-acute loss of central neuro funct | 0.07 (0.01, 0.37) |
| Central nervous system | Visual disturbance or loss | 0.00 (0.00, 0.25) |
| Musculoskeletal | Back pain | 1.52 (1.01, 2.26) |
| Musculoskeletal | Bone pain | 0.07 (0.01, 0.37) |
| Musculoskeletal | Fracture | 0.00 (0.00, 0.25) |
| Skin Lesion | Abnormal mole | 0.00 (0.00, 0.25) |
| Skin Lesion | Lesions suspicious of BCC | 0.00 (0.00, 0.25) |
| Skin Lesion | Non-pigmented lesion | 0.00 (0.00, 0.25) |
| Breast Symptoms | Axillary lump/mass | 0.00 (0.00, 0.25) |
| Breast Symptoms | Breast lump/mass | 0.07 (0.01, 0.37) |
| Breast Symptoms | Breast pain | 0.00 (0.00, 0.25) |
| Breast Symptoms | Nipple changes | 0.00 (0.00, 0.25) |
| Breast Symptoms | Nipple discharge | 0.00 (0.00, 0.25) |
| Female specific | Other vaginal bleeding | 0.33 (0.14, 0.77) |
| Female specific | Post-menopausal bleeding | 0.20 (0.07, 0.58) |
| Female specific | Vaginal discharge | 0.26 (0.10, 0.68) |
| Female specific | Vaginal mass | 0.00 (0.00, 0.25) |
| Female specific | Vulval bleeding | 0.00 (0.00, 0.25) |
| Female specific | Vulval mass | 0.00 (0.00, 0.25) |
| Female specific | Vulval ulceration | 0.00 (0.00, 0.25) |
| Male specific | Erectile dysfunction | 0.07 (0.01, 0.37) |
| Male specific | Penile ulceration | 0.00 (0.00, 0.25) |
| Male specific | Testicular lump | 0.00 (0.00, 0.25) |
| Male specific | Testicular pain | 0.33 (0.14, 0.77) |
| None recorded | N/A | 11.8 (10.3, 13.5) |
| None recorded | N/K | 3.56 (2.74, 4.61) |
| *Urological - Kidney (N = 1695)* | | |
| Non-specific | Bruising, bleeding or petechiae | 0.18 (0.06, 0.52) |
| Non-specific | Deep vein thrombosis | 0.18 (0.06, 0.52) |
| Non-specific | Epistaxis | 0.06 (0.01, 0.33) |
| Non-specific | Fatigue | 3.66 (2.86, 4.66) |
| Non-specific | Fever | 0.53 (0.28, 1.01) |
| Non-specific | Infection | 0.06 (0.01, 0.33) |
| Non-specific | Night sweats | 1.06 (0.67, 1.67) |
| Non-specific | Other symptom | 4.60 (3.70, 5.71) |
| Non-specific | Pallor | 0.06 (0.01, 0.33) |
| Non-specific | Pruritus | 0.00 (0.00, 0.23) |
| Non-specific | Weight loss | 7.37 (6.22, 8.72) |
| Lump/mass/lymph node | Anal mass | 0.00 (0.00, 0.23) |
| Lump/mass/lymph node | Lymph node pain with alcohol | 0.00 (0.00, 0.23) |
| Lump/mass/lymph node | Lymphadenopathy (generalised) | 0.06 (0.01, 0.33) |
| Lump/mass/lymph node | Lymphadenopathy (localised) | 0.06 (0.01, 0.33) |
| Lump/mass/lymph node | Neck lump/mass | 0.53 (0.28, 1.01) |
| Lump/mass/lymph node | Thyroid lump/mass | 0.00 (0.00, 0.23) |
| Lump/mass/lymph node | Unexplained lump suspicious of sarcoma | 0.12 (0.03, 0.43) |
| Ulceration | Leukoplakia | 0.00 (0.00, 0.23) |
| Ulceration | Lip/oral cavity/tongue lump/mass | 0.00 (0.00, 0.23) |
| Ulceration | Lip/oral cavity/tongue ulcer | 0.00 (0.00, 0.23) |
| Ulceration | Ulceration | 0.00 (0.00, 0.23) |
| Upper abdominal | Dyspepsia | 1.06 (0.67, 1.67) |
| Upper abdominal | Dysphagia | 0.53 (0.28, 1.01) |
| Upper abdominal | Early satiety | 0.12 (0.03, 0.43) |
| Upper abdominal | Gastroesophageal reflux | 0.29 (0.13, 0.69) |
| Upper abdominal | Haematemesis | 0.06 (0.01, 0.33) |
| Upper abdominal | Jaundice | 0.18 (0.06, 0.52) |
| Upper abdominal | Loss of appetite | 1.71 (1.19, 2.45) |
| Upper abdominal | Nausea and/or vomiting | 2.06 (1.49, 2.86) |
| Upper abdominal | New onset diabetes | 0.12 (0.03, 0.43) |
| Upper abdominal | Upper abdominal pain | 3.83 (3.02, 4.86) |
| Lower abdominal | Abdominal pain (NOS) | 7.43 (6.28, 8.78) |
| Lower abdominal | Change in bowel habit | 1.71 (1.19, 2.45) |
| Lower abdominal | Constipation | 1.24 (0.81, 1.89) |
| Lower abdominal | Diarrhoea | 1.24 (0.81, 1.89) |
| Lower abdominal | Distension | 1.65 (1.15, 2.38) |
| Lower abdominal | Lower abdominal pain | 3.24 (2.50, 4.20) |
| Lower abdominal | Pelvic pain | 0.71 (0.41, 1.23) |
| Lower abdominal | Rectal bleeding | 1.06 (0.67, 1.67) |
| Respiratory | Chest infection | 0.59 (0.32, 1.08) |
| Respiratory | Chest pain | 1.59 (1.10, 2.31) |
| Respiratory | Clubbing | 0.00 (0.00, 0.23) |
| Respiratory | Cough | 3.07 (2.35, 4.00) |
| Respiratory | Dyspnoea | 2.42 (1.79, 3.26) |
| Respiratory | Haemoptysis | 0.77 (0.45, 1.31) |
| Respiratory | Hoarseness | 0.12 (0.03, 0.43) |
| Respiratory | Sore throat | 0.12 (0.03, 0.43) |
| Respiratory | Stridor | 0.00 (0.00, 0.23) |
| Urological | Dysuria | 2.06 (1.49, 2.86) |
| Urological | Haematuria | 15.0 (13.4, 16.8) |
| Urological | Loin pain | 4.37 (3.49, 5.45) |
| Urological | LUTS (nocturia, frequency, hesitancy, urgency, retention) | 5.25 (4.29, 6.42) |
| Urological | Renal colic | 1.36 (0.91, 2.03) |
| Urological | Urinary tract infection | 3.36 (2.60, 4.33) |
| Central nervous system | Fit/seizure | 0.12 (0.03, 0.43) |
| Central nervous system | Headache | 0.29 (0.13, 0.69) |
| Central nervous system | Prog/sub-acute loss of central neuro funct | 0.47 (0.24, 0.93) |
| Central nervous system | Visual disturbance or loss | 0.12 (0.03, 0.43) |
| Musculoskeletal | Back pain | 4.96 (4.02, 6.09) |
| Musculoskeletal | Bone pain | 1.30 (0.86, 1.96) |
| Musculoskeletal | Fracture | 0.24 (0.09, 0.61) |
| Skin Lesion | Abnormal mole | 0.00 (0.00, 0.23) |
| Skin Lesion | Lesions suspicious of BCC | 0.06 (0.01, 0.33) |
| Skin Lesion | Non-pigmented lesion | 0.00 (0.00, 0.23) |
| Breast Symptoms | Axillary lump/mass | 0.12 (0.03, 0.43) |
| Breast Symptoms | Breast lump/mass | 0.06 (0.01, 0.33) |
| Breast Symptoms | Breast pain | 0.00 (0.00, 0.23) |
| Breast Symptoms | Nipple changes | 0.00 (0.00, 0.23) |
| Breast Symptoms | Nipple discharge | 0.00 (0.00, 0.23) |
| Female specific | Other vaginal bleeding | 0.29 (0.13, 0.69) |
| Female specific | Post-menopausal bleeding | 0.29 (0.13, 0.69) |
| Female specific | Vaginal discharge | 0.00 (0.00, 0.23) |
| Female specific | Vaginal mass | 0.00 (0.00, 0.23) |
| Female specific | Vulval bleeding | 0.00 (0.00, 0.23) |
| Female specific | Vulval mass | 0.00 (0.00, 0.23) |
| Female specific | Vulval ulceration | 0.00 (0.00, 0.23) |
| Male specific | Erectile dysfunction | 0.06 (0.01, 0.33) |
| Male specific | Penile ulceration | 0.06 (0.01, 0.33) |
| Male specific | Testicular lump | 0.18 (0.06, 0.52) |
| Male specific | Testicular pain | 0.59 (0.32, 1.08) |
| None recorded | N/A | 30.1 (28.0, 32.4) |
| None recorded | N/K | 5.96 (4.93, 7.19) |
| *Urological - Ureteric and other urinary (N = 275)* | | |
| Non-specific | Bruising, bleeding or petechiae | 0.36 (0.06, 2.03) |
| Non-specific | Deep vein thrombosis | 0.36 (0.06, 2.03) |
| Non-specific | Epistaxis | 0.00 (0.00, 1.38) |
| Non-specific | Fatigue | 2.91 (1.48, 5.63) |
| Non-specific | Fever | 1.09 (0.37, 3.16) |
| Non-specific | Infection | 0.00 (0.00, 1.38) |
| Non-specific | Night sweats | 0.73 (0.20, 2.61) |
| Non-specific | Other symptom | 2.91 (1.48, 5.63) |
| Non-specific | Pallor | 0.36 (0.06, 2.03) |
| Non-specific | Pruritus | 0.00 (0.00, 1.38) |
| Non-specific | Weight loss | 5.09 (3.06, 8.36) |
| Lump/mass/lymph node | Anal mass | 0.00 (0.00, 1.38) |
| Lump/mass/lymph node | Lymph node pain with alcohol | 0.00 (0.00, 1.38) |
| Lump/mass/lymph node | Lymphadenopathy (generalised) | 0.00 (0.00, 1.38) |
| Lump/mass/lymph node | Lymphadenopathy (localised) | 0.00 (0.00, 1.38) |
| Lump/mass/lymph node | Neck lump/mass | 0.36 (0.06, 2.03) |
| Lump/mass/lymph node | Thyroid lump/mass | 0.00 (0.00, 1.38) |
| Lump/mass/lymph node | Unexplained lump suspicious of sarcoma | 0.00 (0.00, 1.38) |
| Ulceration | Leukoplakia | 0.00 (0.00, 1.38) |
| Ulceration | Lip/oral cavity/tongue lump/mass | 0.00 (0.00, 1.38) |
| Ulceration | Lip/oral cavity/tongue ulcer | 0.00 (0.00, 1.38) |
| Ulceration | Ulceration | 0.36 (0.06, 2.03) |
| Upper abdominal | Dyspepsia | 0.36 (0.06, 2.03) |
| Upper abdominal | Dysphagia | 0.00 (0.00, 1.38) |
| Upper abdominal | Early satiety | 0.00 (0.00, 1.38) |
| Upper abdominal | Gastroesophageal reflux | 0.00 (0.00, 1.38) |
| Upper abdominal | Haematemesis | 0.73 (0.20, 2.61) |
| Upper abdominal | Jaundice | 0.00 (0.00, 1.38) |
| Upper abdominal | Loss of appetite | 1.45 (0.57, 3.68) |
| Upper abdominal | Nausea and/or vomiting | 1.09 (0.37, 3.16) |
| Upper abdominal | New onset diabetes | 0.00 (0.00, 1.38) |
| Upper abdominal | Upper abdominal pain | 1.45 (0.57, 3.68) |
| Lower abdominal | Abdominal pain (NOS) | 6.55 (4.18, 10.1) |
| Lower abdominal | Change in bowel habit | 0.73 (0.20, 2.61) |
| Lower abdominal | Constipation | 1.09 (0.37, 3.16) |
| Lower abdominal | Diarrhoea | 0.73 (0.20, 2.61) |
| Lower abdominal | Distension | 1.09 (0.37, 3.16) |
| Lower abdominal | Lower abdominal pain | 3.64 (1.99, 6.56) |
| Lower abdominal | Pelvic pain | 1.09 (0.37, 3.16) |
| Lower abdominal | Rectal bleeding | 0.36 (0.06, 2.03) |
| Respiratory | Chest infection | 0.00 (0.00, 1.38) |
| Respiratory | Chest pain | 0.36 (0.06, 2.03) |
| Respiratory | Clubbing | 0.00 (0.00, 1.38) |
| Respiratory | Cough | 0.36 (0.06, 2.03) |
| Respiratory | Dyspnoea | 0.73 (0.20, 2.61) |
| Respiratory | Haemoptysis | 0.00 (0.00, 1.38) |
| Respiratory | Hoarseness | 0.00 (0.00, 1.38) |
| Respiratory | Sore throat | 0.36 (0.06, 2.03) |
| Respiratory | Stridor | 0.00 (0.00, 1.38) |
| Urological | Dysuria | 4.36 (2.51, 7.47) |
| Urological | Haematuria | 45.1 (39.3, 51.0) |
| Urological | Loin pain | 2.91 (1.48, 5.63) |
| Urological | LUTS (nocturia, frequency, hesitancy, urgency, retention) | 9.45 (6.53, 13.5) |
| Urological | Renal colic | 1.45 (0.57, 3.68) |
| Urological | Urinary tract infection | 6.18 (3.90, 9.68) |
| Central nervous system | Fit/seizure | 0.00 (0.00, 1.38) |
| Central nervous system | Headache | 0.00 (0.00, 1.38) |
| Central nervous system | Prog/sub-acute loss of central neuro funct | 0.00 (0.00, 1.38) |
| Central nervous system | Visual disturbance or loss | 0.00 (0.00, 1.38) |
| Musculoskeletal | Back pain | 2.55 (1.24, 5.16) |
| Musculoskeletal | Bone pain | 0.36 (0.06, 2.03) |
| Musculoskeletal | Fracture | 0.36 (0.06, 2.03) |
| Skin Lesion | Abnormal mole | 0.00 (0.00, 1.38) |
| Skin Lesion | Lesions suspicious of BCC | 0.00 (0.00, 1.38) |
| Skin Lesion | Non-pigmented lesion | 0.00 (0.00, 1.38) |
| Breast Symptoms | Axillary lump/mass | 0.00 (0.00, 1.38) |
| Breast Symptoms | Breast lump/mass | 0.00 (0.00, 1.38) |
| Breast Symptoms | Breast pain | 0.00 (0.00, 1.38) |
| Breast Symptoms | Nipple changes | 0.00 (0.00, 1.38) |
| Breast Symptoms | Nipple discharge | 0.00 (0.00, 1.38) |
| Female specific | Other vaginal bleeding | 0.36 (0.06, 2.03) |
| Female specific | Post-menopausal bleeding | 0.36 (0.06, 2.03) |
| Female specific | Vaginal discharge | 0.73 (0.20, 2.61) |
| Female specific | Vaginal mass | 0.36 (0.06, 2.03) |
| Female specific | Vulval bleeding | 0.00 (0.00, 1.38) |
| Female specific | Vulval mass | 0.00 (0.00, 1.38) |
| Female specific | Vulval ulceration | 0.00 (0.00, 1.38) |
| Male specific | Erectile dysfunction | 0.00 (0.00, 1.38) |
| Male specific | Penile ulceration | 0.36 (0.06, 2.03) |
| Male specific | Testicular lump | 0.00 (0.00, 1.38) |
| Male specific | Testicular pain | 0.00 (0.00, 1.38) |
| None recorded | N/A | 13.1 (9.61, 17.6) |
| None recorded | N/K | 6.91 (4.47, 10.5) |
| *Haematological - Acute leukaemia (N = 471)* | | |
| Non-specific | Bruising, bleeding or petechiae | 7.01 (5.03, 9.68) |
| Non-specific | Deep vein thrombosis | 0.42 (0.12, 1.53) |
| Non-specific | Epistaxis | 0.64 (0.22, 1.86) |
| Non-specific | Fatigue | 25.9 (22.2, 30.0) |
| Non-specific | Fever | 5.31 (3.62, 7.72) |
| Non-specific | Infection | 4.03 (2.60, 6.21) |
| Non-specific | Night sweats | 3.40 (2.10, 5.45) |
| Non-specific | Other symptom | 8.70 (6.48, 11.6) |
| Non-specific | Pallor | 2.97 (1.78, 4.93) |
| Non-specific | Pruritus | 0.85 (0.33, 2.16) |
| Non-specific | Weight loss | 8.49 (6.30, 11.4) |
| Lump/mass/lymph node | Anal mass | 0.21 (0.04, 1.19) |
| Lump/mass/lymph node | Lymph node pain with alcohol | 0.00 (0.00, 0.81) |
| Lump/mass/lymph node | Lymphadenopathy (generalised) | 0.00 (0.00, 0.81) |
| Lump/mass/lymph node | Lymphadenopathy (localised) | 0.64 (0.22, 1.86) |
| Lump/mass/lymph node | Neck lump/mass | 1.49 (0.72, 3.04) |
| Lump/mass/lymph node | Thyroid lump/mass | 0.00 (0.00, 0.81) |
| Lump/mass/lymph node | Unexplained lump suspicious of sarcoma | 0.00 (0.00, 0.81) |
| Ulceration | Leukoplakia | 0.00 (0.00, 0.81) |
| Ulceration | Lip/oral cavity/tongue lump/mass | 0.00 (0.00, 0.81) |
| Ulceration | Lip/oral cavity/tongue ulcer | 0.64 (0.22, 1.86) |
| Ulceration | Ulceration | 1.06 (0.45, 2.46) |
| Upper abdominal | Dyspepsia | 0.21 (0.04, 1.19) |
| Upper abdominal | Dysphagia | 0.42 (0.12, 1.53) |
| Upper abdominal | Early satiety | 0.00 (0.00, 0.81) |
| Upper abdominal | Gastroesophageal reflux | 0.42 (0.12, 1.53) |
| Upper abdominal | Haematemesis | 0.21 (0.04, 1.19) |
| Upper abdominal | Jaundice | 0.42 (0.12, 1.53) |
| Upper abdominal | Loss of appetite | 5.31 (3.62, 7.72) |
| Upper abdominal | Nausea and/or vomiting | 5.10 (3.45, 7.47) |
| Upper abdominal | New onset diabetes | 0.00 (0.00, 0.81) |
| Upper abdominal | Upper abdominal pain | 1.91 (1.01, 3.59) |
| Lower abdominal | Abdominal pain (NOS) | 3.18 (1.94, 5.19) |
| Lower abdominal | Change in bowel habit | 0.64 (0.22, 1.86) |
| Lower abdominal | Constipation | 0.21 (0.04, 1.19) |
| Lower abdominal | Diarrhoea | 1.27 (0.59, 2.75) |
| Lower abdominal | Distension | 0.42 (0.12, 1.53) |
| Lower abdominal | Lower abdominal pain | 1.06 (0.45, 2.46) |
| Lower abdominal | Pelvic pain | 0.21 (0.04, 1.19) |
| Lower abdominal | Rectal bleeding | 0.85 (0.33, 2.16) |
| Respiratory | Chest infection | 3.82 (2.43, 5.96) |
| Respiratory | Chest pain | 4.67 (3.10, 6.97) |
| Respiratory | Clubbing | 0.42 (0.12, 1.53) |
| Respiratory | Cough | 4.88 (3.28, 7.22) |
| Respiratory | Dyspnoea | 13.6 (10.8, 17.0) |
| Respiratory | Haemoptysis | 0.64 (0.22, 1.86) |
| Respiratory | Hoarseness | 0.00 (0.00, 0.81) |
| Respiratory | Sore throat | 3.40 (2.10, 5.45) |
| Respiratory | Stridor | 0.00 (0.00, 0.81) |
| Urological | Dysuria | 0.85 (0.33, 2.16) |
| Urological | Haematuria | 1.27 (0.59, 2.75) |
| Urological | Loin pain | 1.06 (0.45, 2.46) |
| Urological | LUTS (nocturia, frequency, hesitancy, urgency, retention) | 0.42 (0.12, 1.53) |
| Urological | Renal colic | 0.21 (0.04, 1.19) |
| Urological | Urinary tract infection | 1.27 (0.59, 2.75) |
| Central nervous system | Fit/seizure | 0.00 (0.00, 0.81) |
| Central nervous system | Headache | 2.97 (1.78, 4.93) |
| Central nervous system | Prog/sub-acute loss of central neuro funct | 0.42 (0.12, 1.53) |
| Central nervous system | Visual disturbance or loss | 0.00 (0.00, 0.81) |
| Musculoskeletal | Back pain | 3.18 (1.94, 5.19) |
| Musculoskeletal | Bone pain | 2.12 (1.16, 3.86) |
| Musculoskeletal | Fracture | 0.00 (0.00, 0.81) |
| Skin Lesion | Abnormal mole | 0.00 (0.00, 0.81) |
| Skin Lesion | Lesions suspicious of BCC | 0.00 (0.00, 0.81) |
| Skin Lesion | Non-pigmented lesion | 0.00 (0.00, 0.81) |
| Breast Symptoms | Axillary lump/mass | 0.21 (0.04, 1.19) |
| Breast Symptoms | Breast lump/mass | 0.21 (0.04, 1.19) |
| Breast Symptoms | Breast pain | 0.00 (0.00, 0.81) |
| Breast Symptoms | Nipple changes | 0.00 (0.00, 0.81) |
| Breast Symptoms | Nipple discharge | 0.00 (0.00, 0.81) |
| Female specific | Other vaginal bleeding | 0.42 (0.12, 1.53) |
| Female specific | Post-menopausal bleeding | 0.00 (0.00, 0.81) |
| Female specific | Vaginal discharge | 0.21 (0.04, 1.19) |
| Female specific | Vaginal mass | 0.00 (0.00, 0.81) |
| Female specific | Vulval bleeding | 0.00 (0.00, 0.81) |
| Female specific | Vulval mass | 0.00 (0.00, 0.81) |
| Female specific | Vulval ulceration | 0.00 (0.00, 0.81) |
| Male specific | Erectile dysfunction | 0.21 (0.04, 1.19) |
| Male specific | Penile ulceration | 0.00 (0.00, 0.81) |
| Male specific | Testicular lump | 0.00 (0.00, 0.81) |
| Male specific | Testicular pain | 0.64 (0.22, 1.86) |
| None recorded | N/A | 22.5 (19.0, 26.5) |
| None recorded | N/K | 5.52 (3.79, 7.97) |
| Haematological - Chronic lymphocytic leukaemia (N = 529) | | |
| Non-specific | Bruising, bleeding or petechiae | 0.76 (0.29, 1.93) |
| Non-specific | Deep vein thrombosis | 0.00 (0.00, 0.72) |
| Non-specific | Epistaxis | 0.19 (0.03, 1.06) |
| Non-specific | Fatigue | 7.75 (5.76, 10.3) |
| Non-specific | Fever | 0.95 (0.40, 2.19) |
| Non-specific | Infection | 1.13 (0.52, 2.45) |
| Non-specific | Night sweats | 2.65 (1.58, 4.39) |
| Non-specific | Other symptom | 10.2 (7.91, 13.1) |
| Non-specific | Pallor | 0.57 (0.19, 1.65) |
| Non-specific | Pruritus | 0.57 (0.19, 1.65) |
| Non-specific | Weight loss | 4.16 (2.76, 6.22) |
| Lump/mass/lymph node | Anal mass | 0.19 (0.03, 1.06) |
| Lump/mass/lymph node | Lymph node pain with alcohol | 0.00 (0.00, 0.72) |
| Lump/mass/lymph node | Lymphadenopathy (generalised) | 0.76 (0.29, 1.93) |
| Lump/mass/lymph node | Lymphadenopathy (localised) | 3.02 (1.87, 4.86) |
| Lump/mass/lymph node | Neck lump/mass | 4.73 (3.22, 6.88) |
| Lump/mass/lymph node | Thyroid lump/mass | 0.19 (0.03, 1.06) |
| Lump/mass/lymph node | Unexplained lump suspicious of sarcoma | 0.38 (0.10, 1.37) |
| Ulceration | Leukoplakia | 0.00 (0.00, 0.72) |
| Ulceration | Lip/oral cavity/tongue lump/mass | 0.19 (0.03, 1.06) |
| Ulceration | Lip/oral cavity/tongue ulcer | 0.00 (0.00, 0.72) |
| Ulceration | Ulceration | 0.19 (0.03, 1.06) |
| Upper abdominal | Dyspepsia | 0.38 (0.10, 1.37) |
| Upper abdominal | Dysphagia | 0.38 (0.10, 1.37) |
| Upper abdominal | Early satiety | 0.19 (0.03, 1.06) |
| Upper abdominal | Gastroesophageal reflux | 0.19 (0.03, 1.06) |
| Upper abdominal | Haematemesis | 0.00 (0.00, 0.72) |
| Upper abdominal | Jaundice | 0.19 (0.03, 1.06) |
| Upper abdominal | Loss of appetite | 0.57 (0.19, 1.65) |
| Upper abdominal | Nausea and/or vomiting | 1.32 (0.64, 2.71) |
| Upper abdominal | New onset diabetes | 0.00 (0.00, 0.72) |
| Upper abdominal | Upper abdominal pain | 0.38 (0.10, 1.37) |
| Lower abdominal | Abdominal pain (NOS) | 3.02 (1.87, 4.86) |
| Lower abdominal | Change in bowel habit | 0.38 (0.10, 1.37) |
| Lower abdominal | Constipation | 0.57 (0.19, 1.65) |
| Lower abdominal | Diarrhoea | 1.51 (0.77, 2.96) |
| Lower abdominal | Distension | 0.76 (0.29, 1.93) |
| Lower abdominal | Lower abdominal pain | 0.57 (0.19, 1.65) |
| Lower abdominal | Pelvic pain | 0.19 (0.03, 1.06) |
| Lower abdominal | Rectal bleeding | 0.00 (0.00, 0.72) |
| Respiratory | Chest infection | 1.13 (0.52, 2.45) |
| Respiratory | Chest pain | 2.08 (1.16, 3.68) |
| Respiratory | Clubbing | 0.00 (0.00, 0.72) |
| Respiratory | Cough | 2.08 (1.16, 3.68) |
| Respiratory | Dyspnoea | 2.84 (1.73, 4.63) |
| Respiratory | Haemoptysis | 0.00 (0.00, 0.72) |
| Respiratory | Hoarseness | 0.00 (0.00, 0.72) |
| Respiratory | Sore throat | 0.57 (0.19, 1.65) |
| Respiratory | Stridor | 0.00 (0.00, 0.72) |
| Urological | Dysuria | 0.19 (0.03, 1.06) |
| Urological | Haematuria | 0.76 (0.29, 1.93) |
| Urological | Loin pain | 0.00 (0.00, 0.72) |
| Urological | LUTS (nocturia, frequency, hesitancy, urgency, retention) | 0.95 (0.40, 2.19) |
| Urological | Renal colic | 0.00 (0.00, 0.72) |
| Urological | Urinary tract infection | 0.38 (0.10, 1.37) |
| Central nervous system | Fit/seizure | 0.00 (0.00, 0.72) |
| Central nervous system | Headache | 0.38 (0.10, 1.37) |
| Central nervous system | Prog/sub-acute loss of central neuro funct | 0.38 (0.10, 1.37) |
| Central nervous system | Visual disturbance or loss | 0.38 (0.10, 1.37) |
| Musculoskeletal | Back pain | 1.89 (1.03, 3.44) |
| Musculoskeletal | Bone pain | 1.51 (0.77, 2.96) |
| Musculoskeletal | Fracture | 0.00 (0.00, 0.72) |
| Skin Lesion | Abnormal mole | 0.00 (0.00, 0.72) |
| Skin Lesion | Lesions suspicious of BCC | 0.00 (0.00, 0.72) |
| Skin Lesion | Non-pigmented lesion | 0.00 (0.00, 0.72) |
| Breast Symptoms | Axillary lump/mass | 0.95 (0.40, 2.19) |
| Breast Symptoms | Breast lump/mass | 0.19 (0.03, 1.06) |
| Breast Symptoms | Breast pain | 0.19 (0.03, 1.06) |
| Breast Symptoms | Nipple changes | 0.00 (0.00, 0.72) |
| Breast Symptoms | Nipple discharge | 0.00 (0.00, 0.72) |
| Female specific | Other vaginal bleeding | 0.00 (0.00, 0.72) |
| Female specific | Post-menopausal bleeding | 0.00 (0.00, 0.72) |
| Female specific | Vaginal discharge | 0.00 (0.00, 0.72) |
| Female specific | Vaginal mass | 0.00 (0.00, 0.72) |
| Female specific | Vulval bleeding | 0.00 (0.00, 0.72) |
| Female specific | Vulval mass | 0.00 (0.00, 0.72) |
| Female specific | Vulval ulceration | 0.00 (0.00, 0.72) |
| Male specific | Erectile dysfunction | 0.19 (0.03, 1.06) |
| Male specific | Penile ulceration | 0.00 (0.00, 0.72) |
| Male specific | Testicular lump | 0.19 (0.03, 1.06) |
| Male specific | Testicular pain | 0.00 (0.00, 0.72) |
| None recorded | N/A | 43.5 (39.3, 47.7) |
| None recorded | N/K | 8.88 (6.75, 11.6) |
| *Haematological - Hodgkin lymphoma (N = 243)* | | |
| Non-specific | Bruising, bleeding or petechiae | 0.00 (0.00, 1.56) |
| Non-specific | Deep vein thrombosis | 0.00 (0.00, 1.56) |
| Non-specific | Epistaxis | 0.00 (0.00, 1.56) |
| Non-specific | Fatigue | 11.5 (8.09, 16.1) |
| Non-specific | Fever | 3.29 (1.68, 6.36) |
| Non-specific | Infection | 0.82 (0.23, 2.95) |
| Non-specific | Night sweats | 5.76 (3.46, 9.44) |
| Non-specific | Other symptom | 7.00 (4.41, 10.9) |
| Non-specific | Pallor | 0.00 (0.00, 1.56) |
| Non-specific | Pruritus | 2.47 (1.14, 5.28) |
| Non-specific | Weight loss | 11.1 (7.75, 15.7) |
| Lump/mass/lymph node | Anal mass | 0.00 (0.00, 1.56) |
| Lump/mass/lymph node | Lymph node pain with alcohol | 0.00 (0.00, 1.56) |
| Lump/mass/lymph node | Lymphadenopathy (generalised) | 1.23 (0.42, 3.57) |
| Lump/mass/lymph node | Lymphadenopathy (localised) | 11.9 (8.44, 16.6) |
| Lump/mass/lymph node | Neck lump/mass | 28.4 (23.1, 34.4) |
| Lump/mass/lymph node | Thyroid lump/mass | 0.00 (0.00, 1.56) |
| Lump/mass/lymph node | Unexplained lump suspicious of sarcoma | 0.82 (0.23, 2.95) |
| Ulceration | Leukoplakia | 0.00 (0.00, 1.56) |
| Ulceration | Lip/oral cavity/tongue lump/mass | 0.41 (0.07, 2.29) |
| Ulceration | Lip/oral cavity/tongue ulcer | 0.00 (0.00, 1.56) |
| Ulceration | Ulceration | 0.00 (0.00, 1.56) |
| Upper abdominal | Dyspepsia | 0.82 (0.23, 2.95) |
| Upper abdominal | Dysphagia | 0.82 (0.23, 2.95) |
| Upper abdominal | Early satiety | 0.82 (0.23, 2.95) |
| Upper abdominal | Gastroesophageal reflux | 0.82 (0.23, 2.95) |
| Upper abdominal | Haematemesis | 0.00 (0.00, 1.56) |
| Upper abdominal | Jaundice | 0.00 (0.00, 1.56) |
| Upper abdominal | Loss of appetite | 4.94 (2.85, 8.43) |
| Upper abdominal | Nausea and/or vomiting | 1.23 (0.42, 3.57) |
| Upper abdominal | New onset diabetes | 0.00 (0.00, 1.56) |
| Upper abdominal | Upper abdominal pain | 1.23 (0.42, 3.57) |
| Lower abdominal | Abdominal pain (NOS) | 0.82 (0.23, 2.95) |
| Lower abdominal | Change in bowel habit | 0.82 (0.23, 2.95) |
| Lower abdominal | Constipation | 0.00 (0.00, 1.56) |
| Lower abdominal | Diarrhoea | 1.65 (0.64, 4.16) |
| Lower abdominal | Distension | 0.00 (0.00, 1.56) |
| Lower abdominal | Lower abdominal pain | 0.82 (0.23, 2.95) |
| Lower abdominal | Pelvic pain | 0.41 (0.07, 2.29) |
| Lower abdominal | Rectal bleeding | 0.82 (0.23, 2.95) |
| Respiratory | Chest infection | 0.82 (0.23, 2.95) |
| Respiratory | Chest pain | 2.06 (0.88, 4.73) |
| Respiratory | Clubbing | 0.00 (0.00, 1.56) |
| Respiratory | Cough | 7.82 (5.06, 11.9) |
| Respiratory | Dyspnoea | 1.65 (0.64, 4.16) |
| Respiratory | Haemoptysis | 0.00 (0.00, 1.56) |
| Respiratory | Hoarseness | 1.23 (0.42, 3.57) |
| Respiratory | Sore throat | 1.65 (0.64, 4.16) |
| Respiratory | Stridor | 0.00 (0.00, 1.56) |
| Urological | Dysuria | 0.00 (0.00, 1.56) |
| Urological | Haematuria | 0.41 (0.07, 2.29) |
| Urological | Loin pain | 0.41 (0.07, 2.29) |
| Urological | LUTS (nocturia, frequency, hesitancy, urgency, retention) | 0.82 (0.23, 2.95) |
| Urological | Renal colic | 0.00 (0.00, 1.56) |
| Urological | Urinary tract infection | 0.00 (0.00, 1.56) |
| Central nervous system | Fit/seizure | 0.00 (0.00, 1.56) |
| Central nervous system | Headache | 1.65 (0.64, 4.16) |
| Central nervous system | Prog/sub-acute loss of central neuro funct | 0.00 (0.00, 1.56) |
| Central nervous system | Visual disturbance or loss | 0.00 (0.00, 1.56) |
| Musculoskeletal | Back pain | 2.47 (1.14, 5.28) |
| Musculoskeletal | Bone pain | 1.23 (0.42, 3.57) |
| Musculoskeletal | Fracture | 0.00 (0.00, 1.56) |
| Skin Lesion | Abnormal mole | 0.00 (0.00, 1.56) |
| Skin Lesion | Lesions suspicious of BCC | 0.00 (0.00, 1.56) |
| Skin Lesion | Non-pigmented lesion | 0.00 (0.00, 1.56) |
| Breast Symptoms | Axillary lump/mass | 9.05 (6.05, 13.3) |
| Breast Symptoms | Breast lump/mass | 1.23 (0.42, 3.57) |
| Breast Symptoms | Breast pain | 0.41 (0.07, 2.29) |
| Breast Symptoms | Nipple changes | 0.00 (0.00, 1.56) |
| Breast Symptoms | Nipple discharge | 0.00 (0.00, 1.56) |
| Female specific | Other vaginal bleeding | 0.41 (0.07, 2.29) |
| Female specific | Post-menopausal bleeding | 0.00 (0.00, 1.56) |
| Female specific | Vaginal discharge | 0.00 (0.00, 1.56) |
| Female specific | Vaginal mass | 0.00 (0.00, 1.56) |
| Female specific | Vulval bleeding | 0.00 (0.00, 1.56) |
| Female specific | Vulval mass | 0.00 (0.00, 1.56) |
| Female specific | Vulval ulceration | 0.00 (0.00, 1.56) |
| Male specific | Erectile dysfunction | 0.00 (0.00, 1.56) |
| Male specific | Penile ulceration | 0.00 (0.00, 1.56) |
| Male specific | Testicular lump | 0.41 (0.07, 2.29) |
| Male specific | Testicular pain | 0.82 (0.23, 2.95) |
| None recorded | N/A | 9.47 (6.39, 13.8) |
| None recorded | N/K | 4.53 (2.55, 7.92) |
| *Haematological - Multiple myeloma (N = 904)* | | |
| Non-specific | Bruising, bleeding or petechiae | 1.11 (0.60, 2.02) |
| Non-specific | Deep vein thrombosis | 0.22 (0.06, 0.80) |
| Non-specific | Epistaxis | 0.88 (0.45, 1.74) |
| Non-specific | Fatigue | 9.62 (7.87, 11.7) |
| Non-specific | Fever | 0.77 (0.38, 1.59) |
| Non-specific | Infection | 0.66 (0.30, 1.44) |
| Non-specific | Night sweats | 1.55 (0.92, 2.58) |
| Non-specific | Other symptom | 8.41 (6.77, 10.4) |
| Non-specific | Pallor | 0.44 (0.17, 1.13) |
| Non-specific | Pruritus | 1.11 (0.60, 2.02) |
| Non-specific | Weight loss | 7.63 (6.08, 9.55) |
| Lump/mass/lymph node | Anal mass | 0.00 (0.00, 0.42) |
| Lump/mass/lymph node | Lymph node pain with alcohol | 0.00 (0.00, 0.42) |
| Lump/mass/lymph node | Lymphadenopathy (generalised) | 0.00 (0.00, 0.42) |
| Lump/mass/lymph node | Lymphadenopathy (localised) | 0.22 (0.06, 0.80) |
| Lump/mass/lymph node | Neck lump/mass | 0.66 (0.30, 1.44) |
| Lump/mass/lymph node | Thyroid lump/mass | 0.00 (0.00, 0.42) |
| Lump/mass/lymph node | Unexplained lump suspicious of sarcoma | 0.11 (0.02, 0.62) |
| Ulceration | Leukoplakia | 0.00 (0.00, 0.42) |
| Ulceration | Lip/oral cavity/tongue lump/mass | 0.00 (0.00, 0.42) |
| Ulceration | Lip/oral cavity/tongue ulcer | 0.11 (0.02, 0.62) |
| Ulceration | Ulceration | 0.11 (0.02, 0.62) |
| Upper abdominal | Dyspepsia | 0.22 (0.06, 0.80) |
| Upper abdominal | Dysphagia | 0.22 (0.06, 0.80) |
| Upper abdominal | Early satiety | 0.00 (0.00, 0.42) |
| Upper abdominal | Gastroesophageal reflux | 0.11 (0.02, 0.62) |
| Upper abdominal | Haematemesis | 0.22 (0.06, 0.80) |
| Upper abdominal | Jaundice | 0.00 (0.00, 0.42) |
| Upper abdominal | Loss of appetite | 3.98 (2.89, 5.46) |
| Upper abdominal | Nausea and/or vomiting | 2.10 (1.35, 3.26) |
| Upper abdominal | New onset diabetes | 0.00 (0.00, 0.42) |
| Upper abdominal | Upper abdominal pain | 1.00 (0.52, 1.88) |
| Lower abdominal | Abdominal pain (NOS) | 1.66 (1.01, 2.72) |
| Lower abdominal | Change in bowel habit | 1.22 (0.68, 2.17) |
| Lower abdominal | Constipation | 0.55 (0.24, 1.29) |
| Lower abdominal | Diarrhoea | 1.33 (0.76, 2.31) |
| Lower abdominal | Distension | 0.44 (0.17, 1.13) |
| Lower abdominal | Lower abdominal pain | 0.55 (0.24, 1.29) |
| Lower abdominal | Pelvic pain | 0.77 (0.38, 1.59) |
| Lower abdominal | Rectal bleeding | 0.88 (0.45, 1.74) |
| Respiratory | Chest infection | 1.33 (0.76, 2.31) |
| Respiratory | Chest pain | 5.09 (3.84, 6.72) |
| Respiratory | Clubbing | 0.00 (0.00, 0.42) |
| Respiratory | Cough | 3.32 (2.33, 4.70) |
| Respiratory | Dyspnoea | 3.98 (2.89, 5.46) |
| Respiratory | Haemoptysis | 0.33 (0.11, 0.97) |
| Respiratory | Hoarseness | 0.11 (0.02, 0.62) |
| Respiratory | Sore throat | 0.33 (0.11, 0.97) |
| Respiratory | Stridor | 0.11 (0.02, 0.62) |
| Urological | Dysuria | 0.11 (0.02, 0.62) |
| Urological | Haematuria | 0.44 (0.17, 1.13) |
| Urological | Loin pain | 0.77 (0.38, 1.59) |
| Urological | LUTS (nocturia, frequency, hesitancy, urgency, retention) | 1.11 (0.60, 2.02) |
| Urological | Renal colic | 0.11 (0.02, 0.62) |
| Urological | Urinary tract infection | 0.22 (0.06, 0.80) |
| Central nervous system | Fit/seizure | 0.22 (0.06, 0.80) |
| Central nervous system | Headache | 0.88 (0.45, 1.74) |
| Central nervous system | Prog/sub-acute loss of central neuro funct | 0.77 (0.38, 1.59) |
| Central nervous system | Visual disturbance or loss | 0.22 (0.06, 0.80) |
| Musculoskeletal | Back pain | 22.8 (20.2, 25.6) |
| Musculoskeletal | Bone pain | 8.85 (7.17, 10.9) |
| Musculoskeletal | Fracture | 1.44 (0.84, 2.44) |
| Skin Lesion | Abnormal mole | 0.00 (0.00, 0.42) |
| Skin Lesion | Lesions suspicious of BCC | 0.11 (0.02, 0.62) |
| Skin Lesion | Non-pigmented lesion | 0.00 (0.00, 0.42) |
| Breast Symptoms | Axillary lump/mass | 0.11 (0.02, 0.62) |
| Breast Symptoms | Breast lump/mass | 0.00 (0.00, 0.42) |
| Breast Symptoms | Breast pain | 0.11 (0.02, 0.62) |
| Breast Symptoms | Nipple changes | 0.00 (0.00, 0.42) |
| Breast Symptoms | Nipple discharge | 0.00 (0.00, 0.42) |
| Female specific | Other vaginal bleeding | 0.00 (0.00, 0.42) |
| Female specific | Post-menopausal bleeding | 0.11 (0.02, 0.62) |
| Female specific | Vaginal discharge | 0.00 (0.00, 0.42) |
| Female specific | Vaginal mass | 0.00 (0.00, 0.42) |
| Female specific | Vulval bleeding | 0.00 (0.00, 0.42) |
| Female specific | Vulval mass | 0.00 (0.00, 0.42) |
| Female specific | Vulval ulceration | 0.00 (0.00, 0.42) |
| Male specific | Erectile dysfunction | 0.00 (0.00, 0.42) |
| Male specific | Penile ulceration | 0.11 (0.02, 0.62) |
| Male specific | Testicular lump | 0.11 (0.02, 0.62) |
| Male specific | Testicular pain | 0.11 (0.02, 0.62) |
| None recorded | N/A | 26.0 (23.2, 29.0) |
| None recorded | N/K | 7.41 (5.88, 9.31) |
| *Haematological - Non-Hodgkin lymphoma (N = 2055)* | | |
| Non-specific | Bruising, bleeding or petechiae | 0.49 (0.26, 0.89) |
| Non-specific | Deep vein thrombosis | 0.58 (0.33, 1.02) |
| Non-specific | Epistaxis | 0.34 (0.17, 0.70) |
| Non-specific | Fatigue | 6.76 (5.76, 7.93) |
| Non-specific | Fever | 0.88 (0.55, 1.38) |
| Non-specific | Infection | 0.39 (0.20, 0.77) |
| Non-specific | Night sweats | 2.38 (1.81, 3.14) |
| Non-specific | Other symptom | 8.91 (7.75, 10.2) |
| Non-specific | Pallor | 0.39 (0.20, 0.77) |
| Non-specific | Pruritus | 0.78 (0.48, 1.26) |
| Non-specific | Weight loss | 8.71 (7.57, 10.0) |
| Lump/mass/lymph node | Anal mass | 0.05 (0.01, 0.28) |
| Lump/mass/lymph node | Lymph node pain with alcohol | 0.00 (0.00, 0.19) |
| Lump/mass/lymph node | Lymphadenopathy (generalised) | 0.83 (0.52, 1.32) |
| Lump/mass/lymph node | Lymphadenopathy (localised) | 7.45 (6.39, 8.66) |
| Lump/mass/lymph node | Neck lump/mass | 13.1 (11.7, 14.7) |
| Lump/mass/lymph node | Thyroid lump/mass | 0.15 (0.05, 0.43) |
| Lump/mass/lymph node | Unexplained lump suspicious of sarcoma | 1.41 (0.98, 2.02) |
| Ulceration | Leukoplakia | 0.00 (0.00, 0.19) |
| Ulceration | Lip/oral cavity/tongue lump/mass | 0.78 (0.48, 1.26) |
| Ulceration | Lip/oral cavity/tongue ulcer | 0.19 (0.08, 0.50) |
| Ulceration | Ulceration | 0.29 (0.13, 0.64) |
| Upper abdominal | Dyspepsia | 1.56 (1.11, 2.19) |
| Upper abdominal | Dysphagia | 1.07 (0.71, 1.62) |
| Upper abdominal | Early satiety | 0.39 (0.20, 0.77) |
| Upper abdominal | Gastroesophageal reflux | 0.49 (0.26, 0.89) |
| Upper abdominal | Haematemesis | 0.29 (0.13, 0.64) |
| Upper abdominal | Jaundice | 0.44 (0.23, 0.83) |
| Upper abdominal | Loss of appetite | 3.80 (3.05, 4.71) |
| Upper abdominal | Nausea and/or vomiting | 2.34 (1.77, 3.08) |
| Upper abdominal | New onset diabetes | 0.00 (0.00, 0.19) |
| Upper abdominal | Upper abdominal pain | 2.77 (2.15, 3.58) |
| Lower abdominal | Abdominal pain (NOS) | 6.42 (5.44, 7.57) |
| Lower abdominal | Change in bowel habit | 1.95 (1.43, 2.64) |
| Lower abdominal | Constipation | 1.65 (1.19, 2.30) |
| Lower abdominal | Diarrhoea | 1.46 (1.02, 2.08) |
| Lower abdominal | Distension | 2.24 (1.68, 2.97) |
| Lower abdominal | Lower abdominal pain | 1.65 (1.19, 2.30) |
| Lower abdominal | Pelvic pain | 0.63 (0.37, 1.08) |
| Lower abdominal | Rectal bleeding | 0.88 (0.55, 1.38) |
| Respiratory | Chest infection | 1.31 (0.90, 1.90) |
| Respiratory | Chest pain | 1.70 (1.23, 2.36) |
| Respiratory | Clubbing | 0.00 (0.00, 0.19) |
| Respiratory | Cough | 4.33 (3.53, 5.30) |
| Respiratory | Dyspnoea | 4.53 (3.71, 5.51) |
| Respiratory | Haemoptysis | 0.34 (0.17, 0.70) |
| Respiratory | Hoarseness | 0.63 (0.37, 1.08) |
| Respiratory | Sore throat | 1.90 (1.39, 2.58) |
| Respiratory | Stridor | 0.00 (0.00, 0.19) |
| Urological | Dysuria | 0.15 (0.05, 0.43) |
| Urological | Haematuria | 0.44 (0.23, 0.83) |
| Urological | Loin pain | 0.54 (0.30, 0.96) |
| Urological | LUTS (nocturia, frequency, hesitancy, urgency, retention) | 1.31 (0.90, 1.90) |
| Urological | Renal colic | 0.10 (0.03, 0.35) |
| Urological | Urinary tract infection | 0.44 (0.23, 0.83) |
| Central nervous system | Fit/seizure | 0.19 (0.08, 0.50) |
| Central nervous system | Headache | 1.07 (0.71, 1.62) |
| Central nervous system | Prog/sub-acute loss of central neuro funct | 1.17 (0.79, 1.73) |
| Central nervous system | Visual disturbance or loss | 0.54 (0.30, 0.96) |
| Musculoskeletal | Back pain | 4.23 (3.45, 5.19) |
| Musculoskeletal | Bone pain | 1.80 (1.31, 2.47) |
| Musculoskeletal | Fracture | 0.19 (0.08, 0.50) |
| Skin Lesion | Abnormal mole | 0.24 (0.10, 0.57) |
| Skin Lesion | Lesions suspicious of BCC | 0.34 (0.17, 0.70) |
| Skin Lesion | Non-pigmented lesion | 0.29 (0.13, 0.64) |
| Breast Symptoms | Axillary lump/mass | 1.95 (1.43, 2.64) |
| Breast Symptoms | Breast lump/mass | 1.65 (1.19, 2.30) |
| Breast Symptoms | Breast pain | 0.34 (0.17, 0.70) |
| Breast Symptoms | Nipple changes | 0.05 (0.01, 0.28) |
| Breast Symptoms | Nipple discharge | 0.00 (0.00, 0.19) |
| Female specific | Other vaginal bleeding | 0.05 (0.01, 0.28) |
| Female specific | Post-menopausal bleeding | 0.10 (0.03, 0.35) |
| Female specific | Vaginal discharge | 0.00 (0.00, 0.19) |
| Female specific | Vaginal mass | 0.00 (0.00, 0.19) |
| Female specific | Vulval bleeding | 0.00 (0.00, 0.19) |
| Female specific | Vulval mass | 0.00 (0.00, 0.19) |
| Female specific | Vulval ulceration | 0.05 (0.01, 0.28) |
| Male specific | Erectile dysfunction | 0.10 (0.03, 0.35) |
| Male specific | Penile ulceration | 0.00 (0.00, 0.19) |
| Male specific | Testicular lump | 0.83 (0.52, 1.32) |
| Male specific | Testicular pain | 0.34 (0.17, 0.70) |
| None recorded | N/A | 17.5 (15.9, 19.2) |
| None recorded | N/K | 4.43 (3.62, 5.41) |
| *Haematological - Other haematological (N = 588)* | | |
| Non-specific | Bruising, bleeding or petechiae | 2.55 (1.55, 4.17) |
| Non-specific | Deep vein thrombosis | 0.00 (0.00, 0.65) |
| Non-specific | Epistaxis | 0.85 (0.36, 1.97) |
| Non-specific | Fatigue | 12.9 (10.5, 15.9) |
| Non-specific | Fever | 1.70 (0.93, 3.10) |
| Non-specific | Infection | 1.53 (0.81, 2.88) |
| Non-specific | Night sweats | 4.25 (2.90, 6.20) |
| Non-specific | Other symptom | 13.4 (10.9, 16.4) |
| Non-specific | Pallor | 1.36 (0.69, 2.66) |
| Non-specific | Pruritus | 4.42 (3.04, 6.40) |
| Non-specific | Weight loss | 7.99 (6.06, 10.5) |
| Lump/mass/lymph node | Anal mass | 0.00 (0.00, 0.65) |
| Lump/mass/lymph node | Lymph node pain with alcohol | 0.00 (0.00, 0.65) |
| Lump/mass/lymph node | Lymphadenopathy (generalised) | 0.34 (0.09, 1.23) |
| Lump/mass/lymph node | Lymphadenopathy (localised) | 2.89 (1.81, 4.58) |
| Lump/mass/lymph node | Neck lump/mass | 3.74 (2.48, 5.60) |
| Lump/mass/lymph node | Thyroid lump/mass | 0.00 (0.00, 0.65) |
| Lump/mass/lymph node | Unexplained lump suspicious of sarcoma | 0.34 (0.09, 1.23) |
| Ulceration | Leukoplakia | 0.00 (0.00, 0.65) |
| Ulceration | Lip/oral cavity/tongue lump/mass | 0.17 (0.03, 0.96) |
| Ulceration | Lip/oral cavity/tongue ulcer | 0.34 (0.09, 1.23) |
| Ulceration | Ulceration | 0.85 (0.36, 1.97) |
| Upper abdominal | Dyspepsia | 0.51 (0.17, 1.49) |
| Upper abdominal | Dysphagia | 0.17 (0.03, 0.96) |
| Upper abdominal | Early satiety | 0.17 (0.03, 0.96) |
| Upper abdominal | Gastroesophageal reflux | 0.34 (0.09, 1.23) |
| Upper abdominal | Haematemesis | 0.17 (0.03, 0.96) |
| Upper abdominal | Jaundice | 0.00 (0.00, 0.65) |
| Upper abdominal | Loss of appetite | 3.40 (2.21, 5.20) |
| Upper abdominal | Nausea and/or vomiting | 1.53 (0.81, 2.88) |
| Upper abdominal | New onset diabetes | 0.00 (0.00, 0.65) |
| Upper abdominal | Upper abdominal pain | 1.53 (0.81, 2.88) |
| Lower abdominal | Abdominal pain (NOS) | 2.72 (1.68, 4.37) |
| Lower abdominal | Change in bowel habit | 1.19 (0.58, 2.44) |
| Lower abdominal | Constipation | 0.68 (0.26, 1.74) |
| Lower abdominal | Diarrhoea | 1.70 (0.93, 3.10) |
| Lower abdominal | Distension | 1.36 (0.69, 2.66) |
| Lower abdominal | Lower abdominal pain | 1.36 (0.69, 2.66) |
| Lower abdominal | Pelvic pain | 0.00 (0.00, 0.65) |
| Lower abdominal | Rectal bleeding | 0.51 (0.17, 1.49) |
| Respiratory | Chest infection | 2.04 (1.17, 3.53) |
| Respiratory | Chest pain | 2.55 (1.55, 4.17) |
| Respiratory | Clubbing | 0.00 (0.00, 0.65) |
| Respiratory | Cough | 5.78 (4.17, 7.97) |
| Respiratory | Dyspnoea | 4.08 (2.76, 6.00) |
| Respiratory | Haemoptysis | 0.68 (0.26, 1.74) |
| Respiratory | Hoarseness | 0.34 (0.09, 1.23) |
| Respiratory | Sore throat | 0.68 (0.26, 1.74) |
| Respiratory | Stridor | 0.00 (0.00, 0.65) |
| Urological | Dysuria | 0.00 (0.00, 0.65) |
| Urological | Haematuria | 0.17 (0.03, 0.96) |
| Urological | Loin pain | 0.34 (0.09, 1.23) |
| Urological | LUTS (nocturia, frequency, hesitancy, urgency, retention) | 0.51 (0.17, 1.49) |
| Urological | Renal colic | 0.00 (0.00, 0.65) |
| Urological | Urinary tract infection | 0.00 (0.00, 0.65) |
| Central nervous system | Fit/seizure | 0.00 (0.00, 0.65) |
| Central nervous system | Headache | 0.68 (0.26, 1.74) |
| Central nervous system | Prog/sub-acute loss of central neuro funct | 0.68 (0.26, 1.74) |
| Central nervous system | Visual disturbance or loss | 0.51 (0.17, 1.49) |
| Musculoskeletal | Back pain | 2.55 (1.55, 4.17) |
| Musculoskeletal | Bone pain | 1.53 (0.81, 2.88) |
| Musculoskeletal | Fracture | 0.17 (0.03, 0.96) |
| Skin Lesion | Abnormal mole | 0.17 (0.03, 0.96) |
| Skin Lesion | Lesions suspicious of BCC | 1.02 (0.47, 2.21) |
| Skin Lesion | Non-pigmented lesion | 0.17 (0.03, 0.96) |
| Breast Symptoms | Axillary lump/mass | 0.68 (0.26, 1.74) |
| Breast Symptoms | Breast lump/mass | 0.00 (0.00, 0.65) |
| Breast Symptoms | Breast pain | 0.34 (0.09, 1.23) |
| Breast Symptoms | Nipple changes | 0.00 (0.00, 0.65) |
| Breast Symptoms | Nipple discharge | 0.00 (0.00, 0.65) |
| Female specific | Other vaginal bleeding | 0.00 (0.00, 0.65) |
| Female specific | Post-menopausal bleeding | 0.00 (0.00, 0.65) |
| Female specific | Vaginal discharge | 0.00 (0.00, 0.65) |
| Female specific | Vaginal mass | 0.00 (0.00, 0.65) |
| Female specific | Vulval bleeding | 0.00 (0.00, 0.65) |
| Female specific | Vulval mass | 0.00 (0.00, 0.65) |
| Female specific | Vulval ulceration | 0.17 (0.03, 0.96) |
| Male specific | Erectile dysfunction | 0.34 (0.09, 1.23) |
| Male specific | Penile ulceration | 0.00 (0.00, 0.65) |
| Male specific | Testicular lump | 0.00 (0.00, 0.65) |
| Male specific | Testicular pain | 0.00 (0.00, 0.65) |
| None recorded | N/A | 30.4 (26.9, 34.3) |
| None recorded | N/K | 8.33 (6.36, 10.8) |
| *Central nervous system - Central nervous system (N = 777)* | | |
| Non-specific | Bruising, bleeding or petechiae | 0.13 (0.02, 0.73) |
| Non-specific | Deep vein thrombosis | 0.00 (0.00, 0.49) |
| Non-specific | Epistaxis | 0.00 (0.00, 0.49) |
| Non-specific | Fatigue | 4.25 (3.04, 5.90) |
| Non-specific | Fever | 0.39 (0.13, 1.13) |
| Non-specific | Infection | 0.39 (0.13, 1.13) |
| Non-specific | Night sweats | 0.39 (0.13, 1.13) |
| Non-specific | Other symptom | 17.2 (14.8, 20.1) |
| Non-specific | Pallor | 0.13 (0.02, 0.73) |
| Non-specific | Pruritus | 0.13 (0.02, 0.73) |
| Non-specific | Weight loss | 1.54 (0.89, 2.68) |
| Lump/mass/lymph node | Anal mass | 0.00 (0.00, 0.49) |
| Lump/mass/lymph node | Lymph node pain with alcohol | 0.00 (0.00, 0.49) |
| Lump/mass/lymph node | Lymphadenopathy (generalised) | 0.00 (0.00, 0.49) |
| Lump/mass/lymph node | Lymphadenopathy (localised) | 0.00 (0.00, 0.49) |
| Lump/mass/lymph node | Neck lump/mass | 0.13 (0.02, 0.73) |
| Lump/mass/lymph node | Thyroid lump/mass | 0.00 (0.00, 0.49) |
| Lump/mass/lymph node | Unexplained lump suspicious of sarcoma | 0.00 (0.00, 0.49) |
| Ulceration | Leukoplakia | 0.00 (0.00, 0.49) |
| Ulceration | Lip/oral cavity/tongue lump/mass | 0.00 (0.00, 0.49) |
| Ulceration | Lip/oral cavity/tongue ulcer | 0.00 (0.00, 0.49) |
| Ulceration | Ulceration | 0.00 (0.00, 0.49) |
| Upper abdominal | Dyspepsia | 0.00 (0.00, 0.49) |
| Upper abdominal | Dysphagia | 0.00 (0.00, 0.49) |
| Upper abdominal | Early satiety | 0.00 (0.00, 0.49) |
| Upper abdominal | Gastroesophageal reflux | 0.00 (0.00, 0.49) |
| Upper abdominal | Haematemesis | 0.00 (0.00, 0.49) |
| Upper abdominal | Jaundice | 0.00 (0.00, 0.49) |
| Upper abdominal | Loss of appetite | 0.77 (0.35, 1.67) |
| Upper abdominal | Nausea and/or vomiting | 2.19 (1.37, 3.48) |
| Upper abdominal | New onset diabetes | 0.00 (0.00, 0.49) |
| Upper abdominal | Upper abdominal pain | 0.00 (0.00, 0.49) |
| Lower abdominal | Abdominal pain (NOS) | 0.13 (0.02, 0.73) |
| Lower abdominal | Change in bowel habit | 0.00 (0.00, 0.49) |
| Lower abdominal | Constipation | 0.26 (0.07, 0.93) |
| Lower abdominal | Diarrhoea | 0.00 (0.00, 0.49) |
| Lower abdominal | Distension | 0.00 (0.00, 0.49) |
| Lower abdominal | Lower abdominal pain | 0.00 (0.00, 0.49) |
| Lower abdominal | Pelvic pain | 0.26 (0.07, 0.93) |
| Lower abdominal | Rectal bleeding | 0.00 (0.00, 0.49) |
| Respiratory | Chest infection | 0.13 (0.02, 0.73) |
| Respiratory | Chest pain | 0.13 (0.02, 0.73) |
| Respiratory | Clubbing | 0.00 (0.00, 0.49) |
| Respiratory | Cough | 0.39 (0.13, 1.13) |
| Respiratory | Dyspnoea | 0.00 (0.00, 0.49) |
| Respiratory | Haemoptysis | 0.00 (0.00, 0.49) |
| Respiratory | Hoarseness | 0.13 (0.02, 0.73) |
| Respiratory | Sore throat | 0.13 (0.02, 0.73) |
| Respiratory | Stridor | 0.00 (0.00, 0.49) |
| Urological | Dysuria | 0.26 (0.07, 0.93) |
| Urological | Haematuria | 0.00 (0.00, 0.49) |
| Urological | Loin pain | 0.13 (0.02, 0.73) |
| Urological | LUTS (nocturia, frequency, hesitancy, urgency, retention) | 0.51 (0.20, 1.32) |
| Urological | Renal colic | 0.00 (0.00, 0.49) |
| Urological | Urinary tract infection | 0.13 (0.02, 0.73) |
| Central nervous system | Fit/seizure | 14.9 (12.6, 17.6) |
| Central nervous system | Headache | 19.2 (16.6, 22.1) |
| Central nervous system | Prog/sub-acute loss of central neuro funct | 31.0 (27.9, 34.4) |
| Central nervous system | Visual disturbance or loss | 6.18 (4.69, 8.10) |
| Musculoskeletal | Back pain | 1.67 (0.98, 2.84) |
| Musculoskeletal | Bone pain | 0.51 (0.20, 1.32) |
| Musculoskeletal | Fracture | 0.00 (0.00, 0.49) |
| Skin Lesion | Abnormal mole | 0.00 (0.00, 0.49) |
| Skin Lesion | Lesions suspicious of BCC | 0.00 (0.00, 0.49) |
| Skin Lesion | Non-pigmented lesion | 0.00 (0.00, 0.49) |
| Breast Symptoms | Axillary lump/mass | 0.00 (0.00, 0.49) |
| Breast Symptoms | Breast lump/mass | 0.00 (0.00, 0.49) |
| Breast Symptoms | Breast pain | 0.13 (0.02, 0.73) |
| Breast Symptoms | Nipple changes | 0.00 (0.00, 0.49) |
| Breast Symptoms | Nipple discharge | 0.13 (0.02, 0.73) |
| Female specific | Other vaginal bleeding | 0.00 (0.00, 0.49) |
| Female specific | Post-menopausal bleeding | 0.00 (0.00, 0.49) |
| Female specific | Vaginal discharge | 0.00 (0.00, 0.49) |
| Female specific | Vaginal mass | 0.00 (0.00, 0.49) |
| Female specific | Vulval bleeding | 0.00 (0.00, 0.49) |
| Female specific | Vulval mass | 0.00 (0.00, 0.49) |
| Female specific | Vulval ulceration | 0.00 (0.00, 0.49) |
| Male specific | Erectile dysfunction | 0.00 (0.00, 0.49) |
| Male specific | Penile ulceration | 0.00 (0.00, 0.49) |
| Male specific | Testicular lump | 0.00 (0.00, 0.49) |
| Male specific | Testicular pain | 0.00 (0.00, 0.49) |
| None recorded | N/A | 16.5 (14.0, 19.2) |
| None recorded | N/K | 4.89 (3.58, 6.64) |
| *Sarcoma - Bone sarcoma (N = 63)* | | |
| Non-specific | Bruising, bleeding or petechiae | 0.00 (0.00, 5.75) |
| Non-specific | Deep vein thrombosis | 0.00 (0.00, 5.75) |
| Non-specific | Epistaxis | 0.00 (0.00, 5.75) |
| Non-specific | Fatigue | 0.00 (0.00, 5.75) |
| Non-specific | Fever | 0.00 (0.00, 5.75) |
| Non-specific | Infection | 1.59 (0.28, 8.46) |
| Non-specific | Night sweats | 0.00 (0.00, 5.75) |
| Non-specific | Other symptom | 11.1 (5.49, 21.2) |
| Non-specific | Pallor | 0.00 (0.00, 5.75) |
| Non-specific | Pruritus | 0.00 (0.00, 5.75) |
| Non-specific | Weight loss | 1.59 (0.28, 8.46) |
| Lump/mass/lymph node | Anal mass | 0.00 (0.00, 5.75) |
| Lump/mass/lymph node | Lymph node pain with alcohol | 0.00 (0.00, 5.75) |
| Lump/mass/lymph node | Lymphadenopathy (generalised) | 0.00 (0.00, 5.75) |
| Lump/mass/lymph node | Lymphadenopathy (localised) | 1.59 (0.28, 8.46) |
| Lump/mass/lymph node | Neck lump/mass | 0.00 (0.00, 5.75) |
| Lump/mass/lymph node | Thyroid lump/mass | 0.00 (0.00, 5.75) |
| Lump/mass/lymph node | Unexplained lump suspicious of sarcoma | 11.1 (5.49, 21.2) |
| Ulceration | Leukoplakia | 0.00 (0.00, 5.75) |
| Ulceration | Lip/oral cavity/tongue lump/mass | 3.17 (0.87, 10.9) |
| Ulceration | Lip/oral cavity/tongue ulcer | 0.00 (0.00, 5.75) |
| Ulceration | Ulceration | 0.00 (0.00, 5.75) |
| Upper abdominal | Dyspepsia | 0.00 (0.00, 5.75) |
| Upper abdominal | Dysphagia | 0.00 (0.00, 5.75) |
| Upper abdominal | Early satiety | 1.59 (0.28, 8.46) |
| Upper abdominal | Gastroesophageal reflux | 0.00 (0.00, 5.75) |
| Upper abdominal | Haematemesis | 0.00 (0.00, 5.75) |
| Upper abdominal | Jaundice | 0.00 (0.00, 5.75) |
| Upper abdominal | Loss of appetite | 0.00 (0.00, 5.75) |
| Upper abdominal | Nausea and/or vomiting | 0.00 (0.00, 5.75) |
| Upper abdominal | New onset diabetes | 0.00 (0.00, 5.75) |
| Upper abdominal | Upper abdominal pain | 0.00 (0.00, 5.75) |
| Lower abdominal | Abdominal pain (NOS) | 0.00 (0.00, 5.75) |
| Lower abdominal | Change in bowel habit | 1.59 (0.28, 8.46) |
| Lower abdominal | Constipation | 0.00 (0.00, 5.75) |
| Lower abdominal | Diarrhoea | 0.00 (0.00, 5.75) |
| Lower abdominal | Distension | 0.00 (0.00, 5.75) |
| Lower abdominal | Lower abdominal pain | 0.00 (0.00, 5.75) |
| Lower abdominal | Pelvic pain | 3.17 (0.87, 10.9) |
| Lower abdominal | Rectal bleeding | 0.00 (0.00, 5.75) |
| Respiratory | Chest infection | 0.00 (0.00, 5.75) |
| Respiratory | Chest pain | 1.59 (0.28, 8.46) |
| Respiratory | Clubbing | 0.00 (0.00, 5.75) |
| Respiratory | Cough | 0.00 (0.00, 5.75) |
| Respiratory | Dyspnoea | 0.00 (0.00, 5.75) |
| Respiratory | Haemoptysis | 0.00 (0.00, 5.75) |
| Respiratory | Hoarseness | 0.00 (0.00, 5.75) |
| Respiratory | Sore throat | 0.00 (0.00, 5.75) |
| Respiratory | Stridor | 0.00 (0.00, 5.75) |
| Urological | Dysuria | 0.00 (0.00, 5.75) |
| Urological | Haematuria | 0.00 (0.00, 5.75) |
| Urological | Loin pain | 0.00 (0.00, 5.75) |
| Urological | LUTS (nocturia, frequency, hesitancy, urgency, retention) | 0.00 (0.00, 5.75) |
| Urological | Renal colic | 0.00 (0.00, 5.75) |
| Urological | Urinary tract infection | 0.00 (0.00, 5.75) |
| Central nervous system | Fit/seizure | 0.00 (0.00, 5.75) |
| Central nervous system | Headache | 1.59 (0.28, 8.46) |
| Central nervous system | Prog/sub-acute loss of central neuro funct | 1.59 (0.28, 8.46) |
| Central nervous system | Visual disturbance or loss | 0.00 (0.00, 5.75) |
| Musculoskeletal | Back pain | 19.0 (11.2, 30.4) |
| Musculoskeletal | Bone pain | 28.6 (18.9, 40.7) |
| Musculoskeletal | Fracture | 3.17 (0.87, 10.9) |
| Skin Lesion | Abnormal mole | 0.00 (0.00, 5.75) |
| Skin Lesion | Lesions suspicious of BCC | 0.00 (0.00, 5.75) |
| Skin Lesion | Non-pigmented lesion | 0.00 (0.00, 5.75) |
| Breast Symptoms | Axillary lump/mass | 0.00 (0.00, 5.75) |
| Breast Symptoms | Breast lump/mass | 0.00 (0.00, 5.75) |
| Breast Symptoms | Breast pain | 0.00 (0.00, 5.75) |
| Breast Symptoms | Nipple changes | 0.00 (0.00, 5.75) |
| Breast Symptoms | Nipple discharge | 0.00 (0.00, 5.75) |
| Female specific | Other vaginal bleeding | 0.00 (0.00, 5.75) |
| Female specific | Post-menopausal bleeding | 0.00 (0.00, 5.75) |
| Female specific | Vaginal discharge | 0.00 (0.00, 5.75) |
| Female specific | Vaginal mass | 0.00 (0.00, 5.75) |
| Female specific | Vulval bleeding | 0.00 (0.00, 5.75) |
| Female specific | Vulval mass | 0.00 (0.00, 5.75) |
| Female specific | Vulval ulceration | 0.00 (0.00, 5.75) |
| Male specific | Erectile dysfunction | 0.00 (0.00, 5.75) |
| Male specific | Penile ulceration | 0.00 (0.00, 5.75) |
| Male specific | Testicular lump | 0.00 (0.00, 5.75) |
| Male specific | Testicular pain | 0.00 (0.00, 5.75) |
| None recorded | N/A | 17.5 (10.0, 28.6) |
| None recorded | N/K | 3.17 (0.87, 10.9) |
| *Sarcoma - Connective and soft tissue sarcoma (N = 372)* | | |
| Non-specific | Bruising, bleeding or petechiae | 1.61 (0.74, 3.47) |
| Non-specific | Deep vein thrombosis | 0.54 (0.15, 1.94) |
| Non-specific | Epistaxis | 0.27 (0.05, 1.51) |
| Non-specific | Fatigue | 1.88 (0.91, 3.83) |
| Non-specific | Fever | 0.81 (0.27, 2.34) |
| Non-specific | Infection | 0.00 (0.00, 1.02) |
| Non-specific | Night sweats | 0.27 (0.05, 1.51) |
| Non-specific | Other symptom | 11.6 (8.70, 15.2) |
| Non-specific | Pallor | 0.00 (0.00, 1.02) |
| Non-specific | Pruritus | 0.81 (0.27, 2.34) |
| Non-specific | Weight loss | 2.96 (1.66, 5.22) |
| Lump/mass/lymph node | Anal mass | 0.54 (0.15, 1.94) |
| Lump/mass/lymph node | Lymph node pain with alcohol | 0.00 (0.00, 1.02) |
| Lump/mass/lymph node | Lymphadenopathy (generalised) | 0.00 (0.00, 1.02) |
| Lump/mass/lymph node | Lymphadenopathy (localised) | 0.54 (0.15, 1.94) |
| Lump/mass/lymph node | Neck lump/mass | 1.61 (0.74, 3.47) |
| Lump/mass/lymph node | Thyroid lump/mass | 0.00 (0.00, 1.02) |
| Lump/mass/lymph node | Unexplained lump suspicious of sarcoma | 28.8 (24.4, 33.6) |
| Ulceration | Leukoplakia | 0.00 (0.00, 1.02) |
| Ulceration | Lip/oral cavity/tongue lump/mass | 0.81 (0.27, 2.34) |
| Ulceration | Lip/oral cavity/tongue ulcer | 0.00 (0.00, 1.02) |
| Ulceration | Ulceration | 2.96 (1.66, 5.22) |
| Upper abdominal | Dyspepsia | 1.88 (0.91, 3.83) |
| Upper abdominal | Dysphagia | 0.27 (0.05, 1.51) |
| Upper abdominal | Early satiety | 0.27 (0.05, 1.51) |
| Upper abdominal | Gastroesophageal reflux | 0.27 (0.05, 1.51) |
| Upper abdominal | Haematemesis | 0.00 (0.00, 1.02) |
| Upper abdominal | Jaundice | 0.00 (0.00, 1.02) |
| Upper abdominal | Loss of appetite | 1.88 (0.91, 3.83) |
| Upper abdominal | Nausea and/or vomiting | 4.03 (2.46, 6.55) |
| Upper abdominal | New onset diabetes | 0.00 (0.00, 1.02) |
| Upper abdominal | Upper abdominal pain | 3.23 (1.85, 5.55) |
| Lower abdominal | Abdominal pain (NOS) | 8.33 (5.93, 11.6) |
| Lower abdominal | Change in bowel habit | 2.96 (1.66, 5.22) |
| Lower abdominal | Constipation | 1.34 (0.58, 3.11) |
| Lower abdominal | Diarrhoea | 1.34 (0.58, 3.11) |
| Lower abdominal | Distension | 8.06 (5.71, 11.3) |
| Lower abdominal | Lower abdominal pain | 3.49 (2.05, 5.89) |
| Lower abdominal | Pelvic pain | 1.34 (0.58, 3.11) |
| Lower abdominal | Rectal bleeding | 1.34 (0.58, 3.11) |
| Respiratory | Chest infection | 0.27 (0.05, 1.51) |
| Respiratory | Chest pain | 0.81 (0.27, 2.34) |
| Respiratory | Clubbing | 0.00 (0.00, 1.02) |
| Respiratory | Cough | 2.15 (1.09, 4.19) |
| Respiratory | Dyspnoea | 1.61 (0.74, 3.47) |
| Respiratory | Haemoptysis | 0.27 (0.05, 1.51) |
| Respiratory | Hoarseness | 0.00 (0.00, 1.02) |
| Respiratory | Sore throat | 0.27 (0.05, 1.51) |
| Respiratory | Stridor | 0.27 (0.05, 1.51) |
| Urological | Dysuria | 0.54 (0.15, 1.94) |
| Urological | Haematuria | 0.54 (0.15, 1.94) |
| Urological | Loin pain | 0.81 (0.27, 2.34) |
| Urological | LUTS (nocturia, frequency, hesitancy, urgency, retention) | 1.08 (0.42, 2.73) |
| Urological | Renal colic | 0.00 (0.00, 1.02) |
| Urological | Urinary tract infection | 0.54 (0.15, 1.94) |
| Central nervous system | Fit/seizure | 0.00 (0.00, 1.02) |
| Central nervous system | Headache | 0.27 (0.05, 1.51) |
| Central nervous system | Prog/sub-acute loss of central neuro funct | 0.27 (0.05, 1.51) |
| Central nervous system | Visual disturbance or loss | 0.00 (0.00, 1.02) |
| Musculoskeletal | Back pain | 2.15 (1.09, 4.19) |
| Musculoskeletal | Bone pain | 5.65 (3.72, 8.48) |
| Musculoskeletal | Fracture | 0.00 (0.00, 1.02) |
| Skin Lesion | Abnormal mole | 0.27 (0.05, 1.51) |
| Skin Lesion | Lesions suspicious of BCC | 2.96 (1.66, 5.22) |
| Skin Lesion | Non-pigmented lesion | 0.54 (0.15, 1.94) |
| Breast Symptoms | Axillary lump/mass | 0.54 (0.15, 1.94) |
| Breast Symptoms | Breast lump/mass | 1.08 (0.42, 2.73) |
| Breast Symptoms | Breast pain | 0.00 (0.00, 1.02) |
| Breast Symptoms | Nipple changes | 0.00 (0.00, 1.02) |
| Breast Symptoms | Nipple discharge | 0.00 (0.00, 1.02) |
| Female specific | Other vaginal bleeding | 0.00 (0.00, 1.02) |
| Female specific | Post-menopausal bleeding | 0.00 (0.00, 1.02) |
| Female specific | Vaginal discharge | 0.00 (0.00, 1.02) |
| Female specific | Vaginal mass | 0.27 (0.05, 1.51) |
| Female specific | Vulval bleeding | 0.00 (0.00, 1.02) |
| Female specific | Vulval mass | 0.00 (0.00, 1.02) |
| Female specific | Vulval ulceration | 0.00 (0.00, 1.02) |
| Male specific | Erectile dysfunction | 0.00 (0.00, 1.02) |
| Male specific | Penile ulceration | 0.00 (0.00, 1.02) |
| Male specific | Testicular lump | 0.27 (0.05, 1.51) |
| Male specific | Testicular pain | 0.00 (0.00, 1.02) |
| None recorded | N/A | 11.3 (8.46, 14.9) |
| None recorded | N/K | 2.69 (1.47, 4.88) |
| *Skin - Melanoma (N = 2790)* | | |
| Non-specific | Bruising, bleeding or petechiae | 0.61 (0.38, 0.97) |
| Non-specific | Deep vein thrombosis | 0.00 (0.00, 0.14) |
| Non-specific | Epistaxis | 0.00 (0.00, 0.14) |
| Non-specific | Fatigue | 0.18 (0.08, 0.42) |
| Non-specific | Fever | 0.04 (0.01, 0.20) |
| Non-specific | Infection | 0.22 (0.10, 0.47) |
| Non-specific | Night sweats | 0.04 (0.01, 0.20) |
| Non-specific | Other symptom | 3.15 (2.57, 3.87) |
| Non-specific | Pallor | 0.14 (0.06, 0.37) |
| Non-specific | Pruritus | 1.43 (1.05, 1.95) |
| Non-specific | Weight loss | 0.22 (0.10, 0.47) |
| Lump/mass/lymph node | Anal mass | 0.00 (0.00, 0.14) |
| Lump/mass/lymph node | Lymph node pain with alcohol | 0.00 (0.00, 0.14) |
| Lump/mass/lymph node | Lymphadenopathy (generalised) | 0.04 (0.01, 0.20) |
| Lump/mass/lymph node | Lymphadenopathy (localised) | 0.22 (0.10, 0.47) |
| Lump/mass/lymph node | Neck lump/mass | 0.39 (0.22, 0.70) |
| Lump/mass/lymph node | Thyroid lump/mass | 0.00 (0.00, 0.14) |
| Lump/mass/lymph node | Unexplained lump suspicious of sarcoma | 0.29 (0.15, 0.56) |
| Ulceration | Leukoplakia | 0.07 (0.02, 0.26) |
| Ulceration | Lip/oral cavity/tongue lump/mass | 0.04 (0.01, 0.20) |
| Ulceration | Lip/oral cavity/tongue ulcer | 0.11 (0.04, 0.32) |
| Ulceration | Ulceration | 3.05 (2.47, 3.75) |
| Upper abdominal | Dyspepsia | 0.07 (0.02, 0.26) |
| Upper abdominal | Dysphagia | 0.04 (0.01, 0.20) |
| Upper abdominal | Early satiety | 0.00 (0.00, 0.14) |
| Upper abdominal | Gastroesophageal reflux | 0.04 (0.01, 0.20) |
| Upper abdominal | Haematemesis | 0.04 (0.01, 0.20) |
| Upper abdominal | Jaundice | 0.04 (0.01, 0.20) |
| Upper abdominal | Loss of appetite | 0.11 (0.04, 0.32) |
| Upper abdominal | Nausea and/or vomiting | 0.18 (0.08, 0.42) |
| Upper abdominal | New onset diabetes | 0.00 (0.00, 0.14) |
| Upper abdominal | Upper abdominal pain | 0.04 (0.01, 0.20) |
| Lower abdominal | Abdominal pain (NOS) | 0.00 (0.00, 0.14) |
| Lower abdominal | Change in bowel habit | 0.00 (0.00, 0.14) |
| Lower abdominal | Constipation | 0.00 (0.00, 0.14) |
| Lower abdominal | Diarrhoea | 0.07 (0.02, 0.26) |
| Lower abdominal | Distension | 0.04 (0.01, 0.20) |
| Lower abdominal | Lower abdominal pain | 0.00 (0.00, 0.14) |
| Lower abdominal | Pelvic pain | 0.07 (0.02, 0.26) |
| Lower abdominal | Rectal bleeding | 0.00 (0.00, 0.14) |
| Respiratory | Chest infection | 0.04 (0.01, 0.20) |
| Respiratory | Chest pain | 0.22 (0.10, 0.47) |
| Respiratory | Clubbing | 0.00 (0.00, 0.14) |
| Respiratory | Cough | 0.36 (0.19, 0.66) |
| Respiratory | Dyspnoea | 0.07 (0.02, 0.26) |
| Respiratory | Haemoptysis | 0.00 (0.00, 0.14) |
| Respiratory | Hoarseness | 0.00 (0.00, 0.14) |
| Respiratory | Sore throat | 0.04 (0.01, 0.20) |
| Respiratory | Stridor | 0.00 (0.00, 0.14) |
| Urological | Dysuria | 0.00 (0.00, 0.14) |
| Urological | Haematuria | 0.00 (0.00, 0.14) |
| Urological | Loin pain | 0.00 (0.00, 0.14) |
| Urological | LUTS (nocturia, frequency, hesitancy, urgency, retention) | 0.07 (0.02, 0.26) |
| Urological | Renal colic | 0.00 (0.00, 0.14) |
| Urological | Urinary tract infection | 0.00 (0.00, 0.14) |
| Central nervous system | Fit/seizure | 0.04 (0.01, 0.20) |
| Central nervous system | Headache | 0.07 (0.02, 0.26) |
| Central nervous system | Prog/sub-acute loss of central neuro funct | 0.25 (0.12, 0.52) |
| Central nervous system | Visual disturbance or loss | 0.04 (0.01, 0.20) |
| Musculoskeletal | Back pain | 0.07 (0.02, 0.26) |
| Musculoskeletal | Bone pain | 0.14 (0.06, 0.37) |
| Musculoskeletal | Fracture | 0.00 (0.00, 0.14) |
| Skin Lesion | Abnormal mole | 70.9 (69.2, 72.6) |
| Skin Lesion | Lesions suspicious of BCC | 6.02 (5.20, 6.97) |
| Skin Lesion | Non-pigmented lesion | 7.17 (6.27, 8.19) |
| Breast Symptoms | Axillary lump/mass | 0.36 (0.19, 0.66) |
| Breast Symptoms | Breast lump/mass | 0.04 (0.01, 0.20) |
| Breast Symptoms | Breast pain | 0.00 (0.00, 0.14) |
| Breast Symptoms | Nipple changes | 0.04 (0.01, 0.20) |
| Breast Symptoms | Nipple discharge | 0.00 (0.00, 0.14) |
| Female specific | Other vaginal bleeding | 0.00 (0.00, 0.14) |
| Female specific | Post-menopausal bleeding | 0.00 (0.00, 0.14) |
| Female specific | Vaginal discharge | 0.00 (0.00, 0.14) |
| Female specific | Vaginal mass | 0.00 (0.00, 0.14) |
| Female specific | Vulval bleeding | 0.00 (0.00, 0.14) |
| Female specific | Vulval mass | 0.00 (0.00, 0.14) |
| Female specific | Vulval ulceration | 0.00 (0.00, 0.14) |
| Male specific | Erectile dysfunction | 0.00 (0.00, 0.14) |
| Male specific | Penile ulceration | 0.00 (0.00, 0.14) |
| Male specific | Testicular lump | 0.00 (0.00, 0.14) |
| Male specific | Testicular pain | 0.00 (0.00, 0.14) |
| None recorded | N/A | 6.77 (5.90, 7.77) |
| None recorded | N/K | 1.43 (1.05, 1.95) |
| *Ocular - Ocular (N = 111)* | | |
| Non-specific | Bruising, bleeding or petechiae | 0.00 (0.00, 3.35) |
| Non-specific | Deep vein thrombosis | 0.00 (0.00, 3.35) |
| Non-specific | Epistaxis | 0.00 (0.00, 3.35) |
| Non-specific | Fatigue | 0.00 (0.00, 3.35) |
| Non-specific | Fever | 0.00 (0.00, 3.35) |
| Non-specific | Infection | 0.00 (0.00, 3.35) |
| Non-specific | Night sweats | 0.00 (0.00, 3.35) |
| Non-specific | Other symptom | 14.4 (9.07, 22.1) |
| Non-specific | Pallor | 0.00 (0.00, 3.35) |
| Non-specific | Pruritus | 0.00 (0.00, 3.35) |
| Non-specific | Weight loss | 0.00 (0.00, 3.35) |
| Lump/mass/lymph node | Anal mass | 0.00 (0.00, 3.35) |
| Lump/mass/lymph node | Lymph node pain with alcohol | 0.00 (0.00, 3.35) |
| Lump/mass/lymph node | Lymphadenopathy (generalised) | 0.00 (0.00, 3.35) |
| Lump/mass/lymph node | Lymphadenopathy (localised) | 0.00 (0.00, 3.35) |
| Lump/mass/lymph node | Neck lump/mass | 0.00 (0.00, 3.35) |
| Lump/mass/lymph node | Thyroid lump/mass | 0.00 (0.00, 3.35) |
| Lump/mass/lymph node | Unexplained lump suspicious of sarcoma | 0.00 (0.00, 3.35) |
| Ulceration | Leukoplakia | 0.00 (0.00, 3.35) |
| Ulceration | Lip/oral cavity/tongue lump/mass | 0.00 (0.00, 3.35) |
| Ulceration | Lip/oral cavity/tongue ulcer | 0.00 (0.00, 3.35) |
| Ulceration | Ulceration | 0.00 (0.00, 3.35) |
| Upper abdominal | Dyspepsia | 0.00 (0.00, 3.35) |
| Upper abdominal | Dysphagia | 0.00 (0.00, 3.35) |
| Upper abdominal | Early satiety | 0.00 (0.00, 3.35) |
| Upper abdominal | Gastroesophageal reflux | 0.00 (0.00, 3.35) |
| Upper abdominal | Haematemesis | 0.00 (0.00, 3.35) |
| Upper abdominal | Jaundice | 0.00 (0.00, 3.35) |
| Upper abdominal | Loss of appetite | 0.00 (0.00, 3.35) |
| Upper abdominal | Nausea and/or vomiting | 0.00 (0.00, 3.35) |
| Upper abdominal | New onset diabetes | 0.00 (0.00, 3.35) |
| Upper abdominal | Upper abdominal pain | 0.00 (0.00, 3.35) |
| Lower abdominal | Abdominal pain (NOS) | 0.00 (0.00, 3.35) |
| Lower abdominal | Change in bowel habit | 0.00 (0.00, 3.35) |
| Lower abdominal | Constipation | 0.00 (0.00, 3.35) |
| Lower abdominal | Diarrhoea | 0.00 (0.00, 3.35) |
| Lower abdominal | Distension | 0.00 (0.00, 3.35) |
| Lower abdominal | Lower abdominal pain | 0.00 (0.00, 3.35) |
| Lower abdominal | Pelvic pain | 0.00 (0.00, 3.35) |
| Lower abdominal | Rectal bleeding | 0.00 (0.00, 3.35) |
| Respiratory | Chest infection | 0.00 (0.00, 3.35) |
| Respiratory | Chest pain | 0.00 (0.00, 3.35) |
| Respiratory | Clubbing | 0.00 (0.00, 3.35) |
| Respiratory | Cough | 0.00 (0.00, 3.35) |
| Respiratory | Dyspnoea | 0.00 (0.00, 3.35) |
| Respiratory | Haemoptysis | 0.00 (0.00, 3.35) |
| Respiratory | Hoarseness | 0.00 (0.00, 3.35) |
| Respiratory | Sore throat | 0.00 (0.00, 3.35) |
| Respiratory | Stridor | 0.00 (0.00, 3.35) |
| Urological | Dysuria | 0.00 (0.00, 3.35) |
| Urological | Haematuria | 0.00 (0.00, 3.35) |
| Urological | Loin pain | 0.00 (0.00, 3.35) |
| Urological | LUTS (nocturia, frequency, hesitancy, urgency, retention) | 0.90 (0.16, 4.93) |
| Urological | Renal colic | 0.00 (0.00, 3.35) |
| Urological | Urinary tract infection | 0.00 (0.00, 3.35) |
| Central nervous system | Fit/seizure | 0.00 (0.00, 3.35) |
| Central nervous system | Headache | 0.90 (0.16, 4.93) |
| Central nervous system | Prog/sub-acute loss of central neuro funct | 0.00 (0.00, 3.35) |
| Central nervous system | Visual disturbance or loss | 23.4 (16.5, 32.1) |
| Musculoskeletal | Back pain | 0.00 (0.00, 3.35) |
| Musculoskeletal | Bone pain | 0.00 (0.00, 3.35) |
| Musculoskeletal | Fracture | 0.00 (0.00, 3.35) |
| Skin Lesion | Abnormal mole | 0.90 (0.16, 4.93) |
| Skin Lesion | Lesions suspicious of BCC | 0.00 (0.00, 3.35) |
| Skin Lesion | Non-pigmented lesion | 0.00 (0.00, 3.35) |
| Breast Symptoms | Axillary lump/mass | 0.00 (0.00, 3.35) |
| Breast Symptoms | Breast lump/mass | 0.00 (0.00, 3.35) |
| Breast Symptoms | Breast pain | 0.00 (0.00, 3.35) |
| Breast Symptoms | Nipple changes | 0.00 (0.00, 3.35) |
| Breast Symptoms | Nipple discharge | 0.00 (0.00, 3.35) |
| Female specific | Other vaginal bleeding | 0.00 (0.00, 3.35) |
| Female specific | Post-menopausal bleeding | 0.00 (0.00, 3.35) |
| Female specific | Vaginal discharge | 0.00 (0.00, 3.35) |
| Female specific | Vaginal mass | 0.00 (0.00, 3.35) |
| Female specific | Vulval bleeding | 0.00 (0.00, 3.35) |
| Female specific | Vulval mass | 0.00 (0.00, 3.35) |
| Female specific | Vulval ulceration | 0.00 (0.00, 3.35) |
| Male specific | Erectile dysfunction | 0.00 (0.00, 3.35) |
| Male specific | Penile ulceration | 0.00 (0.00, 3.35) |
| Male specific | Testicular lump | 0.00 (0.00, 3.35) |
| Male specific | Testicular pain | 0.00 (0.00, 3.35) |
| None recorded | N/A | 46.8 (37.8, 56.1) |
| None recorded | N/K | 12.6 (7.66, 20.1) |
| *Breast - Breast (N = 6166)* | | |
| Non-specific | Bruising, bleeding or petechiae | 0.19 (0.11, 0.34) |
| Non-specific | Deep vein thrombosis | 0.03 (0.01, 0.12) |
| Non-specific | Epistaxis | 0.08 (0.03, 0.19) |
| Non-specific | Fatigue | 0.50 (0.35, 0.71) |
| Non-specific | Fever | 0.05 (0.02, 0.14) |
| Non-specific | Infection | 0.10 (0.04, 0.21) |
| Non-specific | Night sweats | 0.10 (0.04, 0.21) |
| Non-specific | Other symptom | 1.27 (1.01, 1.58) |
| Non-specific | Pallor | 0.05 (0.02, 0.14) |
| Non-specific | Pruritus | 0.26 (0.16, 0.42) |
| Non-specific | Weight loss | 1.38 (1.12, 1.70) |
| Lump/mass/lymph node | Anal mass | 0.02 (0.00, 0.09) |
| Lump/mass/lymph node | Lymph node pain with alcohol | 0.00 (0.00, 0.06) |
| Lump/mass/lymph node | Lymphadenopathy (generalised) | 0.03 (0.01, 0.12) |
| Lump/mass/lymph node | Lymphadenopathy (localised) | 0.21 (0.12, 0.36) |
| Lump/mass/lymph node | Neck lump/mass | 0.16 (0.09, 0.30) |
| Lump/mass/lymph node | Thyroid lump/mass | 0.00 (0.00, 0.06) |
| Lump/mass/lymph node | Unexplained lump suspicious of sarcoma | 0.02 (0.00, 0.09) |
| Ulceration | Leukoplakia | 0.00 (0.00, 0.06) |
| Ulceration | Lip/oral cavity/tongue lump/mass | 0.00 (0.00, 0.06) |
| Ulceration | Lip/oral cavity/tongue ulcer | 0.00 (0.00, 0.06) |
| Ulceration | Ulceration | 0.23 (0.14, 0.38) |
| Upper abdominal | Dyspepsia | 0.13 (0.07, 0.26) |
| Upper abdominal | Dysphagia | 0.05 (0.02, 0.14) |
| Upper abdominal | Early satiety | 0.05 (0.02, 0.14) |
| Upper abdominal | Gastroesophageal reflux | 0.05 (0.02, 0.14) |
| Upper abdominal | Haematemesis | 0.00 (0.00, 0.06) |
| Upper abdominal | Jaundice | 0.02 (0.00, 0.09) |
| Upper abdominal | Loss of appetite | 0.29 (0.18, 0.46) |
| Upper abdominal | Nausea and/or vomiting | 0.15 (0.08, 0.28) |
| Upper abdominal | New onset diabetes | 0.00 (0.00, 0.06) |
| Upper abdominal | Upper abdominal pain | 0.26 (0.16, 0.42) |
| Lower abdominal | Abdominal pain (NOS) | 0.18 (0.10, 0.32) |
| Lower abdominal | Change in bowel habit | 0.13 (0.07, 0.26) |
| Lower abdominal | Constipation | 0.08 (0.03, 0.19) |
| Lower abdominal | Diarrhoea | 0.16 (0.09, 0.30) |
| Lower abdominal | Distension | 0.11 (0.06, 0.23) |
| Lower abdominal | Lower abdominal pain | 0.08 (0.03, 0.19) |
| Lower abdominal | Pelvic pain | 0.06 (0.03, 0.17) |
| Lower abdominal | Rectal bleeding | 0.00 (0.00, 0.06) |
| Respiratory | Chest infection | 0.15 (0.08, 0.28) |
| Respiratory | Chest pain | 0.75 (0.56, 0.99) |
| Respiratory | Clubbing | 0.00 (0.00, 0.06) |
| Respiratory | Cough | 0.47 (0.33, 0.67) |
| Respiratory | Dyspnoea | 0.44 (0.30, 0.64) |
| Respiratory | Haemoptysis | 0.02 (0.00, 0.09) |
| Respiratory | Hoarseness | 0.02 (0.00, 0.09) |
| Respiratory | Sore throat | 0.00 (0.00, 0.06) |
| Respiratory | Stridor | 0.02 (0.00, 0.09) |
| Urological | Dysuria | 0.00 (0.00, 0.06) |
| Urological | Haematuria | 0.02 (0.00, 0.09) |
| Urological | Loin pain | 0.02 (0.00, 0.09) |
| Urological | LUTS (nocturia, frequency, hesitancy, urgency, retention) | 0.02 (0.00, 0.09) |
| Urological | Renal colic | 0.02 (0.00, 0.09) |
| Urological | Urinary tract infection | 0.03 (0.01, 0.12) |
| Central nervous system | Fit/seizure | 0.05 (0.02, 0.14) |
| Central nervous system | Headache | 0.06 (0.03, 0.17) |
| Central nervous system | Prog/sub-acute loss of central neuro funct | 0.08 (0.03, 0.19) |
| Central nervous system | Visual disturbance or loss | 0.02 (0.00, 0.09) |
| Musculoskeletal | Back pain | 0.94 (0.73, 1.21) |
| Musculoskeletal | Bone pain | 0.34 (0.22, 0.52) |
| Musculoskeletal | Fracture | 0.05 (0.02, 0.14) |
| Skin Lesion | Abnormal mole | 0.06 (0.03, 0.17) |
| Skin Lesion | Lesions suspicious of BCC | 0.00 (0.00, 0.06) |
| Skin Lesion | Non-pigmented lesion | 0.03 (0.01, 0.12) |
| Breast Symptoms | Axillary lump/mass | 1.54 (1.26, 1.88) |
| Breast Symptoms | Breast lump/mass | 75.0 (73.9, 76.1) |
| Breast Symptoms | Breast pain | 13.5 (12.7, 14.4) |
| Breast Symptoms | Nipple changes | 7.05 (6.44, 7.72) |
| Breast Symptoms | Nipple discharge | 2.81 (2.42, 3.25) |
| Female specific | Other vaginal bleeding | 0.06 (0.03, 0.17) |
| Female specific | Post-menopausal bleeding | 0.03 (0.01, 0.12) |
| Female specific | Vaginal discharge | 0.00 (0.00, 0.06) |
| Female specific | Vaginal mass | 0.03 (0.01, 0.12) |
| Female specific | Vulval bleeding | 0.02 (0.00, 0.09) |
| Female specific | Vulval mass | 0.00 (0.00, 0.06) |
| Female specific | Vulval ulceration | 0.00 (0.00, 0.06) |
| Male specific | Erectile dysfunction | 0.00 (0.00, 0.06) |
| Male specific | Penile ulceration | 0.00 (0.00, 0.06) |
| Male specific | Testicular lump | 0.00 (0.00, 0.06) |
| Male specific | Testicular pain | 0.00 (0.00, 0.06) |
| None recorded | N/A | 6.41 (5.82, 7.04) |
| None recorded | N/K | 1.78 (1.48, 2.15) |
| *Gynaecological - Cervix (N = 252)* | | |
| Non-specific | Bruising, bleeding or petechiae | 0.00 (0.00, 1.50) |
| Non-specific | Deep vein thrombosis | 0.79 (0.22, 2.85) |
| Non-specific | Epistaxis | 0.40 (0.07, 2.21) |
| Non-specific | Fatigue | 2.78 (1.35, 5.62) |
| Non-specific | Fever | 0.40 (0.07, 2.21) |
| Non-specific | Infection | 0.00 (0.00, 1.50) |
| Non-specific | Night sweats | 0.00 (0.00, 1.50) |
| Non-specific | Other symptom | 2.38 (1.10, 5.10) |
| Non-specific | Pallor | 0.00 (0.00, 1.50) |
| Non-specific | Pruritus | 0.00 (0.00, 1.50) |
| Non-specific | Weight loss | 2.78 (1.35, 5.62) |
| Lump/mass/lymph node | Anal mass | 0.00 (0.00, 1.50) |
| Lump/mass/lymph node | Lymph node pain with alcohol | 0.00 (0.00, 1.50) |
| Lump/mass/lymph node | Lymphadenopathy (generalised) | 0.00 (0.00, 1.50) |
| Lump/mass/lymph node | Lymphadenopathy (localised) | 0.40 (0.07, 2.21) |
| Lump/mass/lymph node | Neck lump/mass | 0.00 (0.00, 1.50) |
| Lump/mass/lymph node | Thyroid lump/mass | 0.00 (0.00, 1.50) |
| Lump/mass/lymph node | Unexplained lump suspicious of sarcoma | 0.00 (0.00, 1.50) |
| Ulceration | Leukoplakia | 0.00 (0.00, 1.50) |
| Ulceration | Lip/oral cavity/tongue lump/mass | 0.00 (0.00, 1.50) |
| Ulceration | Lip/oral cavity/tongue ulcer | 0.00 (0.00, 1.50) |
| Ulceration | Ulceration | 0.00 (0.00, 1.50) |
| Upper abdominal | Dyspepsia | 0.40 (0.07, 2.21) |
| Upper abdominal | Dysphagia | 0.00 (0.00, 1.50) |
| Upper abdominal | Early satiety | 0.00 (0.00, 1.50) |
| Upper abdominal | Gastroesophageal reflux | 0.00 (0.00, 1.50) |
| Upper abdominal | Haematemesis | 0.00 (0.00, 1.50) |
| Upper abdominal | Jaundice | 0.00 (0.00, 1.50) |
| Upper abdominal | Loss of appetite | 1.98 (0.85, 4.56) |
| Upper abdominal | Nausea and/or vomiting | 1.19 (0.41, 3.44) |
| Upper abdominal | New onset diabetes | 0.00 (0.00, 1.50) |
| Upper abdominal | Upper abdominal pain | 0.40 (0.07, 2.21) |
| Lower abdominal | Abdominal pain (NOS) | 2.78 (1.35, 5.62) |
| Lower abdominal | Change in bowel habit | 0.79 (0.22, 2.85) |
| Lower abdominal | Constipation | 2.38 (1.10, 5.10) |
| Lower abdominal | Diarrhoea | 1.19 (0.41, 3.44) |
| Lower abdominal | Distension | 0.79 (0.22, 2.85) |
| Lower abdominal | Lower abdominal pain | 7.54 (4.88, 11.5) |
| Lower abdominal | Pelvic pain | 5.16 (3.04, 8.62) |
| Lower abdominal | Rectal bleeding | 0.79 (0.22, 2.85) |
| Respiratory | Chest infection | 0.00 (0.00, 1.50) |
| Respiratory | Chest pain | 0.40 (0.07, 2.21) |
| Respiratory | Clubbing | 0.00 (0.00, 1.50) |
| Respiratory | Cough | 0.00 (0.00, 1.50) |
| Respiratory | Dyspnoea | 0.40 (0.07, 2.21) |
| Respiratory | Haemoptysis | 0.00 (0.00, 1.50) |
| Respiratory | Hoarseness | 0.00 (0.00, 1.50) |
| Respiratory | Sore throat | 0.00 (0.00, 1.50) |
| Respiratory | Stridor | 0.00 (0.00, 1.50) |
| Urological | Dysuria | 1.98 (0.85, 4.56) |
| Urological | Haematuria | 3.97 (2.17, 7.15) |
| Urological | Loin pain | 0.00 (0.00, 1.50) |
| Urological | LUTS (nocturia, frequency, hesitancy, urgency, retention) | 1.59 (0.62, 4.01) |
| Urological | Renal colic | 0.00 (0.00, 1.50) |
| Urological | Urinary tract infection | 2.78 (1.35, 5.62) |
| Central nervous system | Fit/seizure | 0.00 (0.00, 1.50) |
| Central nervous system | Headache | 0.00 (0.00, 1.50) |
| Central nervous system | Prog/sub-acute loss of central neuro funct | 0.00 (0.00, 1.50) |
| Central nervous system | Visual disturbance or loss | 0.00 (0.00, 1.50) |
| Musculoskeletal | Back pain | 3.17 (1.62, 6.14) |
| Musculoskeletal | Bone pain | 0.00 (0.00, 1.50) |
| Musculoskeletal | Fracture | 0.00 (0.00, 1.50) |
| Skin Lesion | Abnormal mole | 0.00 (0.00, 1.50) |
| Skin Lesion | Lesions suspicious of BCC | 0.00 (0.00, 1.50) |
| Skin Lesion | Non-pigmented lesion | 0.00 (0.00, 1.50) |
| Breast Symptoms | Axillary lump/mass | 0.00 (0.00, 1.50) |
| Breast Symptoms | Breast lump/mass | 0.00 (0.00, 1.50) |
| Breast Symptoms | Breast pain | 0.00 (0.00, 1.50) |
| Breast Symptoms | Nipple changes | 0.00 (0.00, 1.50) |
| Breast Symptoms | Nipple discharge | 0.00 (0.00, 1.50) |
| Female specific | Other vaginal bleeding | 34.5 (28.9, 40.6) |
| Female specific | Post-menopausal bleeding | 25.4 (20.4, 31.1) |
| Female specific | Vaginal discharge | 8.73 (5.84, 12.9) |
| Female specific | Vaginal mass | 2.78 (1.35, 5.62) |
| Female specific | Vulval bleeding | 3.17 (1.62, 6.14) |
| Female specific | Vulval mass | 0.79 (0.22, 2.85) |
| Female specific | Vulval ulceration | 0.00 (0.00, 1.50) |
| Male specific | Erectile dysfunction | 0.00 (0.00, 1.50) |
| Male specific | Penile ulceration | 0.00 (0.00, 1.50) |
| Male specific | Testicular lump | 0.00 (0.00, 1.50) |
| Male specific | Testicular pain | 0.00 (0.00, 1.50) |
| None recorded | N/A | 9.92 (6.81, 14.2) |
| None recorded | N/K | 3.17 (1.62, 6.14) |
| *Gynaecological - Ovary (N = 1174)* | | |
| Non-specific | Bruising, bleeding or petechiae | 0.17 (0.05, 0.62) |
| Non-specific | Deep vein thrombosis | 1.36 (0.84, 2.20) |
| Non-specific | Epistaxis | 0.00 (0.00, 0.33) |
| Non-specific | Fatigue | 3.66 (2.73, 4.90) |
| Non-specific | Fever | 0.60 (0.29, 1.23) |
| Non-specific | Infection | 0.26 (0.09, 0.75) |
| Non-specific | Night sweats | 0.77 (0.40, 1.45) |
| Non-specific | Other symptom | 4.68 (3.62, 6.05) |
| Non-specific | Pallor | 0.26 (0.09, 0.75) |
| Non-specific | Pruritus | 0.26 (0.09, 0.75) |
| Non-specific | Weight loss | 9.03 (7.52, 10.8) |
| Lump/mass/lymph node | Anal mass | 0.00 (0.00, 0.33) |
| Lump/mass/lymph node | Lymph node pain with alcohol | 0.00 (0.00, 0.33) |
| Lump/mass/lymph node | Lymphadenopathy (generalised) | 0.00 (0.00, 0.33) |
| Lump/mass/lymph node | Lymphadenopathy (localised) | 0.34 (0.13, 0.87) |
| Lump/mass/lymph node | Neck lump/mass | 0.26 (0.09, 0.75) |
| Lump/mass/lymph node | Thyroid lump/mass | 0.00 (0.00, 0.33) |
| Lump/mass/lymph node | Unexplained lump suspicious of sarcoma | 0.09 (0.02, 0.48) |
| Ulceration | Leukoplakia | 0.00 (0.00, 0.33) |
| Ulceration | Lip/oral cavity/tongue lump/mass | 0.00 (0.00, 0.33) |
| Ulceration | Lip/oral cavity/tongue ulcer | 0.09 (0.02, 0.48) |
| Ulceration | Ulceration | 0.09 (0.02, 0.48) |
| Upper abdominal | Dyspepsia | 2.47 (1.73, 3.53) |
| Upper abdominal | Dysphagia | 0.00 (0.00, 0.33) |
| Upper abdominal | Early satiety | 0.51 (0.23, 1.11) |
| Upper abdominal | Gastroesophageal reflux | 0.60 (0.29, 1.23) |
| Upper abdominal | Haematemesis | 0.09 (0.02, 0.48) |
| Upper abdominal | Jaundice | 0.17 (0.05, 0.62) |
| Upper abdominal | Loss of appetite | 5.54 (4.37, 7.00) |
| Upper abdominal | Nausea and/or vomiting | 5.37 (4.22, 6.81) |
| Upper abdominal | New onset diabetes | 0.00 (0.00, 0.33) |
| Upper abdominal | Upper abdominal pain | 3.66 (2.73, 4.90) |
| Lower abdominal | Abdominal pain (NOS) | 18.8 (16.7, 21.2) |
| Lower abdominal | Change in bowel habit | 5.96 (4.75, 7.47) |
| Lower abdominal | Constipation | 5.96 (4.75, 7.47) |
| Lower abdominal | Diarrhoea | 4.09 (3.10, 5.38) |
| Lower abdominal | Distension | 27.9 (25.4, 30.5) |
| Lower abdominal | Lower abdominal pain | 15.6 (13.6, 17.8) |
| Lower abdominal | Pelvic pain | 6.73 (5.43, 8.31) |
| Lower abdominal | Rectal bleeding | 1.45 (0.91, 2.31) |
| Respiratory | Chest infection | 0.60 (0.29, 1.23) |
| Respiratory | Chest pain | 1.19 (0.71, 1.99) |
| Respiratory | Clubbing | 0.00 (0.00, 0.33) |
| Respiratory | Cough | 1.11 (0.65, 1.89) |
| Respiratory | Dyspnoea | 3.41 (2.51, 4.61) |
| Respiratory | Haemoptysis | 0.00 (0.00, 0.33) |
| Respiratory | Hoarseness | 0.00 (0.00, 0.33) |
| Respiratory | Sore throat | 0.00 (0.00, 0.33) |
| Respiratory | Stridor | 0.00 (0.00, 0.33) |
| Urological | Dysuria | 2.30 (1.59, 3.33) |
| Urological | Haematuria | 0.85 (0.46, 1.56) |
| Urological | Loin pain | 1.02 (0.59, 1.78) |
| Urological | LUTS (nocturia, frequency, hesitancy, urgency, retention) | 5.71 (4.52, 7.18) |
| Urological | Renal colic | 0.51 (0.23, 1.11) |
| Urological | Urinary tract infection | 1.62 (1.04, 2.51) |
| Central nervous system | Fit/seizure | 0.00 (0.00, 0.33) |
| Central nervous system | Headache | 0.09 (0.02, 0.48) |
| Central nervous system | Prog/sub-acute loss of central neuro funct | 0.09 (0.02, 0.48) |
| Central nervous system | Visual disturbance or loss | 0.09 (0.02, 0.48) |
| Musculoskeletal | Back pain | 1.87 (1.24, 2.82) |
| Musculoskeletal | Bone pain | 0.17 (0.05, 0.62) |
| Musculoskeletal | Fracture | 0.00 (0.00, 0.33) |
| Skin Lesion | Abnormal mole | 0.00 (0.00, 0.33) |
| Skin Lesion | Lesions suspicious of BCC | 0.00 (0.00, 0.33) |
| Skin Lesion | Non-pigmented lesion | 0.00 (0.00, 0.33) |
| Breast Symptoms | Axillary lump/mass | 0.00 (0.00, 0.33) |
| Breast Symptoms | Breast lump/mass | 0.51 (0.23, 1.11) |
| Breast Symptoms | Breast pain | 0.09 (0.02, 0.48) |
| Breast Symptoms | Nipple changes | 0.00 (0.00, 0.33) |
| Breast Symptoms | Nipple discharge | 0.00 (0.00, 0.33) |
| Female specific | Other vaginal bleeding | 3.66 (2.73, 4.90) |
| Female specific | Post-menopausal bleeding | 5.28 (4.14, 6.71) |
| Female specific | Vaginal discharge | 1.70 (1.11, 2.62) |
| Female specific | Vaginal mass | 0.51 (0.23, 1.11) |
| Female specific | Vulval bleeding | 0.34 (0.13, 0.87) |
| Female specific | Vulval mass | 0.00 (0.00, 0.33) |
| Female specific | Vulval ulceration | 0.00 (0.00, 0.33) |
| Male specific | Erectile dysfunction | 0.00 (0.00, 0.33) |
| Male specific | Penile ulceration | 0.00 (0.00, 0.33) |
| Male specific | Testicular lump | 0.00 (0.00, 0.33) |
| Male specific | Testicular pain | 0.00 (0.00, 0.33) |
| None recorded | N/A | 10.1 (8.54, 12.0) |
| None recorded | N/K | 3.15 (2.30, 4.31) |
| *Gynaecological - Uterus (N = 1572)* | | |
| Non-specific | Bruising, bleeding or petechiae | 0.19 (0.06, 0.56) |
| Non-specific | Deep vein thrombosis | 0.06 (0.01, 0.36) |
| Non-specific | Epistaxis | 0.00 (0.00, 0.24) |
| Non-specific | Fatigue | 1.46 (0.98, 2.19) |
| Non-specific | Fever | 0.19 (0.06, 0.56) |
| Non-specific | Infection | 0.06 (0.01, 0.36) |
| Non-specific | Night sweats | 0.06 (0.01, 0.36) |
| Non-specific | Other symptom | 1.46 (0.98, 2.19) |
| Non-specific | Pallor | 0.13 (0.03, 0.46) |
| Non-specific | Pruritus | 0.06 (0.01, 0.36) |
| Non-specific | Weight loss | 2.04 (1.45, 2.86) |
| Lump/mass/lymph node | Anal mass | 0.00 (0.00, 0.24) |
| Lump/mass/lymph node | Lymph node pain with alcohol | 0.00 (0.00, 0.24) |
| Lump/mass/lymph node | Lymphadenopathy (generalised) | 0.06 (0.01, 0.36) |
| Lump/mass/lymph node | Lymphadenopathy (localised) | 0.00 (0.00, 0.24) |
| Lump/mass/lymph node | Neck lump/mass | 0.00 (0.00, 0.24) |
| Lump/mass/lymph node | Thyroid lump/mass | 0.06 (0.01, 0.36) |
| Lump/mass/lymph node | Unexplained lump suspicious of sarcoma | 0.00 (0.00, 0.24) |
| Ulceration | Leukoplakia | 0.00 (0.00, 0.24) |
| Ulceration | Lip/oral cavity/tongue lump/mass | 0.00 (0.00, 0.24) |
| Ulceration | Lip/oral cavity/tongue ulcer | 0.00 (0.00, 0.24) |
| Ulceration | Ulceration | 0.00 (0.00, 0.24) |
| Upper abdominal | Dyspepsia | 0.06 (0.01, 0.36) |
| Upper abdominal | Dysphagia | 0.06 (0.01, 0.36) |
| Upper abdominal | Early satiety | 0.06 (0.01, 0.36) |
| Upper abdominal | Gastroesophageal reflux | 0.06 (0.01, 0.36) |
| Upper abdominal | Haematemesis | 0.00 (0.00, 0.24) |
| Upper abdominal | Jaundice | 0.06 (0.01, 0.36) |
| Upper abdominal | Loss of appetite | 0.76 (0.44, 1.33) |
| Upper abdominal | Nausea and/or vomiting | 0.57 (0.30, 1.08) |
| Upper abdominal | New onset diabetes | 0.00 (0.00, 0.24) |
| Upper abdominal | Upper abdominal pain | 0.57 (0.30, 1.08) |
| Lower abdominal | Abdominal pain (NOS) | 3.50 (2.70, 4.53) |
| Lower abdominal | Change in bowel habit | 0.57 (0.30, 1.08) |
| Lower abdominal | Constipation | 0.38 (0.18, 0.83) |
| Lower abdominal | Diarrhoea | 0.38 (0.18, 0.83) |
| Lower abdominal | Distension | 1.84 (1.29, 2.64) |
| Lower abdominal | Lower abdominal pain | 3.56 (2.75, 4.60) |
| Lower abdominal | Pelvic pain | 3.18 (2.42, 4.17) |
| Lower abdominal | Rectal bleeding | 1.02 (0.63, 1.65) |
| Respiratory | Chest infection | 0.06 (0.01, 0.36) |
| Respiratory | Chest pain | 0.00 (0.00, 0.24) |
| Respiratory | Clubbing | 0.00 (0.00, 0.24) |
| Respiratory | Cough | 0.38 (0.18, 0.83) |
| Respiratory | Dyspnoea | 0.38 (0.18, 0.83) |
| Respiratory | Haemoptysis | 0.06 (0.01, 0.36) |
| Respiratory | Hoarseness | 0.00 (0.00, 0.24) |
| Respiratory | Sore throat | 0.00 (0.00, 0.24) |
| Respiratory | Stridor | 0.00 (0.00, 0.24) |
| Urological | Dysuria | 1.21 (0.78, 1.88) |
| Urological | Haematuria | 3.75 (2.92, 4.81) |
| Urological | Loin pain | 0.06 (0.01, 0.36) |
| Urological | LUTS (nocturia, frequency, hesitancy, urgency, retention) | 1.40 (0.93, 2.11) |
| Urological | Renal colic | 0.00 (0.00, 0.24) |
| Urological | Urinary tract infection | 1.15 (0.73, 1.80) |
| Central nervous system | Fit/seizure | 0.00 (0.00, 0.24) |
| Central nervous system | Headache | 0.06 (0.01, 0.36) |
| Central nervous system | Prog/sub-acute loss of central neuro funct | 0.06 (0.01, 0.36) |
| Central nervous system | Visual disturbance or loss | 0.00 (0.00, 0.24) |
| Musculoskeletal | Back pain | 1.65 (1.13, 2.41) |
| Musculoskeletal | Bone pain | 0.06 (0.01, 0.36) |
| Musculoskeletal | Fracture | 0.00 (0.00, 0.24) |
| Skin Lesion | Abnormal mole | 0.00 (0.00, 0.24) |
| Skin Lesion | Lesions suspicious of BCC | 0.00 (0.00, 0.24) |
| Skin Lesion | Non-pigmented lesion | 0.00 (0.00, 0.24) |
| Breast Symptoms | Axillary lump/mass | 0.00 (0.00, 0.24) |
| Breast Symptoms | Breast lump/mass | 0.13 (0.03, 0.46) |
| Breast Symptoms | Breast pain | 0.06 (0.01, 0.36) |
| Breast Symptoms | Nipple changes | 0.06 (0.01, 0.36) |
| Breast Symptoms | Nipple discharge | 0.06 (0.01, 0.36) |
| Female specific | Other vaginal bleeding | 19.2 (17.3, 21.2) |
| Female specific | Post-menopausal bleeding | 56.7 (54.2, 59.1) |
| Female specific | Vaginal discharge | 4.33 (3.43, 5.45) |
| Female specific | Vaginal mass | 0.70 (0.39, 1.25) |
| Female specific | Vulval bleeding | 1.78 (1.24, 2.56) |
| Female specific | Vulval mass | 0.13 (0.03, 0.46) |
| Female specific | Vulval ulceration | 0.06 (0.01, 0.36) |
| Male specific | Erectile dysfunction | 0.00 (0.00, 0.24) |
| Male specific | Penile ulceration | 0.00 (0.00, 0.24) |
| Male specific | Testicular lump | 0.00 (0.00, 0.24) |
| Male specific | Testicular pain | 0.00 (0.00, 0.24) |
| None recorded | N/A | 4.77 (3.82, 5.94) |
| None recorded | N/K | 2.04 (1.45, 2.86) |
| *Gynaecological - Vulva/Vagina (N = 241)* | | |
| Non-specific | Bruising, bleeding or petechiae | 0.00 (0.00, 1.57) |
| Non-specific | Deep vein thrombosis | 0.00 (0.00, 1.57) |
| Non-specific | Epistaxis | 0.00 (0.00, 1.57) |
| Non-specific | Fatigue | 0.41 (0.07, 2.31) |
| Non-specific | Fever | 0.41 (0.07, 2.31) |
| Non-specific | Infection | 0.41 (0.07, 2.31) |
| Non-specific | Night sweats | 0.00 (0.00, 1.57) |
| Non-specific | Other symptom | 4.56 (2.57, 7.99) |
| Non-specific | Pallor | 0.00 (0.00, 1.57) |
| Non-specific | Pruritus | 3.73 (1.98, 6.94) |
| Non-specific | Weight loss | 0.41 (0.07, 2.31) |
| Lump/mass/lymph node | Anal mass | 0.00 (0.00, 1.57) |
| Lump/mass/lymph node | Lymph node pain with alcohol | 0.00 (0.00, 1.57) |
| Lump/mass/lymph node | Lymphadenopathy (generalised) | 0.00 (0.00, 1.57) |
| Lump/mass/lymph node | Lymphadenopathy (localised) | 1.66 (0.65, 4.19) |
| Lump/mass/lymph node | Neck lump/mass | 0.00 (0.00, 1.57) |
| Lump/mass/lymph node | Thyroid lump/mass | 0.00 (0.00, 1.57) |
| Lump/mass/lymph node | Unexplained lump suspicious of sarcoma | 0.00 (0.00, 1.57) |
| Ulceration | Leukoplakia | 1.66 (0.65, 4.19) |
| Ulceration | Lip/oral cavity/tongue lump/mass | 0.00 (0.00, 1.57) |
| Ulceration | Lip/oral cavity/tongue ulcer | 0.00 (0.00, 1.57) |
| Ulceration | Ulceration | 0.00 (0.00, 1.57) |
| Upper abdominal | Dyspepsia | 0.00 (0.00, 1.57) |
| Upper abdominal | Dysphagia | 0.00 (0.00, 1.57) |
| Upper abdominal | Early satiety | 0.00 (0.00, 1.57) |
| Upper abdominal | Gastroesophageal reflux | 0.00 (0.00, 1.57) |
| Upper abdominal | Haematemesis | 0.00 (0.00, 1.57) |
| Upper abdominal | Jaundice | 0.00 (0.00, 1.57) |
| Upper abdominal | Loss of appetite | 0.83 (0.23, 2.97) |
| Upper abdominal | Nausea and/or vomiting | 0.41 (0.07, 2.31) |
| Upper abdominal | New onset diabetes | 0.00 (0.00, 1.57) |
| Upper abdominal | Upper abdominal pain | 0.00 (0.00, 1.57) |
| Lower abdominal | Abdominal pain (NOS) | 0.41 (0.07, 2.31) |
| Lower abdominal | Change in bowel habit | 0.41 (0.07, 2.31) |
| Lower abdominal | Constipation | 0.00 (0.00, 1.57) |
| Lower abdominal | Diarrhoea | 0.41 (0.07, 2.31) |
| Lower abdominal | Distension | 0.00 (0.00, 1.57) |
| Lower abdominal | Lower abdominal pain | 0.00 (0.00, 1.57) |
| Lower abdominal | Pelvic pain | 0.83 (0.23, 2.97) |
| Lower abdominal | Rectal bleeding | 0.41 (0.07, 2.31) |
| Respiratory | Chest infection | 0.00 (0.00, 1.57) |
| Respiratory | Chest pain | 0.00 (0.00, 1.57) |
| Respiratory | Clubbing | 0.00 (0.00, 1.57) |
| Respiratory | Cough | 0.41 (0.07, 2.31) |
| Respiratory | Dyspnoea | 0.00 (0.00, 1.57) |
| Respiratory | Haemoptysis | 0.00 (0.00, 1.57) |
| Respiratory | Hoarseness | 0.00 (0.00, 1.57) |
| Respiratory | Sore throat | 0.00 (0.00, 1.57) |
| Respiratory | Stridor | 0.00 (0.00, 1.57) |
| Urological | Dysuria | 3.73 (1.98, 6.94) |
| Urological | Haematuria | 1.24 (0.42, 3.60) |
| Urological | Loin pain | 0.00 (0.00, 1.57) |
| Urological | LUTS (nocturia, frequency, hesitancy, urgency, retention) | 2.49 (1.15, 5.32) |
| Urological | Renal colic | 0.00 (0.00, 1.57) |
| Urological | Urinary tract infection | 0.83 (0.23, 2.97) |
| Central nervous system | Fit/seizure | 0.00 (0.00, 1.57) |
| Central nervous system | Headache | 0.83 (0.23, 2.97) |
| Central nervous system | Prog/sub-acute loss of central neuro funct | 0.00 (0.00, 1.57) |
| Central nervous system | Visual disturbance or loss | 0.00 (0.00, 1.57) |
| Musculoskeletal | Back pain | 0.41 (0.07, 2.31) |
| Musculoskeletal | Bone pain | 0.00 (0.00, 1.57) |
| Musculoskeletal | Fracture | 0.00 (0.00, 1.57) |
| Skin Lesion | Abnormal mole | 0.41 (0.07, 2.31) |
| Skin Lesion | Lesions suspicious of BCC | 0.83 (0.23, 2.97) |
| Skin Lesion | Non-pigmented lesion | 0.00 (0.00, 1.57) |
| Breast Symptoms | Axillary lump/mass | 0.00 (0.00, 1.57) |
| Breast Symptoms | Breast lump/mass | 0.00 (0.00, 1.57) |
| Breast Symptoms | Breast pain | 0.00 (0.00, 1.57) |
| Breast Symptoms | Nipple changes | 0.00 (0.00, 1.57) |
| Breast Symptoms | Nipple discharge | 0.00 (0.00, 1.57) |
| Female specific | Other vaginal bleeding | 5.81 (3.49, 9.51) |
| Female specific | Post-menopausal bleeding | 4.98 (2.87, 8.50) |
| Female specific | Vaginal discharge | 2.07 (0.89, 4.76) |
| Female specific | Vaginal mass | 4.56 (2.57, 7.99) |
| Female specific | Vulval bleeding | 9.54 (6.44, 13.9) |
| Female specific | Vulval mass | 32.0 (26.4, 38.1) |
| Female specific | Vulval ulceration | 21.6 (16.9, 27.2) |
| Male specific | Erectile dysfunction | 0.00 (0.00, 1.57) |
| Male specific | Penile ulceration | 0.00 (0.00, 1.57) |
| Male specific | Testicular lump | 0.00 (0.00, 1.57) |
| Male specific | Testicular pain | 0.00 (0.00, 1.57) |
| None recorded | N/A | 12.0 (8.51, 16.7) |
| None recorded | N/K | 3.32 (1.69, 6.41) |
| *Prostate and other male organs - Penile (N = 119)* | | |
| Non-specific | Bruising, bleeding or petechiae | 3.36 (1.31, 8.32) |
| Non-specific | Deep vein thrombosis | 0.00 (0.00, 3.13) |
| Non-specific | Epistaxis | 0.00 (0.00, 3.13) |
| Non-specific | Fatigue | 0.00 (0.00, 3.13) |
| Non-specific | Fever | 0.00 (0.00, 3.13) |
| Non-specific | Infection | 0.00 (0.00, 3.13) |
| Non-specific | Night sweats | 0.00 (0.00, 3.13) |
| Non-specific | Other symptom | 18.5 (12.5, 26.4) |
| Non-specific | Pallor | 0.00 (0.00, 3.13) |
| Non-specific | Pruritus | 2.52 (0.86, 7.15) |
| Non-specific | Weight loss | 0.84 (0.15, 4.61) |
| Lump/mass/lymph node | Anal mass | 0.00 (0.00, 3.13) |
| Lump/mass/lymph node | Lymph node pain with alcohol | 0.00 (0.00, 3.13) |
| Lump/mass/lymph node | Lymphadenopathy (generalised) | 0.00 (0.00, 3.13) |
| Lump/mass/lymph node | Lymphadenopathy (localised) | 2.52 (0.86, 7.15) |
| Lump/mass/lymph node | Neck lump/mass | 0.00 (0.00, 3.13) |
| Lump/mass/lymph node | Thyroid lump/mass | 0.84 (0.15, 4.61) |
| Lump/mass/lymph node | Unexplained lump suspicious of sarcoma | 0.00 (0.00, 3.13) |
| Ulceration | Leukoplakia | 0.84 (0.15, 4.61) |
| Ulceration | Lip/oral cavity/tongue lump/mass | 0.00 (0.00, 3.13) |
| Ulceration | Lip/oral cavity/tongue ulcer | 0.84 (0.15, 4.61) |
| Ulceration | Ulceration | 3.36 (1.31, 8.32) |
| Upper abdominal | Dyspepsia | 0.00 (0.00, 3.13) |
| Upper abdominal | Dysphagia | 0.00 (0.00, 3.13) |
| Upper abdominal | Early satiety | 0.00 (0.00, 3.13) |
| Upper abdominal | Gastroesophageal reflux | 0.00 (0.00, 3.13) |
| Upper abdominal | Haematemesis | 0.00 (0.00, 3.13) |
| Upper abdominal | Jaundice | 0.00 (0.00, 3.13) |
| Upper abdominal | Loss of appetite | 0.00 (0.00, 3.13) |
| Upper abdominal | Nausea and/or vomiting | 0.00 (0.00, 3.13) |
| Upper abdominal | New onset diabetes | 0.00 (0.00, 3.13) |
| Upper abdominal | Upper abdominal pain | 0.00 (0.00, 3.13) |
| Lower abdominal | Abdominal pain (NOS) | 0.00 (0.00, 3.13) |
| Lower abdominal | Change in bowel habit | 0.00 (0.00, 3.13) |
| Lower abdominal | Constipation | 0.00 (0.00, 3.13) |
| Lower abdominal | Diarrhoea | 0.00 (0.00, 3.13) |
| Lower abdominal | Distension | 0.00 (0.00, 3.13) |
| Lower abdominal | Lower abdominal pain | 0.00 (0.00, 3.13) |
| Lower abdominal | Pelvic pain | 0.00 (0.00, 3.13) |
| Lower abdominal | Rectal bleeding | 0.00 (0.00, 3.13) |
| Respiratory | Chest infection | 0.00 (0.00, 3.13) |
| Respiratory | Chest pain | 0.00 (0.00, 3.13) |
| Respiratory | Clubbing | 0.84 (0.15, 4.61) |
| Respiratory | Cough | 0.00 (0.00, 3.13) |
| Respiratory | Dyspnoea | 0.00 (0.00, 3.13) |
| Respiratory | Haemoptysis | 0.00 (0.00, 3.13) |
| Respiratory | Hoarseness | 0.00 (0.00, 3.13) |
| Respiratory | Sore throat | 0.00 (0.00, 3.13) |
| Respiratory | Stridor | 0.00 (0.00, 3.13) |
| Urological | Dysuria | 5.04 (2.33, 10.6) |
| Urological | Haematuria | 3.36 (1.31, 8.32) |
| Urological | Loin pain | 1.68 (0.46, 5.92) |
| Urological | LUTS (nocturia, frequency, hesitancy, urgency, retention) | 8.40 (4.63, 14.8) |
| Urological | Renal colic | 0.00 (0.00, 3.13) |
| Urological | Urinary tract infection | 1.68 (0.46, 5.92) |
| Central nervous system | Fit/seizure | 0.00 (0.00, 3.13) |
| Central nervous system | Headache | 0.00 (0.00, 3.13) |
| Central nervous system | Prog/sub-acute loss of central neuro funct | 0.00 (0.00, 3.13) |
| Central nervous system | Visual disturbance or loss | 0.00 (0.00, 3.13) |
| Musculoskeletal | Back pain | 0.00 (0.00, 3.13) |
| Musculoskeletal | Bone pain | 0.00 (0.00, 3.13) |
| Musculoskeletal | Fracture | 0.00 (0.00, 3.13) |
| Skin Lesion | Abnormal mole | 0.00 (0.00, 3.13) |
| Skin Lesion | Lesions suspicious of BCC | 0.84 (0.15, 4.61) |
| Skin Lesion | Non-pigmented lesion | 0.00 (0.00, 3.13) |
| Breast Symptoms | Axillary lump/mass | 0.00 (0.00, 3.13) |
| Breast Symptoms | Breast lump/mass | 0.00 (0.00, 3.13) |
| Breast Symptoms | Breast pain | 0.00 (0.00, 3.13) |
| Breast Symptoms | Nipple changes | 0.00 (0.00, 3.13) |
| Breast Symptoms | Nipple discharge | 0.00 (0.00, 3.13) |
| Female specific | Other vaginal bleeding | 0.00 (0.00, 3.13) |
| Female specific | Post-menopausal bleeding | 0.00 (0.00, 3.13) |
| Female specific | Vaginal discharge | 0.00 (0.00, 3.13) |
| Female specific | Vaginal mass | 0.00 (0.00, 3.13) |
| Female specific | Vulval bleeding | 0.00 (0.00, 3.13) |
| Female specific | Vulval mass | 0.00 (0.00, 3.13) |
| Female specific | Vulval ulceration | 0.00 (0.00, 3.13) |
| Male specific | Erectile dysfunction | 0.84 (0.15, 4.61) |
| Male specific | Penile ulceration | 44.5 (35.9, 53.5) |
| Male specific | Testicular lump | 0.84 (0.15, 4.61) |
| Male specific | Testicular pain | 0.00 (0.00, 3.13) |
| None recorded | N/A | 10.9 (6.50, 17.8) |
| None recorded | N/K | 2.52 (0.86, 7.15) |
| *Prostate and other male organs - Prostate (N = 8747)* | | |
| Non-specific | Bruising, bleeding or petechiae | 0.14 (0.08, 0.24) |
| Non-specific | Deep vein thrombosis | 0.18 (0.11, 0.30) |
| Non-specific | Epistaxis | 0.02 (0.01, 0.08) |
| Non-specific | Fatigue | 1.83 (1.57, 2.13) |
| Non-specific | Fever | 0.22 (0.14, 0.34) |
| Non-specific | Infection | 0.13 (0.07, 0.23) |
| Non-specific | Night sweats | 0.48 (0.36, 0.65) |
| Non-specific | Other symptom | 4.13 (3.73, 4.56) |
| Non-specific | Pallor | 0.11 (0.06, 0.21) |
| Non-specific | Pruritus | 0.05 (0.02, 0.12) |
| Non-specific | Weight loss | 3.19 (2.84, 3.58) |
| Lump/mass/lymph node | Anal mass | 0.02 (0.01, 0.08) |
| Lump/mass/lymph node | Lymph node pain with alcohol | 0.00 (0.00, 0.04) |
| Lump/mass/lymph node | Lymphadenopathy (generalised) | 0.00 (0.00, 0.04) |
| Lump/mass/lymph node | Lymphadenopathy (localised) | 0.07 (0.03, 0.15) |
| Lump/mass/lymph node | Neck lump/mass | 0.05 (0.02, 0.12) |
| Lump/mass/lymph node | Thyroid lump/mass | 0.00 (0.00, 0.04) |
| Lump/mass/lymph node | Unexplained lump suspicious of sarcoma | 0.01 (0.00, 0.06) |
| Ulceration | Leukoplakia | 0.00 (0.00, 0.04) |
| Ulceration | Lip/oral cavity/tongue lump/mass | 0.02 (0.01, 0.08) |
| Ulceration | Lip/oral cavity/tongue ulcer | 0.00 (0.00, 0.04) |
| Ulceration | Ulceration | 0.01 (0.00, 0.06) |
| Upper abdominal | Dyspepsia | 0.18 (0.11, 0.30) |
| Upper abdominal | Dysphagia | 0.05 (0.02, 0.12) |
| Upper abdominal | Early satiety | 0.03 (0.01, 0.10) |
| Upper abdominal | Gastroesophageal reflux | 0.07 (0.03, 0.15) |
| Upper abdominal | Haematemesis | 0.01 (0.00, 0.06) |
| Upper abdominal | Jaundice | 0.05 (0.02, 0.12) |
| Upper abdominal | Loss of appetite | 0.82 (0.65, 1.04) |
| Upper abdominal | Nausea and/or vomiting | 0.27 (0.18, 0.41) |
| Upper abdominal | New onset diabetes | 0.01 (0.00, 0.06) |
| Upper abdominal | Upper abdominal pain | 0.33 (0.23, 0.48) |
| Lower abdominal | Abdominal pain (NOS) | 1.12 (0.92, 1.36) |
| Lower abdominal | Change in bowel habit | 0.67 (0.52, 0.87) |
| Lower abdominal | Constipation | 0.78 (0.61, 0.98) |
| Lower abdominal | Diarrhoea | 0.42 (0.31, 0.58) |
| Lower abdominal | Distension | 0.59 (0.45, 0.78) |
| Lower abdominal | Lower abdominal pain | 1.29 (1.08, 1.55) |
| Lower abdominal | Pelvic pain | 1.01 (0.82, 1.24) |
| Lower abdominal | Rectal bleeding | 0.46 (0.34, 0.62) |
| Respiratory | Chest infection | 0.14 (0.08, 0.24) |
| Respiratory | Chest pain | 0.37 (0.26, 0.52) |
| Respiratory | Clubbing | 0.03 (0.01, 0.10) |
| Respiratory | Cough | 0.37 (0.26, 0.52) |
| Respiratory | Dyspnoea | 0.40 (0.29, 0.56) |
| Respiratory | Haemoptysis | 0.02 (0.01, 0.08) |
| Respiratory | Hoarseness | 0.01 (0.00, 0.06) |
| Respiratory | Sore throat | 0.00 (0.00, 0.04) |
| Respiratory | Stridor | 0.00 (0.00, 0.04) |
| Urological | Dysuria | 3.35 (2.99, 3.75) |
| Urological | Haematuria | 4.38 (3.97, 4.83) |
| Urological | Loin pain | 0.79 (0.62, 1.00) |
| Urological | LUTS (nocturia, frequency, hesitancy, urgency, retention) | 46.2 (45.2, 47.3) |
| Urological | Renal colic | 0.18 (0.11, 0.30) |
| Urological | Urinary tract infection | 2.84 (2.51, 3.20) |
| Central nervous system | Fit/seizure | 0.00 (0.00, 0.04) |
| Central nervous system | Headache | 0.10 (0.05, 0.20) |
| Central nervous system | Prog/sub-acute loss of central neuro funct | 0.03 (0.01, 0.10) |
| Central nervous system | Visual disturbance or loss | 0.06 (0.02, 0.13) |
| Musculoskeletal | Back pain | 4.68 (4.25, 5.14) |
| Musculoskeletal | Bone pain | 1.15 (0.95, 1.40) |
| Musculoskeletal | Fracture | 0.07 (0.03, 0.15) |
| Skin Lesion | Abnormal mole | 0.01 (0.00, 0.06) |
| Skin Lesion | Lesions suspicious of BCC | 0.00 (0.00, 0.04) |
| Skin Lesion | Non-pigmented lesion | 0.00 (0.00, 0.04) |
| Breast Symptoms | Axillary lump/mass | 0.02 (0.01, 0.08) |
| Breast Symptoms | Breast lump/mass | 0.03 (0.01, 0.10) |
| Breast Symptoms | Breast pain | 0.02 (0.01, 0.08) |
| Breast Symptoms | Nipple changes | 0.00 (0.00, 0.04) |
| Breast Symptoms | Nipple discharge | 0.00 (0.00, 0.04) |
| Female specific | Other vaginal bleeding | 0.00 (0.00, 0.04) |
| Female specific | Post-menopausal bleeding | 0.00 (0.00, 0.04) |
| Female specific | Vaginal discharge | 0.00 (0.00, 0.04) |
| Female specific | Vaginal mass | 0.00 (0.00, 0.04) |
| Female specific | Vulval bleeding | 0.00 (0.00, 0.04) |
| Female specific | Vulval mass | 0.00 (0.00, 0.04) |
| Female specific | Vulval ulceration | 0.00 (0.00, 0.04) |
| Male specific | Erectile dysfunction | 3.05 (2.71, 3.43) |
| Male specific | Penile ulceration | 0.01 (0.00, 0.06) |
| Male specific | Testicular lump | 0.27 (0.18, 0.41) |
| Male specific | Testicular pain | 0.69 (0.53, 0.88) |
| None recorded | N/A | 23.4 (22.5, 24.3) |
| None recorded | N/K | 6.48 (5.99, 7.02) |
| *Prostate and other male organs - Testicular (N = 403)* | | |
| Non-specific | Bruising, bleeding or petechiae | 0.00 (0.00, 0.94) |
| Non-specific | Deep vein thrombosis | 0.50 (0.14, 1.79) |
| Non-specific | Epistaxis | 0.00 (0.00, 0.94) |
| Non-specific | Fatigue | 0.00 (0.00, 0.94) |
| Non-specific | Fever | 0.00 (0.00, 0.94) |
| Non-specific | Infection | 0.00 (0.00, 0.94) |
| Non-specific | Night sweats | 0.25 (0.04, 1.39) |
| Non-specific | Other symptom | 2.48 (1.35, 4.51) |
| Non-specific | Pallor | 0.25 (0.04, 1.39) |
| Non-specific | Pruritus | 0.00 (0.00, 0.94) |
| Non-specific | Weight loss | 1.24 (0.53, 2.87) |
| Lump/mass/lymph node | Anal mass | 0.00 (0.00, 0.94) |
| Lump/mass/lymph node | Lymph node pain with alcohol | 0.00 (0.00, 0.94) |
| Lump/mass/lymph node | Lymphadenopathy (generalised) | 0.00 (0.00, 0.94) |
| Lump/mass/lymph node | Lymphadenopathy (localised) | 0.25 (0.04, 1.39) |
| Lump/mass/lymph node | Neck lump/mass | 0.25 (0.04, 1.39) |
| Lump/mass/lymph node | Thyroid lump/mass | 0.00 (0.00, 0.94) |
| Lump/mass/lymph node | Unexplained lump suspicious of sarcoma | 0.00 (0.00, 0.94) |
| Ulceration | Leukoplakia | 0.00 (0.00, 0.94) |
| Ulceration | Lip/oral cavity/tongue lump/mass | 0.00 (0.00, 0.94) |
| Ulceration | Lip/oral cavity/tongue ulcer | 0.00 (0.00, 0.94) |
| Ulceration | Ulceration | 0.50 (0.14, 1.79) |
| Upper abdominal | Dyspepsia | 0.00 (0.00, 0.94) |
| Upper abdominal | Dysphagia | 0.00 (0.00, 0.94) |
| Upper abdominal | Early satiety | 0.00 (0.00, 0.94) |
| Upper abdominal | Gastroesophageal reflux | 0.00 (0.00, 0.94) |
| Upper abdominal | Haematemesis | 0.00 (0.00, 0.94) |
| Upper abdominal | Jaundice | 0.00 (0.00, 0.94) |
| Upper abdominal | Loss of appetite | 0.50 (0.14, 1.79) |
| Upper abdominal | Nausea and/or vomiting | 0.25 (0.04, 1.39) |
| Upper abdominal | New onset diabetes | 0.00 (0.00, 0.94) |
| Upper abdominal | Upper abdominal pain | 0.25 (0.04, 1.39) |
| Lower abdominal | Abdominal pain (NOS) | 1.99 (1.01, 3.87) |
| Lower abdominal | Change in bowel habit | 0.25 (0.04, 1.39) |
| Lower abdominal | Constipation | 0.00 (0.00, 0.94) |
| Lower abdominal | Diarrhoea | 0.25 (0.04, 1.39) |
| Lower abdominal | Distension | 0.99 (0.39, 2.52) |
| Lower abdominal | Lower abdominal pain | 0.99 (0.39, 2.52) |
| Lower abdominal | Pelvic pain | 0.74 (0.25, 2.17) |
| Lower abdominal | Rectal bleeding | 0.25 (0.04, 1.39) |
| Respiratory | Chest infection | 0.25 (0.04, 1.39) |
| Respiratory | Chest pain | 0.25 (0.04, 1.39) |
| Respiratory | Clubbing | 0.00 (0.00, 0.94) |
| Respiratory | Cough | 0.25 (0.04, 1.39) |
| Respiratory | Dyspnoea | 0.25 (0.04, 1.39) |
| Respiratory | Haemoptysis | 0.25 (0.04, 1.39) |
| Respiratory | Hoarseness | 0.00 (0.00, 0.94) |
| Respiratory | Sore throat | 0.00 (0.00, 0.94) |
| Respiratory | Stridor | 0.00 (0.00, 0.94) |
| Urological | Dysuria | 0.00 (0.00, 0.94) |
| Urological | Haematuria | 0.00 (0.00, 0.94) |
| Urological | Loin pain | 0.74 (0.25, 2.17) |
| Urological | LUTS (nocturia, frequency, hesitancy, urgency, retention) | 1.49 (0.68, 3.21) |
| Urological | Renal colic | 0.00 (0.00, 0.94) |
| Urological | Urinary tract infection | 0.25 (0.04, 1.39) |
| Central nervous system | Fit/seizure | 0.25 (0.04, 1.39) |
| Central nervous system | Headache | 0.00 (0.00, 0.94) |
| Central nervous system | Prog/sub-acute loss of central neuro funct | 0.25 (0.04, 1.39) |
| Central nervous system | Visual disturbance or loss | 0.00 (0.00, 0.94) |
| Musculoskeletal | Back pain | 0.99 (0.39, 2.52) |
| Musculoskeletal | Bone pain | 0.25 (0.04, 1.39) |
| Musculoskeletal | Fracture | 0.00 (0.00, 0.94) |
| Skin Lesion | Abnormal mole | 0.00 (0.00, 0.94) |
| Skin Lesion | Lesions suspicious of BCC | 0.00 (0.00, 0.94) |
| Skin Lesion | Non-pigmented lesion | 0.00 (0.00, 0.94) |
| Breast Symptoms | Axillary lump/mass | 0.00 (0.00, 0.94) |
| Breast Symptoms | Breast lump/mass | 0.25 (0.04, 1.39) |
| Breast Symptoms | Breast pain | 0.00 (0.00, 0.94) |
| Breast Symptoms | Nipple changes | 0.00 (0.00, 0.94) |
| Breast Symptoms | Nipple discharge | 0.00 (0.00, 0.94) |
| Female specific | Other vaginal bleeding | 0.00 (0.00, 0.94) |
| Female specific | Post-menopausal bleeding | 0.00 (0.00, 0.94) |
| Female specific | Vaginal discharge | 0.00 (0.00, 0.94) |
| Female specific | Vaginal mass | 0.00 (0.00, 0.94) |
| Female specific | Vulval bleeding | 0.00 (0.00, 0.94) |
| Female specific | Vulval mass | 0.00 (0.00, 0.94) |
| Female specific | Vulval ulceration | 0.00 (0.00, 0.94) |
| Male specific | Erectile dysfunction | 0.00 (0.00, 0.94) |
| Male specific | Penile ulceration | 0.00 (0.00, 0.94) |
| Male specific | Testicular lump | 63.8 (59.0, 68.3) |
| Male specific | Testicular pain | 31.5 (27.2, 36.2) |
| None recorded | N/A | 6.95 (4.85, 9.86) |
| None recorded | N/K | 2.23 (1.18, 4.19) |
| *Other malignant neoplasms - Other malignant neoplasms (N = 190)* | | |
| Non-specific | Bruising, bleeding or petechiae | 0.00 (0.00, 1.98) |
| Non-specific | Deep vein thrombosis | 0.00 (0.00, 1.98) |
| Non-specific | Epistaxis | 0.00 (0.00, 1.98) |
| Non-specific | Fatigue | 6.32 (3.65, 10.7) |
| Non-specific | Fever | 1.05 (0.29, 3.76) |
| Non-specific | Infection | 0.00 (0.00, 1.98) |
| Non-specific | Night sweats | 0.53 (0.09, 2.92) |
| Non-specific | Other symptom | 9.47 (6.08, 14.5) |
| Non-specific | Pallor | 0.53 (0.09, 2.92) |
| Non-specific | Pruritus | 2.11 (0.82, 5.29) |
| Non-specific | Weight loss | 10.0 (6.50, 15.1) |
| Lump/mass/lymph node | Anal mass | 0.00 (0.00, 1.98) |
| Lump/mass/lymph node | Lymph node pain with alcohol | 0.00 (0.00, 1.98) |
| Lump/mass/lymph node | Lymphadenopathy (generalised) | 0.53 (0.09, 2.92) |
| Lump/mass/lymph node | Lymphadenopathy (localised) | 0.00 (0.00, 1.98) |
| Lump/mass/lymph node | Neck lump/mass | 2.11 (0.82, 5.29) |
| Lump/mass/lymph node | Thyroid lump/mass | 0.00 (0.00, 1.98) |
| Lump/mass/lymph node | Unexplained lump suspicious of sarcoma | 2.11 (0.82, 5.29) |
| Ulceration | Leukoplakia | 0.00 (0.00, 1.98) |
| Ulceration | Lip/oral cavity/tongue lump/mass | 0.53 (0.09, 2.92) |
| Ulceration | Lip/oral cavity/tongue ulcer | 0.53 (0.09, 2.92) |
| Ulceration | Ulceration | 0.00 (0.00, 1.98) |
| Upper abdominal | Dyspepsia | 1.58 (0.54, 4.54) |
| Upper abdominal | Dysphagia | 0.53 (0.09, 2.92) |
| Upper abdominal | Early satiety | 0.53 (0.09, 2.92) |
| Upper abdominal | Gastroesophageal reflux | 0.00 (0.00, 1.98) |
| Upper abdominal | Haematemesis | 0.00 (0.00, 1.98) |
| Upper abdominal | Jaundice | 0.00 (0.00, 1.98) |
| Upper abdominal | Loss of appetite | 6.84 (4.04, 11.4) |
| Upper abdominal | Nausea and/or vomiting | 4.21 (2.15, 8.09) |
| Upper abdominal | New onset diabetes | 1.05 (0.29, 3.76) |
| Upper abdominal | Upper abdominal pain | 5.79 (3.26, 10.1) |
| Lower abdominal | Abdominal pain (NOS) | 5.26 (2.88, 9.42) |
| Lower abdominal | Change in bowel habit | 2.11 (0.82, 5.29) |
| Lower abdominal | Constipation | 2.11 (0.82, 5.29) |
| Lower abdominal | Diarrhoea | 3.68 (1.80, 7.41) |
| Lower abdominal | Distension | 4.74 (2.51, 8.76) |
| Lower abdominal | Lower abdominal pain | 2.63 (1.13, 6.01) |
| Lower abdominal | Pelvic pain | 1.05 (0.29, 3.76) |
| Lower abdominal | Rectal bleeding | 2.11 (0.82, 5.29) |
| Respiratory | Chest infection | 4.74 (2.51, 8.76) |
| Respiratory | Chest pain | 5.26 (2.88, 9.42) |
| Respiratory | Clubbing | 0.00 (0.00, 1.98) |
| Respiratory | Cough | 4.21 (2.15, 8.09) |
| Respiratory | Dyspnoea | 8.42 (5.25, 13.2) |
| Respiratory | Haemoptysis | 0.53 (0.09, 2.92) |
| Respiratory | Hoarseness | 1.05 (0.29, 3.76) |
| Respiratory | Sore throat | 1.58 (0.54, 4.54) |
| Respiratory | Stridor | 0.00 (0.00, 1.98) |
| Urological | Dysuria | 1.05 (0.29, 3.76) |
| Urological | Haematuria | 1.05 (0.29, 3.76) |
| Urological | Loin pain | 0.53 (0.09, 2.92) |
| Urological | LUTS (nocturia, frequency, hesitancy, urgency, retention) | 2.11 (0.82, 5.29) |
| Urological | Renal colic | 0.00 (0.00, 1.98) |
| Urological | Urinary tract infection | 0.53 (0.09, 2.92) |
| Central nervous system | Fit/seizure | 0.53 (0.09, 2.92) |
| Central nervous system | Headache | 0.53 (0.09, 2.92) |
| Central nervous system | Prog/sub-acute loss of central neuro funct | 1.05 (0.29, 3.76) |
| Central nervous system | Visual disturbance or loss | 0.00 (0.00, 1.98) |
| Musculoskeletal | Back pain | 2.63 (1.13, 6.01) |
| Musculoskeletal | Bone pain | 2.63 (1.13, 6.01) |
| Musculoskeletal | Fracture | 0.00 (0.00, 1.98) |
| Skin Lesion | Abnormal mole | 2.11 (0.82, 5.29) |
| Skin Lesion | Lesions suspicious of BCC | 0.53 (0.09, 2.92) |
| Skin Lesion | Non-pigmented lesion | 0.00 (0.00, 1.98) |
| Breast Symptoms | Axillary lump/mass | 0.00 (0.00, 1.98) |
| Breast Symptoms | Breast lump/mass | 0.00 (0.00, 1.98) |
| Breast Symptoms | Breast pain | 0.53 (0.09, 2.92) |
| Breast Symptoms | Nipple changes | 0.00 (0.00, 1.98) |
| Breast Symptoms | Nipple discharge | 0.00 (0.00, 1.98) |
| Female specific | Other vaginal bleeding | 2.11 (0.82, 5.29) |
| Female specific | Post-menopausal bleeding | 1.58 (0.54, 4.54) |
| Female specific | Vaginal discharge | 0.00 (0.00, 1.98) |
| Female specific | Vaginal mass | 0.00 (0.00, 1.98) |
| Female specific | Vulval bleeding | 0.00 (0.00, 1.98) |
| Female specific | Vulval mass | 0.00 (0.00, 1.98) |
| Female specific | Vulval ulceration | 0.00 (0.00, 1.98) |
| Male specific | Erectile dysfunction | 0.00 (0.00, 1.98) |
| Male specific | Penile ulceration | 0.00 (0.00, 1.98) |
| Male specific | Testicular lump | 0.00 (0.00, 1.98) |
| Male specific | Testicular pain | 0.53 (0.09, 2.92) |
| None recorded | N/A | 22.1 (16.8, 28.5) |
| None recorded | N/K | 8.42 (5.25, 13.2) |
| *Unknown primary - Unknown primary (N = 1205)* | | |
| Non-specific | Bruising, bleeding or petechiae | 0.25 (0.08, 0.73) |
| Non-specific | Deep vein thrombosis | 0.50 (0.23, 1.08) |
| Non-specific | Epistaxis | 0.08 (0.01, 0.47) |
| Non-specific | Fatigue | 8.71 (7.25, 10.4) |
| Non-specific | Fever | 0.58 (0.28, 1.19) |
| Non-specific | Infection | 0.50 (0.23, 1.08) |
| Non-specific | Night sweats | 1.24 (0.76, 2.04) |
| Non-specific | Other symptom | 8.22 (6.79, 9.90) |
| Non-specific | Pallor | 0.50 (0.23, 1.08) |
| Non-specific | Pruritus | 0.66 (0.34, 1.30) |
| Non-specific | Weight loss | 13.4 (11.6, 15.5) |
| Lump/mass/lymph node | Anal mass | 0.17 (0.05, 0.60) |
| Lump/mass/lymph node | Lymph node pain with alcohol | 0.00 (0.00, 0.32) |
| Lump/mass/lymph node | Lymphadenopathy (generalised) | 0.00 (0.00, 0.32) |
| Lump/mass/lymph node | Lymphadenopathy (localised) | 1.83 (1.21, 2.75) |
| Lump/mass/lymph node | Neck lump/mass | 6.39 (5.14, 7.91) |
| Lump/mass/lymph node | Thyroid lump/mass | 0.08 (0.01, 0.47) |
| Lump/mass/lymph node | Unexplained lump suspicious of sarcoma | 0.50 (0.23, 1.08) |
| Ulceration | Leukoplakia | 0.00 (0.00, 0.32) |
| Ulceration | Lip/oral cavity/tongue lump/mass | 0.17 (0.05, 0.60) |
| Ulceration | Lip/oral cavity/tongue ulcer | 0.08 (0.01, 0.47) |
| Ulceration | Ulceration | 0.66 (0.34, 1.30) |
| Upper abdominal | Dyspepsia | 1.66 (1.08, 2.55) |
| Upper abdominal | Dysphagia | 1.33 (0.82, 2.15) |
| Upper abdominal | Early satiety | 0.33 (0.13, 0.85) |
| Upper abdominal | Gastroesophageal reflux | 1.00 (0.57, 1.73) |
| Upper abdominal | Haematemesis | 0.66 (0.34, 1.30) |
| Upper abdominal | Jaundice | 1.08 (0.63, 1.84) |
| Upper abdominal | Loss of appetite | 8.46 (7.02, 10.2) |
| Upper abdominal | Nausea and/or vomiting | 6.39 (5.14, 7.91) |
| Upper abdominal | New onset diabetes | 0.00 (0.00, 0.32) |
| Upper abdominal | Upper abdominal pain | 5.56 (4.40, 7.00) |
| Lower abdominal | Abdominal pain (NOS) | 10.8 (9.16, 12.7) |
| Lower abdominal | Change in bowel habit | 4.23 (3.23, 5.52) |
| Lower abdominal | Constipation | 4.32 (3.31, 5.62) |
| Lower abdominal | Diarrhoea | 3.90 (2.95, 5.15) |
| Lower abdominal | Distension | 6.14 (4.92, 7.64) |
| Lower abdominal | Lower abdominal pain | 3.15 (2.31, 4.30) |
| Lower abdominal | Pelvic pain | 2.07 (1.41, 3.04) |
| Lower abdominal | Rectal bleeding | 1.58 (1.01, 2.45) |
| Respiratory | Chest infection | 1.74 (1.14, 2.65) |
| Respiratory | Chest pain | 2.82 (2.03, 3.92) |
| Respiratory | Clubbing | 0.08 (0.01, 0.47) |
| Respiratory | Cough | 3.73 (2.80, 4.96) |
| Respiratory | Dyspnoea | 5.73 (4.55, 7.18) |
| Respiratory | Haemoptysis | 0.17 (0.05, 0.60) |
| Respiratory | Hoarseness | 0.66 (0.34, 1.30) |
| Respiratory | Sore throat | 0.33 (0.13, 0.85) |
| Respiratory | Stridor | 0.00 (0.00, 0.32) |
| Urological | Dysuria | 0.41 (0.18, 0.97) |
| Urological | Haematuria | 0.91 (0.51, 1.63) |
| Urological | Loin pain | 0.33 (0.13, 0.85) |
| Urological | LUTS (nocturia, frequency, hesitancy, urgency, retention) | 1.08 (0.63, 1.84) |
| Urological | Renal colic | 0.17 (0.05, 0.60) |
| Urological | Urinary tract infection | 0.58 (0.28, 1.19) |
| Central nervous system | Fit/seizure | 0.41 (0.18, 0.97) |
| Central nervous system | Headache | 1.00 (0.57, 1.73) |
| Central nervous system | Prog/sub-acute loss of central neuro funct | 2.16 (1.48, 3.14) |
| Central nervous system | Visual disturbance or loss | 0.17 (0.05, 0.60) |
| Musculoskeletal | Back pain | 7.30 (5.97, 8.91) |
| Musculoskeletal | Bone pain | 2.66 (1.89, 3.72) |
| Musculoskeletal | Fracture | 0.33 (0.13, 0.85) |
| Skin Lesion | Abnormal mole | 0.17 (0.05, 0.60) |
| Skin Lesion | Lesions suspicious of BCC | 0.00 (0.00, 0.32) |
| Skin Lesion | Non-pigmented lesion | 0.17 (0.05, 0.60) |
| Breast Symptoms | Axillary lump/mass | 0.91 (0.51, 1.63) |
| Breast Symptoms | Breast lump/mass | 0.17 (0.05, 0.60) |
| Breast Symptoms | Breast pain | 0.00 (0.00, 0.32) |
| Breast Symptoms | Nipple changes | 0.00 (0.00, 0.32) |
| Breast Symptoms | Nipple discharge | 0.00 (0.00, 0.32) |
| Female specific | Other vaginal bleeding | 0.25 (0.08, 0.73) |
| Female specific | Post-menopausal bleeding | 0.50 (0.23, 1.08) |
| Female specific | Vaginal discharge | 0.08 (0.01, 0.47) |
| Female specific | Vaginal mass | 0.00 (0.00, 0.32) |
| Female specific | Vulval bleeding | 0.00 (0.00, 0.32) |
| Female specific | Vulval mass | 0.17 (0.05, 0.60) |
| Female specific | Vulval ulceration | 0.00 (0.00, 0.32) |
| Male specific | Erectile dysfunction | 0.00 (0.00, 0.32) |
| Male specific | Penile ulceration | 0.00 (0.00, 0.32) |
| Male specific | Testicular lump | 0.00 (0.00, 0.32) |
| Male specific | Testicular pain | 0.00 (0.00, 0.32) |
| None recorded | N/A | 13.5 (11.7, 15.6) |
| None recorded | N/K | 7.30 (5.97, 8.91) |

## Supplementary table 5

Supplementary Table 5. Number of symptoms per cancer site that occurred in more than 1%, 5%, 10%, 20% and 50% of cases.

|  |  |  | Number of symptoms in | | | | |
| --- | --- | --- | --- | --- | --- | --- | --- |
| Cancer Site | N | % with at least one recorded symptom | >1% | >5% | >10% | >20% | >50% |
| Head and neck | | | | | | | |
| Larynx | 368 | 91.8 (88.6, 94.2) | 13 | 5 | 2 | 1 | 1 |
| Oral cavity | 585 | 70.6 (66.8, 74.1) | 12 | 5 | 2 | 2 | 0 |
| Oropharynx | 653 | 92.2 (89.9, 94.0) | 12 | 6 | 2 | 2 | 0 |
| Thyroid | 633 | 73.6 (70.0, 76.9) | 11 | 3 | 2 | 2 | 0 |
| Other head and neck | 481 | 88.6 (85.4, 91.1) | 15 | 7 | 3 | 1 | 0 |
| Upper gastrointestinal | | | | | | | |
| Oesophagus | 1391 | 89.8 (88.1, 91.3) | 21 | 7 | 4 | 2 | 0 |
| Stomach | 1029 | 84.6 (82.3, 86.7) | 23 | 10 | 5 | 1 | 0 |
| Lower gastrointestinal | | | | | | | |
| Anal | 265 | 91.3 (87.3, 94.1) | 16 | 7 | 3 | 2 | 1 |
| Colon | 4209 | 80.0 (78.7, 81.2) | 17 | 12 | 5 | 0 | 0 |
| Rectum | 1641 | 88.8 (87.2, 90.3) | 15 | 6 | 4 | 2 | 1 |
| Small intestine | 256 | 77.7 (72.2, 82.4) | 21 | 12 | 5 | 0 | 0 |
| Hepato-pancreato-biliary (HPB) | | | | | | | |
| Liver | 929 | 64.4 (61.2, 67.4) | 21 | 9 | 4 | 0 | 0 |
| Pancreas | 1725 | 84.8 (83.0, 86.4) | 23 | 13 | 6 | 1 | 0 |
| Other HPB | 425 | 81.9 (77.9, 85.3) | 20 | 11 | 5 | 1 | 0 |
| Respiratory | | | | | | | |
| Lung | 7608 | 76.4 (75.4, 77.3) | 17 | 8 | 4 | 1 | 0 |
| Mesothelioma | 464 | 87.5 (84.2, 90.2) | 15 | 6 | 4 | 3 | 0 |
| Urological | | | | | | | |
| Bladder | 1518 | 84.7 (82.8, 86.4) | 11 | 4 | 3 | 1 | 1 |
| Kidney | 1695 | 63.9 (61.6, 66.1) | 26 | 4 | 1 | 0 | 0 |
| Ureteric and other urinary | 275 | 80.0 (74.9, 84.3) | 19 | 5 | 1 | 1 | 0 |
| Haematological | | | | | | | |
| Acute leukaemia | 471 | 72.0 (67.8, 75.8) | 27 | 8 | 2 | 1 | 0 |
| Chronic lymphocytic leukaemia | 529 | 47.6 (43.4, 51.9) | 16 | 2 | 1 | 0 | 0 |
| Hodgkin lymphoma | 243 | 86.0 (81.1, 89.8) | 23 | 8 | 4 | 1 | 0 |
| Multiple myeloma | 904 | 66.6 (63.5, 69.6) | 19 | 6 | 1 | 1 | 0 |
| Non-Hodgkin lymphoma | 2055 | 78.1 (76.3, 79.8) | 30 | 6 | 1 | 0 | 0 |
| Other haematological | 588 | 61.2 (57.2, 65.1) | 26 | 4 | 2 | 0 | 0 |
| Central Nervous System (CNS) | | | | | | | |
| CNS | 777 | 78.6 (75.6, 81.4) | 9 | 5 | 4 | 1 | 0 |
| Sarcoma | | | | | | | |
| Bone sarcoma | 63 | 79.4 (67.8, 87.5) | 15 | 4 | 4 | 1 | 0 |
| Connective and soft tissue sarcoma | 372 | 86.0 (82.1, 89.2) | 26 | 5 | 2 | 1 | 0 |
| Skin | | | | | | | |
| Melanoma | 2790 | 91.8 (90.7, 92.8) | 6 | 3 | 1 | 1 | 1 |
| Ocular | | | | | | | |
| Ocular | 111 | 40.5 (31.9, 49.8) | 2 | 2 | 2 | 1 | 0 |
| Breast | | | | | | | |
| Breast | 6166 | 91.8 (91.1, 92.5) | 7 | 3 | 2 | 1 | 1 |
| Gynaecological | | | | | | | |
| Cervix | 252 | 86.9 (82.2, 90.5) | 20 | 5 | 2 | 2 | 0 |
| Ovary | 1174 | 86.7 (84.7, 88.5) | 27 | 11 | 3 | 1 | 0 |
| Uterus | 1572 | 93.2 (91.8, 94.3) | 17 | 2 | 2 | 1 | 1 |
| Vulva/Vagina | 241 | 84.6 (79.6, 88.7) | 14 | 4 | 2 | 2 | 0 |
| Prostate and other male organs | | | | | | | |
| Penile | 119 | 86.6 (79.3, 91.6) | 11 | 4 | 2 | 1 | 0 |
| Prostate | 8747 | 70.1 (69.2, 71.1) | 13 | 1 | 1 | 1 | 0 |
| Testicular | 403 | 90.8 (87.6, 93.3) | 6 | 2 | 2 | 2 | 1 |
| Other malignant neoplasms | | | | | | | |
| Other malignant neoplasms | 190 | 69.5 (62.6, 75.6) | 35 | 8 | 0 | 0 | 0 |
| Unknown primary | | | | | | | |
| Unknown primary | 1205 | 79.2 (76.8, 81.4) | 28 | 11 | 2 | 0 | 0 |

## Supplementary table 6

Supplementary Table 6. Proportion of cancer groups occurring in each symptom group.

|  | Proportion of Patients by Symptom Group % (95% CI) | |
| --- | --- | --- |
| Cancer Group | Men | Men |
| *Non-specific (Men=6086, Women=4739)* | | |
| Head and neck | 3.78 (3.33, 4.29) | 3.19 (2.72, 3.73) |
| Upper gastrointestinal | 8.89 (8.20, 9.63) | 5.34 (4.73, 6.02) |
| Lower gastrointestinal | 13.6 (12.8, 14.5) | 16.5 (15.5, 17.6) |
| Hepato-pancreato-biliary | 10.3 (9.53, 11.1) | 11.5 (10.7, 12.5) |
| Respiratory | 18.5 (17.5, 19.4) | 20.1 (19.0, 21.3) |
| Urological | 4.40 (3.92, 4.95) | 4.07 (3.55, 4.67) |
| Haematological | 17.0 (16.1, 17.9) | 16.1 (15.1, 17.2) |
| Central nervous system | 1.63 (1.34, 1.98) | 1.94 (1.59, 2.37) |
| Sarcoma | 0.72 (0.54, 0.97) | 0.89 (0.66, 1.20) |
| Skin | 1.36 (1.10, 1.69) | 1.79 (1.45, 2.21) |
| Ocular | 0.07 (0.03, 0.17) | 0.25 (0.14, 0.44) |
| Breast | 0.03 (0.01, 0.12) | 5.17 (4.57, 5.84) |
| Gynaecological | - | 8.12 (7.38, 8.94) |
| Prostate and other male organs | 15.9 (15.0, 16.8) | - |
| Other malignant neoplasms | 0.59 (0.43, 0.82) | 0.44 (0.29, 0.68) |
| Unknown primary | 3.37 (2.94, 3.85) | 4.49 (3.94, 5.12) |
| *Lump/mass/lymph node (Men=1180, Women=1005)* | | |
| Head and neck | 41.2 (38.4, 44.0) | 43.1 (40.1, 46.2) |
| Upper gastrointestinal | 1.27 (0.77, 2.09) | 0.10 (0.02, 0.56) |
| Lower gastrointestinal | 4.15 (3.16, 5.45) | 6.77 (5.37, 8.49) |
| Hepato-pancreato-biliary | 0.17 (0.05, 0.62) | 0.60 (0.27, 1.30) |
| Respiratory | 3.81 (2.86, 5.06) | 4.58 (3.45, 6.05) |
| Urological | 0.85 (0.46, 1.55) | 0.60 (0.27, 1.30) |
| Haematological | 32.9 (30.3, 35.6) | 29.9 (27.1, 32.8) |
| Central nervous system | 0.08 (0.01, 0.48) | 0.00 (0.00, 0.38) |
| Sarcoma | 6.10 (4.87, 7.62) | 5.27 (4.05, 6.83) |
| Skin | 1.19 (0.71, 1.98) | 1.19 (0.68, 2.08) |
| Ocular | 0.00 (0.00, 0.32) | 0.00 (0.00, 0.38) |
| Breast | 0.00 (0.00, 0.32) | 2.69 (1.85, 3.88) |
| Gynaecological | - | 1.49 (0.91, 2.45) |
| Prostate and other male organs | 1.61 (1.03, 2.50) | - |
| Other malignant neoplasms | 0.51 (0.23, 1.10) | 0.30 (0.10, 0.87) |
| Unknown primary | 6.19 (4.95, 7.71) | 3.48 (2.51, 4.80) |
| *Ulceration (Men=416, Women=266)* | | |
| Head and neck | 67.1 (62.4, 71.4) | 66.9 (61.1, 72.3) |
| Upper gastrointestinal | 0.72 (0.25, 2.10) | 0.38 (0.07, 2.10) |
| Lower gastrointestinal | 1.20 (0.51, 2.78) | 1.13 (0.38, 3.26) |
| Hepato-pancreato-biliary | 0.24 (0.04, 1.35) | 0.00 (0.00, 1.42) |
| Respiratory | 2.16 (1.14, 4.06) | 1.13 (0.38, 3.26) |
| Urological | 0.48 (0.13, 1.74) | 0.00 (0.00, 1.42) |
| Haematological | 7.45 (5.30, 10.4) | 6.02 (3.74, 9.55) |
| Central nervous system | 0.00 (0.00, 0.91) | 0.00 (0.00, 1.42) |
| Sarcoma | 3.12 (1.84, 5.27) | 1.13 (0.38, 3.26) |
| Skin | 13.2 (10.3, 16.8) | 13.5 (9.94, 18.2) |
| Ocular | 0.00 (0.00, 0.91) | 0.00 (0.00, 1.42) |
| Breast | 0.00 (0.00, 0.91) | 5.26 (3.16, 8.64) |
| Gynaecological | - | 2.26 (1.04, 4.83) |
| Prostate and other male organs | 2.64 (1.48, 4.67) | - |
| Other malignant neoplasms | 0.24 (0.04, 1.35) | 0.38 (0.07, 2.10) |
| Unknown primary | 1.44 (0.66, 3.11) | 1.88 (0.81, 4.32) |
| *Upper abdominal (Men=4177, Women=3664)* | | |
| Head and neck | 2.85 (2.39, 3.40) | 1.28 (0.97, 1.70) |
| Upper gastrointestinal | 36.0 (34.5, 37.4) | 20.3 (19.0, 21.6) |
| Lower gastrointestinal | 12.8 (11.9, 13.9) | 16.4 (15.2, 17.6) |
| Hepato-pancreato-biliary | 21.1 (19.9, 22.3) | 26.6 (25.2, 28.0) |
| Respiratory | 9.48 (8.63, 10.4) | 10.6 (9.61, 11.6) |
| Urological | 2.80 (2.34, 3.35) | 3.03 (2.52, 3.64) |
| Haematological | 6.56 (5.85, 7.35) | 6.11 (5.38, 6.94) |
| Central nervous system | 0.22 (0.11, 0.41) | 0.38 (0.23, 0.64) |
| Sarcoma | 0.24 (0.13, 0.44) | 0.96 (0.69, 1.33) |
| Skin | 0.26 (0.15, 0.47) | 0.11 (0.04, 0.28) |
| Ocular | 0.00 (0.00, 0.09) | 0.00 (0.00, 0.10) |
| Breast | 0.05 (0.01, 0.17) | 1.61 (1.25, 2.07) |
| Gynaecological | - | 7.21 (6.41, 8.09) |
| Prostate and other male organs | 3.93 (3.38, 4.56) | - |
| Other malignant neoplasms | 0.43 (0.27, 0.68) | 0.57 (0.38, 0.87) |
| Unknown primary | 3.33 (2.83, 3.92) | 4.91 (4.26, 5.66) |
| *Lower abdominal (Men=5844, Women=5927)* | | |
| Head and neck | 0.09 (0.04, 0.20) | 0.15 (0.08, 0.29) |
| Upper gastrointestinal | 4.95 (4.42, 5.53) | 2.65 (2.27, 3.09) |
| Lower gastrointestinal | 55.7 (54.4, 56.9) | 46.0 (44.7, 47.2) |
| Hepato-pancreato-biliary | 11.6 (10.8, 12.4) | 10.5 (9.72, 11.3) |
| Respiratory | 4.06 (3.58, 4.59) | 4.03 (3.56, 4.56) |
| Urological | 4.45 (3.95, 5.01) | 3.63 (3.18, 4.13) |
| Haematological | 5.56 (5.00, 6.18) | 3.91 (3.45, 4.44) |
| Central nervous system | 0.02 (0.00, 0.10) | 0.07 (0.03, 0.17) |
| Sarcoma | 0.48 (0.33, 0.69) | 1.35 (1.09, 1.68) |
| Skin | 0.03 (0.01, 0.12) | 0.05 (0.02, 0.15) |
| Ocular | 0.00 (0.00, 0.07) | 0.00 (0.00, 0.06) |
| Breast | 0.02 (0.00, 0.10) | 0.83 (0.63, 1.09) |
| Gynaecological | - | 22.0 (20.9, 23.0) |
| Prostate and other male organs | 9.87 (9.13, 10.7) | - |
| Other malignant neoplasms | 0.27 (0.17, 0.44) | 0.49 (0.34, 0.70) |
| Unknown primary | 2.98 (2.57, 3.45) | 4.42 (3.93, 4.97) |
| *Respiratory (Men=5476, Women=4122)* | | |
| Head and neck | 11.7 (10.9, 12.6) | 6.02 (5.33, 6.78) |
| Upper gastrointestinal | 3.09 (2.66, 3.58) | 1.87 (1.50, 2.33) |
| Lower gastrointestinal | 3.10 (2.68, 3.60) | 3.28 (2.77, 3.86) |
| Hepato-pancreato-biliary | 1.77 (1.45, 2.16) | 1.99 (1.61, 2.46) |
| Respiratory | 64.4 (63.1, 65.7) | 70.6 (69.2, 71.9) |
| Urological | 2.14 (1.79, 2.55) | 1.33 (1.03, 1.73) |
| Haematological | 8.60 (7.89, 9.37) | 7.04 (6.29, 7.86) |
| Central nervous system | 0.07 (0.03, 0.19) | 0.07 (0.02, 0.21) |
| Sarcoma | 0.16 (0.09, 0.31) | 0.32 (0.18, 0.54) |
| Skin | 0.27 (0.17, 0.45) | 0.12 (0.05, 0.28) |
| Ocular | 0.00 (0.00, 0.07) | 0.00 (0.00, 0.09) |
| Breast | 0.05 (0.02, 0.16) | 2.69 (2.24, 3.23) |
| Gynaecological | - | 2.21 (1.80, 2.70) |
| Prostate and other male organs | 2.25 (1.89, 2.67) | - |
| Other malignant neoplasms | 0.60 (0.43, 0.85) | 0.39 (0.24, 0.63) |
| Unknown primary | 1.77 (1.45, 2.16) | 2.11 (1.71, 2.60) |
| *Urological (Men=6900, Women=1104)* | | |
| Head and neck | 0.01 (0.00, 0.08) | 0.09 (0.02, 0.51) |
| Upper gastrointestinal | 0.14 (0.08, 0.27) | 0.63 (0.31, 1.30) |
| Lower gastrointestinal | 1.19 (0.96, 1.47) | 3.53 (2.59, 4.79) |
| Hepato-pancreato-biliary | 0.72 (0.55, 0.95) | 2.90 (2.06, 4.06) |
| Respiratory | 0.61 (0.45, 0.82) | 3.26 (2.36, 4.48) |
| Urological | 21.7 (20.7, 22.7) | 54.3 (51.3, 57.2) |
| Haematological | 1.33 (1.09, 1.63) | 3.62 (2.67, 4.90) |
| Central nervous system | 0.04 (0.01, 0.13) | 0.45 (0.19, 1.06) |
| Sarcoma | 0.06 (0.02, 0.15) | 0.82 (0.43, 1.54) |
| Skin | 0.01 (0.00, 0.08) | 0.09 (0.02, 0.51) |
| Ocular | 0.01 (0.00, 0.08) | 0.00 (0.00, 0.35) |
| Breast | 0.01 (0.00, 0.08) | 0.45 (0.19, 1.06) |
| Gynaecological | - | 27.7 (25.2, 30.4) |
| Prostate and other male organs | 73.7 (72.7, 74.8) | - |
| Other malignant neoplasms | 0.09 (0.04, 0.19) | 0.36 (0.14, 0.93) |
| Unknown primary | 0.32 (0.21, 0.48) | 1.81 (1.18, 2.78) |
| *Central nervous system (Men=633, Women=526)* | | |
| Head and neck | 3.63 (2.43, 5.39) | 2.85 (1.74, 4.65) |
| Upper gastrointestinal | 1.11 (0.54, 2.26) | 1.14 (0.52, 2.47) |
| Lower gastrointestinal | 2.53 (1.56, 4.07) | 2.28 (1.31, 3.94) |
| Hepato-pancreato-biliary | 2.21 (1.32, 3.68) | 1.71 (0.90, 3.22) |
| Respiratory | 18.0 (15.2, 21.2) | 23.4 (20.0, 27.2) |
| Urological | 1.58 (0.86, 2.88) | 1.71 (0.90, 3.22) |
| Haematological | 9.32 (7.29, 11.8) | 11.0 (8.63, 14.0) |
| Central nervous system | 51.0 (47.1, 54.9) | 43.9 (39.7, 48.2) |
| Sarcoma | 0.16 (0.03, 0.89) | 0.57 (0.19, 1.66) |
| Skin | 1.42 (0.75, 2.68) | 0.38 (0.10, 1.38) |
| Ocular | 1.58 (0.86, 2.88) | 3.23 (2.03, 5.11) |
| Breast | 0.00 (0.00, 0.60) | 2.47 (1.45, 4.18) |
| Gynaecological | - | 1.33 (0.65, 2.72) |
| Prostate and other male organs | 3.00 (1.93, 4.64) | - |
| Other malignant neoplasms | 0.63 (0.25, 1.61) | 0.00 (0.00, 0.73) |
| Unknown primary | 3.79 (2.56, 5.58) | 3.99 (2.63, 6.03) |
| *Musculoskeletal (Men=1390, Women=949)* | | |
| Head and neck | 0.29 (0.11, 0.74) | 1.05 (0.57, 1.93) |
| Upper gastrointestinal | 1.73 (1.16, 2.56) | 2.42 (1.62, 3.61) |
| Lower gastrointestinal | 3.24 (2.43, 4.30) | 4.64 (3.47, 6.17) |
| Hepato-pancreato-biliary | 3.74 (2.86, 4.87) | 6.64 (5.22, 8.40) |
| Respiratory | 19.4 (17.4, 21.5) | 29.2 (26.4, 32.2) |
| Urological | 5.97 (4.84, 7.34) | 6.32 (4.94, 8.05) |
| Haematological | 19.9 (17.9, 22.1) | 23.9 (21.3, 26.7) |
| Central nervous system | 0.50 (0.24, 1.04) | 1.05 (0.57, 1.93) |
| Sarcoma | 2.52 (1.82, 3.48) | 2.74 (1.88, 3.98) |
| Skin | 0.22 (0.07, 0.63) | 0.32 (0.11, 0.93) |
| Ocular | 0.00 (0.00, 0.28) | 0.00 (0.00, 0.40) |
| Breast | 0.07 (0.01, 0.41) | 8.54 (6.92, 10.5) |
| Gynaecological | - | 6.32 (4.94, 8.05) |
| Prostate and other male organs | 37.5 (35.0, 40.1) | - |
| Other malignant neoplasms | 0.43 (0.20, 0.94) | 0.42 (0.16, 1.08) |
| Unknown primary | 4.53 (3.56, 5.76) | 6.43 (5.04, 8.17) |
| *Skin Lesion (Men=1138, Women=1282)* | | |
| Head and neck | 0.26 (0.09, 0.77) | 0.08 (0.01, 0.44) |
| Upper gastrointestinal | 0.00 (0.00, 0.34) | 0.00 (0.00, 0.30) |
| Lower gastrointestinal | 0.00 (0.00, 0.34) | 0.00 (0.00, 0.30) |
| Hepato-pancreato-biliary | 0.00 (0.00, 0.34) | 0.00 (0.00, 0.30) |
| Respiratory | 0.53 (0.24, 1.15) | 0.08 (0.01, 0.44) |
| Urological | 0.09 (0.02, 0.50) | 0.00 (0.00, 0.30) |
| Haematological | 1.41 (0.87, 2.27) | 0.86 (0.48, 1.53) |
| Central nervous system | 0.00 (0.00, 0.34) | 0.00 (0.00, 0.30) |
| Sarcoma | 0.88 (0.48, 1.61) | 0.31 (0.12, 0.80) |
| Skin | 96.0 (94.7, 97.0) | 97.7 (96.8, 98.4) |
| Ocular | 0.00 (0.00, 0.34) | 0.08 (0.01, 0.44) |
| Breast | 0.09 (0.02, 0.50) | 0.39 (0.17, 0.91) |
| Gynaecological | - | 0.23 (0.08, 0.69) |
| Prostate and other male organs | 0.18 (0.05, 0.64) | - |
| Other malignant neoplasms | 0.35 (0.14, 0.90) | 0.08 (0.01, 0.44) |
| Unknown primary | 0.18 (0.05, 0.64) | 0.16 (0.04, 0.57) |
| *Breast Symptoms (Men=156, Women=6258)* | | |
| Head and neck | 0.64 (0.11, 3.54) | 0.10 (0.04, 0.21) |
| Upper gastrointestinal | 1.92 (0.66, 5.50) | 0.03 (0.01, 0.12) |
| Lower gastrointestinal | 2.56 (1.00, 6.41) | 0.10 (0.04, 0.21) |
| Hepato-pancreato-biliary | 0.64 (0.11, 3.54) | 0.03 (0.01, 0.12) |
| Respiratory | 5.13 (2.62, 9.79) | 0.56 (0.40, 0.78) |
| Urological | 0.64 (0.11, 3.54) | 0.05 (0.02, 0.14) |
| Haematological | 25.6 (19.4, 33.0) | 1.36 (1.10, 1.68) |
| Central nervous system | 0.00 (0.00, 2.40) | 0.03 (0.01, 0.12) |
| Sarcoma | 0.00 (0.00, 2.40) | 0.10 (0.04, 0.21) |
| Skin | 5.77 (3.06, 10.6) | 0.05 (0.02, 0.14) |
| Ocular | 0.00 (0.00, 2.40) | 0.00 (0.00, 0.06) |
| Breast | 45.5 (37.9, 53.3) | 97.3 (96.9, 97.7) |
| Gynaecological | - | 0.19 (0.11, 0.33) |
| Prostate and other male organs | 5.13 (2.62, 9.79) | - |
| Other malignant neoplasms | 0.64 (0.11, 3.54) | 0.00 (0.00, 0.06) |
| Unknown primary | 5.77 (3.06, 10.6) | 0.06 (0.02, 0.16) |
| *Female specific (Men=-, Women=1918)* | | |
| Head and neck | - | 0.10 (0.03, 0.38) |
| Upper gastrointestinal | - | 0.16 (0.05, 0.46) |
| Lower gastrointestinal | - | 0.78 (0.47, 1.29) |
| Hepato-pancreato-biliary | - | 0.31 (0.14, 0.68) |
| Respiratory | - | 0.21 (0.08, 0.54) |
| Urological | - | 1.41 (0.97, 2.04) |
| Haematological | - | 0.52 (0.28, 0.96) |
| Central nervous system | - | 0.00 (0.00, 0.20) |
| Sarcoma | - | 0.05 (0.01, 0.29) |
| Skin | - | 0.00 (0.00, 0.20) |
| Ocular | - | 0.00 (0.00, 0.20) |
| Breast | - | 0.47 (0.25, 0.89) |
| Gynaecological | - | 95.0 (93.9, 95.9) |
| Other malignant neoplasms | - | 0.36 (0.18, 0.75) |
| Unknown primary | - | 0.63 (0.36, 1.09) |
| *Male specific (Men=867, Women=-)* | | |
| Head and neck | 0.00 (0.00, 0.44) | - |
| Upper gastrointestinal | 0.00 (0.00, 0.44) | - |
| Lower gastrointestinal | 1.04 (0.55, 1.96) | - |
| Hepato-pancreato-biliary | 0.23 (0.06, 0.84) | - |
| Respiratory | 0.12 (0.02, 0.65) | - |
| Urological | 2.54 (1.68, 3.81) | - |
| Haematological | 4.61 (3.41, 6.22) | - |
| Central nervous system | 0.00 (0.00, 0.44) | - |
| Sarcoma | 0.12 (0.02, 0.65) | - |
| Skin | 0.00 (0.00, 0.44) | - |
| Ocular | 0.00 (0.00, 0.44) | - |
| Breast | 0.00 (0.00, 0.44) | - |
| Prostate and other male organs | 91.2 (89.2, 92.9) | - |
| Other malignant neoplasms | 0.12 (0.02, 0.65) | - |
| Unknown primary | 0.00 (0.00, 0.44) | - |
| *None recorded (Men=7016, Women=4050)* | | |
| Head and neck | 3.58 (3.17, 4.04) | 5.53 (4.87, 6.28) |
| Upper gastrointestinal | 2.85 (2.49, 3.27) | 2.47 (2.03, 2.99) |
| Lower gastrointestinal | 8.84 (8.20, 9.52) | 12.0 (11.0, 13.0) |
| Hepato-pancreato-biliary | 6.01 (5.48, 6.60) | 6.12 (5.43, 6.90) |
| Respiratory | 13.8 (13.0, 14.6) | 21.9 (20.6, 23.2) |
| Urological | 8.25 (7.63, 8.92) | 7.93 (7.13, 8.80) |
| Haematological | 11.9 (11.2, 12.7) | 14.5 (13.4, 15.6) |
| Central nervous system | 1.47 (1.21, 1.78) | 1.56 (1.22, 1.99) |
| Sarcoma | 0.50 (0.36, 0.69) | 0.74 (0.52, 1.06) |
| Skin | 2.08 (1.77, 2.44) | 2.05 (1.66, 2.53) |
| Ocular | 0.60 (0.44, 0.81) | 0.59 (0.40, 0.88) |
| Breast | 0.06 (0.02, 0.15) | 12.4 (11.4, 13.4) |
| Gynaecological | - | 8.22 (7.42, 9.11) |
| Prostate and other male organs | 38.0 (36.9, 39.1) | - |
| Other malignant neoplasms | 0.40 (0.28, 0.58) | 0.74 (0.52, 1.06) |
| Unknown primary | 1.65 (1.38, 1.98) | 3.33 (2.82, 3.93) |

## Supplementary table 7

Supplementary Table 7. Proportion of cancer sites occurring in each symptom. Proportions were suppressed for symptoms with N<20.

|  |  | Proportion of Patients by Symptom % (95% CI) | |
| --- | --- | --- | --- |
| Cancer Group | Cancer Site | Men | Women |
| *Non-specific - Bruising, bleeding or petechiae (Men=76, Women=82)* | | | |
| Head and neck | Larynx | 0.00 (0.00, 4.81) | 0.00 (0.00, 4.48) |
| Head and neck | Oral cavity | 0.00 (0.00, 4.81) | 0.00 (0.00, 4.48) |
| Head and neck | Oropharynx | 1.32 (0.23, 7.08) | 0.00 (0.00, 4.48) |
| Head and neck | Thyroid | 0.00 (0.00, 4.81) | 0.00 (0.00, 4.48) |
| Head and neck | Other head and neck | 0.00 (0.00, 4.81) | 0.00 (0.00, 4.48) |
| Upper gastrointestinal | Oesophagus | 1.32 (0.23, 7.08) | 1.22 (0.22, 6.59) |
| Upper gastrointestinal | Stomach | 1.32 (0.23, 7.08) | 0.00 (0.00, 4.48) |
| Lower gastrointestinal | Anal | 0.00 (0.00, 4.81) | 0.00 (0.00, 4.48) |
| Lower gastrointestinal | Colon | 3.95 (1.35, 11.0) | 2.44 (0.67, 8.46) |
| Lower gastrointestinal | Rectum | 0.00 (0.00, 4.81) | 0.00 (0.00, 4.48) |
| Lower gastrointestinal | Small intestine | 0.00 (0.00, 4.81) | 0.00 (0.00, 4.48) |
| Hepato-pancreato-biliary | Liver | 1.32 (0.23, 7.08) | 2.44 (0.67, 8.46) |
| Hepato-pancreato-biliary | Pancreas | 0.00 (0.00, 4.81) | 1.22 (0.22, 6.59) |
| Hepato-pancreato-biliary | Other Hepato-pancreato-biliary | 0.00 (0.00, 4.81) | 1.22 (0.22, 6.59) |
| Respiratory | Lung | 5.26 (2.07, 12.8) | 4.88 (1.91, 11.9) |
| Respiratory | Mesothelioma | 0.00 (0.00, 4.81) | 0.00 (0.00, 4.48) |
| Urological | Bladder | 0.00 (0.00, 4.81) | 0.00 (0.00, 4.48) |
| Urological | Kidney | 2.63 (0.72, 9.10) | 1.22 (0.22, 6.59) |
| Urological | Ureteric and other urinary | 1.32 (0.23, 7.08) | 0.00 (0.00, 4.48) |
| Haematological | Acute leukaemia | 13.2 (7.31, 22.6) | 28.0 (19.5, 38.6) |
| Haematological | Chronic lymphocytic leukaemia | 2.63 (0.72, 9.10) | 2.44 (0.67, 8.46) |
| Haematological | Hodgkin lymphoma | 0.00 (0.00, 4.81) | 0.00 (0.00, 4.48) |
| Haematological | Multiple myeloma | 3.95 (1.35, 11.0) | 8.54 (4.20, 16.6) |
| Haematological | Non-Hodgkin lymphoma | 7.89 (3.67, 16.2) | 4.88 (1.91, 11.9) |
| Haematological | Other haematological | 13.2 (7.31, 22.6) | 6.10 (2.63, 13.5) |
| Central nervous system | Central nervous system | 1.32 (0.23, 7.08) | 0.00 (0.00, 4.48) |
| Sarcoma | Bone sarcoma | 0.00 (0.00, 4.81) | 0.00 (0.00, 4.48) |
| Sarcoma | Connective and soft tissue sarcoma | 6.58 (2.84, 14.5) | 1.22 (0.22, 6.59) |
| Skin | Melanoma | 9.21 (4.53, 17.8) | 12.2 (6.76, 21.0) |
| Ocular | Ocular | 0.00 (0.00, 4.81) | 0.00 (0.00, 4.48) |
| Breast | Breast | 0.00 (0.00, 4.81) | 14.6 (8.57, 23.9) |
| Gynaecological | Cervix | - | 0.00 (0.00, 4.48) |
| Gynaecological | Ovary | - | 2.44 (0.67, 8.46) |
| Gynaecological | Uterus | - | 3.66 (1.25, 10.2) |
| Gynaecological | Vulva/Vagina | - | 0.00 (0.00, 4.48) |
| Prostate and other male organs | Penile | 5.26 (2.07, 12.8) | - |
| Prostate and other male organs | Prostate | 15.8 (9.27, 25.6) | - |
| Prostate and other male organs | Testicular | 0.00 (0.00, 4.81) | - |
| Other malignant neoplasms | Other malignant neoplasms | 0.00 (0.00, 4.81) | 0.00 (0.00, 4.48) |
| Unknown primary | Unknown primary | 2.63 (0.72, 9.10) | 1.22 (0.22, 6.59) |
| *Non-specific - Deep vein thrombosis (Men=55, Women=64)* | | | |
| Head and neck | Larynx | 0.00 (0.00, 6.53) | 0.00 (0.00, 5.66) |
| Head and neck | Oral cavity | 0.00 (0.00, 6.53) | 0.00 (0.00, 5.66) |
| Head and neck | Oropharynx | 1.82 (0.32, 9.61) | 0.00 (0.00, 5.66) |
| Head and neck | Thyroid | 0.00 (0.00, 6.53) | 0.00 (0.00, 5.66) |
| Head and neck | Other head and neck | 0.00 (0.00, 6.53) | 0.00 (0.00, 5.66) |
| Upper gastrointestinal | Oesophagus | 1.82 (0.32, 9.61) | 3.12 (0.86, 10.7) |
| Upper gastrointestinal | Stomach | 1.82 (0.32, 9.61) | 0.00 (0.00, 5.66) |
| Lower gastrointestinal | Anal | 0.00 (0.00, 6.53) | 0.00 (0.00, 5.66) |
| Lower gastrointestinal | Colon | 9.09 (3.95, 19.6) | 7.81 (3.38, 17.0) |
| Lower gastrointestinal | Rectum | 1.82 (0.32, 9.61) | 1.56 (0.28, 8.33) |
| Lower gastrointestinal | Small intestine | 0.00 (0.00, 6.53) | 0.00 (0.00, 5.66) |
| Hepato-pancreato-biliary | Liver | 5.45 (1.87, 14.9) | 0.00 (0.00, 5.66) |
| Hepato-pancreato-biliary | Pancreas | 7.27 (2.86, 17.3) | 12.5 (6.47, 22.8) |
| Hepato-pancreato-biliary | Other Hepato-pancreato-biliary | 3.64 (1.00, 12.3) | 1.56 (0.28, 8.33) |
| Respiratory | Lung | 3.64 (1.00, 12.3) | 20.3 (12.3, 31.7) |
| Respiratory | Mesothelioma | 0.00 (0.00, 6.53) | 0.00 (0.00, 5.66) |
| Urological | Bladder | 1.82 (0.32, 9.61) | 1.56 (0.28, 8.33) |
| Urological | Kidney | 3.64 (1.00, 12.3) | 1.56 (0.28, 8.33) |
| Urological | Ureteric and other urinary | 1.82 (0.32, 9.61) | 0.00 (0.00, 5.66) |
| Haematological | Acute leukaemia | 1.82 (0.32, 9.61) | 1.56 (0.28, 8.33) |
| Haematological | Chronic lymphocytic leukaemia | 0.00 (0.00, 6.53) | 0.00 (0.00, 5.66) |
| Haematological | Hodgkin lymphoma | 0.00 (0.00, 6.53) | 0.00 (0.00, 5.66) |
| Haematological | Multiple myeloma | 3.64 (1.00, 12.3) | 0.00 (0.00, 5.66) |
| Haematological | Non-Hodgkin lymphoma | 10.9 (5.10, 21.8) | 9.38 (4.37, 19.0) |
| Haematological | Other haematological | 0.00 (0.00, 6.53) | 0.00 (0.00, 5.66) |
| Central nervous system | Central nervous system | 0.00 (0.00, 6.53) | 0.00 (0.00, 5.66) |
| Sarcoma | Bone sarcoma | 0.00 (0.00, 6.53) | 0.00 (0.00, 5.66) |
| Sarcoma | Connective and soft tissue sarcoma | 1.82 (0.32, 9.61) | 1.56 (0.28, 8.33) |
| Skin | Melanoma | 0.00 (0.00, 6.53) | 0.00 (0.00, 5.66) |
| Ocular | Ocular | 0.00 (0.00, 6.53) | 0.00 (0.00, 5.66) |
| Breast | Breast | 0.00 (0.00, 6.53) | 3.12 (0.86, 10.7) |
| Gynaecological | Cervix | - | 3.12 (0.86, 10.7) |
| Gynaecological | Ovary | - | 25.0 (16.0, 36.8) |
| Gynaecological | Uterus | - | 1.56 (0.28, 8.33) |
| Gynaecological | Vulva/Vagina | - | 0.00 (0.00, 5.66) |
| Prostate and other male organs | Penile | 0.00 (0.00, 6.53) | - |
| Prostate and other male organs | Prostate | 29.1 (18.8, 42.1) | - |
| Prostate and other male organs | Testicular | 3.64 (1.00, 12.3) | - |
| Other malignant neoplasms | Other malignant neoplasms | 0.00 (0.00, 6.53) | 0.00 (0.00, 5.66) |
| Unknown primary | Unknown primary | 5.45 (1.87, 14.9) | 4.69 (1.61, 12.9) |
| *Non-specific - Epistaxis (Men=49, Women=32)* | | | |
| Head and neck | Larynx | 0.00 (0.00, 7.27) | 0.00 (0.00, 10.7) |
| Head and neck | Oral cavity | 4.08 (1.13, 13.7) | 0.00 (0.00, 10.7) |
| Head and neck | Oropharynx | 4.08 (1.13, 13.7) | 3.12 (0.55, 15.7) |
| Head and neck | Thyroid | 0.00 (0.00, 7.27) | 0.00 (0.00, 10.7) |
| Head and neck | Other head and neck | 34.7 (22.9, 48.7) | 31.2 (18.0, 48.6) |
| Upper gastrointestinal | Oesophagus | 2.04 (0.36, 10.7) | 0.00 (0.00, 10.7) |
| Upper gastrointestinal | Stomach | 0.00 (0.00, 7.27) | 0.00 (0.00, 10.7) |
| Lower gastrointestinal | Anal | 0.00 (0.00, 7.27) | 0.00 (0.00, 10.7) |
| Lower gastrointestinal | Colon | 0.00 (0.00, 7.27) | 3.12 (0.55, 15.7) |
| Lower gastrointestinal | Rectum | 0.00 (0.00, 7.27) | 0.00 (0.00, 10.7) |
| Lower gastrointestinal | Small intestine | 0.00 (0.00, 7.27) | 0.00 (0.00, 10.7) |
| Hepato-pancreato-biliary | Liver | 6.12 (2.10, 16.5) | 0.00 (0.00, 10.7) |
| Hepato-pancreato-biliary | Pancreas | 6.12 (2.10, 16.5) | 0.00 (0.00, 10.7) |
| Hepato-pancreato-biliary | Other Hepato-pancreato-biliary | 0.00 (0.00, 7.27) | 0.00 (0.00, 10.7) |
| Respiratory | Lung | 8.16 (3.22, 19.2) | 6.25 (1.73, 20.1) |
| Respiratory | Mesothelioma | 0.00 (0.00, 7.27) | 0.00 (0.00, 10.7) |
| Urological | Bladder | 0.00 (0.00, 7.27) | 0.00 (0.00, 10.7) |
| Urological | Kidney | 2.04 (0.36, 10.7) | 0.00 (0.00, 10.7) |
| Urological | Ureteric and other urinary | 0.00 (0.00, 7.27) | 0.00 (0.00, 10.7) |
| Haematological | Acute leukaemia | 2.04 (0.36, 10.7) | 6.25 (1.73, 20.1) |
| Haematological | Chronic lymphocytic leukaemia | 2.04 (0.36, 10.7) | 0.00 (0.00, 10.7) |
| Haematological | Hodgkin lymphoma | 0.00 (0.00, 7.27) | 0.00 (0.00, 10.7) |
| Haematological | Multiple myeloma | 4.08 (1.13, 13.7) | 18.8 (8.89, 35.3) |
| Haematological | Non-Hodgkin lymphoma | 10.2 (4.44, 21.8) | 6.25 (1.73, 20.1) |
| Haematological | Other haematological | 10.2 (4.44, 21.8) | 0.00 (0.00, 10.7) |
| Central nervous system | Central nervous system | 0.00 (0.00, 7.27) | 0.00 (0.00, 10.7) |
| Sarcoma | Bone sarcoma | 0.00 (0.00, 7.27) | 0.00 (0.00, 10.7) |
| Sarcoma | Connective and soft tissue sarcoma | 0.00 (0.00, 7.27) | 3.12 (0.55, 15.7) |
| Skin | Melanoma | 0.00 (0.00, 7.27) | 0.00 (0.00, 10.7) |
| Ocular | Ocular | 0.00 (0.00, 7.27) | 0.00 (0.00, 10.7) |
| Breast | Breast | 0.00 (0.00, 7.27) | 15.6 (6.86, 31.8) |
| Gynaecological | Cervix | - | 3.12 (0.55, 15.7) |
| Gynaecological | Ovary | - | 0.00 (0.00, 10.7) |
| Gynaecological | Uterus | - | 0.00 (0.00, 10.7) |
| Gynaecological | Vulva/Vagina | - | 0.00 (0.00, 10.7) |
| Prostate and other male organs | Penile | 0.00 (0.00, 7.27) | - |
| Prostate and other male organs | Prostate | 4.08 (1.13, 13.7) | - |
| Prostate and other male organs | Testicular | 0.00 (0.00, 7.27) | - |
| Other malignant neoplasms | Other malignant neoplasms | 0.00 (0.00, 7.27) | 0.00 (0.00, 10.7) |
| Unknown primary | Unknown primary | 0.00 (0.00, 7.27) | 3.12 (0.55, 15.7) |
| Non-specific - Fatigue (Men=1171, Women=1068) | | | |
| Head and neck | Larynx | 0.00 (0.00, 0.33) | 0.09 (0.02, 0.53) |
| Head and neck | Oral cavity | 0.09 (0.02, 0.48) | 0.09 (0.02, 0.53) |
| Head and neck | Oropharynx | 0.34 (0.13, 0.88) | 0.19 (0.05, 0.68) |
| Head and neck | Thyroid | 0.34 (0.13, 0.88) | 1.40 (0.85, 2.30) |
| Head and neck | Other head and neck | 0.17 (0.05, 0.62) | 0.09 (0.02, 0.53) |
| Upper gastrointestinal | Oesophagus | 2.73 (1.94, 3.83) | 1.22 (0.71, 2.07) |
| Upper gastrointestinal | Stomach | 3.67 (2.74, 4.91) | 2.53 (1.74, 3.65) |
| Lower gastrointestinal | Anal | 0.17 (0.05, 0.62) | 0.47 (0.20, 1.09) |
| Lower gastrointestinal | Colon | 11.4 (9.67, 13.3) | 16.8 (14.6, 19.1) |
| Lower gastrointestinal | Rectum | 2.22 (1.52, 3.23) | 2.15 (1.44, 3.21) |
| Lower gastrointestinal | Small intestine | 0.77 (0.40, 1.45) | 0.66 (0.32, 1.35) |
| Hepato-pancreato-biliary | Liver | 4.27 (3.25, 5.59) | 2.25 (1.51, 3.32) |
| Hepato-pancreato-biliary | Pancreas | 6.23 (4.99, 7.77) | 6.18 (4.89, 7.79) |
| Hepato-pancreato-biliary | Other Hepato-pancreato-biliary | 0.68 (0.35, 1.34) | 1.40 (0.85, 2.30) |
| Respiratory | Lung | 18.5 (16.4, 20.9) | 19.6 (17.3, 22.1) |
| Respiratory | Mesothelioma | 2.48 (1.73, 3.53) | 0.47 (0.20, 1.09) |
| Urological | Bladder | 0.94 (0.53, 1.67) | 1.12 (0.64, 1.95) |
| Urological | Kidney | 2.65 (1.87, 3.73) | 2.90 (2.05, 4.09) |
| Urological | Ureteric and other urinary | 0.34 (0.13, 0.88) | 0.37 (0.15, 0.96) |
| Haematological | Acute leukaemia | 5.29 (4.15, 6.73) | 5.62 (4.39, 7.16) |
| Haematological | Chronic lymphocytic leukaemia | 1.96 (1.31, 2.93) | 1.69 (1.07, 2.65) |
| Haematological | Hodgkin lymphoma | 1.11 (0.65, 1.89) | 1.40 (0.85, 2.30) |
| Haematological | Multiple myeloma | 3.59 (2.66, 4.81) | 4.21 (3.16, 5.59) |
| Haematological | Non-Hodgkin lymphoma | 6.66 (5.37, 8.24) | 5.71 (4.47, 7.27) |
| Haematological | Other haematological | 3.42 (2.52, 4.62) | 3.37 (2.44, 4.63) |
| Central nervous system | Central nervous system | 1.45 (0.91, 2.31) | 1.50 (0.92, 2.42) |
| Sarcoma | Bone sarcoma | 0.00 (0.00, 0.33) | 0.00 (0.00, 0.36) |
| Sarcoma | Connective and soft tissue sarcoma | 0.34 (0.13, 0.88) | 0.28 (0.10, 0.82) |
| Skin | Melanoma | 0.34 (0.13, 0.88) | 0.09 (0.02, 0.53) |
| Ocular | Ocular | 0.00 (0.00, 0.33) | 0.00 (0.00, 0.36) |
| Breast | Breast | 0.00 (0.00, 0.33) | 2.90 (2.05, 4.09) |
| Gynaecological | Cervix | - | 0.66 (0.32, 1.35) |
| Gynaecological | Ovary | - | 4.03 (3.00, 5.38) |
| Gynaecological | Uterus | - | 2.15 (1.44, 3.21) |
| Gynaecological | Vulva/Vagina | - | 0.09 (0.02, 0.53) |
| Prostate and other male organs | Penile | 0.00 (0.00, 0.33) | - |
| Prostate and other male organs | Prostate | 13.7 (11.8, 15.7) | - |
| Prostate and other male organs | Testicular | 0.00 (0.00, 0.33) | - |
| Other malignant neoplasms | Other malignant neoplasms | 0.43 (0.18, 1.00) | 0.66 (0.32, 1.35) |
| Unknown primary | Unknown primary | 3.76 (2.81, 5.01) | 5.71 (4.47, 7.27) |
| *Non-specific - Fever (Men=164, Women=109)* | | | |
| Head and neck | Larynx | 0.00 (0.00, 2.29) | 0.92 (0.16, 5.01) |
| Head and neck | Oral cavity | 0.00 (0.00, 2.29) | 0.00 (0.00, 3.40) |
| Head and neck | Oropharynx | 0.00 (0.00, 2.29) | 0.00 (0.00, 3.40) |
| Head and neck | Thyroid | 0.00 (0.00, 2.29) | 0.92 (0.16, 5.01) |
| Head and neck | Other head and neck | 0.61 (0.11, 3.37) | 0.92 (0.16, 5.01) |
| Upper gastrointestinal | Oesophagus | 0.61 (0.11, 3.37) | 0.00 (0.00, 3.40) |
| Upper gastrointestinal | Stomach | 3.05 (1.31, 6.94) | 0.00 (0.00, 3.40) |
| Lower gastrointestinal | Anal | 0.00 (0.00, 2.29) | 0.00 (0.00, 3.40) |
| Lower gastrointestinal | Colon | 10.4 (6.57, 16.0) | 11.0 (6.41, 18.3) |
| Lower gastrointestinal | Rectum | 0.00 (0.00, 2.29) | 0.92 (0.16, 5.01) |
| Lower gastrointestinal | Small intestine | 2.44 (0.95, 6.10) | 2.75 (0.94, 7.78) |
| Hepato-pancreato-biliary | Liver | 1.83 (0.62, 5.24) | 2.75 (0.94, 7.78) |
| Hepato-pancreato-biliary | Pancreas | 6.10 (3.35, 10.9) | 5.50 (2.55, 11.5) |
| Hepato-pancreato-biliary | Other Hepato-pancreato-biliary | 0.00 (0.00, 2.29) | 3.67 (1.44, 9.06) |
| Respiratory | Lung | 22.0 (16.3, 28.9) | 18.3 (12.2, 26.6) |
| Respiratory | Mesothelioma | 1.83 (0.62, 5.24) | 0.92 (0.16, 5.01) |
| Urological | Bladder | 1.83 (0.62, 5.24) | 1.83 (0.50, 6.44) |
| Urological | Kidney | 3.05 (1.31, 6.94) | 3.67 (1.44, 9.06) |
| Urological | Ureteric and other urinary | 0.61 (0.11, 3.37) | 1.83 (0.50, 6.44) |
| Haematological | Acute leukaemia | 9.15 (5.62, 14.5) | 9.17 (5.06, 16.1) |
| Haematological | Chronic lymphocytic leukaemia | 2.44 (0.95, 6.10) | 0.92 (0.16, 5.01) |
| Haematological | Hodgkin lymphoma | 2.44 (0.95, 6.10) | 3.67 (1.44, 9.06) |
| Haematological | Multiple myeloma | 1.22 (0.34, 4.34) | 4.59 (1.98, 10.3) |
| Haematological | Non-Hodgkin lymphoma | 7.93 (4.69, 13.1) | 4.59 (1.98, 10.3) |
| Haematological | Other haematological | 5.49 (2.91, 10.1) | 0.92 (0.16, 5.01) |
| Central nervous system | Central nervous system | 0.61 (0.11, 3.37) | 1.83 (0.50, 6.44) |
| Sarcoma | Bone sarcoma | 0.00 (0.00, 2.29) | 0.00 (0.00, 3.40) |
| Sarcoma | Connective and soft tissue sarcoma | 0.00 (0.00, 2.29) | 2.75 (0.94, 7.78) |
| Skin | Melanoma | 0.61 (0.11, 3.37) | 0.00 (0.00, 3.40) |
| Ocular | Ocular | 0.00 (0.00, 2.29) | 0.00 (0.00, 3.40) |
| Breast | Breast | 0.00 (0.00, 2.29) | 2.75 (0.94, 7.78) |
| Gynaecological | Cervix | - | 0.92 (0.16, 5.01) |
| Gynaecological | Ovary | - | 6.42 (3.15, 12.7) |
| Gynaecological | Uterus | - | 2.75 (0.94, 7.78) |
| Gynaecological | Vulva/Vagina | - | 0.92 (0.16, 5.01) |
| Prostate and other male organs | Penile | 0.00 (0.00, 2.29) | - |
| Prostate and other male organs | Prostate | 11.6 (7.54, 17.4) | - |
| Prostate and other male organs | Testicular | 0.00 (0.00, 2.29) | - |
| Other malignant neoplasms | Other malignant neoplasms | 1.22 (0.34, 4.34) | 0.00 (0.00, 3.40) |
| Unknown primary | Unknown primary | 3.05 (1.31, 6.94) | 1.83 (0.50, 6.44) |
| *Non-specific - Infection (Men=97, Women=72)* | | | |
| Head and neck | Larynx | 0.00 (0.00, 3.81) | 0.00 (0.00, 5.07) |
| Head and neck | Oral cavity | 1.03 (0.18, 5.61) | 1.39 (0.25, 7.46) |
| Head and neck | Oropharynx | 0.00 (0.00, 3.81) | 0.00 (0.00, 5.07) |
| Head and neck | Thyroid | 0.00 (0.00, 3.81) | 0.00 (0.00, 5.07) |
| Head and neck | Other head and neck | 5.15 (2.22, 11.5) | 0.00 (0.00, 5.07) |
| Upper gastrointestinal | Oesophagus | 3.09 (1.06, 8.70) | 0.00 (0.00, 5.07) |
| Upper gastrointestinal | Stomach | 2.06 (0.57, 7.21) | 1.39 (0.25, 7.46) |
| Lower gastrointestinal | Anal | 1.03 (0.18, 5.61) | 2.78 (0.77, 9.57) |
| Lower gastrointestinal | Colon | 2.06 (0.57, 7.21) | 5.56 (2.18, 13.4) |
| Lower gastrointestinal | Rectum | 1.03 (0.18, 5.61) | 0.00 (0.00, 5.07) |
| Lower gastrointestinal | Small intestine | 0.00 (0.00, 3.81) | 0.00 (0.00, 5.07) |
| Hepato-pancreato-biliary | Liver | 2.06 (0.57, 7.21) | 2.78 (0.77, 9.57) |
| Hepato-pancreato-biliary | Pancreas | 4.12 (1.62, 10.1) | 2.78 (0.77, 9.57) |
| Hepato-pancreato-biliary | Other Hepato-pancreato-biliary | 0.00 (0.00, 3.81) | 0.00 (0.00, 5.07) |
| Respiratory | Lung | 23.7 (16.4, 33.1) | 26.4 (17.6, 37.6) |
| Respiratory | Mesothelioma | 1.03 (0.18, 5.61) | 0.00 (0.00, 5.07) |
| Urological | Bladder | 2.06 (0.57, 7.21) | 2.78 (0.77, 9.57) |
| Urological | Kidney | 1.03 (0.18, 5.61) | 0.00 (0.00, 5.07) |
| Urological | Ureteric and other urinary | 0.00 (0.00, 3.81) | 0.00 (0.00, 5.07) |
| Haematological | Acute leukaemia | 9.28 (4.96, 16.7) | 13.9 (7.72, 23.7) |
| Haematological | Chronic lymphocytic leukaemia | 5.15 (2.22, 11.5) | 1.39 (0.25, 7.46) |
| Haematological | Hodgkin lymphoma | 1.03 (0.18, 5.61) | 1.39 (0.25, 7.46) |
| Haematological | Multiple myeloma | 4.12 (1.62, 10.1) | 2.78 (0.77, 9.57) |
| Haematological | Non-Hodgkin lymphoma | 7.22 (3.54, 14.2) | 1.39 (0.25, 7.46) |
| Haematological | Other haematological | 5.15 (2.22, 11.5) | 5.56 (2.18, 13.4) |
| Central nervous system | Central nervous system | 0.00 (0.00, 3.81) | 4.17 (1.43, 11.5) |
| Sarcoma | Bone sarcoma | 0.00 (0.00, 3.81) | 1.39 (0.25, 7.46) |
| Sarcoma | Connective and soft tissue sarcoma | 0.00 (0.00, 3.81) | 0.00 (0.00, 5.07) |
| Skin | Melanoma | 5.15 (2.22, 11.5) | 1.39 (0.25, 7.46) |
| Ocular | Ocular | 0.00 (0.00, 3.81) | 0.00 (0.00, 5.07) |
| Breast | Breast | 0.00 (0.00, 3.81) | 8.33 (3.88, 17.0) |
| Gynaecological | Cervix | - | 0.00 (0.00, 5.07) |
| Gynaecological | Ovary | - | 4.17 (1.43, 11.5) |
| Gynaecological | Uterus | - | 1.39 (0.25, 7.46) |
| Gynaecological | Vulva/Vagina | - | 1.39 (0.25, 7.46) |
| Prostate and other male organs | Penile | 0.00 (0.00, 3.81) | - |
| Prostate and other male organs | Prostate | 11.3 (6.45, 19.2) | - |
| Prostate and other male organs | Testicular | 0.00 (0.00, 3.81) | - |
| Other malignant neoplasms | Other malignant neoplasms | 0.00 (0.00, 3.81) | 0.00 (0.00, 5.07) |
| Unknown primary | Unknown primary | 2.06 (0.57, 7.21) | 5.56 (2.18, 13.4) |
| *Non-specific - Night sweats (Men=219, Women=125)* | | | |
| Head and neck | Larynx | 0.00 (0.00, 1.72) | 0.00 (0.00, 2.98) |
| Head and neck | Oral cavity | 0.00 (0.00, 1.72) | 0.00 (0.00, 2.98) |
| Head and neck | Oropharynx | 0.46 (0.08, 2.54) | 0.80 (0.14, 4.39) |
| Head and neck | Thyroid | 0.46 (0.08, 2.54) | 0.00 (0.00, 2.98) |
| Head and neck | Other head and neck | 0.00 (0.00, 1.72) | 0.00 (0.00, 2.98) |
| Upper gastrointestinal | Oesophagus | 2.28 (0.98, 5.23) | 0.80 (0.14, 4.39) |
| Upper gastrointestinal | Stomach | 1.37 (0.47, 3.95) | 0.00 (0.00, 2.98) |
| Lower gastrointestinal | Anal | 0.00 (0.00, 1.72) | 0.00 (0.00, 2.98) |
| Lower gastrointestinal | Colon | 5.48 (3.16, 9.33) | 7.20 (3.83, 13.1) |
| Lower gastrointestinal | Rectum | 1.37 (0.47, 3.95) | 0.80 (0.14, 4.39) |
| Lower gastrointestinal | Small intestine | 0.00 (0.00, 1.72) | 0.80 (0.14, 4.39) |
| Hepato-pancreato-biliary | Liver | 0.91 (0.25, 3.27) | 0.80 (0.14, 4.39) |
| Hepato-pancreato-biliary | Pancreas | 2.74 (1.26, 5.85) | 4.00 (1.72, 9.02) |
| Hepato-pancreato-biliary | Other Hepato-pancreato-biliary | 0.00 (0.00, 1.72) | 1.60 (0.44, 5.65) |
| Respiratory | Lung | 12.8 (9.00, 17.9) | 20.0 (13.9, 27.9) |
| Respiratory | Mesothelioma | 1.37 (0.47, 3.95) | 0.00 (0.00, 2.98) |
| Urological | Bladder | 0.91 (0.25, 3.27) | 0.00 (0.00, 2.98) |
| Urological | Kidney | 5.48 (3.16, 9.33) | 4.80 (2.22, 10.1) |
| Urological | Ureteric and other urinary | 0.91 (0.25, 3.27) | 0.00 (0.00, 2.98) |
| Haematological | Acute leukaemia | 5.48 (3.16, 9.33) | 3.20 (1.25, 7.94) |
| Haematological | Chronic lymphocytic leukaemia | 3.20 (1.56, 6.45) | 5.60 (2.74, 11.1) |
| Haematological | Hodgkin lymphoma | 2.74 (1.26, 5.85) | 6.40 (3.28, 12.1) |
| Haematological | Multiple myeloma | 3.20 (1.56, 6.45) | 5.60 (2.74, 11.1) |
| Haematological | Non-Hodgkin lymphoma | 12.8 (9.00, 17.9) | 16.8 (11.3, 24.3) |
| Haematological | Other haematological | 8.68 (5.62, 13.2) | 4.80 (2.22, 10.1) |
| Central nervous system | Central nervous system | 0.91 (0.25, 3.27) | 0.80 (0.14, 4.39) |
| Sarcoma | Bone sarcoma | 0.00 (0.00, 1.72) | 0.00 (0.00, 2.98) |
| Sarcoma | Connective and soft tissue sarcoma | 0.46 (0.08, 2.54) | 0.00 (0.00, 2.98) |
| Skin | Melanoma | 0.46 (0.08, 2.54) | 0.00 (0.00, 2.98) |
| Ocular | Ocular | 0.00 (0.00, 1.72) | 0.00 (0.00, 2.98) |
| Breast | Breast | 0.00 (0.00, 1.72) | 4.80 (2.22, 10.1) |
| Gynaecological | Cervix | - | 0.00 (0.00, 2.98) |
| Gynaecological | Ovary | - | 7.20 (3.83, 13.1) |
| Gynaecological | Uterus | - | 0.80 (0.14, 4.39) |
| Gynaecological | Vulva/Vagina | - | 0.00 (0.00, 2.98) |
| Prostate and other male organs | Penile | 0.00 (0.00, 1.72) | - |
| Prostate and other male organs | Prostate | 19.2 (14.5, 24.9) | - |
| Prostate and other male organs | Testicular | 0.46 (0.08, 2.54) | - |
| Other malignant neoplasms | Other malignant neoplasms | 0.46 (0.08, 2.54) | 0.00 (0.00, 2.98) |
| Unknown primary | Unknown primary | 5.48 (3.16, 9.33) | 2.40 (0.82, 6.82) |
| *Non-specific - Other symptom (Men=1601, Women=1264)* | | | |
| Head and neck | Larynx | 0.69 (0.38, 1.23) | 0.24 (0.08, 0.70) |
| Head and neck | Oral cavity | 1.50 (1.01, 2.22) | 1.34 (0.84, 2.14) |
| Head and neck | Oropharynx | 2.44 (1.79, 3.31) | 0.87 (0.49, 1.55) |
| Head and neck | Thyroid | 0.69 (0.38, 1.23) | 2.29 (1.60, 3.28) |
| Head and neck | Other head and neck | 2.12 (1.52, 2.95) | 2.22 (1.54, 3.18) |
| Upper gastrointestinal | Oesophagus | 2.81 (2.11, 3.74) | 1.11 (0.66, 1.85) |
| Upper gastrointestinal | Stomach | 2.81 (2.11, 3.74) | 1.58 (1.03, 2.43) |
| Lower gastrointestinal | Anal | 0.31 (0.13, 0.73) | 0.71 (0.38, 1.35) |
| Lower gastrointestinal | Colon | 7.31 (6.13, 8.69) | 8.94 (7.49, 10.6) |
| Lower gastrointestinal | Rectum | 1.44 (0.96, 2.15) | 1.82 (1.22, 2.72) |
| Lower gastrointestinal | Small intestine | 0.25 (0.10, 0.64) | 0.32 (0.12, 0.81) |
| Hepato-pancreato-biliary | Liver | 2.12 (1.52, 2.95) | 2.22 (1.54, 3.18) |
| Hepato-pancreato-biliary | Pancreas | 3.25 (2.49, 4.23) | 4.98 (3.91, 6.33) |
| Hepato-pancreato-biliary | Other Hepato-pancreato-biliary | 0.69 (0.38, 1.23) | 1.27 (0.78, 2.05) |
| Respiratory | Lung | 12.7 (11.2, 14.5) | 20.3 (18.1, 22.6) |
| Respiratory | Mesothelioma | 0.94 (0.57, 1.54) | 0.32 (0.12, 0.81) |
| Urological | Bladder | 2.12 (1.52, 2.95) | 0.95 (0.54, 1.65) |
| Urological | Kidney | 2.19 (1.58, 3.03) | 3.40 (2.54, 4.55) |
| Urological | Ureteric and other urinary | 0.37 (0.17, 0.82) | 0.16 (0.04, 0.58) |
| Haematological | Acute leukaemia | 1.44 (0.96, 2.15) | 1.42 (0.90, 2.24) |
| Haematological | Chronic lymphocytic leukaemia | 2.12 (1.52, 2.95) | 1.58 (1.03, 2.43) |
| Haematological | Hodgkin lymphoma | 0.75 (0.43, 1.31) | 0.40 (0.17, 0.92) |
| Haematological | Multiple myeloma | 2.81 (2.11, 3.74) | 2.45 (1.73, 3.46) |
| Haematological | Non-Hodgkin lymphoma | 6.43 (5.33, 7.74) | 6.33 (5.11, 7.81) |
| Haematological | Other haematological | 3.06 (2.32, 4.02) | 2.37 (1.67, 3.37) |
| Central nervous system | Central nervous system | 4.12 (3.25, 5.21) | 5.38 (4.27, 6.76) |
| Sarcoma | Bone sarcoma | 0.37 (0.17, 0.82) | 0.08 (0.01, 0.45) |
| Sarcoma | Connective and soft tissue sarcoma | 1.25 (0.81, 1.92) | 1.82 (1.22, 2.72) |
| Skin | Melanoma | 2.62 (1.95, 3.53) | 3.64 (2.74, 4.82) |
| Ocular | Ocular | 0.25 (0.10, 0.64) | 0.95 (0.54, 1.65) |
| Breast | Breast | 0.00 (0.00, 0.24) | 6.17 (4.97, 7.63) |
| Gynaecological | Cervix | - | 0.47 (0.22, 1.03) |
| Gynaecological | Ovary | - | 4.35 (3.36, 5.62) |
| Gynaecological | Uterus | - | 1.82 (1.22, 2.72) |
| Gynaecological | Vulva/Vagina | - | 0.87 (0.49, 1.55) |
| Prostate and other male organs | Penile | 1.37 (0.91, 2.07) | - |
| Prostate and other male organs | Prostate | 22.5 (20.6, 24.7) | - |
| Prostate and other male organs | Testicular | 0.62 (0.34, 1.15) | - |
| Other malignant neoplasms | Other malignant neoplasms | 0.56 (0.30, 1.06) | 0.71 (0.38, 1.35) |
| Unknown primary | Unknown primary | 2.87 (2.16, 3.81) | 4.19 (3.22, 5.44) |
| *Non-specific - Pallor (Men=100, Women=62)* | | | |
| Head and neck | Larynx | 0.00 (0.00, 3.70) | 0.00 (0.00, 5.83) |
| Head and neck | Oral cavity | 0.00 (0.00, 3.70) | 0.00 (0.00, 5.83) |
| Head and neck | Oropharynx | 1.00 (0.18, 5.45) | 0.00 (0.00, 5.83) |
| Head and neck | Thyroid | 0.00 (0.00, 3.70) | 0.00 (0.00, 5.83) |
| Head and neck | Other head and neck | 1.00 (0.18, 5.45) | 0.00 (0.00, 5.83) |
| Upper gastrointestinal | Oesophagus | 2.00 (0.55, 7.00) | 0.00 (0.00, 5.83) |
| Upper gastrointestinal | Stomach | 7.00 (3.43, 13.7) | 16.1 (9.00, 27.2) |
| Lower gastrointestinal | Anal | 1.00 (0.18, 5.45) | 0.00 (0.00, 5.83) |
| Lower gastrointestinal | Colon | 20.0 (13.3, 28.9) | 22.6 (14.0, 34.4) |
| Lower gastrointestinal | Rectum | 4.00 (1.57, 9.84) | 3.23 (0.89, 11.0) |
| Lower gastrointestinal | Small intestine | 2.00 (0.55, 7.00) | 0.00 (0.00, 5.83) |
| Hepato-pancreato-biliary | Liver | 4.00 (1.57, 9.84) | 3.23 (0.89, 11.0) |
| Hepato-pancreato-biliary | Pancreas | 3.00 (1.03, 8.45) | 4.84 (1.66, 13.3) |
| Hepato-pancreato-biliary | Other Hepato-pancreato-biliary | 1.00 (0.18, 5.45) | 1.61 (0.29, 8.59) |
| Respiratory | Lung | 8.00 (4.11, 15.0) | 6.45 (2.54, 15.4) |
| Respiratory | Mesothelioma | 1.00 (0.18, 5.45) | 0.00 (0.00, 5.83) |
| Urological | Bladder | 1.00 (0.18, 5.45) | 0.00 (0.00, 5.83) |
| Urological | Kidney | 1.00 (0.18, 5.45) | 0.00 (0.00, 5.83) |
| Urological | Ureteric and other urinary | 0.00 (0.00, 3.70) | 1.61 (0.29, 8.59) |
| Haematological | Acute leukaemia | 10.0 (5.52, 17.4) | 6.45 (2.54, 15.4) |
| Haematological | Chronic lymphocytic leukaemia | 3.00 (1.03, 8.45) | 0.00 (0.00, 5.83) |
| Haematological | Hodgkin lymphoma | 0.00 (0.00, 3.70) | 0.00 (0.00, 5.83) |
| Haematological | Multiple myeloma | 2.00 (0.55, 7.00) | 3.23 (0.89, 11.0) |
| Haematological | Non-Hodgkin lymphoma | 6.00 (2.78, 12.5) | 3.23 (0.89, 11.0) |
| Haematological | Other haematological | 6.00 (2.78, 12.5) | 3.23 (0.89, 11.0) |
| Central nervous system | Central nervous system | 1.00 (0.18, 5.45) | 0.00 (0.00, 5.83) |
| Sarcoma | Bone sarcoma | 0.00 (0.00, 3.70) | 0.00 (0.00, 5.83) |
| Sarcoma | Connective and soft tissue sarcoma | 0.00 (0.00, 3.70) | 0.00 (0.00, 5.83) |
| Skin | Melanoma | 1.00 (0.18, 5.45) | 4.84 (1.66, 13.3) |
| Ocular | Ocular | 0.00 (0.00, 3.70) | 0.00 (0.00, 5.83) |
| Breast | Breast | 0.00 (0.00, 3.70) | 4.84 (1.66, 13.3) |
| Gynaecological | Cervix | - | 0.00 (0.00, 5.83) |
| Gynaecological | Ovary | - | 4.84 (1.66, 13.3) |
| Gynaecological | Uterus | - | 3.23 (0.89, 11.0) |
| Gynaecological | Vulva/Vagina | - | 0.00 (0.00, 5.83) |
| Prostate and other male organs | Penile | 0.00 (0.00, 3.70) | - |
| Prostate and other male organs | Prostate | 10.0 (5.52, 17.4) | - |
| Prostate and other male organs | Testicular | 1.00 (0.18, 5.45) | - |
| Other malignant neoplasms | Other malignant neoplasms | 0.00 (0.00, 3.70) | 1.61 (0.29, 8.59) |
| Unknown primary | Unknown primary | 3.00 (1.03, 8.45) | 4.84 (1.66, 13.3) |
| *Non-specific - Pruritus (Men=124, Women=155)* | | | |
| Head and neck | Larynx | 0.00 (0.00, 3.00) | 0.00 (0.00, 2.42) |
| Head and neck | Oral cavity | 0.00 (0.00, 3.00) | 0.00 (0.00, 2.42) |
| Head and neck | Oropharynx | 0.00 (0.00, 3.00) | 0.00 (0.00, 2.42) |
| Head and neck | Thyroid | 0.00 (0.00, 3.00) | 0.00 (0.00, 2.42) |
| Head and neck | Other head and neck | 0.00 (0.00, 3.00) | 0.00 (0.00, 2.42) |
| Upper gastrointestinal | Oesophagus | 0.81 (0.14, 4.43) | 0.65 (0.11, 3.56) |
| Upper gastrointestinal | Stomach | 0.81 (0.14, 4.43) | 0.65 (0.11, 3.56) |
| Lower gastrointestinal | Anal | 1.61 (0.44, 5.69) | 0.65 (0.11, 3.56) |
| Lower gastrointestinal | Colon | 5.65 (2.76, 11.2) | 3.23 (1.39, 7.33) |
| Lower gastrointestinal | Rectum | 2.42 (0.83, 6.87) | 0.00 (0.00, 2.42) |
| Lower gastrointestinal | Small intestine | 1.61 (0.44, 5.69) | 0.65 (0.11, 3.56) |
| Hepato-pancreato-biliary | Liver | 7.26 (3.87, 13.2) | 5.81 (3.08, 10.7) |
| Hepato-pancreato-biliary | Pancreas | 10.5 (6.23, 17.1) | 18.7 (13.4, 25.6) |
| Hepato-pancreato-biliary | Other Hepato-pancreato-biliary | 10.5 (6.23, 17.1) | 7.10 (4.01, 12.3) |
| Respiratory | Lung | 4.03 (1.73, 9.09) | 5.16 (2.64, 9.85) |
| Respiratory | Mesothelioma | 0.00 (0.00, 3.00) | 0.00 (0.00, 2.42) |
| Urological | Bladder | 0.00 (0.00, 3.00) | 0.00 (0.00, 2.42) |
| Urological | Kidney | 0.00 (0.00, 3.00) | 0.00 (0.00, 2.42) |
| Urological | Ureteric and other urinary | 0.00 (0.00, 3.00) | 0.00 (0.00, 2.42) |
| Haematological | Acute leukaemia | 1.61 (0.44, 5.69) | 1.29 (0.35, 4.58) |
| Haematological | Chronic lymphocytic leukaemia | 0.81 (0.14, 4.43) | 1.29 (0.35, 4.58) |
| Haematological | Hodgkin lymphoma | 0.81 (0.14, 4.43) | 3.23 (1.39, 7.33) |
| Haematological | Multiple myeloma | 4.03 (1.73, 9.09) | 3.23 (1.39, 7.33) |
| Haematological | Non-Hodgkin lymphoma | 5.65 (2.76, 11.2) | 5.81 (3.08, 10.7) |
| Haematological | Other haematological | 13.7 (8.74, 20.9) | 5.81 (3.08, 10.7) |
| Central nervous system | Central nervous system | 0.81 (0.14, 4.43) | 0.00 (0.00, 2.42) |
| Sarcoma | Bone sarcoma | 0.00 (0.00, 3.00) | 0.00 (0.00, 2.42) |
| Sarcoma | Connective and soft tissue sarcoma | 1.61 (0.44, 5.69) | 0.65 (0.11, 3.56) |
| Skin | Melanoma | 13.7 (8.74, 20.9) | 14.8 (10.1, 21.3) |
| Ocular | Ocular | 0.00 (0.00, 3.00) | 0.00 (0.00, 2.42) |
| Breast | Breast | 0.00 (0.00, 3.00) | 10.3 (6.45, 16.1) |
| Gynaecological | Cervix | - | 0.00 (0.00, 2.42) |
| Gynaecological | Ovary | - | 1.94 (0.66, 5.54) |
| Gynaecological | Uterus | - | 0.65 (0.11, 3.56) |
| Gynaecological | Vulva/Vagina | - | 5.81 (3.08, 10.7) |
| Prostate and other male organs | Penile | 2.42 (0.83, 6.87) | - |
| Prostate and other male organs | Prostate | 3.23 (1.26, 8.00) | - |
| Prostate and other male organs | Testicular | 0.00 (0.00, 3.00) | - |
| Other malignant neoplasms | Other malignant neoplasms | 3.23 (1.26, 8.00) | 0.00 (0.00, 2.42) |
| Unknown primary | Unknown primary | 3.23 (1.26, 8.00) | 2.58 (1.01, 6.45) |
| *Non-specific - Weight loss (Men=2430, Women=1706)* | | | |
| Head and neck | Larynx | 0.78 (0.50, 1.22) | 0.18 (0.06, 0.52) |
| Head and neck | Oral cavity | 0.41 (0.22, 0.76) | 0.12 (0.03, 0.43) |
| Head and neck | Oropharynx | 0.78 (0.50, 1.22) | 0.59 (0.32, 1.08) |
| Head and neck | Thyroid | 0.16 (0.06, 0.42) | 0.53 (0.28, 1.00) |
| Head and neck | Other head and neck | 0.58 (0.34, 0.96) | 0.18 (0.06, 0.52) |
| Upper gastrointestinal | Oesophagus | 8.52 (7.47, 9.69) | 4.87 (3.94, 5.99) |
| Upper gastrointestinal | Stomach | 5.51 (4.68, 6.49) | 4.63 (3.73, 5.73) |
| Lower gastrointestinal | Anal | 0.12 (0.04, 0.36) | 0.64 (0.36, 1.15) |
| Lower gastrointestinal | Colon | 11.4 (10.2, 12.7) | 15.8 (14.2, 17.6) |
| Lower gastrointestinal | Rectum | 5.06 (4.26, 6.01) | 3.17 (2.43, 4.11) |
| Lower gastrointestinal | Small intestine | 0.66 (0.41, 1.07) | 1.17 (0.76, 1.80) |
| Hepato-pancreato-biliary | Liver | 3.33 (2.69, 4.12) | 2.29 (1.68, 3.11) |
| Hepato-pancreato-biliary | Pancreas | 8.68 (7.63, 9.87) | 10.0 (8.69, 11.5) |
| Hepato-pancreato-biliary | Other Hepato-pancreato-biliary | 0.78 (0.50, 1.22) | 1.88 (1.33, 2.64) |
| Respiratory | Lung | 20.4 (18.9, 22.1) | 22.2 (20.3, 24.2) |
| Respiratory | Mesothelioma | 1.81 (1.35, 2.42) | 0.35 (0.16, 0.77) |
| Urological | Bladder | 0.99 (0.66, 1.47) | 0.88 (0.53, 1.45) |
| Urological | Kidney | 3.21 (2.58, 3.99) | 2.75 (2.08, 3.64) |
| Urological | Ureteric and other urinary | 0.29 (0.14, 0.59) | 0.41 (0.20, 0.84) |
| Haematological | Acute leukaemia | 1.03 (0.70, 1.51) | 0.88 (0.53, 1.45) |
| Haematological | Chronic lymphocytic leukaemia | 0.62 (0.37, 1.02) | 0.41 (0.20, 0.84) |
| Haematological | Hodgkin lymphoma | 0.70 (0.44, 1.12) | 0.59 (0.32, 1.08) |
| Haematological | Multiple myeloma | 1.81 (1.35, 2.42) | 1.47 (0.99, 2.15) |
| Haematological | Non-Hodgkin lymphoma | 4.44 (3.69, 5.34) | 4.16 (3.31, 5.22) |
| Haematological | Other haematological | 1.19 (0.83, 1.71) | 1.06 (0.67, 1.66) |
| Central nervous system | Central nervous system | 0.41 (0.22, 0.76) | 0.12 (0.03, 0.43) |
| Sarcoma | Bone sarcoma | 0.04 (0.01, 0.23) | 0.00 (0.00, 0.22) |
| Sarcoma | Connective and soft tissue sarcoma | 0.16 (0.06, 0.42) | 0.41 (0.20, 0.84) |
| Skin | Melanoma | 0.21 (0.09, 0.48) | 0.06 (0.01, 0.33) |
| Ocular | Ocular | 0.00 (0.00, 0.16) | 0.00 (0.00, 0.22) |
| Breast | Breast | 0.08 (0.02, 0.30) | 4.87 (3.94, 5.99) |
| Gynaecological | Cervix | - | 0.41 (0.20, 0.84) |
| Gynaecological | Ovary | - | 6.21 (5.16, 7.46) |
| Gynaecological | Uterus | - | 1.88 (1.33, 2.64) |
| Gynaecological | Vulva/Vagina | - | 0.06 (0.01, 0.33) |
| Prostate and other male organs | Penile | 0.04 (0.01, 0.23) | - |
| Prostate and other male organs | Prostate | 11.5 (10.3, 12.8) | - |
| Prostate and other male organs | Testicular | 0.21 (0.09, 0.48) | - |
| Other malignant neoplasms | Other malignant neoplasms | 0.62 (0.37, 1.02) | 0.23 (0.09, 0.60) |
| Unknown primary | Unknown primary | 3.46 (2.80, 4.26) | 4.57 (3.68, 5.67) |
| *Lump/mass/lymph node - Anal mass (Men=46, Women=65)* | | | |
| Head and neck | Larynx | 0.00 (0.00, 7.71) | 0.00 (0.00, 5.58) |
| Head and neck | Oral cavity | 0.00 (0.00, 7.71) | 0.00 (0.00, 5.58) |
| Head and neck | Oropharynx | 0.00 (0.00, 7.71) | 0.00 (0.00, 5.58) |
| Head and neck | Thyroid | 0.00 (0.00, 7.71) | 0.00 (0.00, 5.58) |
| Head and neck | Other head and neck | 0.00 (0.00, 7.71) | 0.00 (0.00, 5.58) |
| Upper gastrointestinal | Oesophagus | 0.00 (0.00, 7.71) | 0.00 (0.00, 5.58) |
| Upper gastrointestinal | Stomach | 0.00 (0.00, 7.71) | 0.00 (0.00, 5.58) |
| Lower gastrointestinal | Anal | 45.7 (32.2, 59.8) | 72.3 (60.4, 81.7) |
| Lower gastrointestinal | Colon | 10.9 (4.73, 23.0) | 6.15 (2.42, 14.8) |
| Lower gastrointestinal | Rectum | 26.1 (15.6, 40.3) | 16.9 (9.72, 27.8) |
| Lower gastrointestinal | Small intestine | 0.00 (0.00, 7.71) | 0.00 (0.00, 5.58) |
| Hepato-pancreato-biliary | Liver | 0.00 (0.00, 7.71) | 0.00 (0.00, 5.58) |
| Hepato-pancreato-biliary | Pancreas | 0.00 (0.00, 7.71) | 0.00 (0.00, 5.58) |
| Hepato-pancreato-biliary | Other Hepato-pancreato-biliary | 0.00 (0.00, 7.71) | 0.00 (0.00, 5.58) |
| Respiratory | Lung | 2.17 (0.38, 11.3) | 0.00 (0.00, 5.58) |
| Respiratory | Mesothelioma | 0.00 (0.00, 7.71) | 0.00 (0.00, 5.58) |
| Urological | Bladder | 0.00 (0.00, 7.71) | 0.00 (0.00, 5.58) |
| Urological | Kidney | 0.00 (0.00, 7.71) | 0.00 (0.00, 5.58) |
| Urological | Ureteric and other urinary | 0.00 (0.00, 7.71) | 0.00 (0.00, 5.58) |
| Haematological | Acute leukaemia | 2.17 (0.38, 11.3) | 0.00 (0.00, 5.58) |
| Haematological | Chronic lymphocytic leukaemia | 2.17 (0.38, 11.3) | 0.00 (0.00, 5.58) |
| Haematological | Hodgkin lymphoma | 0.00 (0.00, 7.71) | 0.00 (0.00, 5.58) |
| Haematological | Multiple myeloma | 0.00 (0.00, 7.71) | 0.00 (0.00, 5.58) |
| Haematological | Non-Hodgkin lymphoma | 0.00 (0.00, 7.71) | 1.54 (0.27, 8.21) |
| Haematological | Other haematological | 0.00 (0.00, 7.71) | 0.00 (0.00, 5.58) |
| Central nervous system | Central nervous system | 0.00 (0.00, 7.71) | 0.00 (0.00, 5.58) |
| Sarcoma | Bone sarcoma | 0.00 (0.00, 7.71) | 0.00 (0.00, 5.58) |
| Sarcoma | Connective and soft tissue sarcoma | 2.17 (0.38, 11.3) | 1.54 (0.27, 8.21) |
| Skin | Melanoma | 0.00 (0.00, 7.71) | 0.00 (0.00, 5.58) |
| Ocular | Ocular | 0.00 (0.00, 7.71) | 0.00 (0.00, 5.58) |
| Breast | Breast | 0.00 (0.00, 7.71) | 1.54 (0.27, 8.21) |
| Gynaecological | Cervix | - | 0.00 (0.00, 5.58) |
| Gynaecological | Ovary | - | 0.00 (0.00, 5.58) |
| Gynaecological | Uterus | - | 0.00 (0.00, 5.58) |
| Gynaecological | Vulva/Vagina | - | 0.00 (0.00, 5.58) |
| Prostate and other male organs | Penile | 0.00 (0.00, 7.71) | - |
| Prostate and other male organs | Prostate | 4.35 (1.20, 14.5) | - |
| Prostate and other male organs | Testicular | 0.00 (0.00, 7.71) | - |
| Other malignant neoplasms | Other malignant neoplasms | 0.00 (0.00, 7.71) | 0.00 (0.00, 5.58) |
| Unknown primary | Unknown primary | 4.35 (1.20, 14.5) | 0.00 (0.00, 5.58) |
| *Lump/mass/lymph node - Lymph node pain with alcohol (Men=N<20, Women=N<20)* | | | |
| Head and neck | Larynx | - | - |
| Head and neck | Oral cavity | - | - |
| Head and neck | Oropharynx | - | - |
| Head and neck | Thyroid | - | - |
| Head and neck | Other head and neck | - | - |
| Upper gastrointestinal | Oesophagus | - | - |
| Upper gastrointestinal | Stomach | - | - |
| Lower gastrointestinal | Anal | - | - |
| Lower gastrointestinal | Colon | - | - |
| Lower gastrointestinal | Rectum | - | - |
| Lower gastrointestinal | Small intestine | - | - |
| Hepato-pancreato-biliary | Liver | - | - |
| Hepato-pancreato-biliary | Pancreas | - | - |
| Hepato-pancreato-biliary | Other Hepato-pancreato-biliary | - | - |
| Respiratory | Lung | - | - |
| Respiratory | Mesothelioma | - | - |
| Urological | Bladder | - | - |
| Urological | Kidney | - | - |
| Urological | Ureteric and other urinary | - | - |
| Haematological | Acute leukaemia | - | - |
| Haematological | Chronic lymphocytic leukaemia | - | - |
| Haematological | Hodgkin lymphoma | - | - |
| Haematological | Multiple myeloma | - | - |
| Haematological | Non-Hodgkin lymphoma | - | - |
| Haematological | Other haematological | - | - |
| Central nervous system | Central nervous system | - | - |
| Sarcoma | Bone sarcoma | - | - |
| Sarcoma | Connective and soft tissue sarcoma | - | - |
| Skin | Melanoma | - | - |
| Ocular | Ocular | - | - |
| Breast | Breast | - | - |
| Gynaecological | Cervix | - | - |
| Gynaecological | Ovary | - | - |
| Gynaecological | Uterus | - | - |
| Gynaecological | Vulva/Vagina | - | - |
| Prostate and other male organs | Penile | - | - |
| Prostate and other male organs | Prostate | - | - |
| Prostate and other male organs | Testicular | - | - |
| Other malignant neoplasms | Other malignant neoplasms | - | - |
| Unknown primary | Unknown primary | - | - |
| *Lump/mass/lymph node - Lymphadenopathy (generalised) (Men=N<20, Women=20)* | | | |
| Head and neck | Larynx | - | 0.00 (0.00, 16.1) |
| Head and neck | Oral cavity | - | 0.00 (0.00, 16.1) |
| Head and neck | Oropharynx | - | 5.00 (0.89, 23.6) |
| Head and neck | Thyroid | - | 5.00 (0.89, 23.6) |
| Head and neck | Other head and neck | - | 0.00 (0.00, 16.1) |
| Upper gastrointestinal | Oesophagus | - | 0.00 (0.00, 16.1) |
| Upper gastrointestinal | Stomach | - | 0.00 (0.00, 16.1) |
| Lower gastrointestinal | Anal | - | 0.00 (0.00, 16.1) |
| Lower gastrointestinal | Colon | - | 0.00 (0.00, 16.1) |
| Lower gastrointestinal | Rectum | - | 0.00 (0.00, 16.1) |
| Lower gastrointestinal | Small intestine | - | 0.00 (0.00, 16.1) |
| Hepato-pancreato-biliary | Liver | - | 5.00 (0.89, 23.6) |
| Hepato-pancreato-biliary | Pancreas | - | 0.00 (0.00, 16.1) |
| Hepato-pancreato-biliary | Other Hepato-pancreato-biliary | - | 0.00 (0.00, 16.1) |
| Respiratory | Lung | - | 5.00 (0.89, 23.6) |
| Respiratory | Mesothelioma | - | 0.00 (0.00, 16.1) |
| Urological | Bladder | - | 0.00 (0.00, 16.1) |
| Urological | Kidney | - | 5.00 (0.89, 23.6) |
| Urological | Ureteric and other urinary | - | 0.00 (0.00, 16.1) |
| Haematological | Acute leukaemia | - | 0.00 (0.00, 16.1) |
| Haematological | Chronic lymphocytic leukaemia | - | 0.00 (0.00, 16.1) |
| Haematological | Hodgkin lymphoma | - | 5.00 (0.89, 23.6) |
| Haematological | Multiple myeloma | - | 0.00 (0.00, 16.1) |
| Haematological | Non-Hodgkin lymphoma | - | 45.0 (25.8, 65.8) |
| Haematological | Other haematological | - | 0.00 (0.00, 16.1) |
| Central nervous system | Central nervous system | - | 0.00 (0.00, 16.1) |
| Sarcoma | Bone sarcoma | - | 0.00 (0.00, 16.1) |
| Sarcoma | Connective and soft tissue sarcoma | - | 0.00 (0.00, 16.1) |
| Skin | Melanoma | - | 5.00 (0.89, 23.6) |
| Ocular | Ocular | - | 0.00 (0.00, 16.1) |
| Breast | Breast | - | 10.0 (2.79, 30.1) |
| Gynaecological | Cervix | - | 0.00 (0.00, 16.1) |
| Gynaecological | Ovary | - | 0.00 (0.00, 16.1) |
| Gynaecological | Uterus | - | 5.00 (0.89, 23.6) |
| Gynaecological | Vulva/Vagina | - | 0.00 (0.00, 16.1) |
| Prostate and other male organs | Penile | - | - |
| Prostate and other male organs | Prostate | - | - |
| Prostate and other male organs | Testicular | - | - |
| Other malignant neoplasms | Other malignant neoplasms | - | 5.00 (0.89, 23.6) |
| Unknown primary | Unknown primary | - | 0.00 (0.00, 16.1) |
| *Lump/mass/lymph node - Lymphadenopathy (localised) (Men=220, Women=168)* | | | |
| Head and neck | Larynx | 1.82 (0.71, 4.58) | 0.60 (0.11, 3.29) |
| Head and neck | Oral cavity | 2.27 (0.97, 5.21) | 1.79 (0.61, 5.12) |
| Head and neck | Oropharynx | 10.9 (7.44, 15.7) | 6.55 (3.70, 11.3) |
| Head and neck | Thyroid | 0.00 (0.00, 1.72) | 1.79 (0.61, 5.12) |
| Head and neck | Other head and neck | 5.45 (3.15, 9.29) | 5.36 (2.84, 9.87) |
| Upper gastrointestinal | Oesophagus | 1.36 (0.46, 3.93) | 0.00 (0.00, 2.24) |
| Upper gastrointestinal | Stomach | 0.45 (0.08, 2.53) | 0.00 (0.00, 2.24) |
| Lower gastrointestinal | Anal | 0.45 (0.08, 2.53) | 0.60 (0.11, 3.29) |
| Lower gastrointestinal | Colon | 0.91 (0.25, 3.25) | 1.19 (0.33, 4.24) |
| Lower gastrointestinal | Rectum | 0.45 (0.08, 2.53) | 0.00 (0.00, 2.24) |
| Lower gastrointestinal | Small intestine | 0.00 (0.00, 1.72) | 0.00 (0.00, 2.24) |
| Hepato-pancreato-biliary | Liver | 0.00 (0.00, 1.72) | 0.00 (0.00, 2.24) |
| Hepato-pancreato-biliary | Pancreas | 0.00 (0.00, 1.72) | 1.19 (0.33, 4.24) |
| Hepato-pancreato-biliary | Other Hepato-pancreato-biliary | 0.00 (0.00, 1.72) | 0.00 (0.00, 2.24) |
| Respiratory | Lung | 2.73 (1.26, 5.82) | 5.95 (3.27, 10.6) |
| Respiratory | Mesothelioma | 0.45 (0.08, 2.53) | 0.00 (0.00, 2.24) |
| Urological | Bladder | 0.00 (0.00, 1.72) | 1.19 (0.33, 4.24) |
| Urological | Kidney | 0.45 (0.08, 2.53) | 0.00 (0.00, 2.24) |
| Urological | Ureteric and other urinary | 0.00 (0.00, 1.72) | 0.00 (0.00, 2.24) |
| Haematological | Acute leukaemia | 0.91 (0.25, 3.25) | 0.60 (0.11, 3.29) |
| Haematological | Chronic lymphocytic leukaemia | 5.45 (3.15, 9.29) | 2.38 (0.93, 5.96) |
| Haematological | Hodgkin lymphoma | 6.82 (4.18, 10.9) | 8.33 (5.03, 13.5) |
| Haematological | Multiple myeloma | 0.91 (0.25, 3.25) | 0.00 (0.00, 2.24) |
| Haematological | Non-Hodgkin lymphoma | 39.5 (33.3, 46.1) | 39.3 (32.2, 46.8) |
| Haematological | Other haematological | 5.00 (2.81, 8.73) | 3.57 (1.65, 7.57) |
| Central nervous system | Central nervous system | 0.00 (0.00, 1.72) | 0.00 (0.00, 2.24) |
| Sarcoma | Bone sarcoma | 0.00 (0.00, 1.72) | 0.60 (0.11, 3.29) |
| Sarcoma | Connective and soft tissue sarcoma | 0.45 (0.08, 2.53) | 0.60 (0.11, 3.29) |
| Skin | Melanoma | 2.27 (0.97, 5.21) | 0.60 (0.11, 3.29) |
| Ocular | Ocular | 0.00 (0.00, 1.72) | 0.00 (0.00, 2.24) |
| Breast | Breast | 0.00 (0.00, 1.72) | 7.74 (4.58, 12.8) |
| Gynaecological | Cervix | - | 0.60 (0.11, 3.29) |
| Gynaecological | Ovary | - | 2.38 (0.93, 5.96) |
| Gynaecological | Uterus | - | 0.00 (0.00, 2.24) |
| Gynaecological | Vulva/Vagina | - | 2.38 (0.93, 5.96) |
| Prostate and other male organs | Penile | 1.36 (0.46, 3.93) | - |
| Prostate and other male organs | Prostate | 2.73 (1.26, 5.82) | - |
| Prostate and other male organs | Testicular | 0.45 (0.08, 2.53) | - |
| Other malignant neoplasms | Other malignant neoplasms | 0.00 (0.00, 1.72) | 0.00 (0.00, 2.24) |
| Unknown primary | Unknown primary | 6.36 (3.83, 10.4) | 4.76 (2.43, 9.11) |
| *Lump/mass/lymph node - Neck lump/mass (Men=753, Women=550)* | | | |
| Head and neck | Larynx | 2.12 (1.31, 3.42) | 0.91 (0.39, 2.11) |
| Head and neck | Oral cavity | 2.79 (1.83, 4.23) | 2.00 (1.12, 3.55) |
| Head and neck | Oropharynx | 30.4 (27.2, 33.8) | 9.64 (7.44, 12.4) |
| Head and neck | Thyroid | 7.04 (5.42, 9.09) | 28.4 (24.8, 32.3) |
| Head and neck | Other head and neck | 11.4 (9.34, 13.9) | 11.1 (8.73, 14.0) |
| Upper gastrointestinal | Oesophagus | 0.66 (0.28, 1.54) | 0.18 (0.03, 1.02) |
| Upper gastrointestinal | Stomach | 0.53 (0.21, 1.36) | 0.00 (0.00, 0.69) |
| Lower gastrointestinal | Anal | 0.00 (0.00, 0.51) | 0.00 (0.00, 0.69) |
| Lower gastrointestinal | Colon | 0.40 (0.14, 1.16) | 0.18 (0.03, 1.02) |
| Lower gastrointestinal | Rectum | 0.27 (0.07, 0.96) | 0.00 (0.00, 0.69) |
| Lower gastrointestinal | Small intestine | 0.00 (0.00, 0.51) | 0.18 (0.03, 1.02) |
| Hepato-pancreato-biliary | Liver | 0.00 (0.00, 0.51) | 0.55 (0.19, 1.59) |
| Hepato-pancreato-biliary | Pancreas | 0.00 (0.00, 0.51) | 0.00 (0.00, 0.69) |
| Hepato-pancreato-biliary | Other Hepato-pancreato-biliary | 0.00 (0.00, 0.51) | 0.00 (0.00, 0.69) |
| Respiratory | Lung | 4.25 (3.03, 5.94) | 6.18 (4.46, 8.51) |
| Respiratory | Mesothelioma | 0.00 (0.00, 0.51) | 0.00 (0.00, 0.69) |
| Urological | Bladder | 0.00 (0.00, 0.51) | 0.00 (0.00, 0.69) |
| Urological | Kidney | 0.80 (0.37, 1.73) | 0.55 (0.19, 1.59) |
| Urological | Ureteric and other urinary | 0.13 (0.02, 0.75) | 0.00 (0.00, 0.69) |
| Haematological | Acute leukaemia | 0.66 (0.28, 1.54) | 0.36 (0.10, 1.32) |
| Haematological | Chronic lymphocytic leukaemia | 2.26 (1.41, 3.59) | 1.45 (0.74, 2.84) |
| Haematological | Hodgkin lymphoma | 4.38 (3.14, 6.09) | 6.55 (4.77, 8.93) |
| Haematological | Multiple myeloma | 0.40 (0.14, 1.16) | 0.55 (0.19, 1.59) |
| Haematological | Non-Hodgkin lymphoma | 19.8 (17.1, 22.8) | 22.0 (18.7, 25.6) |
| Haematological | Other haematological | 1.59 (0.91, 2.76) | 1.82 (0.99, 3.31) |
| Central nervous system | Central nervous system | 0.13 (0.02, 0.75) | 0.00 (0.00, 0.69) |
| Sarcoma | Bone sarcoma | 0.00 (0.00, 0.51) | 0.00 (0.00, 0.69) |
| Sarcoma | Connective and soft tissue sarcoma | 0.53 (0.21, 1.36) | 0.36 (0.10, 1.32) |
| Skin | Melanoma | 0.93 (0.45, 1.91) | 0.73 (0.28, 1.85) |
| Ocular | Ocular | 0.00 (0.00, 0.51) | 0.00 (0.00, 0.69) |
| Breast | Breast | 0.00 (0.00, 0.51) | 1.82 (0.99, 3.31) |
| Gynaecological | Cervix | - | 0.00 (0.00, 0.69) |
| Gynaecological | Ovary | - | 0.55 (0.19, 1.59) |
| Gynaecological | Uterus | - | 0.00 (0.00, 0.69) |
| Gynaecological | Vulva/Vagina | - | 0.00 (0.00, 0.69) |
| Prostate and other male organs | Penile | 0.00 (0.00, 0.51) | - |
| Prostate and other male organs | Prostate | 0.53 (0.21, 1.36) | - |
| Prostate and other male organs | Testicular | 0.13 (0.02, 0.75) | - |
| Other malignant neoplasms | Other malignant neoplasms | 0.53 (0.21, 1.36) | 0.00 (0.00, 0.69) |
| Unknown primary | Unknown primary | 7.30 (5.65, 9.39) | 4.00 (2.66, 5.98) |
| *Lump/mass/lymph node - Thyroid lump/mass (Men=35, Women=120)* | | | |
| Head and neck | Larynx | 2.86 (0.51, 14.5) | 0.00 (0.00, 3.10) |
| Head and neck | Oral cavity | 0.00 (0.00, 9.89) | 0.00 (0.00, 3.10) |
| Head and neck | Oropharynx | 0.00 (0.00, 9.89) | 0.00 (0.00, 3.10) |
| Head and neck | Thyroid | 82.9 (67.3, 91.9) | 96.7 (91.7, 98.7) |
| Head and neck | Other head and neck | 0.00 (0.00, 9.89) | 0.00 (0.00, 3.10) |
| Upper gastrointestinal | Oesophagus | 0.00 (0.00, 9.89) | 0.00 (0.00, 3.10) |
| Upper gastrointestinal | Stomach | 2.86 (0.51, 14.5) | 0.00 (0.00, 3.10) |
| Lower gastrointestinal | Anal | 0.00 (0.00, 9.89) | 0.00 (0.00, 3.10) |
| Lower gastrointestinal | Colon | 0.00 (0.00, 9.89) | 0.00 (0.00, 3.10) |
| Lower gastrointestinal | Rectum | 0.00 (0.00, 9.89) | 0.00 (0.00, 3.10) |
| Lower gastrointestinal | Small intestine | 0.00 (0.00, 9.89) | 0.00 (0.00, 3.10) |
| Hepato-pancreato-biliary | Liver | 0.00 (0.00, 9.89) | 0.00 (0.00, 3.10) |
| Hepato-pancreato-biliary | Pancreas | 0.00 (0.00, 9.89) | 0.00 (0.00, 3.10) |
| Hepato-pancreato-biliary | Other Hepato-pancreato-biliary | 0.00 (0.00, 9.89) | 0.00 (0.00, 3.10) |
| Respiratory | Lung | 2.86 (0.51, 14.5) | 0.00 (0.00, 3.10) |
| Respiratory | Mesothelioma | 0.00 (0.00, 9.89) | 0.00 (0.00, 3.10) |
| Urological | Bladder | 0.00 (0.00, 9.89) | 0.00 (0.00, 3.10) |
| Urological | Kidney | 0.00 (0.00, 9.89) | 0.00 (0.00, 3.10) |
| Urological | Ureteric and other urinary | 0.00 (0.00, 9.89) | 0.00 (0.00, 3.10) |
| Haematological | Acute leukaemia | 0.00 (0.00, 9.89) | 0.00 (0.00, 3.10) |
| Haematological | Chronic lymphocytic leukaemia | 2.86 (0.51, 14.5) | 0.00 (0.00, 3.10) |
| Haematological | Hodgkin lymphoma | 0.00 (0.00, 9.89) | 0.00 (0.00, 3.10) |
| Haematological | Multiple myeloma | 0.00 (0.00, 9.89) | 0.00 (0.00, 3.10) |
| Haematological | Non-Hodgkin lymphoma | 2.86 (0.51, 14.5) | 1.67 (0.46, 5.87) |
| Haematological | Other haematological | 0.00 (0.00, 9.89) | 0.00 (0.00, 3.10) |
| Central nervous system | Central nervous system | 0.00 (0.00, 9.89) | 0.00 (0.00, 3.10) |
| Sarcoma | Bone sarcoma | 0.00 (0.00, 9.89) | 0.00 (0.00, 3.10) |
| Sarcoma | Connective and soft tissue sarcoma | 0.00 (0.00, 9.89) | 0.00 (0.00, 3.10) |
| Skin | Melanoma | 0.00 (0.00, 9.89) | 0.00 (0.00, 3.10) |
| Ocular | Ocular | 0.00 (0.00, 9.89) | 0.00 (0.00, 3.10) |
| Breast | Breast | 0.00 (0.00, 9.89) | 0.00 (0.00, 3.10) |
| Gynaecological | Cervix | - | 0.00 (0.00, 3.10) |
| Gynaecological | Ovary | - | 0.00 (0.00, 3.10) |
| Gynaecological | Uterus | - | 0.83 (0.15, 4.57) |
| Gynaecological | Vulva/Vagina | - | 0.00 (0.00, 3.10) |
| Prostate and other male organs | Penile | 2.86 (0.51, 14.5) | - |
| Prostate and other male organs | Prostate | 0.00 (0.00, 9.89) | - |
| Prostate and other male organs | Testicular | 0.00 (0.00, 9.89) | - |
| Other malignant neoplasms | Other malignant neoplasms | 0.00 (0.00, 9.89) | 0.00 (0.00, 3.10) |
| Unknown primary | Unknown primary | 0.00 (0.00, 9.89) | 0.83 (0.15, 4.57) |
| *Lump/mass/lymph node - Unexplained lump suspicious of sarcoma (Men=106, Women=82)* | | | |
| Head and neck | Larynx | 0.00 (0.00, 3.50) | 0.00 (0.00, 4.48) |
| Head and neck | Oral cavity | 0.94 (0.17, 5.15) | 1.22 (0.22, 6.59) |
| Head and neck | Oropharynx | 0.00 (0.00, 3.50) | 0.00 (0.00, 4.48) |
| Head and neck | Thyroid | 0.94 (0.17, 5.15) | 0.00 (0.00, 4.48) |
| Head and neck | Other head and neck | 0.94 (0.17, 5.15) | 1.22 (0.22, 6.59) |
| Upper gastrointestinal | Oesophagus | 0.00 (0.00, 3.50) | 0.00 (0.00, 4.48) |
| Upper gastrointestinal | Stomach | 0.94 (0.17, 5.15) | 0.00 (0.00, 4.48) |
| Lower gastrointestinal | Anal | 0.00 (0.00, 3.50) | 0.00 (0.00, 4.48) |
| Lower gastrointestinal | Colon | 1.89 (0.52, 6.62) | 1.22 (0.22, 6.59) |
| Lower gastrointestinal | Rectum | 0.00 (0.00, 3.50) | 0.00 (0.00, 4.48) |
| Lower gastrointestinal | Small intestine | 0.00 (0.00, 3.50) | 0.00 (0.00, 4.48) |
| Hepato-pancreato-biliary | Liver | 0.94 (0.17, 5.15) | 0.00 (0.00, 4.48) |
| Hepato-pancreato-biliary | Pancreas | 0.00 (0.00, 3.50) | 0.00 (0.00, 4.48) |
| Hepato-pancreato-biliary | Other Hepato-pancreato-biliary | 0.00 (0.00, 3.50) | 0.00 (0.00, 4.48) |
| Respiratory | Lung | 3.77 (1.48, 9.30) | 1.22 (0.22, 6.59) |
| Respiratory | Mesothelioma | 0.00 (0.00, 3.50) | 0.00 (0.00, 4.48) |
| Urological | Bladder | 0.00 (0.00, 3.50) | 0.00 (0.00, 4.48) |
| Urological | Kidney | 1.89 (0.52, 6.62) | 0.00 (0.00, 4.48) |
| Urological | Ureteric and other urinary | 0.00 (0.00, 3.50) | 0.00 (0.00, 4.48) |
| Haematological | Acute leukaemia | 0.00 (0.00, 3.50) | 0.00 (0.00, 4.48) |
| Haematological | Chronic lymphocytic leukaemia | 1.89 (0.52, 6.62) | 0.00 (0.00, 4.48) |
| Haematological | Hodgkin lymphoma | 0.94 (0.17, 5.15) | 1.22 (0.22, 6.59) |
| Haematological | Multiple myeloma | 0.00 (0.00, 3.50) | 1.22 (0.22, 6.59) |
| Haematological | Non-Hodgkin lymphoma | 15.1 (9.51, 23.1) | 15.9 (9.51, 25.3) |
| Haematological | Other haematological | 0.94 (0.17, 5.15) | 1.22 (0.22, 6.59) |
| Central nervous system | Central nervous system | 0.00 (0.00, 3.50) | 0.00 (0.00, 4.48) |
| Sarcoma | Bone sarcoma | 5.66 (2.62, 11.8) | 1.22 (0.22, 6.59) |
| Sarcoma | Connective and soft tissue sarcoma | 56.6 (47.1, 65.6) | 57.3 (46.5, 67.5) |
| Skin | Melanoma | 1.89 (0.52, 6.62) | 7.32 (3.40, 15.1) |
| Ocular | Ocular | 0.00 (0.00, 3.50) | 0.00 (0.00, 4.48) |
| Breast | Breast | 0.00 (0.00, 3.50) | 1.22 (0.22, 6.59) |
| Gynaecological | Cervix | - | 0.00 (0.00, 4.48) |
| Gynaecological | Ovary | - | 1.22 (0.22, 6.59) |
| Gynaecological | Uterus | - | 0.00 (0.00, 4.48) |
| Gynaecological | Vulva/Vagina | - | 0.00 (0.00, 4.48) |
| Prostate and other male organs | Penile | 0.00 (0.00, 3.50) | - |
| Prostate and other male organs | Prostate | 0.94 (0.17, 5.15) | - |
| Prostate and other male organs | Testicular | 0.00 (0.00, 3.50) | - |
| Other malignant neoplasms | Other malignant neoplasms | 1.89 (0.52, 6.62) | 2.44 (0.67, 8.46) |
| Unknown primary | Unknown primary | 1.89 (0.52, 6.62) | 4.88 (1.91, 11.9) |
| *Ulceration - Leukoplakia (Men=N<20, Women=N<20)* | | | |
| Head and neck | Larynx | - | - |
| Head and neck | Oral cavity | - | - |
| Head and neck | Oropharynx | - | - |
| Head and neck | Thyroid | - | - |
| Head and neck | Other head and neck | - | - |
| Upper gastrointestinal | Oesophagus | - | - |
| Upper gastrointestinal | Stomach | - | - |
| Lower gastrointestinal | Anal | - | - |
| Lower gastrointestinal | Colon | - | - |
| Lower gastrointestinal | Rectum | - | - |
| Lower gastrointestinal | Small intestine | - | - |
| Hepato-pancreato-biliary | Liver | - | - |
| Hepato-pancreato-biliary | Pancreas | - | - |
| Hepato-pancreato-biliary | Other Hepato-pancreato-biliary | - | - |
| Respiratory | Lung | - | - |
| Respiratory | Mesothelioma | - | - |
| Urological | Bladder | - | - |
| Urological | Kidney | - | - |
| Urological | Ureteric and other urinary | - | - |
| Haematological | Acute leukaemia | - | - |
| Haematological | Chronic lymphocytic leukaemia | - | - |
| Haematological | Hodgkin lymphoma | - | - |
| Haematological | Multiple myeloma | - | - |
| Haematological | Non-Hodgkin lymphoma | - | - |
| Haematological | Other haematological | - | - |
| Central nervous system | Central nervous system | - | - |
| Sarcoma | Bone sarcoma | - | - |
| Sarcoma | Connective and soft tissue sarcoma | - | - |
| Skin | Melanoma | - | - |
| Ocular | Ocular | - | - |
| Breast | Breast | - | - |
| Gynaecological | Cervix | - | - |
| Gynaecological | Ovary | - | - |
| Gynaecological | Uterus | - | - |
| Gynaecological | Vulva/Vagina | - | - |
| Prostate and other male organs | Penile | - | - |
| Prostate and other male organs | Prostate | - | - |
| Prostate and other male organs | Testicular | - | - |
| Other malignant neoplasms | Other malignant neoplasms | - | - |
| Unknown primary | Unknown primary | - | - |
| *Ulceration - Lip/oral cavity/tongue lump/mass (Men=198, Women=110)* | | | |
| Head and neck | Larynx | 1.01 (0.28, 3.61) | 0.00 (0.00, 3.37) |
| Head and neck | Oral cavity | 50.0 (43.1, 56.9) | 71.8 (62.8, 79.4) |
| Head and neck | Oropharynx | 21.2 (16.1, 27.4) | 7.27 (3.73, 13.7) |
| Head and neck | Thyroid | 0.00 (0.00, 1.90) | 0.91 (0.16, 4.97) |
| Head and neck | Other head and neck | 13.6 (9.54, 19.1) | 10.9 (6.35, 18.1) |
| Upper gastrointestinal | Oesophagus | 1.52 (0.52, 4.36) | 0.00 (0.00, 3.37) |
| Upper gastrointestinal | Stomach | 0.00 (0.00, 1.90) | 0.00 (0.00, 3.37) |
| Lower gastrointestinal | Anal | 0.00 (0.00, 1.90) | 0.00 (0.00, 3.37) |
| Lower gastrointestinal | Colon | 0.00 (0.00, 1.90) | 0.91 (0.16, 4.97) |
| Lower gastrointestinal | Rectum | 0.00 (0.00, 1.90) | 0.00 (0.00, 3.37) |
| Lower gastrointestinal | Small intestine | 0.00 (0.00, 1.90) | 0.00 (0.00, 3.37) |
| Hepato-pancreato-biliary | Liver | 0.00 (0.00, 1.90) | 0.00 (0.00, 3.37) |
| Hepato-pancreato-biliary | Pancreas | 0.00 (0.00, 1.90) | 0.00 (0.00, 3.37) |
| Hepato-pancreato-biliary | Other Hepato-pancreato-biliary | 0.00 (0.00, 1.90) | 0.00 (0.00, 3.37) |
| Respiratory | Lung | 2.02 (0.79, 5.08) | 0.00 (0.00, 3.37) |
| Respiratory | Mesothelioma | 0.00 (0.00, 1.90) | 0.00 (0.00, 3.37) |
| Urological | Bladder | 0.00 (0.00, 1.90) | 0.00 (0.00, 3.37) |
| Urological | Kidney | 0.00 (0.00, 1.90) | 0.00 (0.00, 3.37) |
| Urological | Ureteric and other urinary | 0.00 (0.00, 1.90) | 0.00 (0.00, 3.37) |
| Haematological | Acute leukaemia | 0.00 (0.00, 1.90) | 0.00 (0.00, 3.37) |
| Haematological | Chronic lymphocytic leukaemia | 0.51 (0.09, 2.80) | 0.00 (0.00, 3.37) |
| Haematological | Hodgkin lymphoma | 0.00 (0.00, 1.90) | 0.91 (0.16, 4.97) |
| Haematological | Multiple myeloma | 0.00 (0.00, 1.90) | 0.00 (0.00, 3.37) |
| Haematological | Non-Hodgkin lymphoma | 6.06 (3.50, 10.3) | 3.64 (1.42, 8.98) |
| Haematological | Other haematological | 0.51 (0.09, 2.80) | 0.00 (0.00, 3.37) |
| Central nervous system | Central nervous system | 0.00 (0.00, 1.90) | 0.00 (0.00, 3.37) |
| Sarcoma | Bone sarcoma | 1.01 (0.28, 3.61) | 0.00 (0.00, 3.37) |
| Sarcoma | Connective and soft tissue sarcoma | 0.51 (0.09, 2.80) | 1.82 (0.50, 6.39) |
| Skin | Melanoma | 0.51 (0.09, 2.80) | 0.00 (0.00, 3.37) |
| Ocular | Ocular | 0.00 (0.00, 1.90) | 0.00 (0.00, 3.37) |
| Breast | Breast | 0.00 (0.00, 1.90) | 0.00 (0.00, 3.37) |
| Gynaecological | Cervix | - | 0.00 (0.00, 3.37) |
| Gynaecological | Ovary | - | 0.00 (0.00, 3.37) |
| Gynaecological | Uterus | - | 0.00 (0.00, 3.37) |
| Gynaecological | Vulva/Vagina | - | 0.00 (0.00, 3.37) |
| Prostate and other male organs | Penile | 0.00 (0.00, 1.90) | - |
| Prostate and other male organs | Prostate | 1.01 (0.28, 3.61) | - |
| Prostate and other male organs | Testicular | 0.00 (0.00, 1.90) | - |
| Other malignant neoplasms | Other malignant neoplasms | 0.00 (0.00, 1.90) | 0.91 (0.16, 4.97) |
| Unknown primary | Unknown primary | 0.51 (0.09, 2.80) | 0.91 (0.16, 4.97) |
| *Ulceration - Lip/oral cavity/tongue ulcer (Men=104, Women=79)* | | | |
| Head and neck | Larynx | 0.96 (0.17, 5.25) | 0.00 (0.00, 4.64) |
| Head and neck | Oral cavity | 66.3 (56.8, 74.7) | 69.6 (58.8, 78.7) |
| Head and neck | Oropharynx | 10.6 (6.01, 18.0) | 8.86 (4.36, 17.2) |
| Head and neck | Thyroid | 0.00 (0.00, 3.56) | 0.00 (0.00, 4.64) |
| Head and neck | Other head and neck | 9.62 (5.31, 16.8) | 6.33 (2.73, 14.0) |
| Upper gastrointestinal | Oesophagus | 0.00 (0.00, 3.56) | 0.00 (0.00, 4.64) |
| Upper gastrointestinal | Stomach | 0.00 (0.00, 3.56) | 1.27 (0.22, 6.83) |
| Lower gastrointestinal | Anal | 0.00 (0.00, 3.56) | 0.00 (0.00, 4.64) |
| Lower gastrointestinal | Colon | 0.96 (0.17, 5.25) | 1.27 (0.22, 6.83) |
| Lower gastrointestinal | Rectum | 0.00 (0.00, 3.56) | 0.00 (0.00, 4.64) |
| Lower gastrointestinal | Small intestine | 0.00 (0.00, 3.56) | 0.00 (0.00, 4.64) |
| Hepato-pancreato-biliary | Liver | 0.00 (0.00, 3.56) | 0.00 (0.00, 4.64) |
| Hepato-pancreato-biliary | Pancreas | 0.00 (0.00, 3.56) | 0.00 (0.00, 4.64) |
| Hepato-pancreato-biliary | Other Hepato-pancreato-biliary | 0.00 (0.00, 3.56) | 0.00 (0.00, 4.64) |
| Respiratory | Lung | 1.92 (0.53, 6.74) | 2.53 (0.70, 8.77) |
| Respiratory | Mesothelioma | 0.96 (0.17, 5.25) | 0.00 (0.00, 4.64) |
| Urological | Bladder | 0.00 (0.00, 3.56) | 0.00 (0.00, 4.64) |
| Urological | Kidney | 0.00 (0.00, 3.56) | 0.00 (0.00, 4.64) |
| Urological | Ureteric and other urinary | 0.00 (0.00, 3.56) | 0.00 (0.00, 4.64) |
| Haematological | Acute leukaemia | 1.92 (0.53, 6.74) | 1.27 (0.22, 6.83) |
| Haematological | Chronic lymphocytic leukaemia | 0.00 (0.00, 3.56) | 0.00 (0.00, 4.64) |
| Haematological | Hodgkin lymphoma | 0.00 (0.00, 3.56) | 0.00 (0.00, 4.64) |
| Haematological | Multiple myeloma | 0.96 (0.17, 5.25) | 0.00 (0.00, 4.64) |
| Haematological | Non-Hodgkin lymphoma | 1.92 (0.53, 6.74) | 2.53 (0.70, 8.77) |
| Haematological | Other haematological | 0.00 (0.00, 3.56) | 2.53 (0.70, 8.77) |
| Central nervous system | Central nervous system | 0.00 (0.00, 3.56) | 0.00 (0.00, 4.64) |
| Sarcoma | Bone sarcoma | 0.00 (0.00, 3.56) | 0.00 (0.00, 4.64) |
| Sarcoma | Connective and soft tissue sarcoma | 0.00 (0.00, 3.56) | 0.00 (0.00, 4.64) |
| Skin | Melanoma | 0.96 (0.17, 5.25) | 2.53 (0.70, 8.77) |
| Ocular | Ocular | 0.00 (0.00, 3.56) | 0.00 (0.00, 4.64) |
| Breast | Breast | 0.00 (0.00, 3.56) | 0.00 (0.00, 4.64) |
| Gynaecological | Cervix | - | 0.00 (0.00, 4.64) |
| Gynaecological | Ovary | - | 1.27 (0.22, 6.83) |
| Gynaecological | Uterus | - | 0.00 (0.00, 4.64) |
| Gynaecological | Vulva/Vagina | - | 0.00 (0.00, 4.64) |
| Prostate and other male organs | Penile | 0.96 (0.17, 5.25) | - |
| Prostate and other male organs | Prostate | 0.00 (0.00, 3.56) | - |
| Prostate and other male organs | Testicular | 0.00 (0.00, 3.56) | - |
| Other malignant neoplasms | Other malignant neoplasms | 0.96 (0.17, 5.25) | 0.00 (0.00, 4.64) |
| Unknown primary | Unknown primary | 0.96 (0.17, 5.25) | 0.00 (0.00, 4.64) |
| *Ulceration - Ulceration (Men=105, Women=68)* | | | |
| Head and neck | Larynx | 0.00 (0.00, 3.53) | 0.00 (0.00, 5.35) |
| Head and neck | Oral cavity | 1.90 (0.52, 6.68) | 5.88 (2.31, 14.2) |
| Head and neck | Oropharynx | 0.95 (0.17, 5.20) | 0.00 (0.00, 5.35) |
| Head and neck | Thyroid | 0.00 (0.00, 3.53) | 0.00 (0.00, 5.35) |
| Head and neck | Other head and neck | 6.67 (3.27, 13.1) | 7.35 (3.18, 16.1) |
| Upper gastrointestinal | Oesophagus | 0.00 (0.00, 3.53) | 0.00 (0.00, 5.35) |
| Upper gastrointestinal | Stomach | 0.00 (0.00, 3.53) | 0.00 (0.00, 5.35) |
| Lower gastrointestinal | Anal | 2.86 (0.98, 8.07) | 1.47 (0.26, 7.87) |
| Lower gastrointestinal | Colon | 0.95 (0.17, 5.20) | 0.00 (0.00, 5.35) |
| Lower gastrointestinal | Rectum | 0.00 (0.00, 3.53) | 0.00 (0.00, 5.35) |
| Lower gastrointestinal | Small intestine | 0.00 (0.00, 3.53) | 0.00 (0.00, 5.35) |
| Hepato-pancreato-biliary | Liver | 0.00 (0.00, 3.53) | 0.00 (0.00, 5.35) |
| Hepato-pancreato-biliary | Pancreas | 0.95 (0.17, 5.20) | 0.00 (0.00, 5.35) |
| Hepato-pancreato-biliary | Other Hepato-pancreato-biliary | 0.00 (0.00, 3.53) | 0.00 (0.00, 5.35) |
| Respiratory | Lung | 1.90 (0.52, 6.68) | 0.00 (0.00, 5.35) |
| Respiratory | Mesothelioma | 0.00 (0.00, 3.53) | 0.00 (0.00, 5.35) |
| Urological | Bladder | 0.95 (0.17, 5.20) | 0.00 (0.00, 5.35) |
| Urological | Kidney | 0.00 (0.00, 3.53) | 0.00 (0.00, 5.35) |
| Urological | Ureteric and other urinary | 0.95 (0.17, 5.20) | 0.00 (0.00, 5.35) |
| Haematological | Acute leukaemia | 2.86 (0.98, 8.07) | 2.94 (0.81, 10.1) |
| Haematological | Chronic lymphocytic leukaemia | 0.95 (0.17, 5.20) | 0.00 (0.00, 5.35) |
| Haematological | Hodgkin lymphoma | 0.00 (0.00, 3.53) | 0.00 (0.00, 5.35) |
| Haematological | Multiple myeloma | 0.95 (0.17, 5.20) | 0.00 (0.00, 5.35) |
| Haematological | Non-Hodgkin lymphoma | 2.86 (0.98, 8.07) | 4.41 (1.51, 12.2) |
| Haematological | Other haematological | 3.81 (1.49, 9.39) | 1.47 (0.26, 7.87) |
| Central nervous system | Central nervous system | 0.00 (0.00, 3.53) | 0.00 (0.00, 5.35) |
| Sarcoma | Bone sarcoma | 0.00 (0.00, 3.53) | 0.00 (0.00, 5.35) |
| Sarcoma | Connective and soft tissue sarcoma | 9.52 (5.26, 16.6) | 1.47 (0.26, 7.87) |
| Skin | Melanoma | 50.5 (41.1, 59.9) | 47.1 (35.7, 58.8) |
| Ocular | Ocular | 0.00 (0.00, 3.53) | 0.00 (0.00, 5.35) |
| Breast | Breast | 0.00 (0.00, 3.53) | 20.6 (12.7, 31.6) |
| Gynaecological | Cervix | - | 0.00 (0.00, 5.35) |
| Gynaecological | Ovary | - | 1.47 (0.26, 7.87) |
| Gynaecological | Uterus | - | 0.00 (0.00, 5.35) |
| Gynaecological | Vulva/Vagina | - | 0.00 (0.00, 5.35) |
| Prostate and other male organs | Penile | 3.81 (1.49, 9.39) | - |
| Prostate and other male organs | Prostate | 0.95 (0.17, 5.20) | - |
| Prostate and other male organs | Testicular | 1.90 (0.52, 6.68) | - |
| Other malignant neoplasms | Other malignant neoplasms | 0.00 (0.00, 3.53) | 0.00 (0.00, 5.35) |
| Unknown primary | Unknown primary | 3.81 (1.49, 9.39) | 5.88 (2.31, 14.2) |
| *Upper abdominal - Dyspepsia (Men=530, Women=378)* | | | |
| Head and neck | Larynx | 0.19 (0.03, 1.06) | 0.26 (0.05, 1.48) |
| Head and neck | Oral cavity | 0.00 (0.00, 0.72) | 0.00 (0.00, 1.01) |
| Head and neck | Oropharynx | 0.00 (0.00, 0.72) | 0.00 (0.00, 1.01) |
| Head and neck | Thyroid | 0.00 (0.00, 0.72) | 0.00 (0.00, 1.01) |
| Head and neck | Other head and neck | 0.38 (0.10, 1.37) | 0.00 (0.00, 1.01) |
| Upper gastrointestinal | Oesophagus | 34.9 (31.0, 39.1) | 19.0 (15.4, 23.3) |
| Upper gastrointestinal | Stomach | 21.7 (18.4, 25.4) | 16.4 (13.0, 20.5) |
| Lower gastrointestinal | Anal | 0.00 (0.00, 0.72) | 0.00 (0.00, 1.01) |
| Lower gastrointestinal | Colon | 7.55 (5.59, 10.1) | 10.6 (7.87, 14.1) |
| Lower gastrointestinal | Rectum | 0.75 (0.29, 1.92) | 1.59 (0.73, 3.42) |
| Lower gastrointestinal | Small intestine | 2.08 (1.16, 3.68) | 2.38 (1.26, 4.46) |
| Hepato-pancreato-biliary | Liver | 3.02 (1.87, 4.85) | 3.17 (1.83, 5.47) |
| Hepato-pancreato-biliary | Pancreas | 10.2 (7.89, 13.1) | 13.2 (10.2, 17.0) |
| Hepato-pancreato-biliary | Other Hepato-pancreato-biliary | 2.08 (1.16, 3.68) | 3.44 (2.02, 5.79) |
| Respiratory | Lung | 4.34 (2.91, 6.43) | 6.88 (4.74, 9.89) |
| Respiratory | Mesothelioma | 0.38 (0.10, 1.37) | 0.00 (0.00, 1.01) |
| Urological | Bladder | 0.57 (0.19, 1.65) | 0.53 (0.15, 1.91) |
| Urological | Kidney | 1.70 (0.90, 3.20) | 2.38 (1.26, 4.46) |
| Urological | Ureteric and other urinary | 0.00 (0.00, 0.72) | 0.26 (0.05, 1.48) |
| Haematological | Acute leukaemia | 0.19 (0.03, 1.06) | 0.00 (0.00, 1.01) |
| Haematological | Chronic lymphocytic leukaemia | 0.19 (0.03, 1.06) | 0.26 (0.05, 1.48) |
| Haematological | Hodgkin lymphoma | 0.38 (0.10, 1.37) | 0.00 (0.00, 1.01) |
| Haematological | Multiple myeloma | 0.38 (0.10, 1.37) | 0.00 (0.00, 1.01) |
| Haematological | Non-Hodgkin lymphoma | 3.21 (2.01, 5.08) | 3.97 (2.42, 6.44) |
| Haematological | Other haematological | 0.19 (0.03, 1.06) | 0.53 (0.15, 1.91) |
| Central nervous system | Central nervous system | 0.00 (0.00, 0.72) | 0.00 (0.00, 1.01) |
| Sarcoma | Bone sarcoma | 0.00 (0.00, 0.72) | 0.00 (0.00, 1.01) |
| Sarcoma | Connective and soft tissue sarcoma | 0.19 (0.03, 1.06) | 1.59 (0.73, 3.42) |
| Skin | Melanoma | 0.38 (0.10, 1.37) | 0.00 (0.00, 1.01) |
| Ocular | Ocular | 0.00 (0.00, 0.72) | 0.00 (0.00, 1.01) |
| Breast | Breast | 0.00 (0.00, 0.72) | 2.12 (1.08, 4.12) |
| Gynaecological | Cervix | - | 0.26 (0.05, 1.48) |
| Gynaecological | Ovary | - | 7.67 (5.39, 10.8) |
| Gynaecological | Uterus | - | 0.26 (0.05, 1.48) |
| Gynaecological | Vulva/Vagina | - | 0.00 (0.00, 1.01) |
| Prostate and other male organs | Penile | 0.00 (0.00, 0.72) | - |
| Prostate and other male organs | Prostate | 3.02 (1.87, 4.85) | - |
| Prostate and other male organs | Testicular | 0.00 (0.00, 0.72) | - |
| Other malignant neoplasms | Other malignant neoplasms | 0.38 (0.10, 1.37) | 0.26 (0.05, 1.48) |
| Unknown primary | Unknown primary | 1.70 (0.90, 3.20) | 2.91 (1.63, 5.14) |
| *Upper abdominal - Dysphagia (Men=761, Women=377)* | | | |
| Head and neck | Larynx | 2.10 (1.30, 3.39) | 0.53 (0.15, 1.91) |
| Head and neck | Oral cavity | 0.66 (0.28, 1.53) | 1.33 (0.57, 3.07) |
| Head and neck | Oropharynx | 3.68 (2.56, 5.27) | 1.86 (0.90, 3.78) |
| Head and neck | Thyroid | 0.66 (0.28, 1.53) | 3.18 (1.83, 5.48) |
| Head and neck | Other head and neck | 3.68 (2.56, 5.27) | 2.39 (1.26, 4.47) |
| Upper gastrointestinal | Oesophagus | 57.7 (54.1, 61.2) | 53.3 (48.3, 58.3) |
| Upper gastrointestinal | Stomach | 14.3 (12.0, 17.0) | 12.5 (9.51, 16.2) |
| Lower gastrointestinal | Anal | 0.00 (0.00, 0.50) | 0.00 (0.00, 1.01) |
| Lower gastrointestinal | Colon | 2.50 (1.60, 3.87) | 3.71 (2.22, 6.14) |
| Lower gastrointestinal | Rectum | 0.26 (0.07, 0.95) | 0.27 (0.05, 1.49) |
| Lower gastrointestinal | Small intestine | 0.13 (0.02, 0.74) | 0.80 (0.27, 2.31) |
| Hepato-pancreato-biliary | Liver | 0.79 (0.36, 1.71) | 0.80 (0.27, 2.31) |
| Hepato-pancreato-biliary | Pancreas | 2.76 (1.81, 4.18) | 2.92 (1.64, 5.15) |
| Hepato-pancreato-biliary | Other Hepato-pancreato-biliary | 0.13 (0.02, 0.74) | 0.53 (0.15, 1.91) |
| Respiratory | Lung | 5.65 (4.22, 7.52) | 8.22 (5.85, 11.4) |
| Respiratory | Mesothelioma | 0.00 (0.00, 0.50) | 0.00 (0.00, 1.01) |
| Urological | Bladder | 0.00 (0.00, 0.50) | 0.00 (0.00, 1.01) |
| Urological | Kidney | 0.79 (0.36, 1.71) | 0.80 (0.27, 2.31) |
| Urological | Ureteric and other urinary | 0.00 (0.00, 0.50) | 0.00 (0.00, 1.01) |
| Haematological | Acute leukaemia | 0.00 (0.00, 0.50) | 0.53 (0.15, 1.91) |
| Haematological | Chronic lymphocytic leukaemia | 0.13 (0.02, 0.74) | 0.27 (0.05, 1.49) |
| Haematological | Hodgkin lymphoma | 0.13 (0.02, 0.74) | 0.27 (0.05, 1.49) |
| Haematological | Multiple myeloma | 0.26 (0.07, 0.95) | 0.00 (0.00, 1.01) |
| Haematological | Non-Hodgkin lymphoma | 1.71 (1.00, 2.90) | 2.39 (1.26, 4.47) |
| Haematological | Other haematological | 0.00 (0.00, 0.50) | 0.27 (0.05, 1.49) |
| Central nervous system | Central nervous system | 0.00 (0.00, 0.50) | 0.00 (0.00, 1.01) |
| Sarcoma | Bone sarcoma | 0.00 (0.00, 0.50) | 0.00 (0.00, 1.01) |
| Sarcoma | Connective and soft tissue sarcoma | 0.13 (0.02, 0.74) | 0.00 (0.00, 1.01) |
| Skin | Melanoma | 0.13 (0.02, 0.74) | 0.00 (0.00, 1.01) |
| Ocular | Ocular | 0.00 (0.00, 0.50) | 0.00 (0.00, 1.01) |
| Breast | Breast | 0.00 (0.00, 0.50) | 0.80 (0.27, 2.31) |
| Gynaecological | Cervix | - | 0.00 (0.00, 1.01) |
| Gynaecological | Ovary | - | 0.00 (0.00, 1.01) |
| Gynaecological | Uterus | - | 0.27 (0.05, 1.49) |
| Gynaecological | Vulva/Vagina | - | 0.00 (0.00, 1.01) |
| Prostate and other male organs | Penile | 0.00 (0.00, 0.50) | - |
| Prostate and other male organs | Prostate | 0.53 (0.20, 1.34) | - |
| Prostate and other male organs | Testicular | 0.00 (0.00, 0.50) | - |
| Other malignant neoplasms | Other malignant neoplasms | 0.00 (0.00, 0.50) | 0.27 (0.05, 1.49) |
| Unknown primary | Unknown primary | 1.18 (0.62, 2.23) | 1.86 (0.90, 3.78) |
| *Upper abdominal - Early satiety (Men=85, Women=60)* | | | |
| Head and neck | Larynx | 0.00 (0.00, 4.32) | 0.00 (0.00, 6.02) |
| Head and neck | Oral cavity | 0.00 (0.00, 4.32) | 0.00 (0.00, 6.02) |
| Head and neck | Oropharynx | 0.00 (0.00, 4.32) | 1.67 (0.29, 8.86) |
| Head and neck | Thyroid | 0.00 (0.00, 4.32) | 0.00 (0.00, 6.02) |
| Head and neck | Other head and neck | 0.00 (0.00, 4.32) | 0.00 (0.00, 6.02) |
| Upper gastrointestinal | Oesophagus | 15.3 (9.16, 24.4) | 15.0 (8.10, 26.1) |
| Upper gastrointestinal | Stomach | 21.2 (13.8, 31.0) | 13.3 (6.91, 24.2) |
| Lower gastrointestinal | Anal | 0.00 (0.00, 4.32) | 0.00 (0.00, 6.02) |
| Lower gastrointestinal | Colon | 5.88 (2.54, 13.0) | 16.7 (9.31, 28.0) |
| Lower gastrointestinal | Rectum | 0.00 (0.00, 4.32) | 0.00 (0.00, 6.02) |
| Lower gastrointestinal | Small intestine | 0.00 (0.00, 4.32) | 3.33 (0.92, 11.4) |
| Hepato-pancreato-biliary | Liver | 2.35 (0.65, 8.18) | 6.67 (2.62, 15.9) |
| Hepato-pancreato-biliary | Pancreas | 16.5 (10.1, 25.8) | 15.0 (8.10, 26.1) |
| Hepato-pancreato-biliary | Other Hepato-pancreato-biliary | 0.00 (0.00, 4.32) | 0.00 (0.00, 6.02) |
| Respiratory | Lung | 14.1 (8.26, 23.1) | 5.00 (1.71, 13.7) |
| Respiratory | Mesothelioma | 0.00 (0.00, 4.32) | 1.67 (0.29, 8.86) |
| Urological | Bladder | 0.00 (0.00, 4.32) | 0.00 (0.00, 6.02) |
| Urological | Kidney | 1.18 (0.21, 6.37) | 1.67 (0.29, 8.86) |
| Urological | Ureteric and other urinary | 0.00 (0.00, 4.32) | 0.00 (0.00, 6.02) |
| Haematological | Acute leukaemia | 0.00 (0.00, 4.32) | 0.00 (0.00, 6.02) |
| Haematological | Chronic lymphocytic leukaemia | 1.18 (0.21, 6.37) | 0.00 (0.00, 6.02) |
| Haematological | Hodgkin lymphoma | 2.35 (0.65, 8.18) | 0.00 (0.00, 6.02) |
| Haematological | Multiple myeloma | 0.00 (0.00, 4.32) | 0.00 (0.00, 6.02) |
| Haematological | Non-Hodgkin lymphoma | 9.41 (4.85, 17.5) | 0.00 (0.00, 6.02) |
| Haematological | Other haematological | 1.18 (0.21, 6.37) | 0.00 (0.00, 6.02) |
| Central nervous system | Central nervous system | 0.00 (0.00, 4.32) | 0.00 (0.00, 6.02) |
| Sarcoma | Bone sarcoma | 0.00 (0.00, 4.32) | 1.67 (0.29, 8.86) |
| Sarcoma | Connective and soft tissue sarcoma | 1.18 (0.21, 6.37) | 0.00 (0.00, 6.02) |
| Skin | Melanoma | 0.00 (0.00, 4.32) | 0.00 (0.00, 6.02) |
| Ocular | Ocular | 0.00 (0.00, 4.32) | 0.00 (0.00, 6.02) |
| Breast | Breast | 0.00 (0.00, 4.32) | 5.00 (1.71, 13.7) |
| Gynaecological | Cervix | - | 0.00 (0.00, 6.02) |
| Gynaecological | Ovary | - | 10.0 (4.66, 20.1) |
| Gynaecological | Uterus | - | 1.67 (0.29, 8.86) |
| Gynaecological | Vulva/Vagina | - | 0.00 (0.00, 6.02) |
| Prostate and other male organs | Penile | 0.00 (0.00, 4.32) | - |
| Prostate and other male organs | Prostate | 3.53 (1.21, 9.87) | - |
| Prostate and other male organs | Testicular | 0.00 (0.00, 4.32) | - |
| Other malignant neoplasms | Other malignant neoplasms | 1.18 (0.21, 6.37) | 0.00 (0.00, 6.02) |
| Unknown primary | Unknown primary | 3.53 (1.21, 9.87) | 1.67 (0.29, 8.86) |
| *Upper abdominal - Gastroesophageal reflux (Men=199, Women=145)* | | | |
| Head and neck | Larynx | 0.50 (0.09, 2.79) | 0.00 (0.00, 2.58) |
| Head and neck | Oral cavity | 0.50 (0.09, 2.79) | 0.00 (0.00, 2.58) |
| Head and neck | Oropharynx | 1.01 (0.28, 3.59) | 0.69 (0.12, 3.80) |
| Head and neck | Thyroid | 0.00 (0.00, 1.89) | 0.00 (0.00, 2.58) |
| Head and neck | Other head and neck | 0.50 (0.09, 2.79) | 0.00 (0.00, 2.58) |
| Upper gastrointestinal | Oesophagus | 35.2 (28.9, 42.0) | 29.7 (22.8, 37.5) |
| Upper gastrointestinal | Stomach | 22.1 (16.9, 28.4) | 9.66 (5.84, 15.6) |
| Lower gastrointestinal | Anal | 0.00 (0.00, 1.89) | 0.00 (0.00, 2.58) |
| Lower gastrointestinal | Colon | 5.53 (3.11, 9.63) | 11.7 (7.45, 18.0) |
| Lower gastrointestinal | Rectum | 1.51 (0.51, 4.34) | 1.38 (0.38, 4.89) |
| Lower gastrointestinal | Small intestine | 2.51 (1.08, 5.75) | 4.83 (2.36, 9.63) |
| Hepato-pancreato-biliary | Liver | 2.01 (0.78, 5.05) | 4.83 (2.36, 9.63) |
| Hepato-pancreato-biliary | Pancreas | 6.03 (3.48, 10.2) | 9.66 (5.84, 15.6) |
| Hepato-pancreato-biliary | Other Hepato-pancreato-biliary | 0.50 (0.09, 2.79) | 0.00 (0.00, 2.58) |
| Respiratory | Lung | 7.04 (4.24, 11.5) | 8.97 (5.31, 14.7) |
| Respiratory | Mesothelioma | 0.50 (0.09, 2.79) | 0.00 (0.00, 2.58) |
| Urological | Bladder | 0.50 (0.09, 2.79) | 0.69 (0.12, 3.80) |
| Urological | Kidney | 1.01 (0.28, 3.59) | 2.07 (0.71, 5.91) |
| Urological | Ureteric and other urinary | 0.00 (0.00, 1.89) | 0.00 (0.00, 2.58) |
| Haematological | Acute leukaemia | 1.01 (0.28, 3.59) | 0.00 (0.00, 2.58) |
| Haematological | Chronic lymphocytic leukaemia | 0.50 (0.09, 2.79) | 0.00 (0.00, 2.58) |
| Haematological | Hodgkin lymphoma | 0.50 (0.09, 2.79) | 0.69 (0.12, 3.80) |
| Haematological | Multiple myeloma | 0.50 (0.09, 2.79) | 0.00 (0.00, 2.58) |
| Haematological | Non-Hodgkin lymphoma | 3.02 (1.39, 6.42) | 2.76 (1.08, 6.88) |
| Haematological | Other haematological | 1.01 (0.28, 3.59) | 0.00 (0.00, 2.58) |
| Central nervous system | Central nervous system | 0.00 (0.00, 1.89) | 0.00 (0.00, 2.58) |
| Sarcoma | Bone sarcoma | 0.00 (0.00, 1.89) | 0.00 (0.00, 2.58) |
| Sarcoma | Connective and soft tissue sarcoma | 0.00 (0.00, 1.89) | 0.69 (0.12, 3.80) |
| Skin | Melanoma | 0.50 (0.09, 2.79) | 0.00 (0.00, 2.58) |
| Ocular | Ocular | 0.00 (0.00, 1.89) | 0.00 (0.00, 2.58) |
| Breast | Breast | 0.50 (0.09, 2.79) | 1.38 (0.38, 4.89) |
| Gynaecological | Cervix | - | 0.00 (0.00, 2.58) |
| Gynaecological | Ovary | - | 4.83 (2.36, 9.63) |
| Gynaecological | Uterus | - | 0.69 (0.12, 3.80) |
| Gynaecological | Vulva/Vagina | - | 0.00 (0.00, 2.58) |
| Prostate and other male organs | Penile | 0.00 (0.00, 1.89) | - |
| Prostate and other male organs | Prostate | 3.02 (1.39, 6.42) | - |
| Prostate and other male organs | Testicular | 0.00 (0.00, 1.89) | - |
| Other malignant neoplasms | Other malignant neoplasms | 0.00 (0.00, 1.89) | 0.00 (0.00, 2.58) |
| Unknown primary | Unknown primary | 2.51 (1.08, 5.75) | 4.83 (2.36, 9.63) |
| *Upper abdominal - Haematemesis (Men=68, Women=61)* | | | |
| Head and neck | Larynx | 0.00 (0.00, 5.35) | 0.00 (0.00, 5.92) |
| Head and neck | Oral cavity | 0.00 (0.00, 5.35) | 0.00 (0.00, 5.92) |
| Head and neck | Oropharynx | 0.00 (0.00, 5.35) | 1.64 (0.29, 8.72) |
| Head and neck | Thyroid | 0.00 (0.00, 5.35) | 0.00 (0.00, 5.92) |
| Head and neck | Other head and neck | 1.47 (0.26, 7.87) | 0.00 (0.00, 5.92) |
| Upper gastrointestinal | Oesophagus | 19.1 (11.5, 30.0) | 6.56 (2.58, 15.7) |
| Upper gastrointestinal | Stomach | 25.0 (16.2, 36.4) | 19.7 (11.6, 31.3) |
| Lower gastrointestinal | Anal | 0.00 (0.00, 5.35) | 0.00 (0.00, 5.92) |
| Lower gastrointestinal | Colon | 11.8 (6.08, 21.5) | 11.5 (5.67, 21.8) |
| Lower gastrointestinal | Rectum | 0.00 (0.00, 5.35) | 3.28 (0.90, 11.2) |
| Lower gastrointestinal | Small intestine | 2.94 (0.81, 10.1) | 4.92 (1.69, 13.5) |
| Hepato-pancreato-biliary | Liver | 7.35 (3.18, 16.1) | 4.92 (1.69, 13.5) |
| Hepato-pancreato-biliary | Pancreas | 4.41 (1.51, 12.2) | 11.5 (5.67, 21.8) |
| Hepato-pancreato-biliary | Other Hepato-pancreato-biliary | 4.41 (1.51, 12.2) | 3.28 (0.90, 11.2) |
| Respiratory | Lung | 2.94 (0.81, 10.1) | 11.5 (5.67, 21.8) |
| Respiratory | Mesothelioma | 0.00 (0.00, 5.35) | 0.00 (0.00, 5.92) |
| Urological | Bladder | 1.47 (0.26, 7.87) | 3.28 (0.90, 11.2) |
| Urological | Kidney | 1.47 (0.26, 7.87) | 0.00 (0.00, 5.92) |
| Urological | Ureteric and other urinary | 1.47 (0.26, 7.87) | 1.64 (0.29, 8.72) |
| Haematological | Acute leukaemia | 0.00 (0.00, 5.35) | 1.64 (0.29, 8.72) |
| Haematological | Chronic lymphocytic leukaemia | 0.00 (0.00, 5.35) | 0.00 (0.00, 5.92) |
| Haematological | Hodgkin lymphoma | 0.00 (0.00, 5.35) | 0.00 (0.00, 5.92) |
| Haematological | Multiple myeloma | 2.94 (0.81, 10.1) | 0.00 (0.00, 5.92) |
| Haematological | Non-Hodgkin lymphoma | 5.88 (2.31, 14.2) | 3.28 (0.90, 11.2) |
| Haematological | Other haematological | 0.00 (0.00, 5.35) | 1.64 (0.29, 8.72) |
| Central nervous system | Central nervous system | 0.00 (0.00, 5.35) | 0.00 (0.00, 5.92) |
| Sarcoma | Bone sarcoma | 0.00 (0.00, 5.35) | 0.00 (0.00, 5.92) |
| Sarcoma | Connective and soft tissue sarcoma | 0.00 (0.00, 5.35) | 0.00 (0.00, 5.92) |
| Skin | Melanoma | 1.47 (0.26, 7.87) | 0.00 (0.00, 5.92) |
| Ocular | Ocular | 0.00 (0.00, 5.35) | 0.00 (0.00, 5.92) |
| Breast | Breast | 0.00 (0.00, 5.35) | 0.00 (0.00, 5.92) |
| Gynaecological | Cervix | - | 0.00 (0.00, 5.92) |
| Gynaecological | Ovary | - | 1.64 (0.29, 8.72) |
| Gynaecological | Uterus | - | 0.00 (0.00, 5.92) |
| Gynaecological | Vulva/Vagina | - | 0.00 (0.00, 5.92) |
| Prostate and other male organs | Penile | 0.00 (0.00, 5.35) | - |
| Prostate and other male organs | Prostate | 1.47 (0.26, 7.87) | - |
| Prostate and other male organs | Testicular | 0.00 (0.00, 5.35) | - |
| Other malignant neoplasms | Other malignant neoplasms | 0.00 (0.00, 5.35) | 0.00 (0.00, 5.92) |
| Unknown primary | Unknown primary | 4.41 (1.51, 12.2) | 8.20 (3.55, 17.8) |
| *Upper abdominal - Jaundice (Men=287, Women=231)* | | | |
| Head and neck | Larynx | 0.00 (0.00, 1.32) | 0.00 (0.00, 1.64) |
| Head and neck | Oral cavity | 0.00 (0.00, 1.32) | 0.00 (0.00, 1.64) |
| Head and neck | Oropharynx | 0.00 (0.00, 1.32) | 0.00 (0.00, 1.64) |
| Head and neck | Thyroid | 0.00 (0.00, 1.32) | 0.00 (0.00, 1.64) |
| Head and neck | Other head and neck | 0.00 (0.00, 1.32) | 0.00 (0.00, 1.64) |
| Upper gastrointestinal | Oesophagus | 0.70 (0.19, 2.50) | 0.43 (0.08, 2.41) |
| Upper gastrointestinal | Stomach | 0.35 (0.06, 1.95) | 0.87 (0.24, 3.10) |
| Lower gastrointestinal | Anal | 0.00 (0.00, 1.32) | 0.00 (0.00, 1.64) |
| Lower gastrointestinal | Colon | 3.48 (1.90, 6.29) | 0.00 (0.00, 1.64) |
| Lower gastrointestinal | Rectum | 0.35 (0.06, 1.95) | 0.00 (0.00, 1.64) |
| Lower gastrointestinal | Small intestine | 1.74 (0.75, 4.01) | 1.73 (0.68, 4.37) |
| Hepato-pancreato-biliary | Liver | 20.2 (16.0, 25.2) | 17.7 (13.4, 23.2) |
| Hepato-pancreato-biliary | Pancreas | 47.4 (41.7, 53.2) | 44.6 (38.3, 51.0) |
| Hepato-pancreato-biliary | Other Hepato-pancreato-biliary | 15.7 (11.9, 20.3) | 23.4 (18.4, 29.2) |
| Respiratory | Lung | 3.14 (1.66, 5.85) | 1.30 (0.44, 3.75) |
| Respiratory | Mesothelioma | 0.35 (0.06, 1.95) | 0.00 (0.00, 1.64) |
| Urological | Bladder | 1.39 (0.54, 3.53) | 0.43 (0.08, 2.41) |
| Urological | Kidney | 0.70 (0.19, 2.50) | 0.43 (0.08, 2.41) |
| Urological | Ureteric and other urinary | 0.00 (0.00, 1.32) | 0.00 (0.00, 1.64) |
| Haematological | Acute leukaemia | 0.35 (0.06, 1.95) | 0.43 (0.08, 2.41) |
| Haematological | Chronic lymphocytic leukaemia | 0.00 (0.00, 1.32) | 0.43 (0.08, 2.41) |
| Haematological | Hodgkin lymphoma | 0.00 (0.00, 1.32) | 0.00 (0.00, 1.64) |
| Haematological | Multiple myeloma | 0.00 (0.00, 1.32) | 0.00 (0.00, 1.64) |
| Haematological | Non-Hodgkin lymphoma | 1.05 (0.36, 3.03) | 2.60 (1.20, 5.55) |
| Haematological | Other haematological | 0.00 (0.00, 1.32) | 0.00 (0.00, 1.64) |
| Central nervous system | Central nervous system | 0.00 (0.00, 1.32) | 0.00 (0.00, 1.64) |
| Sarcoma | Bone sarcoma | 0.00 (0.00, 1.32) | 0.00 (0.00, 1.64) |
| Sarcoma | Connective and soft tissue sarcoma | 0.00 (0.00, 1.32) | 0.00 (0.00, 1.64) |
| Skin | Melanoma | 0.00 (0.00, 1.32) | 0.43 (0.08, 2.41) |
| Ocular | Ocular | 0.00 (0.00, 1.32) | 0.00 (0.00, 1.64) |
| Breast | Breast | 0.00 (0.00, 1.32) | 0.43 (0.08, 2.41) |
| Gynaecological | Cervix | - | 0.00 (0.00, 1.64) |
| Gynaecological | Ovary | - | 0.87 (0.24, 3.10) |
| Gynaecological | Uterus | - | 0.43 (0.08, 2.41) |
| Gynaecological | Vulva/Vagina | - | 0.00 (0.00, 1.64) |
| Prostate and other male organs | Penile | 0.00 (0.00, 1.32) | - |
| Prostate and other male organs | Prostate | 1.39 (0.54, 3.53) | - |
| Prostate and other male organs | Testicular | 0.00 (0.00, 1.32) | - |
| Other malignant neoplasms | Other malignant neoplasms | 0.00 (0.00, 1.32) | 0.00 (0.00, 1.64) |
| Unknown primary | Unknown primary | 1.74 (0.75, 4.01) | 3.46 (1.77, 6.68) |
| *Upper abdominal - Loss of appetite (Men=869, Women=795)* | | | |
| Head and neck | Larynx | 0.81 (0.39, 1.65) | 0.13 (0.02, 0.71) |
| Head and neck | Oral cavity | 0.23 (0.06, 0.84) | 0.00 (0.00, 0.48) |
| Head and neck | Oropharynx | 0.69 (0.32, 1.50) | 0.25 (0.07, 0.91) |
| Head and neck | Thyroid | 0.12 (0.02, 0.65) | 0.13 (0.02, 0.71) |
| Head and neck | Other head and neck | 0.46 (0.18, 1.18) | 0.00 (0.00, 0.48) |
| Upper gastrointestinal | Oesophagus | 5.52 (4.19, 7.25) | 3.14 (2.14, 4.60) |
| Upper gastrointestinal | Stomach | 5.64 (4.29, 7.38) | 5.66 (4.26, 7.49) |
| Lower gastrointestinal | Anal | 0.12 (0.02, 0.65) | 0.25 (0.07, 0.91) |
| Lower gastrointestinal | Colon | 11.9 (9.87, 14.2) | 14.3 (12.1, 16.9) |
| Lower gastrointestinal | Rectum | 4.26 (3.10, 5.81) | 2.52 (1.63, 3.85) |
| Lower gastrointestinal | Small intestine | 0.46 (0.18, 1.18) | 1.38 (0.77, 2.46) |
| Hepato-pancreato-biliary | Liver | 5.18 (3.89, 6.86) | 3.65 (2.55, 5.19) |
| Hepato-pancreato-biliary | Pancreas | 11.4 (9.45, 13.7) | 13.1 (10.9, 15.6) |
| Hepato-pancreato-biliary | Other Hepato-pancreato-biliary | 1.38 (0.79, 2.40) | 3.02 (2.04, 4.45) |
| Respiratory | Lung | 20.0 (17.5, 22.8) | 18.9 (16.3, 21.7) |
| Respiratory | Mesothelioma | 1.73 (1.05, 2.83) | 0.13 (0.02, 0.71) |
| Urological | Bladder | 1.04 (0.55, 1.96) | 0.63 (0.27, 1.46) |
| Urological | Kidney | 1.73 (1.05, 2.83) | 1.76 (1.05, 2.93) |
| Urological | Ureteric and other urinary | 0.23 (0.06, 0.84) | 0.25 (0.07, 0.91) |
| Haematological | Acute leukaemia | 1.61 (0.96, 2.69) | 1.38 (0.77, 2.46) |
| Haematological | Chronic lymphocytic leukaemia | 0.23 (0.06, 0.84) | 0.13 (0.02, 0.71) |
| Haematological | Hodgkin lymphoma | 0.69 (0.32, 1.50) | 0.75 (0.35, 1.64) |
| Haematological | Multiple myeloma | 2.19 (1.40, 3.39) | 2.14 (1.34, 3.40) |
| Haematological | Non-Hodgkin lymphoma | 5.41 (4.09, 7.12) | 3.90 (2.76, 5.48) |
| Haematological | Other haematological | 1.15 (0.63, 2.11) | 1.26 (0.68, 2.30) |
| Central nervous system | Central nervous system | 0.35 (0.12, 1.01) | 0.38 (0.13, 1.10) |
| Sarcoma | Bone sarcoma | 0.00 (0.00, 0.44) | 0.00 (0.00, 0.48) |
| Sarcoma | Connective and soft tissue sarcoma | 0.12 (0.02, 0.65) | 0.75 (0.35, 1.64) |
| Skin | Melanoma | 0.23 (0.06, 0.84) | 0.13 (0.02, 0.71) |
| Ocular | Ocular | 0.00 (0.00, 0.44) | 0.00 (0.00, 0.48) |
| Breast | Breast | 0.00 (0.00, 0.44) | 2.26 (1.44, 3.55) |
| Gynaecological | Cervix | - | 0.63 (0.27, 1.46) |
| Gynaecological | Ovary | - | 8.18 (6.47, 10.3) |
| Gynaecological | Uterus | - | 1.51 (0.87, 2.62) |
| Gynaecological | Vulva/Vagina | - | 0.25 (0.07, 0.91) |
| Prostate and other male organs | Penile | 0.00 (0.00, 0.44) | - |
| Prostate and other male organs | Prostate | 8.29 (6.63, 10.3) | - |
| Prostate and other male organs | Testicular | 0.23 (0.06, 0.84) | - |
| Other malignant neoplasms | Other malignant neoplasms | 0.92 (0.47, 1.81) | 0.63 (0.27, 1.46) |
| Unknown primary | Unknown primary | 5.75 (4.39, 7.51) | 6.54 (5.02, 8.48) |
| *Upper abdominal - Nausea and/or vomiting (Men=622, Women=881)* | | | |
| Head and neck | Larynx | 0.32 (0.09, 1.16) | 0.11 (0.02, 0.64) |
| Head and neck | Oral cavity | 0.00 (0.00, 0.61) | 0.00 (0.00, 0.43) |
| Head and neck | Oropharynx | 0.16 (0.03, 0.90) | 0.11 (0.02, 0.64) |
| Head and neck | Thyroid | 0.16 (0.03, 0.90) | 0.11 (0.02, 0.64) |
| Head and neck | Other head and neck | 0.32 (0.09, 1.16) | 0.00 (0.00, 0.43) |
| Upper gastrointestinal | Oesophagus | 17.4 (14.6, 20.5) | 6.58 (5.13, 8.42) |
| Upper gastrointestinal | Stomach | 10.3 (8.14, 12.9) | 7.04 (5.53, 8.92) |
| Lower gastrointestinal | Anal | 0.00 (0.00, 0.61) | 0.00 (0.00, 0.43) |
| Lower gastrointestinal | Colon | 20.1 (17.1, 23.4) | 20.0 (17.5, 22.7) |
| Lower gastrointestinal | Rectum | 1.93 (1.11, 3.34) | 1.82 (1.12, 2.93) |
| Lower gastrointestinal | Small intestine | 3.05 (1.96, 4.72) | 2.61 (1.75, 3.89) |
| Hepato-pancreato-biliary | Liver | 4.34 (3.00, 6.24) | 4.20 (3.06, 5.74) |
| Hepato-pancreato-biliary | Pancreas | 10.5 (8.28, 13.1) | 14.4 (12.3, 16.9) |
| Hepato-pancreato-biliary | Other Hepato-pancreato-biliary | 1.45 (0.76, 2.73) | 4.65 (3.45, 6.25) |
| Respiratory | Lung | 8.04 (6.15, 10.4) | 9.42 (7.66, 11.5) |
| Respiratory | Mesothelioma | 0.32 (0.09, 1.16) | 0.11 (0.02, 0.64) |
| Urological | Bladder | 0.80 (0.34, 1.87) | 0.45 (0.18, 1.16) |
| Urological | Kidney | 2.73 (1.71, 4.33) | 2.04 (1.30, 3.21) |
| Urological | Ureteric and other urinary | 0.32 (0.09, 1.16) | 0.11 (0.02, 0.64) |
| Haematological | Acute leukaemia | 1.45 (0.76, 2.73) | 1.70 (1.03, 2.79) |
| Haematological | Chronic lymphocytic leukaemia | 0.80 (0.34, 1.87) | 0.23 (0.06, 0.82) |
| Haematological | Hodgkin lymphoma | 0.32 (0.09, 1.16) | 0.11 (0.02, 0.64) |
| Haematological | Multiple myeloma | 1.13 (0.55, 2.30) | 1.36 (0.78, 2.37) |
| Haematological | Non-Hodgkin lymphoma | 2.73 (1.71, 4.33) | 3.52 (2.49, 4.95) |
| Haematological | Other haematological | 0.64 (0.25, 1.64) | 0.57 (0.24, 1.32) |
| Central nervous system | Central nervous system | 0.96 (0.44, 2.09) | 1.25 (0.70, 2.22) |
| Sarcoma | Bone sarcoma | 0.00 (0.00, 0.61) | 0.00 (0.00, 0.43) |
| Sarcoma | Connective and soft tissue sarcoma | 0.48 (0.16, 1.41) | 1.36 (0.78, 2.37) |
| Skin | Melanoma | 0.48 (0.16, 1.41) | 0.23 (0.06, 0.82) |
| Ocular | Ocular | 0.00 (0.00, 0.61) | 0.00 (0.00, 0.43) |
| Breast | Breast | 0.16 (0.03, 0.90) | 0.91 (0.46, 1.78) |
| Gynaecological | Cervix | - | 0.34 (0.12, 1.00) |
| Gynaecological | Ovary | - | 7.15 (5.63, 9.04) |
| Gynaecological | Uterus | - | 1.02 (0.54, 1.93) |
| Gynaecological | Vulva/Vagina | - | 0.11 (0.02, 0.64) |
| Prostate and other male organs | Penile | 0.00 (0.00, 0.61) | - |
| Prostate and other male organs | Prostate | 3.86 (2.61, 5.68) | - |
| Prostate and other male organs | Testicular | 0.16 (0.03, 0.90) | - |
| Other malignant neoplasms | Other malignant neoplasms | 0.32 (0.09, 1.16) | 0.68 (0.31, 1.48) |
| Unknown primary | Unknown primary | 4.34 (3.00, 6.24) | 5.68 (4.33, 7.40) |
| *Upper abdominal - New onset diabetes (Men=N<20, Women=N<20)* | | | |
| Head and neck | Larynx | - | - |
| Head and neck | Oral cavity | - | - |
| Head and neck | Oropharynx | - | - |
| Head and neck | Thyroid | - | - |
| Head and neck | Other head and neck | - | - |
| Upper gastrointestinal | Oesophagus | - | - |
| Upper gastrointestinal | Stomach | - | - |
| Lower gastrointestinal | Anal | - | - |
| Lower gastrointestinal | Colon | - | - |
| Lower gastrointestinal | Rectum | - | - |
| Lower gastrointestinal | Small intestine | - | - |
| Hepato-pancreato-biliary | Liver | - | - |
| Hepato-pancreato-biliary | Pancreas | - | - |
| Hepato-pancreato-biliary | Other Hepato-pancreato-biliary | - | - |
| Respiratory | Lung | - | - |
| Respiratory | Mesothelioma | - | - |
| Urological | Bladder | - | - |
| Urological | Kidney | - | - |
| Urological | Ureteric and other urinary | - | - |
| Haematological | Acute leukaemia | - | - |
| Haematological | Chronic lymphocytic leukaemia | - | - |
| Haematological | Hodgkin lymphoma | - | - |
| Haematological | Multiple myeloma | - | - |
| Haematological | Non-Hodgkin lymphoma | - | - |
| Haematological | Other haematological | - | - |
| Central nervous system | Central nervous system | - | - |
| Sarcoma | Bone sarcoma | - | - |
| Sarcoma | Connective and soft tissue sarcoma | - | - |
| Skin | Melanoma | - | - |
| Ocular | Ocular | - | - |
| Breast | Breast | - | - |
| Gynaecological | Cervix | - | - |
| Gynaecological | Ovary | - | - |
| Gynaecological | Uterus | - | - |
| Gynaecological | Vulva/Vagina | - | - |
| Prostate and other male organs | Penile | - | - |
| Prostate and other male organs | Prostate | - | - |
| Prostate and other male organs | Testicular | - | - |
| Other malignant neoplasms | Other malignant neoplasms | - | - |
| Unknown primary | Unknown primary | - | - |
| *Upper abdominal - Upper abdominal pain (Men=741, Women=728)* | | | |
| Head and neck | Larynx | 0.00 (0.00, 0.52) | 0.00 (0.00, 0.52) |
| Head and neck | Oral cavity | 0.00 (0.00, 0.52) | 0.00 (0.00, 0.52) |
| Head and neck | Oropharynx | 0.00 (0.00, 0.52) | 0.14 (0.02, 0.77) |
| Head and neck | Thyroid | 0.13 (0.02, 0.76) | 0.00 (0.00, 0.52) |
| Head and neck | Other head and neck | 0.00 (0.00, 0.52) | 0.00 (0.00, 0.52) |
| Upper gastrointestinal | Oesophagus | 13.9 (11.6, 16.6) | 3.98 (2.79, 5.66) |
| Upper gastrointestinal | Stomach | 14.0 (11.7, 16.7) | 6.87 (5.25, 8.94) |
| Lower gastrointestinal | Anal | 0.00 (0.00, 0.52) | 0.14 (0.02, 0.77) |
| Lower gastrointestinal | Colon | 11.2 (9.13, 13.7) | 11.7 (9.54, 14.2) |
| Lower gastrointestinal | Rectum | 1.08 (0.55, 2.12) | 1.79 (1.05, 3.03) |
| Lower gastrointestinal | Small intestine | 2.16 (1.33, 3.48) | 1.79 (1.05, 3.03) |
| Hepato-pancreato-biliary | Liver | 5.67 (4.22, 7.57) | 7.14 (5.49, 9.25) |
| Hepato-pancreato-biliary | Pancreas | 20.8 (18.0, 23.9) | 23.2 (20.3, 26.4) |
| Hepato-pancreato-biliary | Other Hepato-pancreato-biliary | 3.24 (2.19, 4.77) | 6.87 (5.25, 8.94) |
| Respiratory | Lung | 5.67 (4.22, 7.57) | 9.07 (7.19, 11.4) |
| Respiratory | Mesothelioma | 0.81 (0.37, 1.76) | 0.27 (0.08, 1.00) |
| Urological | Bladder | 0.67 (0.29, 1.57) | 0.27 (0.08, 1.00) |
| Urological | Kidney | 3.78 (2.63, 5.41) | 5.08 (3.71, 6.93) |
| Urological | Ureteric and other urinary | 0.40 (0.14, 1.18) | 0.14 (0.02, 0.77) |
| Haematological | Acute leukaemia | 0.54 (0.21, 1.38) | 0.69 (0.29, 1.60) |
| Haematological | Chronic lymphocytic leukaemia | 0.13 (0.02, 0.76) | 0.14 (0.02, 0.77) |
| Haematological | Hodgkin lymphoma | 0.40 (0.14, 1.18) | 0.00 (0.00, 0.52) |
| Haematological | Multiple myeloma | 0.67 (0.29, 1.57) | 0.55 (0.21, 1.40) |
| Haematological | Non-Hodgkin lymphoma | 4.86 (3.53, 6.65) | 2.88 (1.89, 4.37) |
| Haematological | Other haematological | 0.94 (0.46, 1.94) | 0.27 (0.08, 1.00) |
| Central nervous system | Central nervous system | 0.00 (0.00, 0.52) | 0.00 (0.00, 0.52) |
| Sarcoma | Bone sarcoma | 0.00 (0.00, 0.52) | 0.00 (0.00, 0.52) |
| Sarcoma | Connective and soft tissue sarcoma | 0.40 (0.14, 1.18) | 1.24 (0.65, 2.33) |
| Skin | Melanoma | 0.13 (0.02, 0.76) | 0.00 (0.00, 0.52) |
| Ocular | Ocular | 0.00 (0.00, 0.52) | 0.00 (0.00, 0.52) |
| Breast | Breast | 0.00 (0.00, 0.52) | 2.20 (1.36, 3.54) |
| Gynaecological | Cervix | - | 0.14 (0.02, 0.77) |
| Gynaecological | Ovary | - | 5.91 (4.41, 7.86) |
| Gynaecological | Uterus | - | 1.24 (0.65, 2.33) |
| Gynaecological | Vulva/Vagina | - | 0.00 (0.00, 0.52) |
| Prostate and other male organs | Penile | 0.00 (0.00, 0.52) | - |
| Prostate and other male organs | Prostate | 3.91 (2.74, 5.56) | - |
| Prostate and other male organs | Testicular | 0.13 (0.02, 0.76) | - |
| Other malignant neoplasms | Other malignant neoplasms | 0.54 (0.21, 1.38) | 0.96 (0.47, 1.97) |
| Unknown primary | Unknown primary | 3.78 (2.63, 5.41) | 5.36 (3.94, 7.24) |
| *Lower abdominal - Abdominal pain (NOS) (Men=1278, Women=1346)* | | | |
| Head and neck | Larynx | 0.00 (0.00, 0.30) | 0.00 (0.00, 0.28) |
| Head and neck | Oral cavity | 0.00 (0.00, 0.30) | 0.00 (0.00, 0.28) |
| Head and neck | Oropharynx | 0.00 (0.00, 0.30) | 0.00 (0.00, 0.28) |
| Head and neck | Thyroid | 0.00 (0.00, 0.30) | 0.07 (0.01, 0.42) |
| Head and neck | Other head and neck | 0.00 (0.00, 0.30) | 0.00 (0.00, 0.28) |
| Upper gastrointestinal | Oesophagus | 2.66 (1.91, 3.69) | 1.04 (0.62, 1.74) |
| Upper gastrointestinal | Stomach | 4.62 (3.60, 5.91) | 3.12 (2.32, 4.19) |
| Lower gastrointestinal | Anal | 0.16 (0.04, 0.57) | 0.67 (0.35, 1.27) |
| Lower gastrointestinal | Colon | 28.4 (26.0, 30.9) | 26.4 (24.1, 28.8) |
| Lower gastrointestinal | Rectum | 4.07 (3.12, 5.30) | 2.90 (2.13, 3.94) |
| Lower gastrointestinal | Small intestine | 2.03 (1.39, 2.96) | 1.63 (1.08, 2.46) |
| Hepato-pancreato-biliary | Liver | 5.40 (4.29, 6.78) | 2.90 (2.13, 3.94) |
| Hepato-pancreato-biliary | Pancreas | 14.0 (12.2, 16.0) | 11.7 (10.1, 13.6) |
| Hepato-pancreato-biliary | Other Hepato-pancreato-biliary | 1.72 (1.14, 2.59) | 3.34 (2.51, 4.44) |
| Respiratory | Lung | 5.95 (4.78, 7.38) | 5.42 (4.34, 6.77) |
| Respiratory | Mesothelioma | 0.55 (0.27, 1.13) | 0.07 (0.01, 0.42) |
| Urological | Bladder | 1.25 (0.77, 2.02) | 0.59 (0.30, 1.17) |
| Urological | Kidney | 5.87 (4.71, 7.29) | 3.79 (2.89, 4.95) |
| Urological | Ureteric and other urinary | 0.63 (0.32, 1.23) | 0.74 (0.40, 1.36) |
| Haematological | Acute leukaemia | 0.47 (0.22, 1.02) | 0.67 (0.35, 1.27) |
| Haematological | Chronic lymphocytic leukaemia | 1.02 (0.60, 1.73) | 0.22 (0.08, 0.65) |
| Haematological | Hodgkin lymphoma | 0.08 (0.01, 0.44) | 0.07 (0.01, 0.42) |
| Haematological | Multiple myeloma | 0.55 (0.27, 1.13) | 0.59 (0.30, 1.17) |
| Haematological | Non-Hodgkin lymphoma | 6.26 (5.06, 7.72) | 3.86 (2.96, 5.03) |
| Haematological | Other haematological | 0.63 (0.32, 1.23) | 0.59 (0.30, 1.17) |
| Central nervous system | Central nervous system | 0.00 (0.00, 0.30) | 0.07 (0.01, 0.42) |
| Sarcoma | Bone sarcoma | 0.00 (0.00, 0.30) | 0.00 (0.00, 0.28) |
| Sarcoma | Connective and soft tissue sarcoma | 0.70 (0.37, 1.33) | 1.63 (1.08, 2.46) |
| Skin | Melanoma | 0.00 (0.00, 0.30) | 0.00 (0.00, 0.28) |
| Ocular | Ocular | 0.00 (0.00, 0.30) | 0.00 (0.00, 0.28) |
| Breast | Breast | 0.00 (0.00, 0.30) | 0.82 (0.46, 1.46) |
| Gynaecological | Cervix | - | 0.52 (0.25, 1.07) |
| Gynaecological | Ovary | - | 16.4 (14.5, 18.5) |
| Gynaecological | Uterus | - | 4.09 (3.15, 5.28) |
| Gynaecological | Vulva/Vagina | - | 0.07 (0.01, 0.42) |
| Prostate and other male organs | Penile | 0.00 (0.00, 0.30) | - |
| Prostate and other male organs | Prostate | 7.67 (6.33, 9.26) | - |
| Prostate and other male organs | Testicular | 0.63 (0.32, 1.23) | - |
| Other malignant neoplasms | Other malignant neoplasms | 0.39 (0.17, 0.91) | 0.37 (0.16, 0.87) |
| Unknown primary | Unknown primary | 4.30 (3.32, 5.56) | 5.57 (4.47, 6.93) |
| *Lower abdominal - Change in bowel habit (Men=1044, Women=838)* | | | |
| Head and neck | Larynx | 0.00 (0.00, 0.37) | 0.00 (0.00, 0.46) |
| Head and neck | Oral cavity | 0.00 (0.00, 0.37) | 0.00 (0.00, 0.46) |
| Head and neck | Oropharynx | 0.00 (0.00, 0.37) | 0.00 (0.00, 0.46) |
| Head and neck | Thyroid | 0.10 (0.02, 0.54) | 0.24 (0.07, 0.87) |
| Head and neck | Other head and neck | 0.00 (0.00, 0.37) | 0.00 (0.00, 0.46) |
| Upper gastrointestinal | Oesophagus | 1.82 (1.17, 2.82) | 0.60 (0.26, 1.39) |
| Upper gastrointestinal | Stomach | 2.39 (1.63, 3.51) | 1.79 (1.09, 2.93) |
| Lower gastrointestinal | Anal | 0.86 (0.45, 1.63) | 2.98 (2.03, 4.37) |
| Lower gastrointestinal | Colon | 36.3 (33.4, 39.3) | 34.7 (31.6, 38.0) |
| Lower gastrointestinal | Rectum | 33.0 (30.2, 35.9) | 22.9 (20.2, 25.9) |
| Lower gastrointestinal | Small intestine | 0.96 (0.52, 1.75) | 1.67 (1.00, 2.78) |
| Hepato-pancreato-biliary | Liver | 2.01 (1.32, 3.06) | 1.19 (0.65, 2.18) |
| Hepato-pancreato-biliary | Pancreas | 6.32 (5.00, 7.96) | 7.16 (5.60, 9.11) |
| Hepato-pancreato-biliary | Other Hepato-pancreato-biliary | 0.48 (0.20, 1.12) | 0.72 (0.33, 1.55) |
| Respiratory | Lung | 2.30 (1.55, 3.40) | 4.06 (2.92, 5.62) |
| Respiratory | Mesothelioma | 0.38 (0.15, 0.98) | 0.12 (0.02, 0.67) |
| Urological | Bladder | 0.19 (0.05, 0.70) | 0.60 (0.26, 1.39) |
| Urological | Kidney | 1.44 (0.87, 2.36) | 1.67 (1.00, 2.78) |
| Urological | Ureteric and other urinary | 0.10 (0.02, 0.54) | 0.12 (0.02, 0.67) |
| Haematological | Acute leukaemia | 0.29 (0.10, 0.84) | 0.00 (0.00, 0.46) |
| Haematological | Chronic lymphocytic leukaemia | 0.19 (0.05, 0.70) | 0.00 (0.00, 0.46) |
| Haematological | Hodgkin lymphoma | 0.00 (0.00, 0.37) | 0.24 (0.07, 0.87) |
| Haematological | Multiple myeloma | 0.57 (0.26, 1.25) | 0.60 (0.26, 1.39) |
| Haematological | Non-Hodgkin lymphoma | 1.92 (1.24, 2.94) | 2.39 (1.55, 3.66) |
| Haematological | Other haematological | 0.29 (0.10, 0.84) | 0.48 (0.19, 1.22) |
| Central nervous system | Central nervous system | 0.00 (0.00, 0.37) | 0.00 (0.00, 0.46) |
| Sarcoma | Bone sarcoma | 0.10 (0.02, 0.54) | 0.00 (0.00, 0.46) |
| Sarcoma | Connective and soft tissue sarcoma | 0.38 (0.15, 0.98) | 0.84 (0.41, 1.71) |
| Skin | Melanoma | 0.00 (0.00, 0.37) | 0.00 (0.00, 0.46) |
| Ocular | Ocular | 0.00 (0.00, 0.37) | 0.00 (0.00, 0.46) |
| Breast | Breast | 0.00 (0.00, 0.37) | 0.95 (0.48, 1.87) |
| Gynaecological | Cervix | - | 0.24 (0.07, 0.87) |
| Gynaecological | Ovary | - | 8.35 (6.66, 10.4) |
| Gynaecological | Uterus | - | 1.07 (0.57, 2.03) |
| Gynaecological | Vulva/Vagina | - | 0.12 (0.02, 0.67) |
| Prostate and other male organs | Penile | 0.00 (0.00, 0.37) | - |
| Prostate and other male organs | Prostate | 5.65 (4.41, 7.22) | - |
| Prostate and other male organs | Testicular | 0.10 (0.02, 0.54) | - |
| Other malignant neoplasms | Other malignant neoplasms | 0.10 (0.02, 0.54) | 0.36 (0.12, 1.05) |
| Unknown primary | Unknown primary | 1.82 (1.17, 2.82) | 3.82 (2.72, 5.34) |
| *Lower abdominal - Constipation (Men=551, Women=495)* | | | |
| Head and neck | Larynx | 0.00 (0.00, 0.69) | 0.20 (0.04, 1.14) |
| Head and neck | Oral cavity | 0.00 (0.00, 0.69) | 0.00 (0.00, 0.77) |
| Head and neck | Oropharynx | 0.00 (0.00, 0.69) | 0.00 (0.00, 0.77) |
| Head and neck | Thyroid | 0.18 (0.03, 1.02) | 0.00 (0.00, 0.77) |
| Head and neck | Other head and neck | 0.18 (0.03, 1.02) | 0.00 (0.00, 0.77) |
| Upper gastrointestinal | Oesophagus | 2.54 (1.52, 4.22) | 1.62 (0.82, 3.16) |
| Upper gastrointestinal | Stomach | 4.36 (2.94, 6.40) | 1.21 (0.56, 2.62) |
| Lower gastrointestinal | Anal | 0.91 (0.39, 2.11) | 3.84 (2.47, 5.92) |
| Lower gastrointestinal | Colon | 30.7 (27.0, 34.6) | 34.3 (30.3, 38.6) |
| Lower gastrointestinal | Rectum | 13.4 (10.8, 16.5) | 11.3 (8.82, 14.4) |
| Lower gastrointestinal | Small intestine | 1.45 (0.74, 2.84) | 1.21 (0.56, 2.62) |
| Hepato-pancreato-biliary | Liver | 2.54 (1.52, 4.22) | 1.82 (0.96, 3.42) |
| Hepato-pancreato-biliary | Pancreas | 8.53 (6.48, 11.2) | 9.49 (7.22, 12.4) |
| Hepato-pancreato-biliary | Other Hepato-pancreato-biliary | 0.18 (0.03, 1.02) | 2.02 (1.10, 3.68) |
| Respiratory | Lung | 5.99 (4.30, 8.29) | 3.64 (2.31, 5.67) |
| Respiratory | Mesothelioma | 0.54 (0.19, 1.59) | 0.20 (0.04, 1.14) |
| Urological | Bladder | 1.27 (0.62, 2.60) | 1.01 (0.43, 2.34) |
| Urological | Kidney | 2.90 (1.80, 4.66) | 1.01 (0.43, 2.34) |
| Urological | Ureteric and other urinary | 0.36 (0.10, 1.31) | 0.20 (0.04, 1.14) |
| Haematological | Acute leukaemia | 0.00 (0.00, 0.69) | 0.20 (0.04, 1.14) |
| Haematological | Chronic lymphocytic leukaemia | 0.36 (0.10, 1.31) | 0.20 (0.04, 1.14) |
| Haematological | Hodgkin lymphoma | 0.00 (0.00, 0.69) | 0.00 (0.00, 0.77) |
| Haematological | Multiple myeloma | 0.73 (0.28, 1.85) | 0.20 (0.04, 1.14) |
| Haematological | Non-Hodgkin lymphoma | 3.99 (2.65, 5.97) | 2.42 (1.39, 4.19) |
| Haematological | Other haematological | 0.73 (0.28, 1.85) | 0.00 (0.00, 0.77) |
| Central nervous system | Central nervous system | 0.00 (0.00, 0.69) | 0.40 (0.11, 1.46) |
| Sarcoma | Bone sarcoma | 0.00 (0.00, 0.69) | 0.00 (0.00, 0.77) |
| Sarcoma | Connective and soft tissue sarcoma | 0.36 (0.10, 1.31) | 0.61 (0.21, 1.77) |
| Skin | Melanoma | 0.00 (0.00, 0.69) | 0.00 (0.00, 0.77) |
| Ocular | Ocular | 0.00 (0.00, 0.69) | 0.00 (0.00, 0.77) |
| Breast | Breast | 0.18 (0.03, 1.02) | 0.81 (0.31, 2.06) |
| Gynaecological | Cervix | - | 1.21 (0.56, 2.62) |
| Gynaecological | Ovary | - | 14.1 (11.3, 17.5) |
| Gynaecological | Uterus | - | 1.21 (0.56, 2.62) |
| Gynaecological | Vulva/Vagina | - | 0.00 (0.00, 0.77) |
| Prostate and other male organs | Penile | 0.00 (0.00, 0.69) | - |
| Prostate and other male organs | Prostate | 12.3 (9.85, 15.4) | - |
| Prostate and other male organs | Testicular | 0.00 (0.00, 0.69) | - |
| Other malignant neoplasms | Other malignant neoplasms | 0.36 (0.10, 1.31) | 0.40 (0.11, 1.46) |
| Unknown primary | Unknown primary | 4.90 (3.39, 7.04) | 5.05 (3.44, 7.35) |
| *Lower abdominal - Diarrhoea (Men=683, Women=616)* | | | |
| Head and neck | Larynx | 0.00 (0.00, 0.56) | 0.00 (0.00, 0.62) |
| Head and neck | Oral cavity | 0.00 (0.00, 0.56) | 0.00 (0.00, 0.62) |
| Head and neck | Oropharynx | 0.00 (0.00, 0.56) | 0.00 (0.00, 0.62) |
| Head and neck | Thyroid | 0.00 (0.00, 0.56) | 0.00 (0.00, 0.62) |
| Head and neck | Other head and neck | 0.00 (0.00, 0.56) | 0.00 (0.00, 0.62) |
| Upper gastrointestinal | Oesophagus | 0.88 (0.40, 1.90) | 0.81 (0.35, 1.89) |
| Upper gastrointestinal | Stomach | 2.64 (1.67, 4.13) | 2.60 (1.61, 4.18) |
| Lower gastrointestinal | Anal | 0.88 (0.40, 1.90) | 1.95 (1.12, 3.37) |
| Lower gastrointestinal | Colon | 33.5 (30.1, 37.2) | 34.1 (30.5, 37.9) |
| Lower gastrointestinal | Rectum | 24.9 (21.8, 28.3) | 13.5 (11.0, 16.4) |
| Lower gastrointestinal | Small intestine | 1.76 (1.01, 3.05) | 3.57 (2.37, 5.35) |
| Hepato-pancreato-biliary | Liver | 3.51 (2.37, 5.18) | 1.62 (0.88, 2.96) |
| Hepato-pancreato-biliary | Pancreas | 8.20 (6.37, 10.5) | 8.60 (6.64, 11.1) |
| Hepato-pancreato-biliary | Other Hepato-pancreato-biliary | 2.05 (1.22, 3.41) | 2.11 (1.24, 3.58) |
| Respiratory | Lung | 3.95 (2.73, 5.69) | 6.17 (4.53, 8.35) |
| Respiratory | Mesothelioma | 0.73 (0.31, 1.70) | 0.16 (0.03, 0.91) |
| Urological | Bladder | 0.73 (0.31, 1.70) | 0.65 (0.25, 1.66) |
| Urological | Kidney | 1.76 (1.01, 3.05) | 1.46 (0.77, 2.75) |
| Urological | Ureteric and other urinary | 0.15 (0.03, 0.82) | 0.16 (0.03, 0.91) |
| Haematological | Acute leukaemia | 0.73 (0.31, 1.70) | 0.16 (0.03, 0.91) |
| Haematological | Chronic lymphocytic leukaemia | 0.59 (0.23, 1.50) | 0.65 (0.25, 1.66) |
| Haematological | Hodgkin lymphoma | 0.15 (0.03, 0.82) | 0.49 (0.17, 1.42) |
| Haematological | Multiple myeloma | 0.73 (0.31, 1.70) | 1.14 (0.55, 2.33) |
| Haematological | Non-Hodgkin lymphoma | 2.64 (1.67, 4.13) | 1.95 (1.12, 3.37) |
| Haematological | Other haematological | 1.17 (0.59, 2.29) | 0.32 (0.09, 1.18) |
| Central nervous system | Central nervous system | 0.00 (0.00, 0.56) | 0.00 (0.00, 0.62) |
| Sarcoma | Bone sarcoma | 0.00 (0.00, 0.56) | 0.00 (0.00, 0.62) |
| Sarcoma | Connective and soft tissue sarcoma | 0.15 (0.03, 0.82) | 0.65 (0.25, 1.66) |
| Skin | Melanoma | 0.15 (0.03, 0.82) | 0.16 (0.03, 0.91) |
| Ocular | Ocular | 0.00 (0.00, 0.56) | 0.00 (0.00, 0.62) |
| Breast | Breast | 0.00 (0.00, 0.56) | 1.62 (0.88, 2.96) |
| Gynaecological | Cervix | - | 0.49 (0.17, 1.42) |
| Gynaecological | Ovary | - | 7.79 (5.93, 10.2) |
| Gynaecological | Uterus | - | 0.97 (0.45, 2.11) |
| Gynaecological | Vulva/Vagina | - | 0.16 (0.03, 0.91) |
| Prostate and other male organs | Penile | 0.00 (0.00, 0.56) | - |
| Prostate and other male organs | Prostate | 5.42 (3.96, 7.38) | - |
| Prostate and other male organs | Testicular | 0.15 (0.03, 0.82) | - |
| Other malignant neoplasms | Other malignant neoplasms | 0.29 (0.08, 1.06) | 0.81 (0.35, 1.89) |
| Unknown primary | Unknown primary | 2.20 (1.34, 3.59) | 5.19 (3.70, 7.24) |
| *Lower abdominal - Distension (Men=438, Women=764)* | | | |
| Head and neck | Larynx | 0.00 (0.00, 0.87) | 0.00 (0.00, 0.50) |
| Head and neck | Oral cavity | 0.00 (0.00, 0.87) | 0.00 (0.00, 0.50) |
| Head and neck | Oropharynx | 0.00 (0.00, 0.87) | 0.00 (0.00, 0.50) |
| Head and neck | Thyroid | 0.00 (0.00, 0.87) | 0.13 (0.02, 0.74) |
| Head and neck | Other head and neck | 0.00 (0.00, 0.87) | 0.00 (0.00, 0.50) |
| Upper gastrointestinal | Oesophagus | 4.11 (2.62, 6.40) | 0.52 (0.20, 1.34) |
| Upper gastrointestinal | Stomach | 5.48 (3.71, 8.02) | 1.05 (0.53, 2.05) |
| Lower gastrointestinal | Anal | 0.68 (0.23, 1.99) | 0.00 (0.00, 0.50) |
| Lower gastrointestinal | Colon | 22.6 (18.9, 26.7) | 15.4 (13.1, 18.2) |
| Lower gastrointestinal | Rectum | 6.16 (4.27, 8.82) | 3.01 (2.01, 4.48) |
| Lower gastrointestinal | Small intestine | 1.14 (0.49, 2.64) | 1.18 (0.62, 2.22) |
| Hepato-pancreato-biliary | Liver | 9.13 (6.78, 12.2) | 2.49 (1.60, 3.85) |
| Hepato-pancreato-biliary | Pancreas | 11.6 (8.97, 15.0) | 6.81 (5.23, 8.82) |
| Hepato-pancreato-biliary | Other Hepato-pancreato-biliary | 1.60 (0.78, 3.26) | 1.57 (0.90, 2.73) |
| Respiratory | Lung | 4.34 (2.79, 6.68) | 2.62 (1.70, 4.01) |
| Respiratory | Mesothelioma | 0.91 (0.36, 2.32) | 0.26 (0.07, 0.95) |
| Urological | Bladder | 0.68 (0.23, 1.99) | 0.52 (0.20, 1.34) |
| Urological | Kidney | 2.97 (1.74, 5.01) | 1.96 (1.19, 3.21) |
| Urological | Ureteric and other urinary | 0.23 (0.04, 1.28) | 0.26 (0.07, 0.95) |
| Haematological | Acute leukaemia | 0.00 (0.00, 0.87) | 0.26 (0.07, 0.95) |
| Haematological | Chronic lymphocytic leukaemia | 0.91 (0.36, 2.32) | 0.00 (0.00, 0.50) |
| Haematological | Hodgkin lymphoma | 0.00 (0.00, 0.87) | 0.00 (0.00, 0.50) |
| Haematological | Multiple myeloma | 0.46 (0.13, 1.65) | 0.26 (0.07, 0.95) |
| Haematological | Non-Hodgkin lymphoma | 5.48 (3.71, 8.02) | 2.88 (1.91, 4.32) |
| Haematological | Other haematological | 0.91 (0.36, 2.32) | 0.52 (0.20, 1.34) |
| Central nervous system | Central nervous system | 0.00 (0.00, 0.87) | 0.00 (0.00, 0.50) |
| Sarcoma | Bone sarcoma | 0.00 (0.00, 0.87) | 0.00 (0.00, 0.50) |
| Sarcoma | Connective and soft tissue sarcoma | 1.37 (0.63, 2.96) | 3.14 (2.12, 4.63) |
| Skin | Melanoma | 0.00 (0.00, 0.87) | 0.13 (0.02, 0.74) |
| Ocular | Ocular | 0.00 (0.00, 0.87) | 0.00 (0.00, 0.50) |
| Breast | Breast | 0.00 (0.00, 0.87) | 0.92 (0.44, 1.88) |
| Gynaecological | Cervix | - | 0.26 (0.07, 0.95) |
| Gynaecological | Ovary | - | 42.8 (39.3, 46.3) |
| Gynaecological | Uterus | - | 3.80 (2.66, 5.40) |
| Gynaecological | Vulva/Vagina | - | 0.00 (0.00, 0.50) |
| Prostate and other male organs | Penile | 0.00 (0.00, 0.87) | - |
| Prostate and other male organs | Prostate | 11.9 (9.17, 15.2) | - |
| Prostate and other male organs | Testicular | 0.91 (0.36, 2.32) | - |
| Other malignant neoplasms | Other malignant neoplasms | 0.23 (0.04, 1.28) | 1.05 (0.53, 2.05) |
| Unknown primary | Unknown primary | 6.16 (4.27, 8.82) | 6.15 (4.66, 8.08) |
| *Lower abdominal - Lower abdominal pain (Men=540, Women=752)* | | | |
| Head and neck | Larynx | 0.00 (0.00, 0.71) | 0.00 (0.00, 0.51) |
| Head and neck | Oral cavity | 0.00 (0.00, 0.71) | 0.00 (0.00, 0.51) |
| Head and neck | Oropharynx | 0.00 (0.00, 0.71) | 0.13 (0.02, 0.75) |
| Head and neck | Thyroid | 0.00 (0.00, 0.71) | 0.00 (0.00, 0.51) |
| Head and neck | Other head and neck | 0.00 (0.00, 0.71) | 0.27 (0.07, 0.96) |
| Upper gastrointestinal | Oesophagus | 1.48 (0.75, 2.90) | 0.40 (0.14, 1.17) |
| Upper gastrointestinal | Stomach | 1.67 (0.88, 3.14) | 1.46 (0.82, 2.60) |
| Lower gastrointestinal | Anal | 0.56 (0.19, 1.62) | 0.93 (0.45, 1.91) |
| Lower gastrointestinal | Colon | 35.7 (31.8, 39.9) | 30.2 (27.0, 33.6) |
| Lower gastrointestinal | Rectum | 8.33 (6.29, 11.0) | 4.79 (3.48, 6.56) |
| Lower gastrointestinal | Small intestine | 1.48 (0.75, 2.90) | 1.06 (0.54, 2.09) |
| Hepato-pancreato-biliary | Liver | 1.11 (0.51, 2.40) | 0.80 (0.37, 1.73) |
| Hepato-pancreato-biliary | Pancreas | 4.26 (2.85, 6.31) | 5.19 (3.82, 7.01) |
| Hepato-pancreato-biliary | Other Hepato-pancreato-biliary | 1.48 (0.75, 2.90) | 1.06 (0.54, 2.09) |
| Respiratory | Lung | 2.22 (1.28, 3.84) | 3.19 (2.15, 4.70) |
| Respiratory | Mesothelioma | 0.37 (0.10, 1.34) | 0.13 (0.02, 0.75) |
| Urological | Bladder | 4.44 (3.00, 6.53) | 2.66 (1.73, 4.07) |
| Urological | Kidney | 4.81 (3.31, 6.96) | 3.86 (2.70, 5.48) |
| Urological | Ureteric and other urinary | 0.93 (0.40, 2.15) | 0.66 (0.28, 1.55) |
| Haematological | Acute leukaemia | 0.37 (0.10, 1.34) | 0.40 (0.14, 1.17) |
| Haematological | Chronic lymphocytic leukaemia | 0.56 (0.19, 1.62) | 0.00 (0.00, 0.51) |
| Haematological | Hodgkin lymphoma | 0.19 (0.03, 1.04) | 0.13 (0.02, 0.75) |
| Haematological | Multiple myeloma | 0.56 (0.19, 1.62) | 0.27 (0.07, 0.96) |
| Haematological | Non-Hodgkin lymphoma | 3.15 (1.97, 4.98) | 2.26 (1.42, 3.59) |
| Haematological | Other haematological | 1.11 (0.51, 2.40) | 0.27 (0.07, 0.96) |
| Central nervous system | Central nervous system | 0.00 (0.00, 0.71) | 0.00 (0.00, 0.51) |
| Sarcoma | Bone sarcoma | 0.00 (0.00, 0.71) | 0.00 (0.00, 0.51) |
| Sarcoma | Connective and soft tissue sarcoma | 0.37 (0.10, 1.34) | 1.46 (0.82, 2.60) |
| Skin | Melanoma | 0.00 (0.00, 0.71) | 0.00 (0.00, 0.51) |
| Ocular | Ocular | 0.00 (0.00, 0.71) | 0.00 (0.00, 0.51) |
| Breast | Breast | 0.00 (0.00, 0.71) | 0.66 (0.28, 1.55) |
| Gynaecological | Cervix | - | 2.53 (1.62, 3.91) |
| Gynaecological | Ovary | - | 24.3 (21.4, 27.5) |
| Gynaecological | Uterus | - | 7.45 (5.78, 9.55) |
| Gynaecological | Vulva/Vagina | - | 0.00 (0.00, 0.51) |
| Prostate and other male organs | Penile | 0.00 (0.00, 0.71) | - |
| Prostate and other male organs | Prostate | 20.9 (17.7, 24.6) | - |
| Prostate and other male organs | Testicular | 0.74 (0.29, 1.89) | - |
| Other malignant neoplasms | Other malignant neoplasms | 0.37 (0.10, 1.34) | 0.40 (0.14, 1.17) |
| Unknown primary | Unknown primary | 2.78 (1.69, 4.53) | 3.06 (2.05, 4.55) |
| *Lower abdominal - Pelvic pain (Men=170, Women=252)* | | | |
| Head and neck | Larynx | 0.00 (0.00, 2.21) | 0.00 (0.00, 1.50) |
| Head and neck | Oral cavity | 0.00 (0.00, 2.21) | 0.00 (0.00, 1.50) |
| Head and neck | Oropharynx | 0.00 (0.00, 2.21) | 0.00 (0.00, 1.50) |
| Head and neck | Thyroid | 0.00 (0.00, 2.21) | 0.40 (0.07, 2.21) |
| Head and neck | Other head and neck | 0.59 (0.10, 3.26) | 0.00 (0.00, 1.50) |
| Upper gastrointestinal | Oesophagus | 0.00 (0.00, 2.21) | 0.00 (0.00, 1.50) |
| Upper gastrointestinal | Stomach | 0.00 (0.00, 2.21) | 0.00 (0.00, 1.50) |
| Lower gastrointestinal | Anal | 1.76 (0.60, 5.06) | 1.19 (0.41, 3.44) |
| Lower gastrointestinal | Colon | 8.24 (4.97, 13.3) | 7.14 (4.57, 11.0) |
| Lower gastrointestinal | Rectum | 5.29 (2.81, 9.75) | 1.98 (0.85, 4.56) |
| Lower gastrointestinal | Small intestine | 0.00 (0.00, 2.21) | 0.40 (0.07, 2.21) |
| Hepato-pancreato-biliary | Liver | 0.59 (0.10, 3.26) | 0.40 (0.07, 2.21) |
| Hepato-pancreato-biliary | Pancreas | 0.00 (0.00, 2.21) | 2.38 (1.10, 5.10) |
| Hepato-pancreato-biliary | Other Hepato-pancreato-biliary | 0.00 (0.00, 2.21) | 0.00 (0.00, 1.50) |
| Respiratory | Lung | 6.47 (3.65, 11.2) | 6.35 (3.95, 10.1) |
| Respiratory | Mesothelioma | 0.00 (0.00, 2.21) | 0.00 (0.00, 1.50) |
| Urological | Bladder | 4.12 (2.01, 8.25) | 3.97 (2.17, 7.15) |
| Urological | Kidney | 3.53 (1.63, 7.49) | 2.38 (1.10, 5.10) |
| Urological | Ureteric and other urinary | 1.18 (0.32, 4.19) | 0.40 (0.07, 2.21) |
| Haematological | Acute leukaemia | 0.59 (0.10, 3.26) | 0.00 (0.00, 1.50) |
| Haematological | Chronic lymphocytic leukaemia | 0.00 (0.00, 2.21) | 0.40 (0.07, 2.21) |
| Haematological | Hodgkin lymphoma | 0.59 (0.10, 3.26) | 0.00 (0.00, 1.50) |
| Haematological | Multiple myeloma | 2.35 (0.92, 5.89) | 1.19 (0.41, 3.44) |
| Haematological | Non-Hodgkin lymphoma | 4.71 (2.40, 9.01) | 1.98 (0.85, 4.56) |
| Haematological | Other haematological | 0.00 (0.00, 2.21) | 0.00 (0.00, 1.50) |
| Central nervous system | Central nervous system | 0.59 (0.10, 3.26) | 0.40 (0.07, 2.21) |
| Sarcoma | Bone sarcoma | 1.18 (0.32, 4.19) | 0.00 (0.00, 1.50) |
| Sarcoma | Connective and soft tissue sarcoma | 0.59 (0.10, 3.26) | 1.59 (0.62, 4.01) |
| Skin | Melanoma | 0.59 (0.10, 3.26) | 0.40 (0.07, 2.21) |
| Ocular | Ocular | 0.00 (0.00, 2.21) | 0.00 (0.00, 1.50) |
| Breast | Breast | 0.00 (0.00, 2.21) | 1.59 (0.62, 4.01) |
| Gynaecological | Cervix | - | 5.16 (3.04, 8.62) |
| Gynaecological | Ovary | - | 31.3 (25.9, 37.3) |
| Gynaecological | Uterus | - | 19.8 (15.4, 25.2) |
| Gynaecological | Vulva/Vagina | - | 0.79 (0.22, 2.85) |
| Prostate and other male organs | Penile | 0.00 (0.00, 2.21) | - |
| Prostate and other male organs | Prostate | 51.8 (44.3, 59.2) | - |
| Prostate and other male organs | Testicular | 1.76 (0.60, 5.06) | - |
| Other malignant neoplasms | Other malignant neoplasms | 0.00 (0.00, 2.21) | 0.79 (0.22, 2.85) |
| Unknown primary | Unknown primary | 3.53 (1.63, 7.49) | 7.54 (4.88, 11.5) |
| *Lower abdominal - Rectal bleeding (Men=1140, Women=864)* | | | |
| Head and neck | Larynx | 0.00 (0.00, 0.34) | 0.00 (0.00, 0.44) |
| Head and neck | Oral cavity | 0.00 (0.00, 0.34) | 0.00 (0.00, 0.44) |
| Head and neck | Oropharynx | 0.00 (0.00, 0.34) | 0.00 (0.00, 0.44) |
| Head and neck | Thyroid | 0.00 (0.00, 0.34) | 0.00 (0.00, 0.44) |
| Head and neck | Other head and neck | 0.09 (0.02, 0.50) | 0.00 (0.00, 0.44) |
| Upper gastrointestinal | Oesophagus | 0.96 (0.54, 1.72) | 0.46 (0.18, 1.18) |
| Upper gastrointestinal | Stomach | 1.75 (1.14, 2.69) | 1.85 (1.14, 2.99) |
| Lower gastrointestinal | Anal | 3.60 (2.66, 4.84) | 11.5 (9.50, 13.8) |
| Lower gastrointestinal | Colon | 38.1 (35.3, 40.9) | 35.4 (32.3, 38.7) |
| Lower gastrointestinal | Rectum | 44.5 (41.6, 47.4) | 38.7 (35.5, 41.9) |
| Lower gastrointestinal | Small intestine | 0.35 (0.14, 0.90) | 0.69 (0.32, 1.51) |
| Hepato-pancreato-biliary | Liver | 0.88 (0.48, 1.61) | 0.46 (0.18, 1.18) |
| Hepato-pancreato-biliary | Pancreas | 0.79 (0.42, 1.49) | 1.39 (0.80, 2.41) |
| Hepato-pancreato-biliary | Other Hepato-pancreato-biliary | 0.26 (0.09, 0.77) | 0.23 (0.06, 0.84) |
| Respiratory | Lung | 0.88 (0.48, 1.61) | 1.04 (0.55, 1.97) |
| Respiratory | Mesothelioma | 0.00 (0.00, 0.34) | 0.00 (0.00, 0.44) |
| Urological | Bladder | 0.18 (0.05, 0.64) | 0.12 (0.02, 0.65) |
| Urological | Kidney | 0.88 (0.48, 1.61) | 0.93 (0.47, 1.82) |
| Urological | Ureteric and other urinary | 0.09 (0.02, 0.50) | 0.00 (0.00, 0.44) |
| Haematological | Acute leukaemia | 0.26 (0.09, 0.77) | 0.12 (0.02, 0.65) |
| Haematological | Chronic lymphocytic leukaemia | 0.00 (0.00, 0.34) | 0.00 (0.00, 0.44) |
| Haematological | Hodgkin lymphoma | 0.09 (0.02, 0.50) | 0.12 (0.02, 0.65) |
| Haematological | Multiple myeloma | 0.53 (0.24, 1.14) | 0.23 (0.06, 0.84) |
| Haematological | Non-Hodgkin lymphoma | 0.96 (0.54, 1.72) | 0.81 (0.39, 1.66) |
| Haematological | Other haematological | 0.18 (0.05, 0.64) | 0.12 (0.02, 0.65) |
| Central nervous system | Central nervous system | 0.00 (0.00, 0.34) | 0.00 (0.00, 0.44) |
| Sarcoma | Bone sarcoma | 0.00 (0.00, 0.34) | 0.00 (0.00, 0.44) |
| Sarcoma | Connective and soft tissue sarcoma | 0.00 (0.00, 0.34) | 0.58 (0.25, 1.35) |
| Skin | Melanoma | 0.00 (0.00, 0.34) | 0.00 (0.00, 0.44) |
| Ocular | Ocular | 0.00 (0.00, 0.34) | 0.00 (0.00, 0.44) |
| Breast | Breast | 0.00 (0.00, 0.34) | 0.00 (0.00, 0.44) |
| Gynaecological | Cervix | - | 0.23 (0.06, 0.84) |
| Gynaecological | Ovary | - | 1.97 (1.23, 3.13) |
| Gynaecological | Uterus | - | 1.85 (1.14, 2.99) |
| Gynaecological | Vulva/Vagina | - | 0.12 (0.02, 0.65) |
| Prostate and other male organs | Penile | 0.00 (0.00, 0.34) | - |
| Prostate and other male organs | Prostate | 3.51 (2.59, 4.74) | - |
| Prostate and other male organs | Testicular | 0.09 (0.02, 0.50) | - |
| Other malignant neoplasms | Other malignant neoplasms | 0.26 (0.09, 0.77) | 0.12 (0.02, 0.65) |
| Unknown primary | Unknown primary | 0.88 (0.48, 1.61) | 1.04 (0.55, 1.97) |
| *Respiratory - Chest infection (Men=561, Women=497)* | | | |
| Head and neck | Larynx | 0.36 (0.10, 1.29) | 0.40 (0.11, 1.46) |
| Head and neck | Oral cavity | 0.00 (0.00, 0.68) | 0.00 (0.00, 0.77) |
| Head and neck | Oropharynx | 0.53 (0.18, 1.56) | 0.00 (0.00, 0.77) |
| Head and neck | Thyroid | 0.36 (0.10, 1.29) | 0.00 (0.00, 0.77) |
| Head and neck | Other head and neck | 0.00 (0.00, 0.68) | 0.20 (0.04, 1.13) |
| Upper gastrointestinal | Oesophagus | 0.36 (0.10, 1.29) | 0.40 (0.11, 1.46) |
| Upper gastrointestinal | Stomach | 0.53 (0.18, 1.56) | 0.60 (0.21, 1.76) |
| Lower gastrointestinal | Anal | 0.00 (0.00, 0.68) | 0.00 (0.00, 0.77) |
| Lower gastrointestinal | Colon | 1.60 (0.85, 3.02) | 0.60 (0.21, 1.76) |
| Lower gastrointestinal | Rectum | 0.53 (0.18, 1.56) | 0.00 (0.00, 0.77) |
| Lower gastrointestinal | Small intestine | 0.00 (0.00, 0.68) | 0.00 (0.00, 0.77) |
| Hepato-pancreato-biliary | Liver | 0.36 (0.10, 1.29) | 0.40 (0.11, 1.46) |
| Hepato-pancreato-biliary | Pancreas | 0.89 (0.38, 2.07) | 0.60 (0.21, 1.76) |
| Hepato-pancreato-biliary | Other Hepato-pancreato-biliary | 0.00 (0.00, 0.68) | 0.00 (0.00, 0.77) |
| Respiratory | Lung | 74.0 (70.2, 77.4) | 81.7 (78.1, 84.8) |
| Respiratory | Mesothelioma | 5.88 (4.22, 8.15) | 1.01 (0.43, 2.33) |
| Urological | Bladder | 0.36 (0.10, 1.29) | 0.00 (0.00, 0.77) |
| Urological | Kidney | 0.53 (0.18, 1.56) | 1.41 (0.68, 2.88) |
| Urological | Ureteric and other urinary | 0.00 (0.00, 0.68) | 0.00 (0.00, 0.77) |
| Haematological | Acute leukaemia | 1.96 (1.10, 3.48) | 1.41 (0.68, 2.88) |
| Haematological | Chronic lymphocytic leukaemia | 0.89 (0.38, 2.07) | 0.20 (0.04, 1.13) |
| Haematological | Hodgkin lymphoma | 0.18 (0.03, 1.00) | 0.20 (0.04, 1.13) |
| Haematological | Multiple myeloma | 1.07 (0.49, 2.31) | 1.21 (0.55, 2.61) |
| Haematological | Non-Hodgkin lymphoma | 2.67 (1.63, 4.36) | 2.41 (1.39, 4.17) |
| Haematological | Other haematological | 1.60 (0.85, 3.02) | 0.60 (0.21, 1.76) |
| Central nervous system | Central nervous system | 0.18 (0.03, 1.00) | 0.00 (0.00, 0.77) |
| Sarcoma | Bone sarcoma | 0.00 (0.00, 0.68) | 0.00 (0.00, 0.77) |
| Sarcoma | Connective and soft tissue sarcoma | 0.00 (0.00, 0.68) | 0.20 (0.04, 1.13) |
| Skin | Melanoma | 0.18 (0.03, 1.00) | 0.00 (0.00, 0.77) |
| Ocular | Ocular | 0.00 (0.00, 0.68) | 0.00 (0.00, 0.77) |
| Breast | Breast | 0.00 (0.00, 0.68) | 1.81 (0.96, 3.41) |
| Gynaecological | Cervix | - | 0.00 (0.00, 0.77) |
| Gynaecological | Ovary | - | 1.41 (0.68, 2.88) |
| Gynaecological | Uterus | - | 0.20 (0.04, 1.13) |
| Gynaecological | Vulva/Vagina | - | 0.00 (0.00, 0.77) |
| Prostate and other male organs | Penile | 0.00 (0.00, 0.68) | - |
| Prostate and other male organs | Prostate | 2.14 (1.23, 3.70) | - |
| Prostate and other male organs | Testicular | 0.18 (0.03, 1.00) | - |
| Other malignant neoplasms | Other malignant neoplasms | 1.25 (0.61, 2.55) | 0.40 (0.11, 1.46) |
| Unknown primary | Unknown primary | 1.43 (0.72, 2.79) | 2.62 (1.53, 4.42) |
| *Respiratory - Chest pain (Men=750, Women=579)* | | | |
| Head and neck | Larynx | 0.00 (0.00, 0.51) | 0.00 (0.00, 0.66) |
| Head and neck | Oral cavity | 0.00 (0.00, 0.51) | 0.00 (0.00, 0.66) |
| Head and neck | Oropharynx | 0.40 (0.14, 1.17) | 0.35 (0.09, 1.25) |
| Head and neck | Thyroid | 0.13 (0.02, 0.75) | 0.69 (0.27, 1.76) |
| Head and neck | Other head and neck | 0.13 (0.02, 0.75) | 0.17 (0.03, 0.97) |
| Upper gastrointestinal | Oesophagus | 5.47 (4.06, 7.33) | 3.11 (1.98, 4.86) |
| Upper gastrointestinal | Stomach | 1.60 (0.92, 2.78) | 1.21 (0.59, 2.47) |
| Lower gastrointestinal | Anal | 0.00 (0.00, 0.51) | 0.00 (0.00, 0.66) |
| Lower gastrointestinal | Colon | 2.40 (1.52, 3.76) | 1.73 (0.94, 3.15) |
| Lower gastrointestinal | Rectum | 0.13 (0.02, 0.75) | 0.52 (0.18, 1.51) |
| Lower gastrointestinal | Small intestine | 0.00 (0.00, 0.51) | 0.00 (0.00, 0.66) |
| Hepato-pancreato-biliary | Liver | 0.67 (0.29, 1.55) | 1.55 (0.82, 2.93) |
| Hepato-pancreato-biliary | Pancreas | 2.53 (1.63, 3.92) | 3.45 (2.25, 5.27) |
| Hepato-pancreato-biliary | Other Hepato-pancreato-biliary | 0.93 (0.45, 1.91) | 0.35 (0.09, 1.25) |
| Respiratory | Lung | 51.2 (47.6, 54.8) | 61.0 (56.9, 64.9) |
| Respiratory | Mesothelioma | 10.8 (8.78, 13.2) | 2.42 (1.45, 4.02) |
| Urological | Bladder | 0.27 (0.07, 0.97) | 0.00 (0.00, 0.66) |
| Urological | Kidney | 2.53 (1.63, 3.92) | 1.38 (0.70, 2.70) |
| Urological | Ureteric and other urinary | 0.13 (0.02, 0.75) | 0.00 (0.00, 0.66) |
| Haematological | Acute leukaemia | 2.40 (1.52, 3.76) | 0.69 (0.27, 1.76) |
| Haematological | Chronic lymphocytic leukaemia | 1.07 (0.54, 2.09) | 0.52 (0.18, 1.51) |
| Haematological | Hodgkin lymphoma | 0.40 (0.14, 1.17) | 0.35 (0.09, 1.25) |
| Haematological | Multiple myeloma | 4.00 (2.82, 5.65) | 2.76 (1.71, 4.44) |
| Haematological | Non-Hodgkin lymphoma | 3.07 (2.05, 4.56) | 2.07 (1.19, 3.59) |
| Haematological | Other haematological | 1.20 (0.63, 2.26) | 1.04 (0.48, 2.24) |
| Central nervous system | Central nervous system | 0.13 (0.02, 0.75) | 0.00 (0.00, 0.66) |
| Sarcoma | Bone sarcoma | 0.13 (0.02, 0.75) | 0.00 (0.00, 0.66) |
| Sarcoma | Connective and soft tissue sarcoma | 0.00 (0.00, 0.51) | 0.52 (0.18, 1.51) |
| Skin | Melanoma | 0.53 (0.21, 1.36) | 0.35 (0.09, 1.25) |
| Ocular | Ocular | 0.00 (0.00, 0.51) | 0.00 (0.00, 0.66) |
| Breast | Breast | 0.27 (0.07, 0.97) | 7.60 (5.71, 10.0) |
| Gynaecological | Cervix | - | 0.17 (0.03, 0.97) |
| Gynaecological | Ovary | - | 2.42 (1.45, 4.02) |
| Gynaecological | Uterus | - | 0.00 (0.00, 0.66) |
| Gynaecological | Vulva/Vagina | - | 0.00 (0.00, 0.66) |
| Prostate and other male organs | Penile | 0.00 (0.00, 0.51) | - |
| Prostate and other male organs | Prostate | 4.27 (3.04, 5.96) | - |
| Prostate and other male organs | Testicular | 0.13 (0.02, 0.75) | - |
| Other malignant neoplasms | Other malignant neoplasms | 0.93 (0.45, 1.91) | 0.52 (0.18, 1.51) |
| Unknown primary | Unknown primary | 2.13 (1.32, 3.44) | 3.11 (1.98, 4.86) |
| *Respiratory - Clubbing (Men=32, Women=N<20)* | | | |
| Head and neck | Larynx | 0.00 (0.00, 10.7) | - |
| Head and neck | Oral cavity | 0.00 (0.00, 10.7) | - |
| Head and neck | Oropharynx | 0.00 (0.00, 10.7) | - |
| Head and neck | Thyroid | 0.00 (0.00, 10.7) | - |
| Head and neck | Other head and neck | 0.00 (0.00, 10.7) | - |
| Upper gastrointestinal | Oesophagus | 3.12 (0.55, 15.7) | - |
| Upper gastrointestinal | Stomach | 0.00 (0.00, 10.7) | - |
| Lower gastrointestinal | Anal | 0.00 (0.00, 10.7) | - |
| Lower gastrointestinal | Colon | 6.25 (1.73, 20.1) | - |
| Lower gastrointestinal | Rectum | 0.00 (0.00, 10.7) | - |
| Lower gastrointestinal | Small intestine | 0.00 (0.00, 10.7) | - |
| Hepato-pancreato-biliary | Liver | 0.00 (0.00, 10.7) | - |
| Hepato-pancreato-biliary | Pancreas | 0.00 (0.00, 10.7) | - |
| Hepato-pancreato-biliary | Other Hepato-pancreato-biliary | 0.00 (0.00, 10.7) | - |
| Respiratory | Lung | 68.8 (51.4, 82.0) | - |
| Respiratory | Mesothelioma | 3.12 (0.55, 15.7) | - |
| Urological | Bladder | 0.00 (0.00, 10.7) | - |
| Urological | Kidney | 0.00 (0.00, 10.7) | - |
| Urological | Ureteric and other urinary | 0.00 (0.00, 10.7) | - |
| Haematological | Acute leukaemia | 3.12 (0.55, 15.7) | - |
| Haematological | Chronic lymphocytic leukaemia | 0.00 (0.00, 10.7) | - |
| Haematological | Hodgkin lymphoma | 0.00 (0.00, 10.7) | - |
| Haematological | Multiple myeloma | 0.00 (0.00, 10.7) | - |
| Haematological | Non-Hodgkin lymphoma | 0.00 (0.00, 10.7) | - |
| Haematological | Other haematological | 0.00 (0.00, 10.7) | - |
| Central nervous system | Central nervous system | 0.00 (0.00, 10.7) | - |
| Sarcoma | Bone sarcoma | 0.00 (0.00, 10.7) | - |
| Sarcoma | Connective and soft tissue sarcoma | 0.00 (0.00, 10.7) | - |
| Skin | Melanoma | 0.00 (0.00, 10.7) | - |
| Ocular | Ocular | 0.00 (0.00, 10.7) | - |
| Breast | Breast | 0.00 (0.00, 10.7) | - |
| Gynaecological | Cervix | - | - |
| Gynaecological | Ovary | - | - |
| Gynaecological | Uterus | - | - |
| Gynaecological | Vulva/Vagina | - | - |
| Prostate and other male organs | Penile | 3.12 (0.55, 15.7) | - |
| Prostate and other male organs | Prostate | 9.38 (3.24, 24.2) | - |
| Prostate and other male organs | Testicular | 0.00 (0.00, 10.7) | - |
| Other malignant neoplasms | Other malignant neoplasms | 0.00 (0.00, 10.7) | - |
| Unknown primary | Unknown primary | 3.12 (0.55, 15.7) | - |
| *Respiratory - Cough (Men=1703, Women=1380)* | | | |
| Head and neck | Larynx | 1.47 (1.00, 2.16) | 0.72 (0.39, 1.33) |
| Head and neck | Oral cavity | 0.23 (0.09, 0.60) | 0.22 (0.07, 0.64) |
| Head and neck | Oropharynx | 1.00 (0.62, 1.59) | 0.14 (0.04, 0.53) |
| Head and neck | Thyroid | 0.70 (0.40, 1.23) | 0.65 (0.34, 1.23) |
| Head and neck | Other head and neck | 0.18 (0.06, 0.52) | 0.36 (0.15, 0.85) |
| Upper gastrointestinal | Oesophagus | 2.00 (1.43, 2.78) | 0.58 (0.29, 1.14) |
| Upper gastrointestinal | Stomach | 0.65 (0.36, 1.15) | 0.43 (0.20, 0.95) |
| Lower gastrointestinal | Anal | 0.00 (0.00, 0.23) | 0.00 (0.00, 0.28) |
| Lower gastrointestinal | Colon | 1.76 (1.24, 2.50) | 1.30 (0.83, 2.05) |
| Lower gastrointestinal | Rectum | 0.35 (0.16, 0.77) | 0.22 (0.07, 0.64) |
| Lower gastrointestinal | Small intestine | 0.06 (0.01, 0.33) | 0.07 (0.01, 0.41) |
| Hepato-pancreato-biliary | Liver | 0.35 (0.16, 0.77) | 0.29 (0.11, 0.74) |
| Hepato-pancreato-biliary | Pancreas | 0.59 (0.32, 1.08) | 0.72 (0.39, 1.33) |
| Hepato-pancreato-biliary | Other Hepato-pancreato-biliary | 0.12 (0.03, 0.43) | 0.14 (0.04, 0.53) |
| Respiratory | Lung | 68.4 (66.2, 70.6) | 79.9 (77.7, 81.9) |
| Respiratory | Mesothelioma | 7.52 (6.36, 8.87) | 1.81 (1.23, 2.66) |
| Urological | Bladder | 0.18 (0.06, 0.52) | 0.22 (0.07, 0.64) |
| Urological | Kidney | 2.11 (1.53, 2.91) | 1.16 (0.71, 1.88) |
| Urological | Ureteric and other urinary | 0.00 (0.00, 0.23) | 0.07 (0.01, 0.41) |
| Haematological | Acute leukaemia | 0.88 (0.53, 1.45) | 0.58 (0.29, 1.14) |
| Haematological | Chronic lymphocytic leukaemia | 0.59 (0.32, 1.08) | 0.07 (0.01, 0.41) |
| Haematological | Hodgkin lymphoma | 0.70 (0.40, 1.23) | 0.51 (0.25, 1.04) |
| Haematological | Multiple myeloma | 1.23 (0.81, 1.88) | 0.65 (0.34, 1.23) |
| Haematological | Non-Hodgkin lymphoma | 2.82 (2.13, 3.72) | 2.97 (2.20, 4.01) |
| Haematological | Other haematological | 1.29 (0.85, 1.95) | 0.87 (0.50, 1.51) |
| Central nervous system | Central nervous system | 0.06 (0.01, 0.33) | 0.14 (0.04, 0.53) |
| Sarcoma | Bone sarcoma | 0.00 (0.00, 0.23) | 0.00 (0.00, 0.28) |
| Sarcoma | Connective and soft tissue sarcoma | 0.23 (0.09, 0.60) | 0.29 (0.11, 0.74) |
| Skin | Melanoma | 0.47 (0.24, 0.92) | 0.14 (0.04, 0.53) |
| Ocular | Ocular | 0.00 (0.00, 0.23) | 0.00 (0.00, 0.28) |
| Breast | Breast | 0.06 (0.01, 0.33) | 2.03 (1.41, 2.92) |
| Gynaecological | Cervix | - | 0.00 (0.00, 0.28) |
| Gynaecological | Ovary | - | 0.94 (0.55, 1.61) |
| Gynaecological | Uterus | - | 0.43 (0.20, 0.95) |
| Gynaecological | Vulva/Vagina | - | 0.07 (0.01, 0.41) |
| Prostate and other male organs | Penile | 0.00 (0.00, 0.23) | - |
| Prostate and other male organs | Prostate | 1.88 (1.33, 2.64) | - |
| Prostate and other male organs | Testicular | 0.06 (0.01, 0.33) | - |
| Other malignant neoplasms | Other malignant neoplasms | 0.35 (0.16, 0.77) | 0.14 (0.04, 0.53) |
| Unknown primary | Unknown primary | 1.70 (1.19, 2.43) | 1.16 (0.71, 1.88) |
| *Respiratory - Dyspnoea (Men=1306, Women=1111)* | | | |
| Head and neck | Larynx | 0.77 (0.42, 1.40) | 0.36 (0.14, 0.92) |
| Head and neck | Oral cavity | 0.15 (0.04, 0.56) | 0.09 (0.02, 0.51) |
| Head and neck | Oropharynx | 0.23 (0.08, 0.67) | 0.09 (0.02, 0.51) |
| Head and neck | Thyroid | 0.15 (0.04, 0.56) | 0.99 (0.55, 1.76) |
| Head and neck | Other head and neck | 0.00 (0.00, 0.29) | 0.18 (0.05, 0.65) |
| Upper gastrointestinal | Oesophagus | 1.45 (0.93, 2.26) | 0.72 (0.37, 1.41) |
| Upper gastrointestinal | Stomach | 1.45 (0.93, 2.26) | 1.26 (0.75, 2.10) |
| Lower gastrointestinal | Anal | 0.08 (0.01, 0.43) | 0.00 (0.00, 0.34) |
| Lower gastrointestinal | Colon | 6.05 (4.88, 7.48) | 7.74 (6.31, 9.46) |
| Lower gastrointestinal | Rectum | 0.61 (0.31, 1.20) | 0.18 (0.05, 0.65) |
| Lower gastrointestinal | Small intestine | 0.15 (0.04, 0.56) | 0.27 (0.09, 0.79) |
| Hepato-pancreato-biliary | Liver | 1.15 (0.70, 1.89) | 1.08 (0.62, 1.88) |
| Hepato-pancreato-biliary | Pancreas | 1.15 (0.70, 1.89) | 0.81 (0.43, 1.53) |
| Hepato-pancreato-biliary | Other Hepato-pancreato-biliary | 0.15 (0.04, 0.56) | 0.36 (0.14, 0.92) |
| Respiratory | Lung | 53.4 (50.7, 56.1) | 61.7 (58.8, 64.5) |
| Respiratory | Mesothelioma | 13.5 (11.7, 15.4) | 3.06 (2.20, 4.25) |
| Urological | Bladder | 0.38 (0.16, 0.89) | 0.36 (0.14, 0.92) |
| Urological | Kidney | 2.22 (1.55, 3.17) | 1.08 (0.62, 1.88) |
| Urological | Ureteric and other urinary | 0.15 (0.04, 0.56) | 0.00 (0.00, 0.34) |
| Haematological | Acute leukaemia | 2.30 (1.61, 3.26) | 3.06 (2.20, 4.25) |
| Haematological | Chronic lymphocytic leukaemia | 1.00 (0.58, 1.70) | 0.18 (0.05, 0.65) |
| Haematological | Hodgkin lymphoma | 0.23 (0.08, 0.67) | 0.09 (0.02, 0.51) |
| Haematological | Multiple myeloma | 1.68 (1.12, 2.54) | 1.26 (0.75, 2.10) |
| Haematological | Non-Hodgkin lymphoma | 4.44 (3.45, 5.70) | 3.15 (2.27, 4.35) |
| Haematological | Other haematological | 1.23 (0.76, 1.98) | 0.72 (0.37, 1.41) |
| Central nervous system | Central nervous system | 0.00 (0.00, 0.29) | 0.00 (0.00, 0.34) |
| Sarcoma | Bone sarcoma | 0.00 (0.00, 0.29) | 0.00 (0.00, 0.34) |
| Sarcoma | Connective and soft tissue sarcoma | 0.15 (0.04, 0.56) | 0.36 (0.14, 0.92) |
| Skin | Melanoma | 0.08 (0.01, 0.43) | 0.09 (0.02, 0.51) |
| Ocular | Ocular | 0.00 (0.00, 0.29) | 0.00 (0.00, 0.34) |
| Breast | Breast | 0.00 (0.00, 0.29) | 2.43 (1.68, 3.51) |
| Gynaecological | Cervix | - | 0.09 (0.02, 0.51) |
| Gynaecological | Ovary | - | 3.60 (2.66, 4.87) |
| Gynaecological | Uterus | - | 0.54 (0.25, 1.17) |
| Gynaecological | Vulva/Vagina | - | 0.00 (0.00, 0.34) |
| Prostate and other male organs | Penile | 0.00 (0.00, 0.29) | - |
| Prostate and other male organs | Prostate | 2.68 (1.93, 3.70) | - |
| Prostate and other male organs | Testicular | 0.08 (0.01, 0.43) | - |
| Other malignant neoplasms | Other malignant neoplasms | 0.61 (0.31, 1.20) | 0.72 (0.37, 1.41) |
| Unknown primary | Unknown primary | 2.37 (1.68, 3.35) | 3.42 (2.50, 4.66) |
| *Respiratory - Haemoptysis (Men=361, Women=214)* | | | |
| Head and neck | Larynx | 0.55 (0.15, 2.00) | 0.93 (0.26, 3.34) |
| Head and neck | Oral cavity | 0.00 (0.00, 1.05) | 0.00 (0.00, 1.76) |
| Head and neck | Oropharynx | 1.94 (0.94, 3.95) | 0.00 (0.00, 1.76) |
| Head and neck | Thyroid | 0.28 (0.05, 1.55) | 0.47 (0.08, 2.60) |
| Head and neck | Other head and neck | 0.55 (0.15, 2.00) | 0.00 (0.00, 1.76) |
| Upper gastrointestinal | Oesophagus | 0.55 (0.15, 2.00) | 0.00 (0.00, 1.76) |
| Upper gastrointestinal | Stomach | 0.28 (0.05, 1.55) | 0.47 (0.08, 2.60) |
| Lower gastrointestinal | Anal | 0.00 (0.00, 1.05) | 0.47 (0.08, 2.60) |
| Lower gastrointestinal | Colon | 0.83 (0.28, 2.41) | 0.47 (0.08, 2.60) |
| Lower gastrointestinal | Rectum | 0.00 (0.00, 1.05) | 0.47 (0.08, 2.60) |
| Lower gastrointestinal | Small intestine | 0.00 (0.00, 1.05) | 0.00 (0.00, 1.76) |
| Hepato-pancreato-biliary | Liver | 0.83 (0.28, 2.41) | 1.87 (0.73, 4.71) |
| Hepato-pancreato-biliary | Pancreas | 0.28 (0.05, 1.55) | 0.00 (0.00, 1.76) |
| Hepato-pancreato-biliary | Other Hepato-pancreato-biliary | 0.00 (0.00, 1.05) | 0.00 (0.00, 1.76) |
| Respiratory | Lung | 84.2 (80.1, 87.6) | 89.7 (84.9, 93.1) |
| Respiratory | Mesothelioma | 1.66 (0.76, 3.58) | 0.00 (0.00, 1.76) |
| Urological | Bladder | 0.28 (0.05, 1.55) | 0.00 (0.00, 1.76) |
| Urological | Kidney | 3.05 (1.71, 5.37) | 0.93 (0.26, 3.34) |
| Urological | Ureteric and other urinary | 0.00 (0.00, 1.05) | 0.00 (0.00, 1.76) |
| Haematological | Acute leukaemia | 0.00 (0.00, 1.05) | 1.40 (0.48, 4.04) |
| Haematological | Chronic lymphocytic leukaemia | 0.00 (0.00, 1.05) | 0.00 (0.00, 1.76) |
| Haematological | Hodgkin lymphoma | 0.00 (0.00, 1.05) | 0.00 (0.00, 1.76) |
| Haematological | Multiple myeloma | 0.55 (0.15, 2.00) | 0.47 (0.08, 2.60) |
| Haematological | Non-Hodgkin lymphoma | 1.39 (0.59, 3.20) | 0.93 (0.26, 3.34) |
| Haematological | Other haematological | 1.11 (0.43, 2.81) | 0.00 (0.00, 1.76) |
| Central nervous system | Central nervous system | 0.00 (0.00, 1.05) | 0.00 (0.00, 1.76) |
| Sarcoma | Bone sarcoma | 0.00 (0.00, 1.05) | 0.00 (0.00, 1.76) |
| Sarcoma | Connective and soft tissue sarcoma | 0.28 (0.05, 1.55) | 0.00 (0.00, 1.76) |
| Skin | Melanoma | 0.00 (0.00, 1.05) | 0.00 (0.00, 1.76) |
| Ocular | Ocular | 0.00 (0.00, 1.05) | 0.00 (0.00, 1.76) |
| Breast | Breast | 0.00 (0.00, 1.05) | 0.47 (0.08, 2.60) |
| Gynaecological | Cervix | - | 0.00 (0.00, 1.76) |
| Gynaecological | Ovary | - | 0.00 (0.00, 1.76) |
| Gynaecological | Uterus | - | 0.47 (0.08, 2.60) |
| Gynaecological | Vulva/Vagina | - | 0.00 (0.00, 1.76) |
| Prostate and other male organs | Penile | 0.00 (0.00, 1.05) | - |
| Prostate and other male organs | Prostate | 0.55 (0.15, 2.00) | - |
| Prostate and other male organs | Testicular | 0.28 (0.05, 1.55) | - |
| Other malignant neoplasms | Other malignant neoplasms | 0.28 (0.05, 1.55) | 0.00 (0.00, 1.76) |
| Unknown primary | Unknown primary | 0.28 (0.05, 1.55) | 0.47 (0.08, 2.60) |
| *Respiratory - Hoarseness (Men=390, Women=141)* | | | |
| Head and neck | Larynx | 53.1 (48.1, 58.0) | 32.6 (25.4, 40.7) |
| Head and neck | Oral cavity | 0.77 (0.26, 2.24) | 2.13 (0.73, 6.07) |
| Head and neck | Oropharynx | 6.15 (4.17, 8.99) | 2.13 (0.73, 6.07) |
| Head and neck | Thyroid | 3.08 (1.77, 5.30) | 9.93 (6.01, 16.0) |
| Head and neck | Other head and neck | 7.18 (5.01, 10.2) | 4.96 (2.43, 9.89) |
| Upper gastrointestinal | Oesophagus | 2.31 (1.22, 4.33) | 1.42 (0.39, 5.02) |
| Upper gastrointestinal | Stomach | 0.00 (0.00, 0.98) | 0.00 (0.00, 2.65) |
| Lower gastrointestinal | Anal | 0.00 (0.00, 0.98) | 0.00 (0.00, 2.65) |
| Lower gastrointestinal | Colon | 1.28 (0.55, 2.97) | 0.00 (0.00, 2.65) |
| Lower gastrointestinal | Rectum | 0.00 (0.00, 0.98) | 0.00 (0.00, 2.65) |
| Lower gastrointestinal | Small intestine | 0.00 (0.00, 0.98) | 0.00 (0.00, 2.65) |
| Hepato-pancreato-biliary | Liver | 0.00 (0.00, 0.98) | 0.00 (0.00, 2.65) |
| Hepato-pancreato-biliary | Pancreas | 0.77 (0.26, 2.24) | 0.00 (0.00, 2.65) |
| Hepato-pancreato-biliary | Other Hepato-pancreato-biliary | 0.00 (0.00, 0.98) | 0.00 (0.00, 2.65) |
| Respiratory | Lung | 21.0 (17.3, 25.3) | 34.0 (26.7, 42.2) |
| Respiratory | Mesothelioma | 0.26 (0.05, 1.44) | 0.00 (0.00, 2.65) |
| Urological | Bladder | 0.00 (0.00, 0.98) | 0.00 (0.00, 2.65) |
| Urological | Kidney | 0.00 (0.00, 0.98) | 1.42 (0.39, 5.02) |
| Urological | Ureteric and other urinary | 0.00 (0.00, 0.98) | 0.00 (0.00, 2.65) |
| Haematological | Acute leukaemia | 0.00 (0.00, 0.98) | 0.00 (0.00, 2.65) |
| Haematological | Chronic lymphocytic leukaemia | 0.00 (0.00, 0.98) | 0.00 (0.00, 2.65) |
| Haematological | Hodgkin lymphoma | 0.26 (0.05, 1.44) | 1.42 (0.39, 5.02) |
| Haematological | Multiple myeloma | 0.26 (0.05, 1.44) | 0.00 (0.00, 2.65) |
| Haematological | Non-Hodgkin lymphoma | 0.77 (0.26, 2.24) | 7.09 (3.90, 12.6) |
| Haematological | Other haematological | 0.00 (0.00, 0.98) | 1.42 (0.39, 5.02) |
| Central nervous system | Central nervous system | 0.26 (0.05, 1.44) | 0.00 (0.00, 2.65) |
| Sarcoma | Bone sarcoma | 0.00 (0.00, 0.98) | 0.00 (0.00, 2.65) |
| Sarcoma | Connective and soft tissue sarcoma | 0.00 (0.00, 0.98) | 0.00 (0.00, 2.65) |
| Skin | Melanoma | 0.00 (0.00, 0.98) | 0.00 (0.00, 2.65) |
| Ocular | Ocular | 0.00 (0.00, 0.98) | 0.00 (0.00, 2.65) |
| Breast | Breast | 0.00 (0.00, 0.98) | 0.71 (0.13, 3.91) |
| Gynaecological | Cervix | - | 0.00 (0.00, 2.65) |
| Gynaecological | Ovary | - | 0.00 (0.00, 2.65) |
| Gynaecological | Uterus | - | 0.00 (0.00, 2.65) |
| Gynaecological | Vulva/Vagina | - | 0.00 (0.00, 2.65) |
| Prostate and other male organs | Penile | 0.00 (0.00, 0.98) | - |
| Prostate and other male organs | Prostate | 0.26 (0.05, 1.44) | - |
| Prostate and other male organs | Testicular | 0.00 (0.00, 0.98) | - |
| Other malignant neoplasms | Other malignant neoplasms | 0.51 (0.14, 1.85) | 0.00 (0.00, 2.65) |
| Unknown primary | Unknown primary | 1.79 (0.87, 3.66) | 0.71 (0.13, 3.91) |
| *Respiratory - Sore throat (Men=360, Women=171)* | | | |
| Head and neck | Larynx | 14.2 (10.9, 18.1) | 10.5 (6.76, 16.0) |
| Head and neck | Oral cavity | 5.83 (3.85, 8.75) | 6.43 (3.63, 11.2) |
| Head and neck | Oropharynx | 36.7 (31.9, 41.8) | 29.8 (23.5, 37.1) |
| Head and neck | Thyroid | 3.06 (1.71, 5.39) | 9.36 (5.84, 14.7) |
| Head and neck | Other head and neck | 12.2 (9.23, 16.0) | 7.02 (4.06, 11.9) |
| Upper gastrointestinal | Oesophagus | 3.89 (2.33, 6.42) | 4.68 (2.39, 8.96) |
| Upper gastrointestinal | Stomach | 0.28 (0.05, 1.56) | 0.00 (0.00, 2.20) |
| Lower gastrointestinal | Anal | 0.00 (0.00, 1.06) | 0.00 (0.00, 2.20) |
| Lower gastrointestinal | Colon | 0.56 (0.15, 2.00) | 0.58 (0.10, 3.24) |
| Lower gastrointestinal | Rectum | 0.00 (0.00, 1.06) | 0.00 (0.00, 2.20) |
| Lower gastrointestinal | Small intestine | 0.00 (0.00, 1.06) | 0.00 (0.00, 2.20) |
| Hepato-pancreato-biliary | Liver | 0.56 (0.15, 2.00) | 0.58 (0.10, 3.24) |
| Hepato-pancreato-biliary | Pancreas | 0.00 (0.00, 1.06) | 0.00 (0.00, 2.20) |
| Hepato-pancreato-biliary | Other Hepato-pancreato-biliary | 0.00 (0.00, 1.06) | 0.00 (0.00, 2.20) |
| Respiratory | Lung | 7.22 (4.98, 10.4) | 15.2 (10.6, 21.3) |
| Respiratory | Mesothelioma | 0.00 (0.00, 1.06) | 0.58 (0.10, 3.24) |
| Urological | Bladder | 0.00 (0.00, 1.06) | 0.00 (0.00, 2.20) |
| Urological | Kidney | 0.56 (0.15, 2.00) | 0.00 (0.00, 2.20) |
| Urological | Ureteric and other urinary | 0.28 (0.05, 1.56) | 0.00 (0.00, 2.20) |
| Haematological | Acute leukaemia | 2.22 (1.13, 4.32) | 4.68 (2.39, 8.96) |
| Haematological | Chronic lymphocytic leukaemia | 0.83 (0.28, 2.42) | 0.00 (0.00, 2.20) |
| Haematological | Hodgkin lymphoma | 0.83 (0.28, 2.42) | 0.58 (0.10, 3.24) |
| Haematological | Multiple myeloma | 0.56 (0.15, 2.00) | 0.58 (0.10, 3.24) |
| Haematological | Non-Hodgkin lymphoma | 8.06 (5.67, 11.3) | 5.85 (3.21, 10.4) |
| Haematological | Other haematological | 0.00 (0.00, 1.06) | 2.34 (0.91, 5.86) |
| Central nervous system | Central nervous system | 0.00 (0.00, 1.06) | 0.58 (0.10, 3.24) |
| Sarcoma | Bone sarcoma | 0.00 (0.00, 1.06) | 0.00 (0.00, 2.20) |
| Sarcoma | Connective and soft tissue sarcoma | 0.28 (0.05, 1.56) | 0.00 (0.00, 2.20) |
| Skin | Melanoma | 0.28 (0.05, 1.56) | 0.00 (0.00, 2.20) |
| Ocular | Ocular | 0.00 (0.00, 1.06) | 0.00 (0.00, 2.20) |
| Breast | Breast | 0.00 (0.00, 1.06) | 0.00 (0.00, 2.20) |
| Gynaecological | Cervix | - | 0.00 (0.00, 2.20) |
| Gynaecological | Ovary | - | 0.00 (0.00, 2.20) |
| Gynaecological | Uterus | - | 0.00 (0.00, 2.20) |
| Gynaecological | Vulva/Vagina | - | 0.00 (0.00, 2.20) |
| Prostate and other male organs | Penile | 0.00 (0.00, 1.06) | - |
| Prostate and other male organs | Prostate | 0.00 (0.00, 1.06) | - |
| Prostate and other male organs | Testicular | 0.00 (0.00, 1.06) | - |
| Other malignant neoplasms | Other malignant neoplasms | 0.56 (0.15, 2.00) | 0.58 (0.10, 3.24) |
| Unknown primary | Unknown primary | 1.11 (0.43, 2.82) | 0.00 (0.00, 2.20) |
| *Respiratory - Stridor (Men=N<20, Women=N<20)* | | | |
| Head and neck | Larynx | - | - |
| Head and neck | Oral cavity | - | - |
| Head and neck | Oropharynx | - | - |
| Head and neck | Thyroid | - | - |
| Head and neck | Other head and neck | - | - |
| Upper gastrointestinal | Oesophagus | - | - |
| Upper gastrointestinal | Stomach | - | - |
| Lower gastrointestinal | Anal | - | - |
| Lower gastrointestinal | Colon | - | - |
| Lower gastrointestinal | Rectum | - | - |
| Lower gastrointestinal | Small intestine | - | - |
| Hepato-pancreato-biliary | Liver | - | - |
| Hepato-pancreato-biliary | Pancreas | - | - |
| Hepato-pancreato-biliary | Other Hepato-pancreato-biliary | - | - |
| Respiratory | Lung | - | - |
| Respiratory | Mesothelioma | - | - |
| Urological | Bladder | - | - |
| Urological | Kidney | - | - |
| Urological | Ureteric and other urinary | - | - |
| Haematological | Acute leukaemia | - | - |
| Haematological | Chronic lymphocytic leukaemia | - | - |
| Haematological | Hodgkin lymphoma | - | - |
| Haematological | Multiple myeloma | - | - |
| Haematological | Non-Hodgkin lymphoma | - | - |
| Haematological | Other haematological | - | - |
| Central nervous system | Central nervous system | - | - |
| Sarcoma | Bone sarcoma | - | - |
| Sarcoma | Connective and soft tissue sarcoma | - | - |
| Skin | Melanoma | - | - |
| Ocular | Ocular | - | - |
| Breast | Breast | - | - |
| Gynaecological | Cervix | - | - |
| Gynaecological | Ovary | - | - |
| Gynaecological | Uterus | - | - |
| Gynaecological | Vulva/Vagina | - | - |
| Prostate and other male organs | Penile | - | - |
| Prostate and other male organs | Prostate | - | - |
| Prostate and other male organs | Testicular | - | - |
| Other malignant neoplasms | Other malignant neoplasms | - | - |
| Unknown primary | Unknown primary | - | - |
| *Urological - Dysuria (Men=430, Women=169)* | | | |
| Head and neck | Larynx | 0.00 (0.00, 0.89) | 0.00 (0.00, 2.22) |
| Head and neck | Oral cavity | 0.00 (0.00, 0.89) | 0.00 (0.00, 2.22) |
| Head and neck | Oropharynx | 0.00 (0.00, 0.89) | 0.00 (0.00, 2.22) |
| Head and neck | Thyroid | 0.00 (0.00, 0.89) | 0.00 (0.00, 2.22) |
| Head and neck | Other head and neck | 0.00 (0.00, 0.89) | 0.00 (0.00, 2.22) |
| Upper gastrointestinal | Oesophagus | 0.00 (0.00, 0.89) | 0.00 (0.00, 2.22) |
| Upper gastrointestinal | Stomach | 0.70 (0.24, 2.03) | 0.59 (0.10, 3.28) |
| Lower gastrointestinal | Anal | 0.00 (0.00, 0.89) | 0.00 (0.00, 2.22) |
| Lower gastrointestinal | Colon | 1.40 (0.64, 3.01) | 3.55 (1.64, 7.53) |
| Lower gastrointestinal | Rectum | 0.00 (0.00, 0.89) | 0.59 (0.10, 3.28) |
| Lower gastrointestinal | Small intestine | 0.00 (0.00, 0.89) | 0.00 (0.00, 2.22) |
| Hepato-pancreato-biliary | Liver | 0.70 (0.24, 2.03) | 0.00 (0.00, 2.22) |
| Hepato-pancreato-biliary | Pancreas | 0.47 (0.13, 1.68) | 2.37 (0.92, 5.93) |
| Hepato-pancreato-biliary | Other Hepato-pancreato-biliary | 0.00 (0.00, 0.89) | 0.59 (0.10, 3.28) |
| Respiratory | Lung | 0.23 (0.04, 1.31) | 1.78 (0.61, 5.09) |
| Respiratory | Mesothelioma | 0.23 (0.04, 1.31) | 0.00 (0.00, 2.22) |
| Urological | Bladder | 18.6 (15.2, 22.6) | 36.1 (29.2, 43.6) |
| Urological | Kidney | 4.88 (3.22, 7.35) | 8.28 (5.00, 13.4) |
| Urological | Ureteric and other urinary | 1.63 (0.79, 3.32) | 2.96 (1.27, 6.74) |
| Haematological | Acute leukaemia | 0.47 (0.13, 1.68) | 1.18 (0.33, 4.21) |
| Haematological | Chronic lymphocytic leukaemia | 0.23 (0.04, 1.31) | 0.00 (0.00, 2.22) |
| Haematological | Hodgkin lymphoma | 0.00 (0.00, 0.89) | 0.00 (0.00, 2.22) |
| Haematological | Multiple myeloma | 0.23 (0.04, 1.31) | 0.00 (0.00, 2.22) |
| Haematological | Non-Hodgkin lymphoma | 0.00 (0.00, 0.89) | 1.78 (0.61, 5.09) |
| Haematological | Other haematological | 0.00 (0.00, 0.89) | 0.00 (0.00, 2.22) |
| Central nervous system | Central nervous system | 0.00 (0.00, 0.89) | 1.18 (0.33, 4.21) |
| Sarcoma | Bone sarcoma | 0.00 (0.00, 0.89) | 0.00 (0.00, 2.22) |
| Sarcoma | Connective and soft tissue sarcoma | 0.00 (0.00, 0.89) | 1.18 (0.33, 4.21) |
| Skin | Melanoma | 0.00 (0.00, 0.89) | 0.00 (0.00, 2.22) |
| Ocular | Ocular | 0.00 (0.00, 0.89) | 0.00 (0.00, 2.22) |
| Breast | Breast | 0.00 (0.00, 0.89) | 0.00 (0.00, 2.22) |
| Gynaecological | Cervix | - | 2.96 (1.27, 6.74) |
| Gynaecological | Ovary | - | 16.0 (11.2, 22.2) |
| Gynaecological | Uterus | - | 11.2 (7.32, 16.9) |
| Gynaecological | Vulva/Vagina | - | 5.33 (2.83, 9.81) |
| Prostate and other male organs | Penile | 1.40 (0.64, 3.01) | - |
| Prostate and other male organs | Prostate | 68.1 (63.6, 72.4) | - |
| Prostate and other male organs | Testicular | 0.00 (0.00, 0.89) | - |
| Other malignant neoplasms | Other malignant neoplasms | 0.23 (0.04, 1.31) | 0.59 (0.10, 3.28) |
| Unknown primary | Unknown primary | 0.47 (0.13, 1.68) | 1.78 (0.61, 5.09) |
| *Urological - Haematuria (Men=1386, Women=376)* | | | |
| Head and neck | Larynx | 0.00 (0.00, 0.28) | 0.00 (0.00, 1.01) |
| Head and neck | Oral cavity | 0.00 (0.00, 0.28) | 0.00 (0.00, 1.01) |
| Head and neck | Oropharynx | 0.00 (0.00, 0.28) | 0.00 (0.00, 1.01) |
| Head and neck | Thyroid | 0.00 (0.00, 0.28) | 0.00 (0.00, 1.01) |
| Head and neck | Other head and neck | 0.00 (0.00, 0.28) | 0.00 (0.00, 1.01) |
| Upper gastrointestinal | Oesophagus | 0.00 (0.00, 0.28) | 0.27 (0.05, 1.49) |
| Upper gastrointestinal | Stomach | 0.14 (0.04, 0.52) | 0.53 (0.15, 1.92) |
| Lower gastrointestinal | Anal | 0.00 (0.00, 0.28) | 0.00 (0.00, 1.01) |
| Lower gastrointestinal | Colon | 0.65 (0.34, 1.23) | 1.33 (0.57, 3.07) |
| Lower gastrointestinal | Rectum | 0.14 (0.04, 0.52) | 0.27 (0.05, 1.49) |
| Lower gastrointestinal | Small intestine | 0.07 (0.01, 0.41) | 0.27 (0.05, 1.49) |
| Hepato-pancreato-biliary | Liver | 0.65 (0.34, 1.23) | 0.00 (0.00, 1.01) |
| Hepato-pancreato-biliary | Pancreas | 0.51 (0.24, 1.04) | 0.53 (0.15, 1.92) |
| Hepato-pancreato-biliary | Other Hepato-pancreato-biliary | 0.22 (0.07, 0.63) | 0.00 (0.00, 1.01) |
| Respiratory | Lung | 0.94 (0.55, 1.60) | 1.33 (0.57, 3.07) |
| Respiratory | Mesothelioma | 0.07 (0.01, 0.41) | 0.00 (0.00, 1.01) |
| Urological | Bladder | 46.1 (43.5, 48.7) | 45.2 (40.3, 50.3) |
| Urological | Kidney | 13.8 (12.1, 15.7) | 17.0 (13.6, 21.1) |
| Urological | Ureteric and other urinary | 6.57 (5.38, 7.99) | 8.78 (6.32, 12.1) |
| Haematological | Acute leukaemia | 0.22 (0.07, 0.63) | 0.80 (0.27, 2.32) |
| Haematological | Chronic lymphocytic leukaemia | 0.29 (0.11, 0.74) | 0.00 (0.00, 1.01) |
| Haematological | Hodgkin lymphoma | 0.00 (0.00, 0.28) | 0.27 (0.05, 1.49) |
| Haematological | Multiple myeloma | 0.22 (0.07, 0.63) | 0.27 (0.05, 1.49) |
| Haematological | Non-Hodgkin lymphoma | 0.51 (0.24, 1.04) | 0.53 (0.15, 1.92) |
| Haematological | Other haematological | 0.07 (0.01, 0.41) | 0.00 (0.00, 1.01) |
| Central nervous system | Central nervous system | 0.00 (0.00, 0.28) | 0.00 (0.00, 1.01) |
| Sarcoma | Bone sarcoma | 0.00 (0.00, 0.28) | 0.00 (0.00, 1.01) |
| Sarcoma | Connective and soft tissue sarcoma | 0.07 (0.01, 0.41) | 0.27 (0.05, 1.49) |
| Skin | Melanoma | 0.00 (0.00, 0.28) | 0.00 (0.00, 1.01) |
| Ocular | Ocular | 0.00 (0.00, 0.28) | 0.00 (0.00, 1.01) |
| Breast | Breast | 0.07 (0.01, 0.41) | 0.00 (0.00, 1.01) |
| Gynaecological | Cervix | - | 2.66 (1.45, 4.83) |
| Gynaecological | Ovary | - | 2.66 (1.45, 4.83) |
| Gynaecological | Uterus | - | 15.7 (12.4, 19.7) |
| Gynaecological | Vulva/Vagina | - | 0.80 (0.27, 2.32) |
| Prostate and other male organs | Penile | 0.29 (0.11, 0.74) | - |
| Prostate and other male organs | Prostate | 27.6 (25.3, 30.0) | - |
| Prostate and other male organs | Testicular | 0.00 (0.00, 0.28) | - |
| Other malignant neoplasms | Other malignant neoplasms | 0.14 (0.04, 0.52) | 0.00 (0.00, 1.01) |
| Unknown primary | Unknown primary | 0.65 (0.34, 1.23) | 0.53 (0.15, 1.92) |
| *Urological - Loin pain (Men=180, Women=73)* | | | |
| Head and neck | Larynx | 0.00 (0.00, 2.09) | 0.00 (0.00, 5.00) |
| Head and neck | Oral cavity | 0.00 (0.00, 2.09) | 0.00 (0.00, 5.00) |
| Head and neck | Oropharynx | 0.00 (0.00, 2.09) | 0.00 (0.00, 5.00) |
| Head and neck | Thyroid | 0.00 (0.00, 2.09) | 0.00 (0.00, 5.00) |
| Head and neck | Other head and neck | 0.00 (0.00, 2.09) | 0.00 (0.00, 5.00) |
| Upper gastrointestinal | Oesophagus | 1.11 (0.31, 3.96) | 0.00 (0.00, 5.00) |
| Upper gastrointestinal | Stomach | 0.00 (0.00, 2.09) | 0.00 (0.00, 5.00) |
| Lower gastrointestinal | Anal | 0.00 (0.00, 2.09) | 0.00 (0.00, 5.00) |
| Lower gastrointestinal | Colon | 2.78 (1.19, 6.34) | 5.48 (2.15, 13.3) |
| Lower gastrointestinal | Rectum | 0.00 (0.00, 2.09) | 1.37 (0.24, 7.36) |
| Lower gastrointestinal | Small intestine | 1.11 (0.31, 3.96) | 0.00 (0.00, 5.00) |
| Hepato-pancreato-biliary | Liver | 1.11 (0.31, 3.96) | 1.37 (0.24, 7.36) |
| Hepato-pancreato-biliary | Pancreas | 1.11 (0.31, 3.96) | 2.74 (0.75, 9.45) |
| Hepato-pancreato-biliary | Other Hepato-pancreato-biliary | 0.00 (0.00, 2.09) | 0.00 (0.00, 5.00) |
| Respiratory | Lung | 3.33 (1.54, 7.08) | 8.22 (3.82, 16.8) |
| Respiratory | Mesothelioma | 0.00 (0.00, 2.09) | 0.00 (0.00, 5.00) |
| Urological | Bladder | 5.56 (3.05, 9.92) | 6.85 (2.96, 15.1) |
| Urological | Kidney | 29.4 (23.3, 36.5) | 28.8 (19.7, 40.0) |
| Urological | Ureteric and other urinary | 2.22 (0.87, 5.57) | 5.48 (2.15, 13.3) |
| Haematological | Acute leukaemia | 1.11 (0.31, 3.96) | 4.11 (1.41, 11.4) |
| Haematological | Chronic lymphocytic leukaemia | 0.00 (0.00, 2.09) | 0.00 (0.00, 5.00) |
| Haematological | Hodgkin lymphoma | 0.56 (0.10, 3.08) | 0.00 (0.00, 5.00) |
| Haematological | Multiple myeloma | 2.22 (0.87, 5.57) | 4.11 (1.41, 11.4) |
| Haematological | Non-Hodgkin lymphoma | 3.33 (1.54, 7.08) | 6.85 (2.96, 15.1) |
| Haematological | Other haematological | 0.56 (0.10, 3.08) | 1.37 (0.24, 7.36) |
| Central nervous system | Central nervous system | 0.56 (0.10, 3.08) | 0.00 (0.00, 5.00) |
| Sarcoma | Bone sarcoma | 0.00 (0.00, 2.09) | 0.00 (0.00, 5.00) |
| Sarcoma | Connective and soft tissue sarcoma | 0.56 (0.10, 3.08) | 2.74 (0.75, 9.45) |
| Skin | Melanoma | 0.00 (0.00, 2.09) | 0.00 (0.00, 5.00) |
| Ocular | Ocular | 0.00 (0.00, 2.09) | 0.00 (0.00, 5.00) |
| Breast | Breast | 0.00 (0.00, 2.09) | 1.37 (0.24, 7.36) |
| Gynaecological | Cervix | - | 0.00 (0.00, 5.00) |
| Gynaecological | Ovary | - | 16.4 (9.66, 26.6) |
| Gynaecological | Uterus | - | 1.37 (0.24, 7.36) |
| Gynaecological | Vulva/Vagina | - | 0.00 (0.00, 5.00) |
| Prostate and other male organs | Penile | 1.11 (0.31, 3.96) | - |
| Prostate and other male organs | Prostate | 38.3 (31.5, 45.6) | - |
| Prostate and other male organs | Testicular | 1.67 (0.57, 4.78) | - |
| Other malignant neoplasms | Other malignant neoplasms | 0.56 (0.10, 3.08) | 0.00 (0.00, 5.00) |
| Unknown primary | Unknown primary | 1.67 (0.57, 4.78) | 1.37 (0.24, 7.36) |
| *Urological - LUTS (nocturia, frequency, hesitancy, urgency, retention) (Men=4470, Women=233)* | | | |
| Head and neck | Larynx | 0.00 (0.00, 0.09) | 0.00 (0.00, 1.62) |
| Head and neck | Oral cavity | 0.00 (0.00, 0.09) | 0.00 (0.00, 1.62) |
| Head and neck | Oropharynx | 0.02 (0.00, 0.13) | 0.00 (0.00, 1.62) |
| Head and neck | Thyroid | 0.00 (0.00, 0.09) | 0.43 (0.08, 2.39) |
| Head and neck | Other head and neck | 0.00 (0.00, 0.09) | 0.00 (0.00, 1.62) |
| Upper gastrointestinal | Oesophagus | 0.02 (0.00, 0.13) | 0.00 (0.00, 1.62) |
| Upper gastrointestinal | Stomach | 0.02 (0.00, 0.13) | 0.00 (0.00, 1.62) |
| Lower gastrointestinal | Anal | 0.04 (0.01, 0.16) | 0.00 (0.00, 1.62) |
| Lower gastrointestinal | Colon | 0.60 (0.42, 0.88) | 3.00 (1.46, 6.07) |
| Lower gastrointestinal | Rectum | 0.36 (0.22, 0.58) | 0.43 (0.08, 2.39) |
| Lower gastrointestinal | Small intestine | 0.04 (0.01, 0.16) | 0.00 (0.00, 1.62) |
| Hepato-pancreato-biliary | Liver | 0.11 (0.05, 0.26) | 1.29 (0.44, 3.72) |
| Hepato-pancreato-biliary | Pancreas | 0.18 (0.09, 0.35) | 3.00 (1.46, 6.07) |
| Hepato-pancreato-biliary | Other Hepato-pancreato-biliary | 0.02 (0.00, 0.13) | 0.00 (0.00, 1.62) |
| Respiratory | Lung | 0.34 (0.20, 0.55) | 6.01 (3.61, 9.83) |
| Respiratory | Mesothelioma | 0.00 (0.00, 0.09) | 0.00 (0.00, 1.62) |
| Urological | Bladder | 4.09 (3.55, 4.72) | 23.6 (18.6, 29.5) |
| Urological | Kidney | 1.59 (1.26, 2.00) | 7.73 (4.94, 11.9) |
| Urological | Ureteric and other urinary | 0.47 (0.31, 0.72) | 2.15 (0.92, 4.92) |
| Haematological | Acute leukaemia | 0.04 (0.01, 0.16) | 0.00 (0.00, 1.62) |
| Haematological | Chronic lymphocytic leukaemia | 0.11 (0.05, 0.26) | 0.00 (0.00, 1.62) |
| Haematological | Hodgkin lymphoma | 0.04 (0.01, 0.16) | 0.00 (0.00, 1.62) |
| Haematological | Multiple myeloma | 0.22 (0.12, 0.41) | 0.00 (0.00, 1.62) |
| Haematological | Non-Hodgkin lymphoma | 0.49 (0.33, 0.74) | 2.15 (0.92, 4.92) |
| Haematological | Other haematological | 0.04 (0.01, 0.16) | 0.43 (0.08, 2.39) |
| Central nervous system | Central nervous system | 0.04 (0.01, 0.16) | 0.86 (0.24, 3.08) |
| Sarcoma | Bone sarcoma | 0.00 (0.00, 0.09) | 0.00 (0.00, 1.62) |
| Sarcoma | Connective and soft tissue sarcoma | 0.02 (0.00, 0.13) | 1.29 (0.44, 3.72) |
| Skin | Melanoma | 0.02 (0.00, 0.13) | 0.43 (0.08, 2.39) |
| Ocular | Ocular | 0.02 (0.00, 0.13) | 0.00 (0.00, 1.62) |
| Breast | Breast | 0.00 (0.00, 0.09) | 0.43 (0.08, 2.39) |
| Gynaecological | Cervix | - | 1.72 (0.67, 4.33) |
| Gynaecological | Ovary | - | 28.8 (23.3, 34.9) |
| Gynaecological | Uterus | - | 9.44 (6.32, 13.9) |
| Gynaecological | Vulva/Vagina | - | 2.58 (1.19, 5.50) |
| Prostate and other male organs | Penile | 0.22 (0.12, 0.41) | - |
| Prostate and other male organs | Prostate | 90.5 (89.6, 91.3) | - |
| Prostate and other male organs | Testicular | 0.13 (0.06, 0.29) | - |
| Other malignant neoplasms | Other malignant neoplasms | 0.04 (0.01, 0.16) | 0.86 (0.24, 3.08) |
| Unknown primary | Unknown primary | 0.11 (0.05, 0.26) | 3.43 (1.75, 6.63) |
| *Urological - Renal colic (Men=51, Women=N<20)* | | | |
| Head and neck | Larynx | 0.00 (0.00, 7.00) | - |
| Head and neck | Oral cavity | 0.00 (0.00, 7.00) | - |
| Head and neck | Oropharynx | 0.00 (0.00, 7.00) | - |
| Head and neck | Thyroid | 0.00 (0.00, 7.00) | - |
| Head and neck | Other head and neck | 0.00 (0.00, 7.00) | - |
| Upper gastrointestinal | Oesophagus | 1.96 (0.35, 10.3) | - |
| Upper gastrointestinal | Stomach | 0.00 (0.00, 7.00) | - |
| Lower gastrointestinal | Anal | 0.00 (0.00, 7.00) | - |
| Lower gastrointestinal | Colon | 1.96 (0.35, 10.3) | - |
| Lower gastrointestinal | Rectum | 0.00 (0.00, 7.00) | - |
| Lower gastrointestinal | Small intestine | 1.96 (0.35, 10.3) | - |
| Hepato-pancreato-biliary | Liver | 0.00 (0.00, 7.00) | - |
| Hepato-pancreato-biliary | Pancreas | 1.96 (0.35, 10.3) | - |
| Hepato-pancreato-biliary | Other Hepato-pancreato-biliary | 0.00 (0.00, 7.00) | - |
| Respiratory | Lung | 7.84 (3.09, 18.5) | - |
| Respiratory | Mesothelioma | 0.00 (0.00, 7.00) | - |
| Urological | Bladder | 1.96 (0.35, 10.3) | - |
| Urological | Kidney | 41.2 (28.8, 54.8) | - |
| Urological | Ureteric and other urinary | 3.92 (1.08, 13.2) | - |
| Haematological | Acute leukaemia | 1.96 (0.35, 10.3) | - |
| Haematological | Chronic lymphocytic leukaemia | 0.00 (0.00, 7.00) | - |
| Haematological | Hodgkin lymphoma | 0.00 (0.00, 7.00) | - |
| Haematological | Multiple myeloma | 0.00 (0.00, 7.00) | - |
| Haematological | Non-Hodgkin lymphoma | 1.96 (0.35, 10.3) | - |
| Haematological | Other haematological | 0.00 (0.00, 7.00) | - |
| Central nervous system | Central nervous system | 0.00 (0.00, 7.00) | - |
| Sarcoma | Bone sarcoma | 0.00 (0.00, 7.00) | - |
| Sarcoma | Connective and soft tissue sarcoma | 0.00 (0.00, 7.00) | - |
| Skin | Melanoma | 0.00 (0.00, 7.00) | - |
| Ocular | Ocular | 0.00 (0.00, 7.00) | - |
| Breast | Breast | 0.00 (0.00, 7.00) | - |
| Gynaecological | Cervix | - | - |
| Gynaecological | Ovary | - | - |
| Gynaecological | Uterus | - | - |
| Gynaecological | Vulva/Vagina | - | - |
| Prostate and other male organs | Penile | 0.00 (0.00, 7.00) | - |
| Prostate and other male organs | Prostate | 31.4 (20.3, 45.0) | - |
| Prostate and other male organs | Testicular | 0.00 (0.00, 7.00) | - |
| Other malignant neoplasms | Other malignant neoplasms | 0.00 (0.00, 7.00) | - |
| Unknown primary | Unknown primary | 1.96 (0.35, 10.3) | - |
| *Urological - Urinary tract infection (Men=383, Women=238)* | | | |
| Head and neck | Larynx | 0.00 (0.00, 0.99) | 0.00 (0.00, 1.59) |
| Head and neck | Oral cavity | 0.00 (0.00, 0.99) | 0.00 (0.00, 1.59) |
| Head and neck | Oropharynx | 0.00 (0.00, 0.99) | 0.00 (0.00, 1.59) |
| Head and neck | Thyroid | 0.00 (0.00, 0.99) | 0.00 (0.00, 1.59) |
| Head and neck | Other head and neck | 0.00 (0.00, 0.99) | 0.00 (0.00, 1.59) |
| Upper gastrointestinal | Oesophagus | 0.00 (0.00, 0.99) | 0.00 (0.00, 1.59) |
| Upper gastrointestinal | Stomach | 0.00 (0.00, 0.99) | 1.26 (0.43, 3.64) |
| Lower gastrointestinal | Anal | 0.00 (0.00, 0.99) | 0.00 (0.00, 1.59) |
| Lower gastrointestinal | Colon | 2.09 (1.06, 4.07) | 4.20 (2.30, 7.56) |
| Lower gastrointestinal | Rectum | 0.00 (0.00, 0.99) | 0.84 (0.23, 3.01) |
| Lower gastrointestinal | Small intestine | 0.00 (0.00, 0.99) | 0.00 (0.00, 1.59) |
| Hepato-pancreato-biliary | Liver | 0.78 (0.27, 2.28) | 1.26 (0.43, 3.64) |
| Hepato-pancreato-biliary | Pancreas | 0.78 (0.27, 2.28) | 3.36 (1.71, 6.49) |
| Hepato-pancreato-biliary | Other Hepato-pancreato-biliary | 0.26 (0.05, 1.46) | 0.42 (0.07, 2.34) |
| Respiratory | Lung | 0.26 (0.05, 1.46) | 3.36 (1.71, 6.49) |
| Respiratory | Mesothelioma | 0.00 (0.00, 0.99) | 0.00 (0.00, 1.59) |
| Urological | Bladder | 19.3 (15.7, 23.6) | 39.1 (33.1, 45.4) |
| Urological | Kidney | 5.48 (3.61, 8.24) | 15.1 (11.1, 20.2) |
| Urological | Ureteric and other urinary | 1.83 (0.89, 3.72) | 4.20 (2.30, 7.56) |
| Haematological | Acute leukaemia | 0.26 (0.05, 1.46) | 2.10 (0.90, 4.82) |
| Haematological | Chronic lymphocytic leukaemia | 0.52 (0.14, 1.88) | 0.00 (0.00, 1.59) |
| Haematological | Hodgkin lymphoma | 0.00 (0.00, 0.99) | 0.00 (0.00, 1.59) |
| Haematological | Multiple myeloma | 0.52 (0.14, 1.88) | 0.00 (0.00, 1.59) |
| Haematological | Non-Hodgkin lymphoma | 1.57 (0.72, 3.38) | 1.26 (0.43, 3.64) |
| Haematological | Other haematological | 0.00 (0.00, 0.99) | 0.00 (0.00, 1.59) |
| Central nervous system | Central nervous system | 0.00 (0.00, 0.99) | 0.42 (0.07, 2.34) |
| Sarcoma | Bone sarcoma | 0.00 (0.00, 0.99) | 0.00 (0.00, 1.59) |
| Sarcoma | Connective and soft tissue sarcoma | 0.26 (0.05, 1.46) | 0.42 (0.07, 2.34) |
| Skin | Melanoma | 0.00 (0.00, 0.99) | 0.00 (0.00, 1.59) |
| Ocular | Ocular | 0.00 (0.00, 0.99) | 0.00 (0.00, 1.59) |
| Breast | Breast | 0.00 (0.00, 0.99) | 0.84 (0.23, 3.01) |
| Gynaecological | Cervix | - | 2.94 (1.43, 5.95) |
| Gynaecological | Ovary | - | 7.98 (5.17, 12.1) |
| Gynaecological | Uterus | - | 7.56 (4.84, 11.6) |
| Gynaecological | Vulva/Vagina | - | 0.84 (0.23, 3.01) |
| Prostate and other male organs | Penile | 0.52 (0.14, 1.88) | - |
| Prostate and other male organs | Prostate | 64.8 (59.8, 69.4) | - |
| Prostate and other male organs | Testicular | 0.26 (0.05, 1.46) | - |
| Other malignant neoplasms | Other malignant neoplasms | 0.00 (0.00, 0.99) | 0.42 (0.07, 2.34) |
| Unknown primary | Unknown primary | 0.52 (0.14, 1.88) | 2.10 (0.90, 4.82) |
| *Central nervous system - Fit/seizure (Men=94, Women=69)* | | | |
| Head and neck | Larynx | 0.00 (0.00, 3.93) | 0.00 (0.00, 5.27) |
| Head and neck | Oral cavity | 0.00 (0.00, 3.93) | 0.00 (0.00, 5.27) |
| Head and neck | Oropharynx | 0.00 (0.00, 3.93) | 1.45 (0.26, 7.76) |
| Head and neck | Thyroid | 0.00 (0.00, 3.93) | 1.45 (0.26, 7.76) |
| Head and neck | Other head and neck | 0.00 (0.00, 3.93) | 0.00 (0.00, 5.27) |
| Upper gastrointestinal | Oesophagus | 0.00 (0.00, 3.93) | 0.00 (0.00, 5.27) |
| Upper gastrointestinal | Stomach | 1.06 (0.19, 5.78) | 0.00 (0.00, 5.27) |
| Lower gastrointestinal | Anal | 0.00 (0.00, 3.93) | 0.00 (0.00, 5.27) |
| Lower gastrointestinal | Colon | 0.00 (0.00, 3.93) | 1.45 (0.26, 7.76) |
| Lower gastrointestinal | Rectum | 0.00 (0.00, 3.93) | 0.00 (0.00, 5.27) |
| Lower gastrointestinal | Small intestine | 0.00 (0.00, 3.93) | 0.00 (0.00, 5.27) |
| Hepato-pancreato-biliary | Liver | 0.00 (0.00, 3.93) | 0.00 (0.00, 5.27) |
| Hepato-pancreato-biliary | Pancreas | 0.00 (0.00, 3.93) | 0.00 (0.00, 5.27) |
| Hepato-pancreato-biliary | Other Hepato-pancreato-biliary | 0.00 (0.00, 3.93) | 1.45 (0.26, 7.76) |
| Respiratory | Lung | 17.0 (10.8, 25.9) | 10.1 (5.00, 19.5) |
| Respiratory | Mesothelioma | 0.00 (0.00, 3.93) | 0.00 (0.00, 5.27) |
| Urological | Bladder | 0.00 (0.00, 3.93) | 0.00 (0.00, 5.27) |
| Urological | Kidney | 2.13 (0.59, 7.43) | 0.00 (0.00, 5.27) |
| Urological | Ureteric and other urinary | 0.00 (0.00, 3.93) | 0.00 (0.00, 5.27) |
| Haematological | Acute leukaemia | 0.00 (0.00, 3.93) | 0.00 (0.00, 5.27) |
| Haematological | Chronic lymphocytic leukaemia | 0.00 (0.00, 3.93) | 0.00 (0.00, 5.27) |
| Haematological | Hodgkin lymphoma | 0.00 (0.00, 3.93) | 0.00 (0.00, 5.27) |
| Haematological | Multiple myeloma | 0.00 (0.00, 3.93) | 2.90 (0.80, 9.97) |
| Haematological | Non-Hodgkin lymphoma | 2.13 (0.59, 7.43) | 2.90 (0.80, 9.97) |
| Haematological | Other haematological | 0.00 (0.00, 3.93) | 0.00 (0.00, 5.27) |
| Central nervous system | Central nervous system | 74.5 (64.8, 82.2) | 66.7 (54.9, 76.6) |
| Sarcoma | Bone sarcoma | 0.00 (0.00, 3.93) | 0.00 (0.00, 5.27) |
| Sarcoma | Connective and soft tissue sarcoma | 0.00 (0.00, 3.93) | 0.00 (0.00, 5.27) |
| Skin | Melanoma | 0.00 (0.00, 3.93) | 1.45 (0.26, 7.76) |
| Ocular | Ocular | 0.00 (0.00, 3.93) | 0.00 (0.00, 5.27) |
| Breast | Breast | 0.00 (0.00, 3.93) | 4.35 (1.49, 12.0) |
| Gynaecological | Cervix | - | 0.00 (0.00, 5.27) |
| Gynaecological | Ovary | - | 0.00 (0.00, 5.27) |
| Gynaecological | Uterus | - | 0.00 (0.00, 5.27) |
| Gynaecological | Vulva/Vagina | - | 0.00 (0.00, 5.27) |
| Prostate and other male organs | Penile | 0.00 (0.00, 3.93) | - |
| Prostate and other male organs | Prostate | 0.00 (0.00, 3.93) | - |
| Prostate and other male organs | Testicular | 1.06 (0.19, 5.78) | - |
| Other malignant neoplasms | Other malignant neoplasms | 1.06 (0.19, 5.78) | 0.00 (0.00, 5.27) |
| Unknown primary | Unknown primary | 1.06 (0.19, 5.78) | 5.80 (2.28, 14.0) |
| *Central nervous system - Headache (Men=201, Women=190)* | | | |
| Head and neck | Larynx | 0.50 (0.09, 2.76) | 0.00 (0.00, 1.98) |
| Head and neck | Oral cavity | 0.00 (0.00, 1.88) | 0.00 (0.00, 1.98) |
| Head and neck | Oropharynx | 1.49 (0.51, 4.30) | 1.58 (0.54, 4.54) |
| Head and neck | Thyroid | 0.50 (0.09, 2.76) | 0.53 (0.09, 2.92) |
| Head and neck | Other head and neck | 3.98 (2.03, 7.66) | 4.21 (2.15, 8.09) |
| Upper gastrointestinal | Oesophagus | 1.00 (0.27, 3.55) | 0.53 (0.09, 2.92) |
| Upper gastrointestinal | Stomach | 1.49 (0.51, 4.30) | 2.11 (0.82, 5.29) |
| Lower gastrointestinal | Anal | 0.00 (0.00, 1.88) | 0.00 (0.00, 1.98) |
| Lower gastrointestinal | Colon | 4.48 (2.37, 8.29) | 2.11 (0.82, 5.29) |
| Lower gastrointestinal | Rectum | 0.00 (0.00, 1.88) | 0.53 (0.09, 2.92) |
| Lower gastrointestinal | Small intestine | 0.00 (0.00, 1.88) | 0.53 (0.09, 2.92) |
| Hepato-pancreato-biliary | Liver | 1.49 (0.51, 4.30) | 0.00 (0.00, 1.98) |
| Hepato-pancreato-biliary | Pancreas | 1.49 (0.51, 4.30) | 1.05 (0.29, 3.76) |
| Hepato-pancreato-biliary | Other Hepato-pancreato-biliary | 0.50 (0.09, 2.76) | 1.05 (0.29, 3.76) |
| Respiratory | Lung | 17.4 (12.8, 23.3) | 26.8 (21.0, 33.6) |
| Respiratory | Mesothelioma | 0.00 (0.00, 1.88) | 0.00 (0.00, 1.98) |
| Urological | Bladder | 0.50 (0.09, 2.76) | 0.00 (0.00, 1.98) |
| Urological | Kidney | 1.99 (0.78, 5.00) | 0.53 (0.09, 2.92) |
| Urological | Ureteric and other urinary | 0.00 (0.00, 1.88) | 0.00 (0.00, 1.98) |
| Haematological | Acute leukaemia | 3.48 (1.70, 7.01) | 3.68 (1.80, 7.41) |
| Haematological | Chronic lymphocytic leukaemia | 0.50 (0.09, 2.76) | 0.53 (0.09, 2.92) |
| Haematological | Hodgkin lymphoma | 1.00 (0.27, 3.55) | 1.05 (0.29, 3.76) |
| Haematological | Multiple myeloma | 2.49 (1.07, 5.69) | 1.58 (0.54, 4.54) |
| Haematological | Non-Hodgkin lymphoma | 5.47 (3.08, 9.53) | 5.79 (3.26, 10.1) |
| Haematological | Other haematological | 1.00 (0.27, 3.55) | 1.05 (0.29, 3.76) |
| Central nervous system | Central nervous system | 39.3 (32.8, 46.2) | 36.8 (30.3, 43.9) |
| Sarcoma | Bone sarcoma | 0.00 (0.00, 1.88) | 0.53 (0.09, 2.92) |
| Sarcoma | Connective and soft tissue sarcoma | 0.00 (0.00, 1.88) | 0.53 (0.09, 2.92) |
| Skin | Melanoma | 1.00 (0.27, 3.55) | 0.00 (0.00, 1.98) |
| Ocular | Ocular | 0.00 (0.00, 1.88) | 0.53 (0.09, 2.92) |
| Breast | Breast | 0.00 (0.00, 1.88) | 2.11 (0.82, 5.29) |
| Gynaecological | Cervix | - | 0.00 (0.00, 1.98) |
| Gynaecological | Ovary | - | 0.53 (0.09, 2.92) |
| Gynaecological | Uterus | - | 0.53 (0.09, 2.92) |
| Gynaecological | Vulva/Vagina | - | 1.05 (0.29, 3.76) |
| Prostate and other male organs | Penile | 0.00 (0.00, 1.88) | - |
| Prostate and other male organs | Prostate | 4.48 (2.37, 8.29) | - |
| Prostate and other male organs | Testicular | 0.00 (0.00, 1.88) | - |
| Other malignant neoplasms | Other malignant neoplasms | 0.50 (0.09, 2.76) | 0.00 (0.00, 1.98) |
| Unknown primary | Unknown primary | 3.98 (2.03, 7.66) | 2.11 (0.82, 5.29) |
| *Central nervous system - Prog/sub-acute loss of central neuro funct (Men=263, Women=209)* | | | |
| Head and neck | Larynx | 0.00 (0.00, 1.44) | 0.00 (0.00, 1.80) |
| Head and neck | Oral cavity | 0.00 (0.00, 1.44) | 0.00 (0.00, 1.80) |
| Head and neck | Oropharynx | 0.38 (0.07, 2.12) | 0.00 (0.00, 1.80) |
| Head and neck | Thyroid | 0.00 (0.00, 1.44) | 0.00 (0.00, 1.80) |
| Head and neck | Other head and neck | 1.52 (0.59, 3.84) | 0.00 (0.00, 1.80) |
| Upper gastrointestinal | Oesophagus | 0.38 (0.07, 2.12) | 0.48 (0.08, 2.66) |
| Upper gastrointestinal | Stomach | 0.00 (0.00, 1.44) | 0.00 (0.00, 1.80) |
| Lower gastrointestinal | Anal | 0.00 (0.00, 1.44) | 0.00 (0.00, 1.80) |
| Lower gastrointestinal | Colon | 1.52 (0.59, 3.84) | 1.44 (0.49, 4.13) |
| Lower gastrointestinal | Rectum | 0.38 (0.07, 2.12) | 0.96 (0.26, 3.42) |
| Lower gastrointestinal | Small intestine | 0.38 (0.07, 2.12) | 0.00 (0.00, 1.80) |
| Hepato-pancreato-biliary | Liver | 0.76 (0.21, 2.73) | 0.48 (0.08, 2.66) |
| Hepato-pancreato-biliary | Pancreas | 1.52 (0.59, 3.84) | 0.96 (0.26, 3.42) |
| Hepato-pancreato-biliary | Other Hepato-pancreato-biliary | 0.38 (0.07, 2.12) | 0.48 (0.08, 2.66) |
| Respiratory | Lung | 19.4 (15.1, 24.6) | 25.8 (20.4, 32.2) |
| Respiratory | Mesothelioma | 0.38 (0.07, 2.12) | 0.00 (0.00, 1.80) |
| Urological | Bladder | 0.00 (0.00, 1.44) | 0.48 (0.08, 2.66) |
| Urological | Kidney | 1.14 (0.39, 3.30) | 2.39 (1.03, 5.48) |
| Urological | Ureteric and other urinary | 0.00 (0.00, 1.44) | 0.00 (0.00, 1.80) |
| Haematological | Acute leukaemia | 0.00 (0.00, 1.44) | 0.96 (0.26, 3.42) |
| Haematological | Chronic lymphocytic leukaemia | 0.00 (0.00, 1.44) | 0.96 (0.26, 3.42) |
| Haematological | Hodgkin lymphoma | 0.00 (0.00, 1.44) | 0.00 (0.00, 1.80) |
| Haematological | Multiple myeloma | 1.52 (0.59, 3.84) | 1.44 (0.49, 4.13) |
| Haematological | Non-Hodgkin lymphoma | 4.56 (2.63, 7.80) | 5.74 (3.31, 9.77) |
| Haematological | Other haematological | 0.38 (0.07, 2.12) | 1.44 (0.49, 4.13) |
| Central nervous system | Central nervous system | 55.1 (49.1, 61.0) | 45.9 (39.3, 52.7) |
| Sarcoma | Bone sarcoma | 0.00 (0.00, 1.44) | 0.48 (0.08, 2.66) |
| Sarcoma | Connective and soft tissue sarcoma | 0.38 (0.07, 2.12) | 0.00 (0.00, 1.80) |
| Skin | Melanoma | 2.28 (1.05, 4.89) | 0.48 (0.08, 2.66) |
| Ocular | Ocular | 0.00 (0.00, 1.44) | 0.00 (0.00, 1.80) |
| Breast | Breast | 0.00 (0.00, 1.44) | 2.39 (1.03, 5.48) |
| Gynaecological | Cervix | - | 0.00 (0.00, 1.80) |
| Gynaecological | Ovary | - | 0.48 (0.08, 2.66) |
| Gynaecological | Uterus | - | 0.48 (0.08, 2.66) |
| Gynaecological | Vulva/Vagina | - | 0.00 (0.00, 1.80) |
| Prostate and other male organs | Penile | 0.00 (0.00, 1.44) | - |
| Prostate and other male organs | Prostate | 1.14 (0.39, 3.30) | - |
| Prostate and other male organs | Testicular | 0.38 (0.07, 2.12) | - |
| Other malignant neoplasms | Other malignant neoplasms | 0.76 (0.21, 2.73) | 0.00 (0.00, 1.80) |
| Unknown primary | Unknown primary | 5.32 (3.20, 8.74) | 5.74 (3.31, 9.77) |
| Central nervous system - Visual disturbance or loss (Men=75, Women=58) | | | |
| Head and neck | Larynx | 0.00 (0.00, 4.87) | 0.00 (0.00, 6.21) |
| Head and neck | Oral cavity | 0.00 (0.00, 4.87) | 0.00 (0.00, 6.21) |
| Head and neck | Oropharynx | 0.00 (0.00, 4.87) | 0.00 (0.00, 6.21) |
| Head and neck | Thyroid | 0.00 (0.00, 4.87) | 0.00 (0.00, 6.21) |
| Head and neck | Other head and neck | 6.67 (2.88, 14.7) | 1.72 (0.31, 9.14) |
| Upper gastrointestinal | Oesophagus | 0.00 (0.00, 4.87) | 0.00 (0.00, 6.21) |
| Upper gastrointestinal | Stomach | 0.00 (0.00, 4.87) | 0.00 (0.00, 6.21) |
| Lower gastrointestinal | Anal | 0.00 (0.00, 4.87) | 0.00 (0.00, 6.21) |
| Lower gastrointestinal | Colon | 1.33 (0.24, 7.17) | 0.00 (0.00, 6.21) |
| Lower gastrointestinal | Rectum | 0.00 (0.00, 4.87) | 0.00 (0.00, 6.21) |
| Lower gastrointestinal | Small intestine | 0.00 (0.00, 4.87) | 0.00 (0.00, 6.21) |
| Hepato-pancreato-biliary | Liver | 0.00 (0.00, 4.87) | 0.00 (0.00, 6.21) |
| Hepato-pancreato-biliary | Pancreas | 0.00 (0.00, 4.87) | 0.00 (0.00, 6.21) |
| Hepato-pancreato-biliary | Other Hepato-pancreato-biliary | 0.00 (0.00, 4.87) | 0.00 (0.00, 6.21) |
| Respiratory | Lung | 14.7 (8.39, 24.4) | 19.0 (10.9, 30.9) |
| Respiratory | Mesothelioma | 0.00 (0.00, 4.87) | 0.00 (0.00, 6.21) |
| Urological | Bladder | 0.00 (0.00, 4.87) | 0.00 (0.00, 6.21) |
| Urological | Kidney | 0.00 (0.00, 4.87) | 3.45 (0.95, 11.7) |
| Urological | Ureteric and other urinary | 0.00 (0.00, 4.87) | 0.00 (0.00, 6.21) |
| Haematological | Acute leukaemia | 0.00 (0.00, 4.87) | 0.00 (0.00, 6.21) |
| Haematological | Chronic lymphocytic leukaemia | 2.67 (0.73, 9.21) | 0.00 (0.00, 6.21) |
| Haematological | Hodgkin lymphoma | 0.00 (0.00, 4.87) | 0.00 (0.00, 6.21) |
| Haematological | Multiple myeloma | 1.33 (0.24, 7.17) | 1.72 (0.31, 9.14) |
| Haematological | Non-Hodgkin lymphoma | 9.33 (4.59, 18.0) | 6.90 (2.71, 16.4) |
| Haematological | Other haematological | 2.67 (0.73, 9.21) | 1.72 (0.31, 9.14) |
| Central nervous system | Central nervous system | 38.7 (28.5, 50.0) | 32.8 (22.1, 45.6) |
| Sarcoma | Bone sarcoma | 0.00 (0.00, 4.87) | 0.00 (0.00, 6.21) |
| Sarcoma | Connective and soft tissue sarcoma | 0.00 (0.00, 4.87) | 0.00 (0.00, 6.21) |
| Skin | Melanoma | 1.33 (0.24, 7.17) | 0.00 (0.00, 6.21) |
| Ocular | Ocular | 13.3 (7.41, 22.8) | 27.6 (17.8, 40.2) |
| Breast | Breast | 0.00 (0.00, 4.87) | 1.72 (0.31, 9.14) |
| Gynaecological | Cervix | - | 0.00 (0.00, 6.21) |
| Gynaecological | Ovary | - | 1.72 (0.31, 9.14) |
| Gynaecological | Uterus | - | 0.00 (0.00, 6.21) |
| Gynaecological | Vulva/Vagina | - | 0.00 (0.00, 6.21) |
| Prostate and other male organs | Penile | 0.00 (0.00, 4.87) | - |
| Prostate and other male organs | Prostate | 6.67 (2.88, 14.7) | - |
| Prostate and other male organs | Testicular | 0.00 (0.00, 4.87) | - |
| Other malignant neoplasms | Other malignant neoplasms | 0.00 (0.00, 4.87) | 0.00 (0.00, 6.21) |
| Unknown primary | Unknown primary | 1.33 (0.24, 7.17) | 1.72 (0.31, 9.14) |
| *Musculoskeletal - Back pain (Men=1007, Women=688)* | | | |
| Head and neck | Larynx | 0.00 (0.00, 0.38) | 0.00 (0.00, 0.56) |
| Head and neck | Oral cavity | 0.10 (0.02, 0.56) | 0.00 (0.00, 0.56) |
[truncated: 63,960 more chars]
