# Supplementary material for: The presenting symptom signatures of incident cancer: evidence from the English 2018 National Cancer Diagnosis Audit
Source: Br J Cancer. 2023 Dec 6;130(2):297–307. doi: 10.1038/s41416-023-02507-4 (PMC10803766; doi:10.1038/s41416-023-02507-4)
Supplement: Supplementary file 2 — STROBE checklist [file 41416_2023_2507_MOESM2_ESM.docx]

STROBE Statement—Checklist of items that should be included in reports of ***cross-sectional studies***

|  | Item No | Recommendation | Page No |
| --- | --- | --- | --- |
| **Title and abstract** | 1 | (*a*) Indicate the study’s design with a commonly used term in the title or the abstract | Page 1 / the term ‘audit’ implies a cross-sectional survey design. |
|  |  | (*b*) Provide in the abstract an informative and balanced summary of what was done and what was found | Please see Abstract, page 2. |
| Introduction | | | |
| Background/rationale | 2 | Explain the scientific background and rationale for the investigation being reported | Please see Introduction, para 1 – the scientific background and rationale are provided. |
| Objectives | 3 | State specific objectives, including any prespecified hypotheses | Please see Introduction, para 2 – where objectives are provided. |
| Methods | | | |
| Study design | 4 | Present key elements of study design early in the paper | Please see Methods, para 1. |
| Setting | 5 | Describe the setting, locations, and relevant dates, including periods of recruitment, exposure, follow-up, and data collection | Please see Methods, para 1-3. |
| Participants | 6 | (*a*) Give the eligibility criteria, and the sources and methods of selection of participants | Please see Methods, para 1. |
| Variables | 7 | Clearly define all outcomes, exposures, predictors, potential confounders, and effect modifiers. Give diagnostic criteria, if applicable | Please see Methods, para 2 and 3. Symptoms / symptom groups and cancer sites / cancer site groups were the main variables of interest. There was no adjustment for these bivariate associations, given the research object. |
| Data sources/ measurement | 8* | For each variable of interest, give sources of data and details of methods of assessment (measurement). Describe comparability of assessment methods if there is more than one group | Please see Methods, para 2 and 3. |
| Bias | 9 | Describe any efforts to address potential sources of bias | Bias may be arising from missing symptom data – there was no attempt to handle this issue statistically though such data are treated as a explicit category. |
| Study size | 10 | Explain how the study size was arrived at | The study design and size is guided by the research question and the cross-sectional nature of the data. |
| Quantitative variables | 11 | Explain how quantitative variables were handled in the analyses. If applicable, describe which groupings were chosen and why | All available categories are used as provided by the data source; in addition a higher level aggregation of symptom groups, and of cancer groups, was also implemented. |
| Statistical methods | 12 | (*a*) Describe all statistical methods, including those used to control for confounding | The approach taken was descriptive, as applicable for ‘discovery’ studies of this type. As such no formal statistical analysis was undertaken. |
|  |  | (*b*) Describe any methods used to examine subgroups and interactions | Not applicable. |
|  |  | (*c*) Explain how missing data were addressed | Patients with missing data (e.g. not known symptom) were described, no statistical handling was used. |
|  |  | (*d*) If applicable, describe analytical methods taking account of sampling strategy | There was no sampling as such, as all the available data were used. |
|  |  | (*e*) Describe any sensitivity analyses | None. |
| Results | | | |
| Participants | 13* | (a) Report numbers of individuals at each stage of study—eg numbers potentially eligible, examined for eligibility, confirmed eligible, included in the study, completing follow-up, and analysed | Please see para 1 in results and figure 1. |
|  |  | (b) Give reasons for non-participation at each stage | Please see Figure 1. |
|  |  | (c) Consider use of a flow diagram | Please see Figure 1. |
| Descriptive data | 14* | (a) Give characteristics of study participants (eg demographic, clinical, social) and information on exposures and potential confounders | Please see para 1 in results and table 1. |
|  |  | (b) Indicate number of participants with missing data for each variable of interest | Not applicable. |
| Outcome data | 15* | Report numbers of outcome events or summary measures | Please see “Symptom signatures” and “Cancer site case-mix” paragraphs in results. |
| Main results | 16 | (*a*) Give unadjusted estimates and, if applicable, confounder-adjusted estimates and their precision (eg, 95% confidence interval). Make clear which confounders were adjusted for and why they were included | Not applicable – all estimates are unadjusted |
|  |  | (*b*) Report category boundaries when continuous variables were categorized | Not applicable. |
|  |  | (*c*) If relevant, consider translating estimates of relative risk into absolute risk for a meaningful time period | Not applicable. |
| Other analyses | 17 | Report other analyses done—eg analyses of subgroups and interactions, and sensitivity analyses | Not applicable. |
| Discussion | | | |
| Key results | 18 | Summarise key results with reference to study objectives | Please see para 1 in Discussion. |
| Limitations | 19 | Discuss limitations of the study, taking into account sources of potential bias or imprecision. Discuss both direction and magnitude of any potential bias | Please see “Strengths and limitations” paragraph in Discussion. |
| Interpretation | 20 | Give a cautious overall interpretation of results considering objectives, limitations, multiplicity of analyses, results from similar studies, and other relevant evidence | Please see “Comparisons with literature” paragraph in Discussion. |
| Generalisability | 21 | Discuss the generalisability (external validity) of the study results | Please see “Implications” paragraph in Discussion. |
| Other information | | | |
| Funding | 22 | Give the source of funding and the role of the funders for the present study and, if applicable, for the original study on which the present article is based | Please see “Funding information” section on page 14. |

*Give information separately for exposed and unexposed groups.

**Note:** An Explanation and Elaboration article discusses each checklist item and gives methodological background and published examples of transparent reporting. The STROBE checklist is best used in conjunction with this article (freely available on the Web sites of PLoS Medicine at http://www.plosmedicine.org/, Annals of Internal Medicine at http://www.annals.org/, and Epidemiology at http://www.epidem.com/). Information on the STROBE Initiative is available at www.strobe-statement.org.
